# Design, Synthesis and Evaluation of 2,5-Diketopiperazines as Inhibitors of the MDM2-p53 Interaction

**DOI:** 10.1371/journal.pone.0137867

**Published:** 2015-10-01

**Authors:** Mariell Pettersson, Maria Quant, Jaeki Min, Luigi Iconaru, Richard W. Kriwacki, M. Brett Waddell, R. Kiplin Guy, Kristina Luthman, Morten Grøtli

**Affiliations:** 1 Department of Chemistry and Molecular Biology, University of Gothenburg, 412 96, Gothenburg, Sweden; 2 Department of Chemical Biology and Therapeutics, St. Jude Children’s Research Hospital, 262 Danny Thomas Place, Memphis, Tennessee, 38105, United States of America; 3 Department of Structural Biology, St. Jude Children’s Research Hospital, 262 Danny Thomas Place, Memphis, Tennessee, 38105, United States of America; 4 Molecular Interaction Analysis Shared Resource, St. Jude Children’s Research Hospital, 262 Danny Thomas Place, Memphis, Tennessee, 38105, United States of America; University of East Anglia, UNITED KINGDOM

## Abstract

The transcription factor p53 is the main tumour suppressor in cells and many cancer types have p53 mutations resulting in a loss of its function. In tumours that retain wild-type p53 function, p53 activity is down-regulated by MDM2 (human murine double minute 2) *via* a direct protein—protein interaction. We have designed and synthesised two series of 2,5-diketopiperazines as inhibitors of the MDM2-p53 interaction. The first set was designed to directly mimic the α-helical region of the p53 peptide, containing key residues in the *i*, *i+4* and *i+7* positions of a natural α-helix. Conformational analysis indicated that 1,3,6-trisubstituted 2,5-diketopiperazines were able to place substituents in the same spatial orientation as an α-helix template. The key step of the synthesis involved the cyclisation of substituted dipeptides. The other set of tetrasubstituted 2,5-diketopiperazines were designed based on structure-based docking studies and the Ugi multicomponent reaction was used for the synthesis. This latter set comprised the most potent inhibitors which displayed micromolar IC_50_-values in a biochemical fluorescence polarisation assay.

## Introduction

The tumour suppressor protein p53 plays a crucial role in many physiological processes [1−5]. TP53 (the gene encoding the p53 protein) is mutated or deleted in almost 50% of all human cancers, resulting in non-functional p53 [6,7]. In the remaining 50% of human cancers, the wild-type p53 is occasionally effectively inhibited by overexpression of an endogenous negative regulator called MDM2 [8]. MDM2 ubiquitinates p53 leading to the proteasomal degradation of p53 [9]. In a complex with p53, MDM2 also blocks the binding of p53 to its target DNA, making p53 ineffective as a transcription factor. It also promotes the export of p53 from the cell nucleus, making p53 inaccessible to targeted DNA and reducing its transcriptional ability.

Disruption of the MDM2-p53 protein-protein interaction would liberate p53 from MDM2, thus restoring the tumour suppressor function of wild-type p53. Agents designed to block the MDM2-p53 interaction may therefore have therapeutic potential for the treatment of human cancers retaining wild-type p53 [10].

Structural studies have been utilised to characterise the interaction between a hydrophobic pocket within the *N*-terminal region of MDM2 and p53 [11]. The MDM2-bound p53 peptide adopts an α-helical conformation and interacts with MDM2 primarily through three hydrophobic residues: Phe19, Trp23, and Leu26.

**Fig 1:**
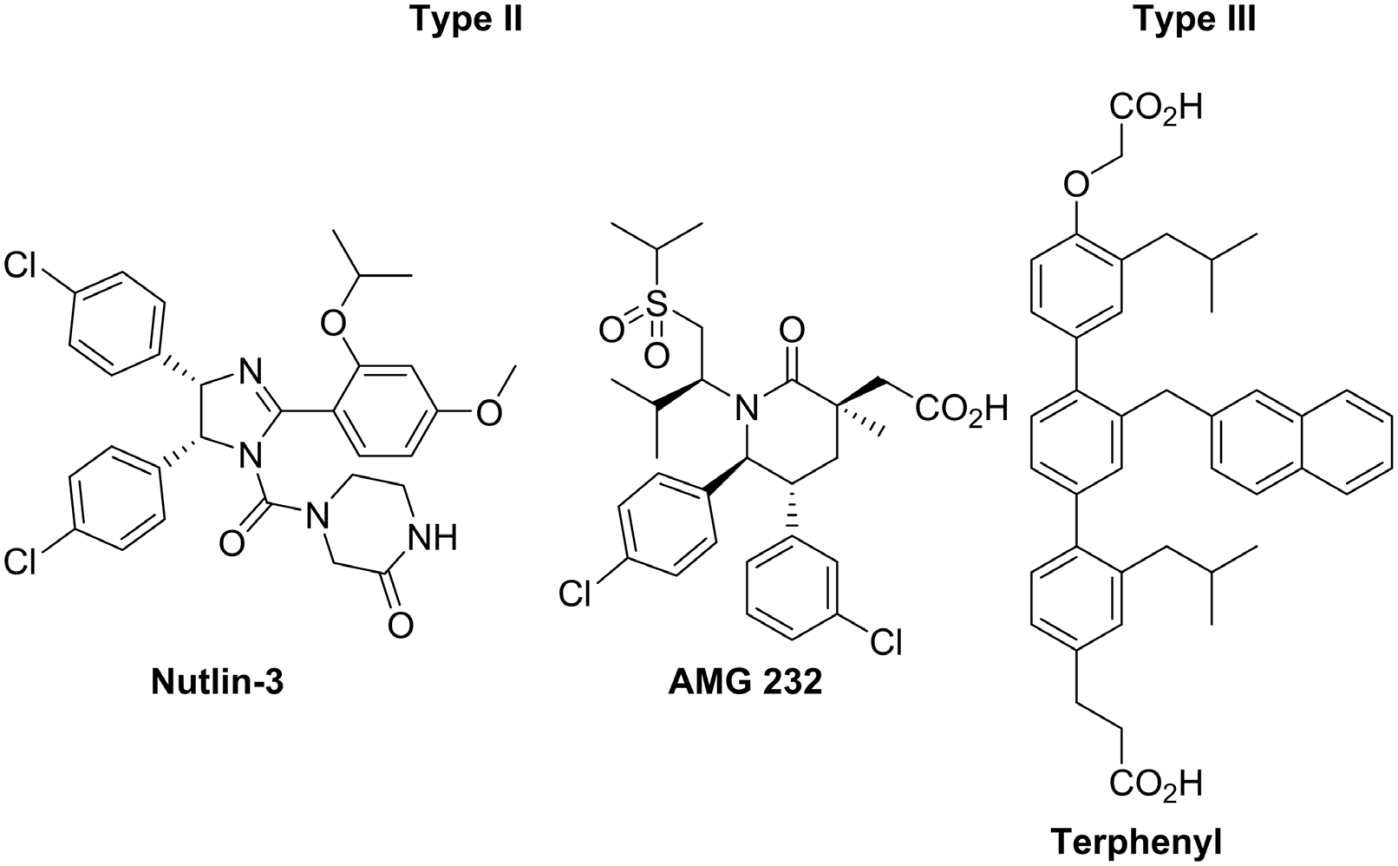
Examples of type II and III inhibitors previously reported.

Several inhibitors which target the MDM2-p53 interaction have been published [12]. These inhibitors can be divided into three groups: type I, II and III [13]. Type I inhibitors are peptide oligomers designed to mimic the α-helical topography. Type II inhibitors are based on scaffolds that place substituents in the same spatial orientation as that of the parent helix, but the scaffolds themselves are not designed to mimic the α-helix topography. These scaffolds vary widely in structure, but all can arrange the substituents in an analogous manner with the *i*, *i*+4 and *i*+7 amino acid side chains of an α-helical structure. Representative examples of type II inhibitors targeting the MDM2-p53 interaction include the nutlins [14], piperidinones [15] (Fig 1) and spiroindolines [16]

Most of the reported type II inhibitors have been identified by high throughput screening (HTS), followed by optimisation. Type III inhibitors or α-helix mimetics are characterised by highly modified structures which may not contain the basic peptide backbone structure, but still retain the functional groups necessary for key binding site recognition (the *i*, *i*+4 and *i*+7 amino acid side chains of an α-helical structure). The terphenyl scaffold reported by Hamilton and co-workers is the first example of this type of mimetic (Fig 1) [17–18]. Other relevant examples of type III inhibitors targeting the MDM2-p53 interaction include oxazole-pyridazine-piperazine mimetics [19], oligobenzamide mimetics [20] and pyrrolopyrimidine-based α-helix mimetics [21]. Although the MDM2-p53 interaction has been the focus of considerable investigation, a range of different PPIs have been targeted with type I-III inhibitors [22–24]

The synthesis and functionalisation of 2,5-diketopiperazines (2,5-DKPs) has been of continuous interest within our research group [25–28] because of their designation as privileged structures [29]. Pursuant to our interest in this area, we herein present the design, synthesis, and biological evaluation of 2,5-DKP derivatives as potential MDM2-p53 inhibitors.

## Results and Discussion

### Design of type III inhibitors

**Fig 2:**
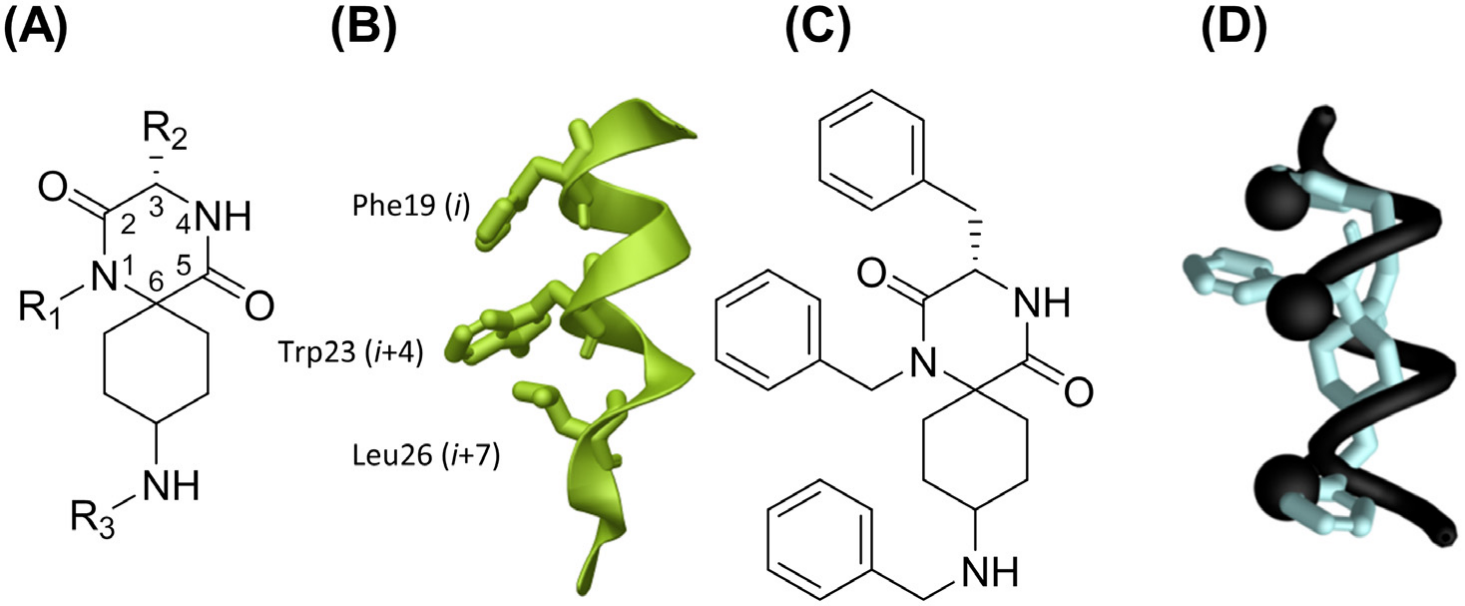
Spiro-DKPs as potential α-helix mimetics. (**A**) General structure of the target spiro-DKPs with numbering. (**B**) The side chains of **Leu26**, **Trp23**, and **Phe19** in the **p53** helix.(**C**) Spiro-DKPs. (**D**) Superimposition of an Ala-helix (black), with a low energy conformation of a spiro-DKP B (green).

It was anticipated that spiro-DKPs (Fig 2A) would be a suitable starting point for the development of novel type III inhibitors, as these shape-programmable scaffolds can project functional groups into defined three-dimensional constellations to mimic the positioning of the relevant side chains of p53 (Phe19, Trp23, and Leu26) (Fig 2B).

Conformational analysis was used to evaluate spiro-DKPs as potential α-helix mimetics. The comparison of low energy conformations of spiro-DKPs (Fig 2C) with an alanine-based α-helix indicated that the scaffold has the ability to arrange the substituents R_1-3_ analogous to the *i*, *i*+4 and *i*+7 residues from the α-helix (Fig 2D). This demands an *S*-configuration at the C3-position of the spiro-DKP and that the amino function is positioned in an equatorial position.

**Fig 3:**
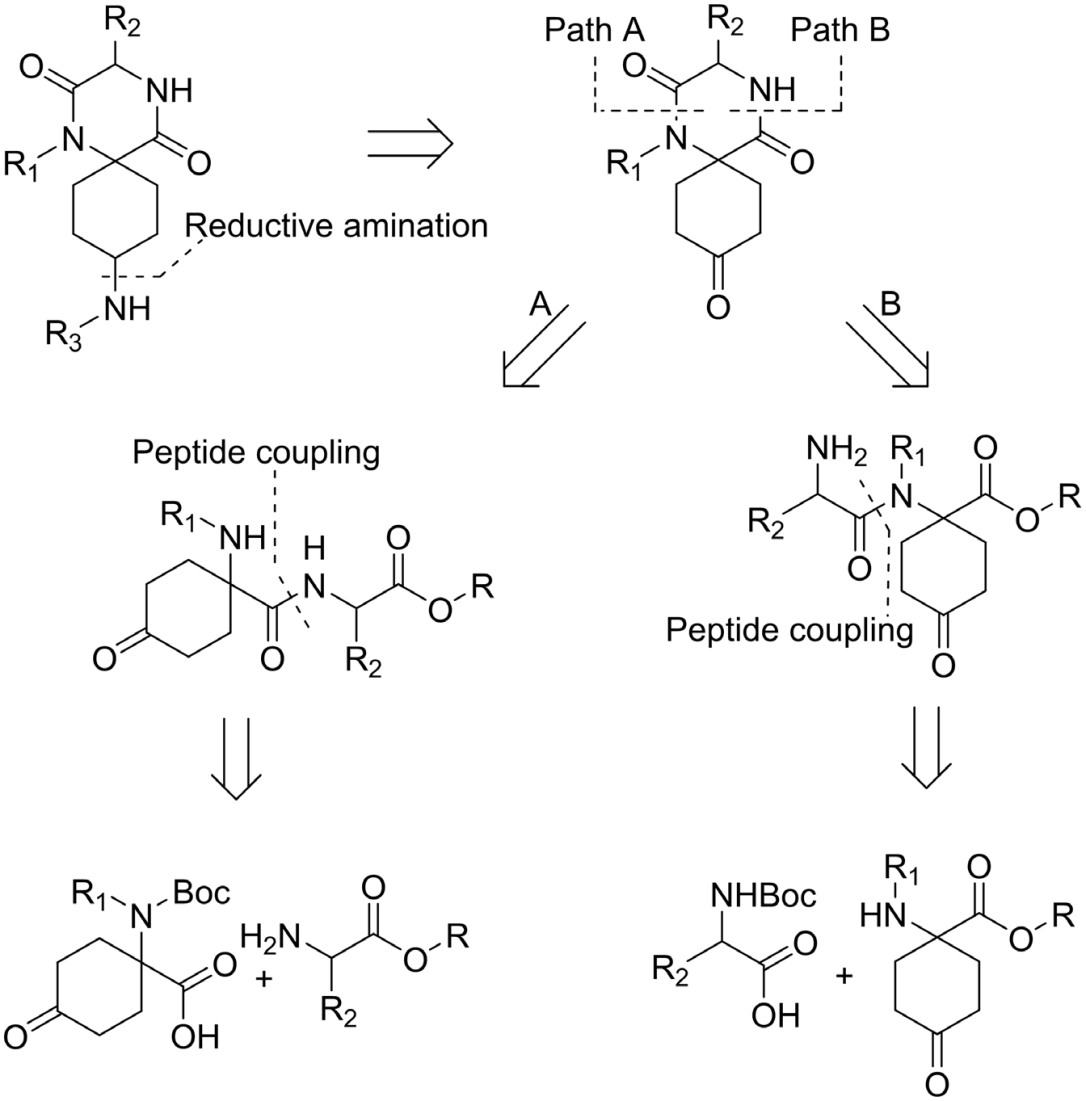
Retrosynthetic analysis of spiro-DKPs.

A retrosynthetic analysis for the synthesis of target spiro-DKPs is outlined in Fig 3. It was envisioned that the R_3_ substituent could be introduced *via* reductive amination in the final step of the synthesis. The formation of the 2,5-DKP-core could be achieved *via* cyclisation using a secondary amine (path A) or a primary amine (path B) as a nucleophile. The dipeptide could be obtained by peptide coupling of the appropriate amino acids.

### Synthesis of Type III inhibitors

The key residues required for MDM2-p53 binding are hydrophobic (Phe, Trp and Leu); therefore, hydrophobic R_1-3_ substituents were selected.

**Fig 4:**
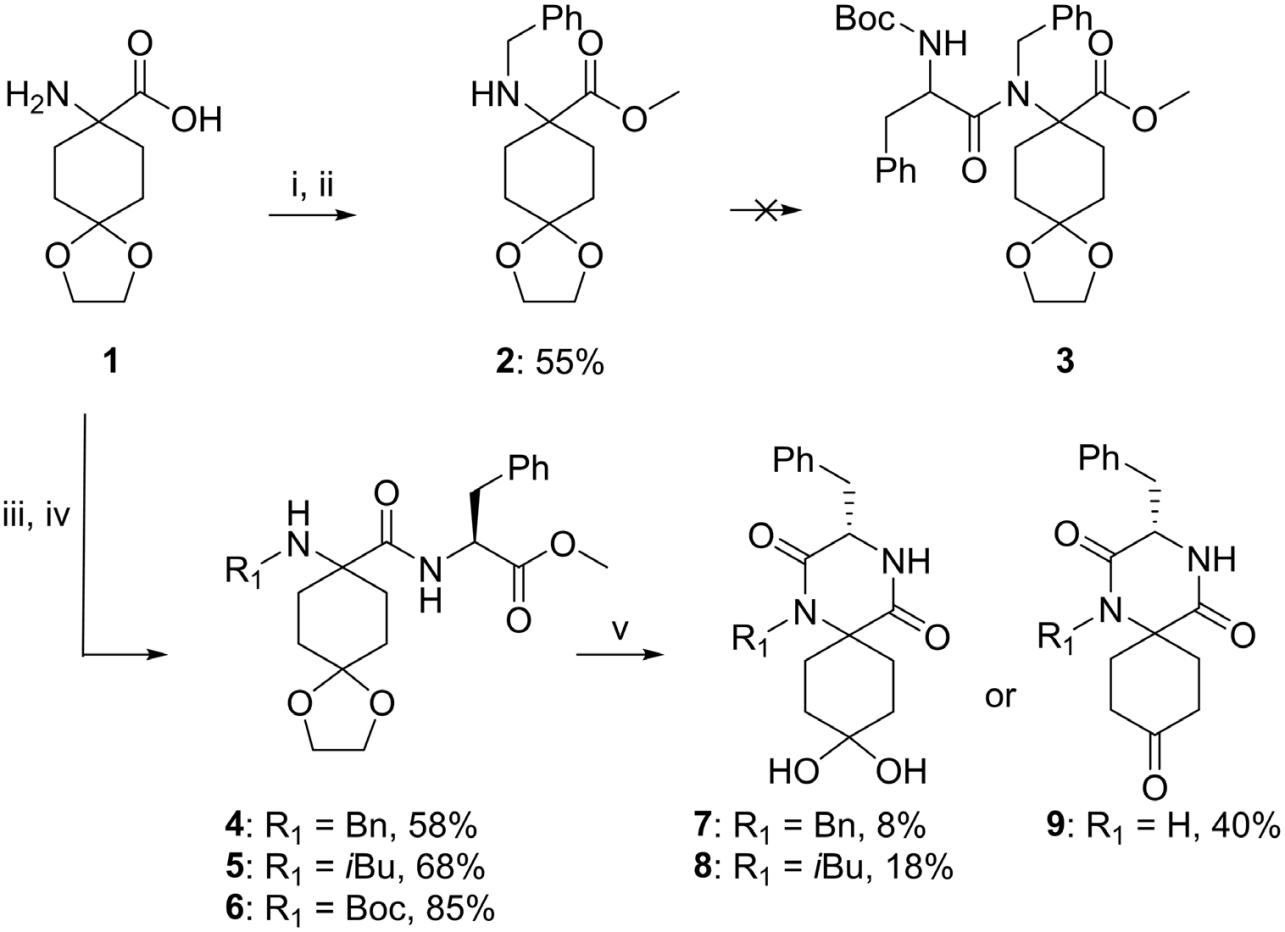
Synthesis of spiro-DKPs 7–9. Reagents and reaction conditions: i) PhCHO (1.2 eq.), Et_3_N (1.2 eq.), NaCNBH_3_ (1.0 eq.), MeOH, r.t. ii) (CH_3_)_3_SiCHN_2_ (6.4 eq.), MeOH/toluene (1:3), r.t. iii) **4** or **5**: R_1_CHO (1.2–1.5 eq.), Et_3_N (1.2 eq.), NaCNBH_3_ (1.0 eq.), MeOH, r.t. iv) Phe-OMe (2.0 eq.), HATU (2.0 eq.), DIPEA (12 eq.), DMF, 60°C, 30 min. **6**: iii) Boc_2_O, 3M NaOH and 1,4-dioxane (1:2, pH~12), r.t. iv) Phe-OMe (2.0 eq.), HATU (2.0 eq.), DIPEA (6.0 eq.), DMF, 60°C, 30 min. v) **4**: water, MW, 160°C, 30 min; **5**: HCl (1M, aq.)/acetone (1:1), 55°C, 72 h **6**: water, MW, 160°C, 90 min.

Initially it was attempted to prepare the spiro-DKPs by path A (Fig 3), using commercially available 8-amino-1,4-dioxa-spiro[4.5]decane-8-carboxylic acid (**1**) as a starting material (Fig 4). The benzyl substituent (R_1_) was introduced *via* a reductive amination protocol [30] with benzaldehyde, NaCNBH_3_ and Et_3_N as a base. The product was identified by LCMS analysis and the crude product was used in the next step without further purification. Conversion of the carboxylic acid to the corresponding methyl ester with trimetylsilyldiazomethane [31], afforded **2** in a yield of 55% over two synthetic steps.

The coupling of Boc-Phe to **2** in order to obtain **3** was then explored using different peptide-coupling reagents [32], such as HATU, EDC/HOBt and T3P; however, only starting material was recovered from the reaction mixture. The lack of reactivity under the explored reaction conditions could probably be ascribed to the steric hindrance of the amine.

It was then decided to explore the alternative path B for the cyclisation (Fig 3), starting from the same starting material as for path A. The R_1_ substituent was introduced using the same reductive amination protocol shown in Fig 4, followed by a HATU-mediated peptide coupling using Phe-OMe (Fig 4). Compounds **4** and **5** were isolated in yields of 58% and 68%, respectively, over two steps. We have previously reported a microwave heated synthesis of spiro-DKPs *via* cyclisation of Boc-protected dipeptide methyl esters using water as solvent [26]. It was anticipated that these reaction conditions would result in cyclisation of the dipeptides to afford the corresponding spiro-DKPs as well as the removal of both the acetal- and Boc-protecting groups. However, LCMS analysis following microwave-assisted heating of **4** in distilled water at 100°C for 30 min showed only trace amounts of **7**. The major mass ions observed corresponded to products derived from hydrolysis of the ester and/or the acetal. Following an increase of both the reaction temperature, to 160°C, and prolonged reaction time, to 90 min, **7** was isolated in 8% yield. Further increasing the temperature or reaction time did not improve the yield.

Using a 1:1 mixture of 1M HCl (aq.) and acetone as the solvent, at 55°C (conventional heating) for 72 h afforded **8** in a yield of 18%. LCMS analysis of the reaction mixture indicated that hydrolysis of the methyl ester prevented the efficient conversion of the starting material to the desired product. ^13^C NMR spectra of **7** and **8** showed C-4 signals at 93.0 and 92.9 ppm, respectively, indicating that the products were geminal diols and not ketones. HRMS analysis of **7** and **8** confirmed the diol structure.

The low yield observed for the cyclisation step was ascribed to the steric bulk of the secondary amine; therefore, it was decided to explore the same reaction with a primary amine. Compound **6** was synthesised in 85% yield over two steps (Fig 4). Heating of **6** under microwave irradiation at 160°C for 30 min using distilled water as solvent afforded **9** in 40% isolated yield. For **9**, only the ketone was observed in ^13^C NMR spectra. Changing solvent to a 1:1 mixture of 1M HCl (aq.) and acetone did not improve the yield.

**Fig 5:**
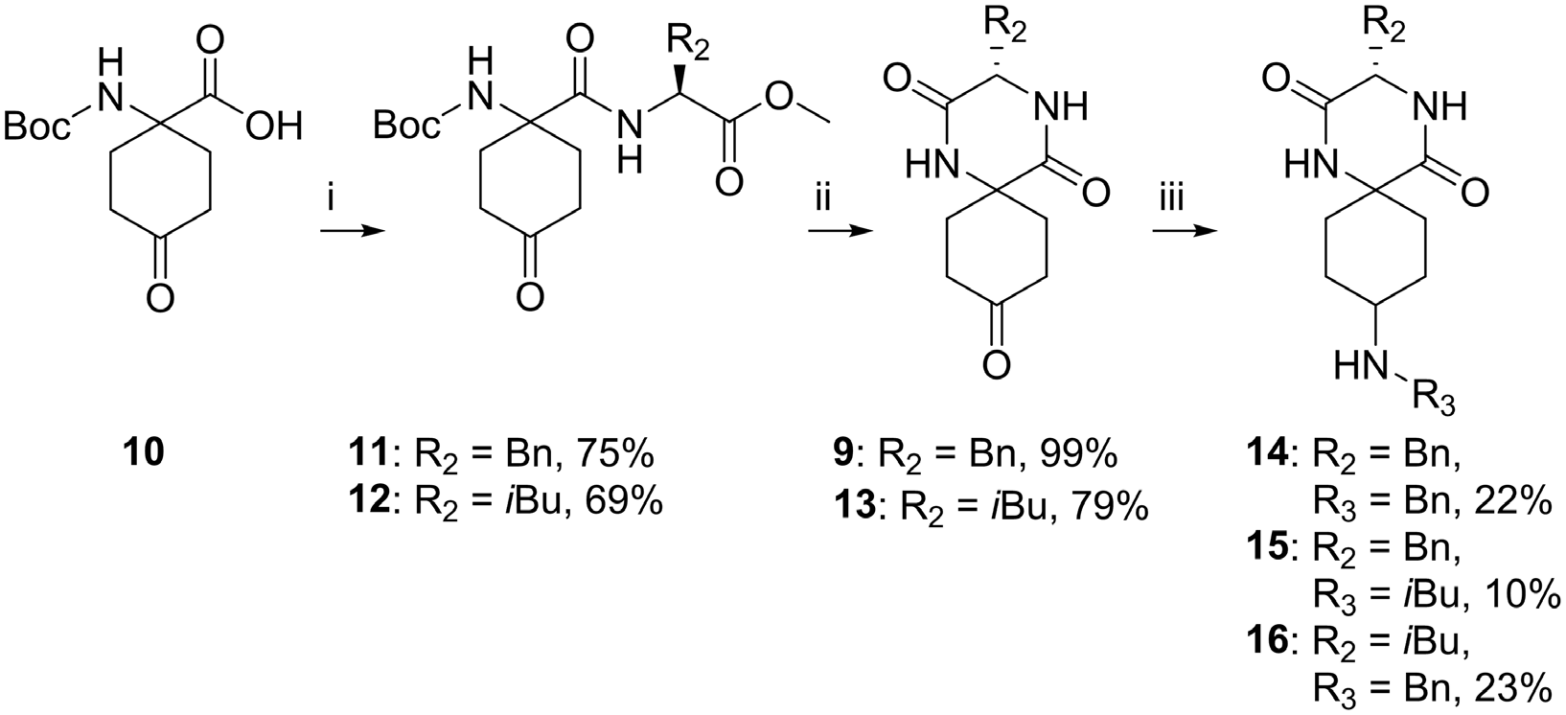
Synthesis of spiro-DKPs 9–16. Reagents and reaction conditions: i) AA-OMe (1.5–1.7 eq.). HATU (2.0 eq.), DIPEA (6.0 eq.), DMF, 60°C, 30 min. ii) water, MW, 160°C, 70 min. iii) R_3_-NH_2_ (1.7–2.0 eq.), PEMB (1.0 eq.), glacial acetic acid (2.3–2.6 eq.), MeOH, r.t. o.n.

At this stage, it was decided to synthesise a series of spiro-DKPs without the R_1_ substituent. Since side-products in which the acetal-protecting group was retained had been observed after cyclisation, the removal of acetal before the cyclisation was examined (Fig 5). Compounds **11** and **12** were prepared from commercially available **10**, in yields of 75% and 69%, respectively, using HATU as the coupling agent. Subsequent cyclisation in water under microwave assisted heating followed by evaporation of water and purification by flash column chromatography generated **9** and **13** in high yields.

Finally, the R_3_ substituent was introduced by reductive amination using a 5-ethyl-2-methylpyridine borane (PEMB) protocol (Fig 5) [33]. LCMS analysis indicated full consumption of the starting materials, but together with the desired product, unidentified by-products were also observed. Isolation of **14**–**16** from the complex reaction mixture resulted in only low yields (10–23%).

### Biological evaluation of type III inhibitors against the MDM2-p53 PPI

Compounds **7–9** and **14–16** were evaluated as MDM2 inhibitors in a fluorescence polarisation (FP) assay which measures displacement of a wild-type p53 peptide tagged with a fluorescent probe (Texas Red) bound to MDM2 [34]. Unfortunately, the compounds showed no activity. Re-evaluation of the design was therefore performed, aiming for type II inhibitors.

### Design of type II inhibitors

During our studies with type III inhibitors, new crystal structures of MDM2 co-crystallised with highly potent type II inhibitors were published [15,35–37]. Analysis of these as well as previously published crystal structures [10] of ligand/MDM2 complexes suggested several modifications to our original design in order to generate type II inhibitors. First, the size of the inhibitor could be decreased, since type II inhibitors are generally smaller and not as extended as type III inhibitors. Secondly, the size of one or two of the hydrophobic substituents should be reduced. Lastly, several of the published binders of MDM2 have a hydrophilic substituent pointing towards the bulk solvent/hydrophilic surface of MDM2 enabling hydrogen bonding and/or ionic interactions with the His96 and Lys94 residues of MDM2.

**Fig 6:**
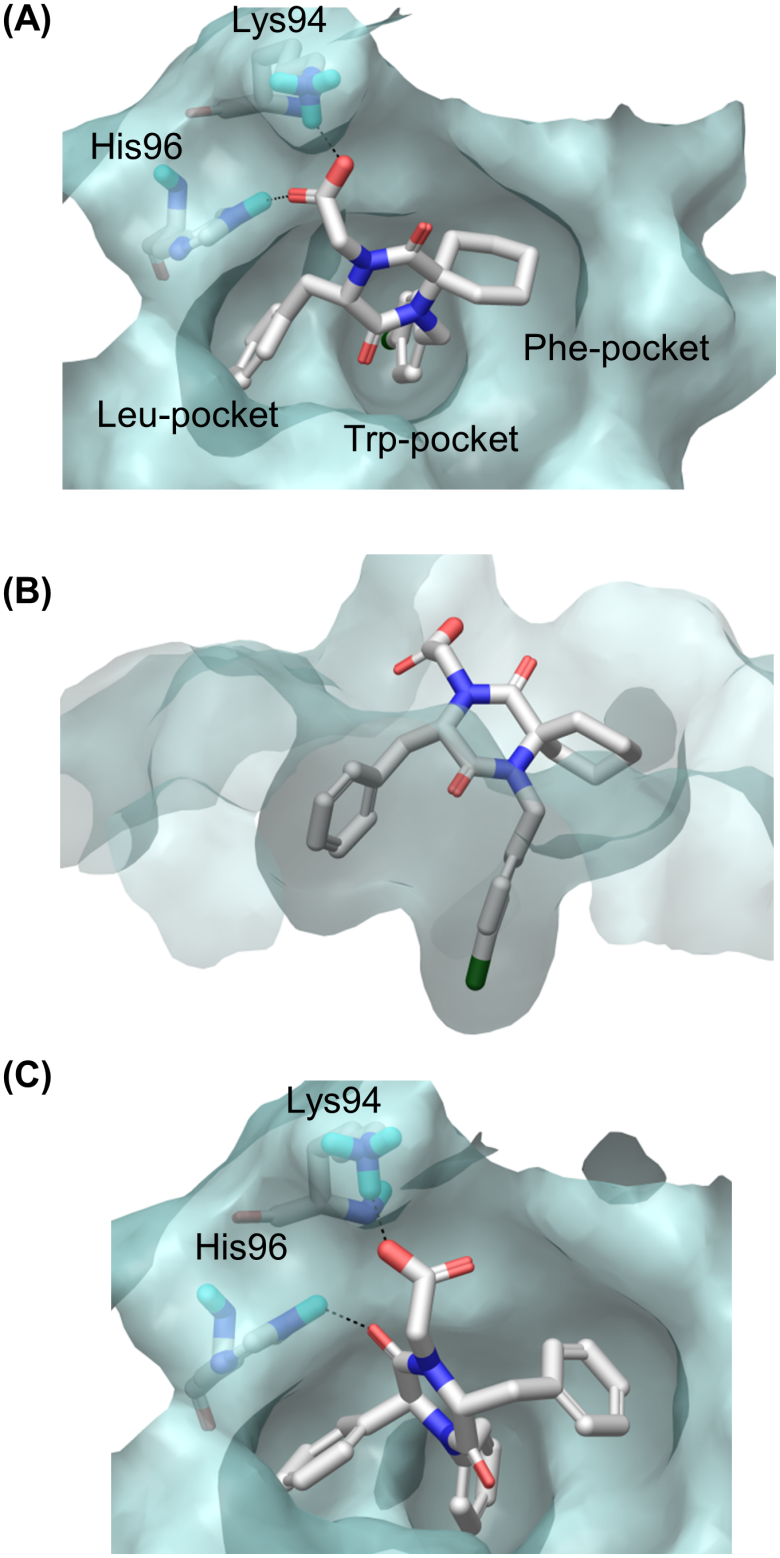
2,5-DKP derivatives docked into the α-helix binding site of MDM2 (PDB code: 4HBM). (**A**) and (**B**) *N*1 = 4-chlorobenzyl, C3 = Benzyl, *N*4 = CH_2_CO_2_H, C6 = cyclohexyl. (**C**) *N*1 = C6 = Phenyl, C3 = benzyl, *N*4 = CH_2_CO_2_H.

A series of structurally varied 2,5-DKP derivatives was docked into the α-helix binding site of MDM2 (PDB code: 4HBM), using the Schrödinger package (Glide, XP mode), to find a suitable substitution pattern on the 2,5-DKP scaffold (Fig 6). The docking results indicated that the spiro-cyclohexyl group was well accommodated in the Phe-binding pocket (Fig 6A and 6B) and could work as one of the interacting hydrophobic substituents. Likewise, exchanging the cyclohexyl group for a phenyl group (Fig 6C), gave compounds that docked equally well in the Trp-pocket compared to the spiro-cyclohexyl derivatives. The model did however show that the DKP ring is rotated 90°, switching the places of the C3 and C6 substituents, so that they now interact with the Phe- and Leu-pocket, respectively. Replacing the *N*1 benzyl group with a phenyl group resulted in compounds which had similar binding modes. The docking results also suggested the introduction of a CH_2_X group at the *N*4-position, where X = methyl ester, carboxylic acid, amide, or alcohol could provide additional interactions *via* hydrogen bonding and/or ionic interactions with the His96 and Lys94 residues of MDM2 (Fig 6A and 6C).

**Fig 7:**
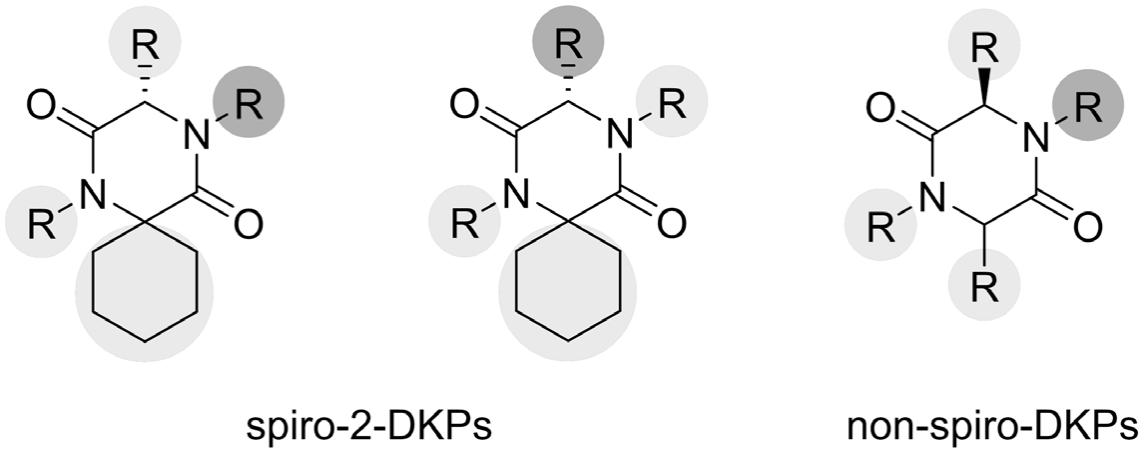
General structures of spiro-2-DKPs and non-spiro-DKPs. Hydrophobic substituents are indicated by light grey, while hydrophilic substituents are shown in dark grey.

Furthermore, docking results showed that derivatives with the hydrophilic substituent in the C3-position and one of the interacting hydrophobic substituents in the *N*4-position were also accommodated in the MDM2-pockets. The model indicated that at the C3-position, the *S*-configuration was preferred for the new series of spiro-DKPs (spiro-2-DKPs), while the *R*-configuration was preferred for the derivatives having phenyl substituents at the C6-position (non-spiro-DKPs). Two series of 2,5-DKPs, spiro-2-DKPs and non-spiro-DKP derivatives were selected for synthesis and evaluation as type II inhibitors. The general structures are shown in Fig 7.

### Synthesis of type II via the Ugi reaction

As the cyclisation protocol for the *N*-alkylated dipeptide methyl esters (Fig 4) only gave low yields and only provided disubstituted 2,5-DKPs, an alternative strategy for the synthesis of the 2,5-DKP-ring system was considered. Highly substituted 2,5-DKPs [37] can be synthesised *via* a Ugi reaction, followed by a deprotection and cyclisation step of the Ugi product [38].

**Fig 8:**
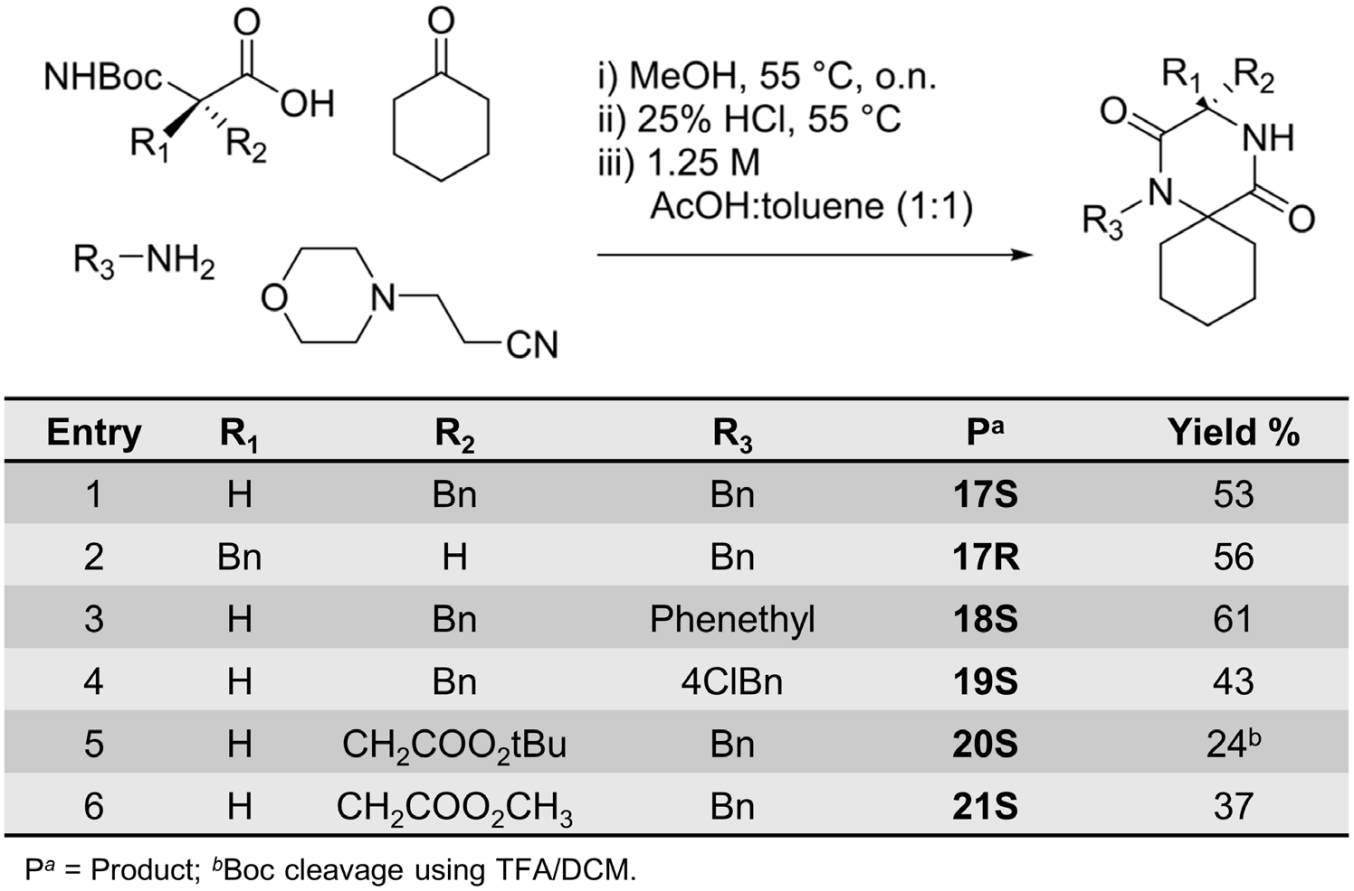
Synthesis of spiro-2-DKPs 17–21.

The Ugi reaction protocol was therefore used to synthesise a series of spiro-2-DKPs (Fig 8).

Compounds **17S** and **17R** (Fig 8, entries 1 and 2) were synthesised in order to confirm the preference of either the *S*- or *R*-configuration at C3 for binding to MDM2. Different R_3_-substituents (Fig 8, entries 1–4) were introduced in an attempt to find optimal substituents for interaction with the Trp-pocket. In general, these reactions proceeded in good yields, typically 50–60% over the three reaction steps. The lower yield for the 4-Cl-benzyl substituent was probably due to the lower reactivity of the electron-deficient 4-chlorobenzylamine.

Compound **20S** was synthesised using Boc-L-aspartic acid 4-*tert*-butyl ester as the starting material. To avoid hydrolysis of the *tert*-butyl ester during Boc-deprotection, TFA in dry DCM was used instead of HCl (aq). Nevertheless, the *tert*-butyl ester was cleaved under these conditions and the carboxylic acid obtained was lost during workup. Re-running the reaction in methanolic HCl resulted in transesterification of the *tert*-butyl ester to the methyl ester with a slight improvement in the isolated yield of 37% for **21S** from 24% for **20S** (Fig 8, entries 5–6).

**Fig 9:**
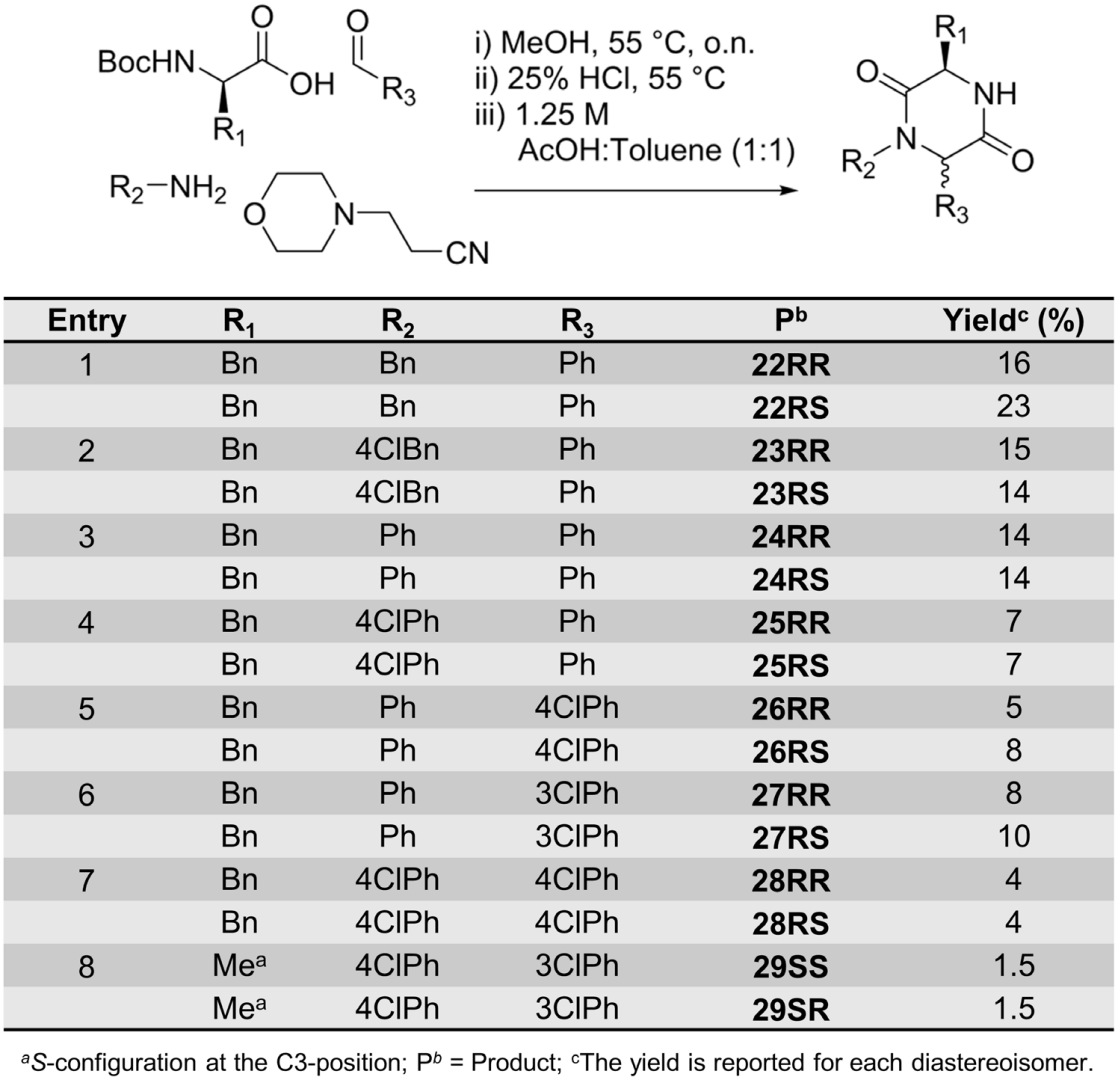
Synthesis of non-spiro-DKPs 22–29.

The same Ugi reaction protocol was used for the synthesis of non-spiro-DKPs (Fig 9). The yields were however generally lower compared with those for the spiro-2-DKPs (Fig 8). When an aldehyde was used instead of a cyclic ketone, the products were obtained as diastereomeric mixtures; the stereoisomers could be separated by silica column chromatography. Compounds **22RR** and **22RS** were isolated in a combined yield of 39%, while **23RR** and **23RS** were isolated in a combined yield of 29%.

The use of aniline instead of benzylamine as the amine component in the Ugi reaction resulted in lower yields (Fig 9, entries 3, 5–6). A further reduction in yield was also observed for the electron-deficient 4-choroaniline (14%) (Fig 9, entries 3–4). The combination of an aniline with a chlorobenzaldehyde, which are both electron-deficient, led to a further reduction in yield (Fig 9, entries 7–8). Attempts to improve the yields *via* pre-formation of the imine were not successful. In addition to the low yield, some of the products (**24**–**29**) proved to be very difficult to purify; the impurities could however be removed after alkylation at the *N*4-position.

**Fig 10:**
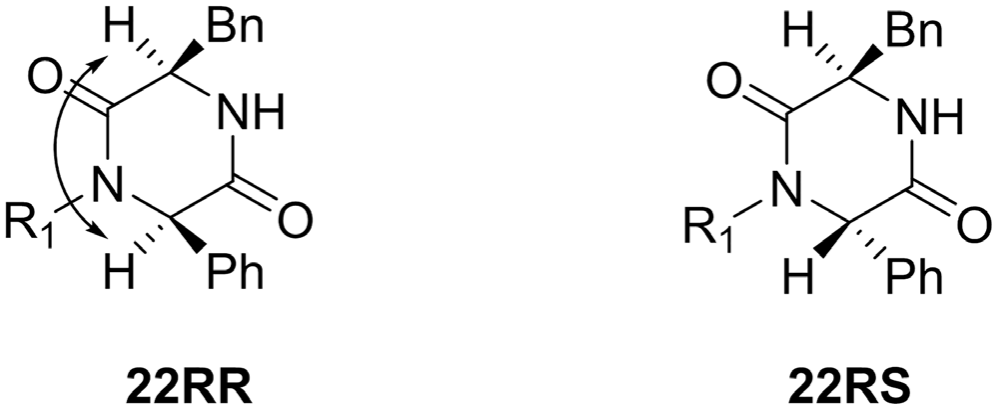
Stereochemical assignment of non-spiro-DKPs. NOE correlations are shown by a double headed arrow.

The absolute stereochemistry of compounds **22RR** and **22RS** were determined by NOE correlations. NOEs were observed for the diastereoisomer **22RR**, with the C3 and C6 substituents being on the same side of the 2,5-DKPs ring (Fig 10). For the other diastereoisomer, **22RS** with the C3 and C6 substituents on opposite sides of the 2,5-DKPs ring, no NOE was observed (see S1 Information for details).

**Fig 11:**
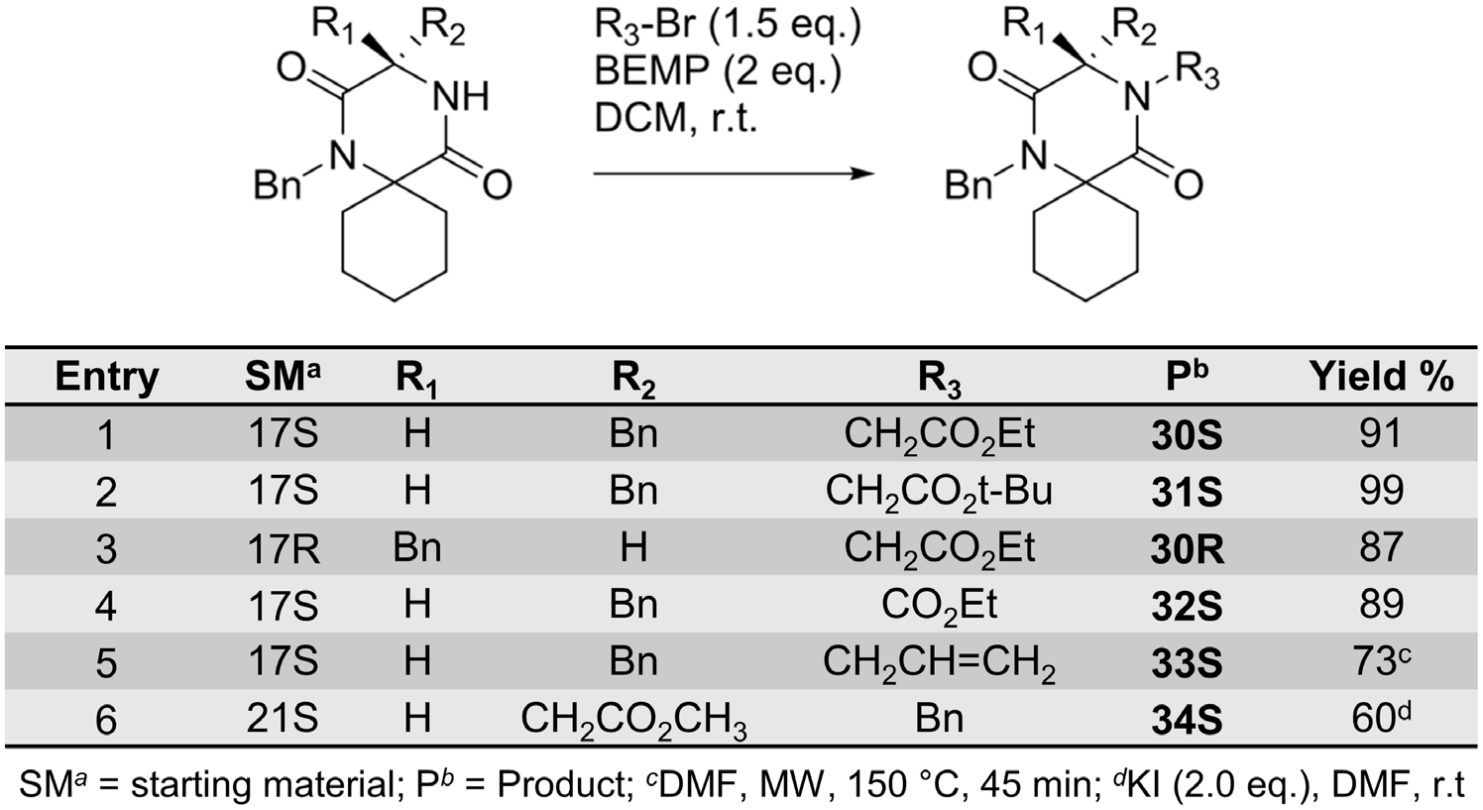
*N*4-Alkylation of spiro-2-DKPs 30–34.

For the introduction of the fourth substituent in the *N*4-posititon of the spiro-2-DKPs (Fig 11), a previously reported alkylation protocol was used utilising the strong base 2-*tert*-butylimino-2-diethylamino-1,3-dimethylperhydro-1,3,2-diazaphosphorine (BEMP) [25]. Ethoxycarbonylmethyl, *tert*-butoxycarbonylmethyl and ethoxycarbonyl moieties (Fig 11, entries 1–4) were introduced in excellent yields when running the reactions at room temperature. Allylation of **17S** at *N*4 was slow at room temperature and required microwave heating to afford a good yield (Fig 11, entry 5). Introduction of the benzyl group in position *N*4 was accomplished by activation of benzyl bromide using potassium iodide and DMF as solvent at room temperature (Fig 11, entry 6).

**Fig 12:**
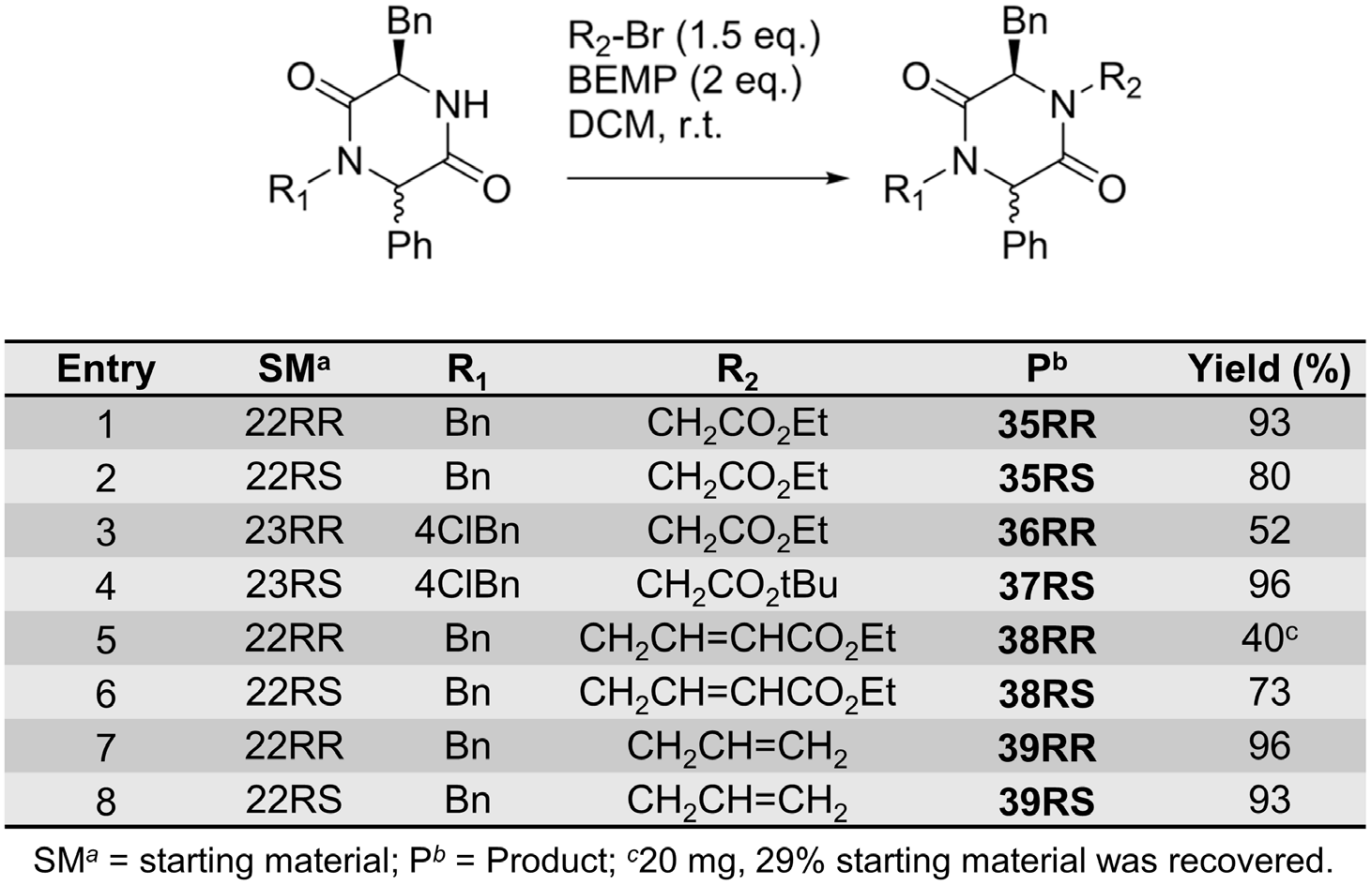
*N*4-alkylation of non-spiro-DKPs 35–39.

The BEMP protocol was also very well suited for the alkylation of *N*4 of the non-spiro-DKPs (Fig 12). Generally, the reactions were complete within 12 hours. However, alkylation with ethyl 4-bromocrotonate (Fig 12, entries 5–6) required extended reaction times and these reactions did not go to completion even after 72 hours.

**Fig 13:**
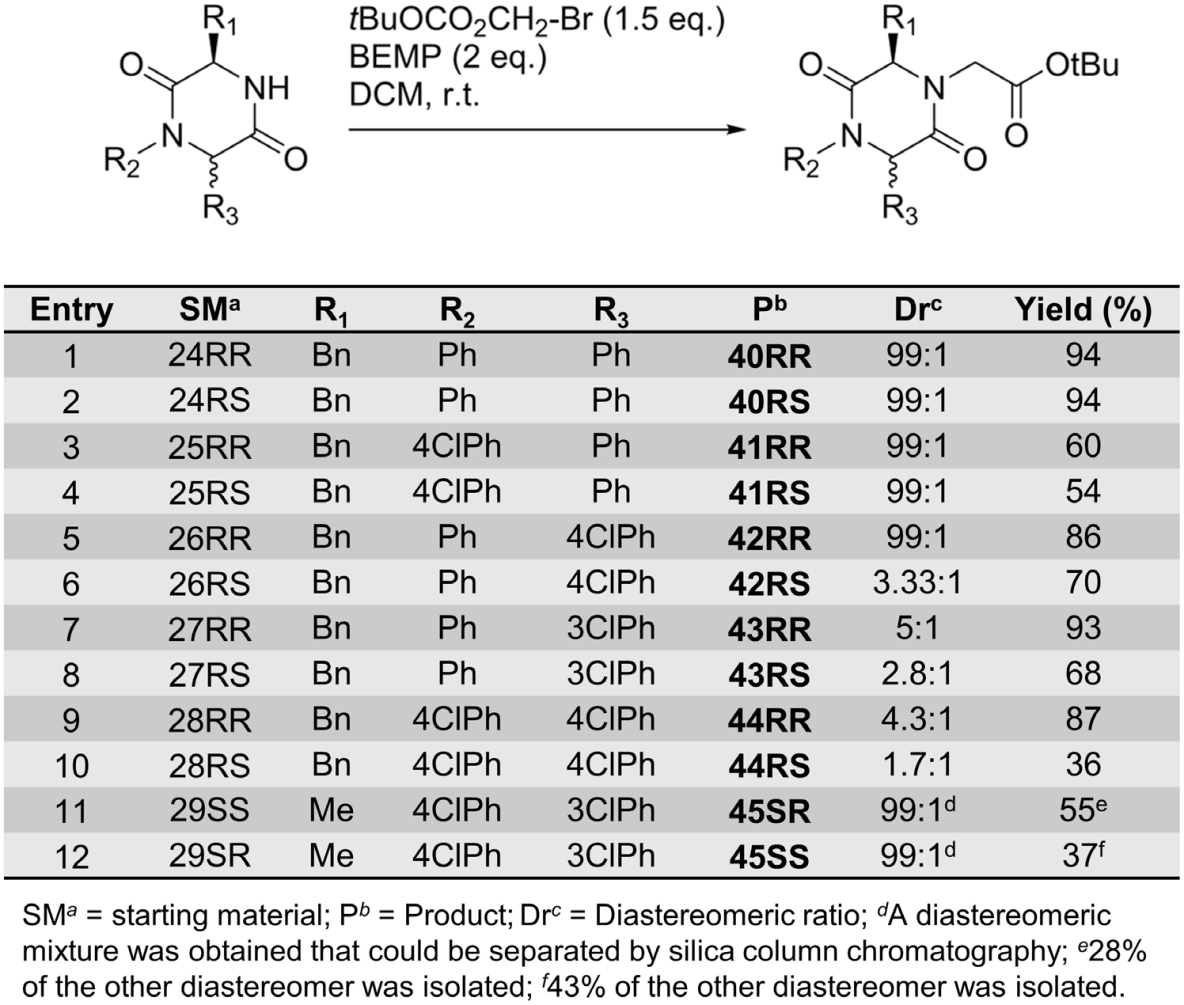
*N*4-Alkylation of non-spiro-DKPs afforded 40–45.

The BEMP alkylation protocol was also used to synthesise compounds **40–45** (Fig 13). The lower yields obtained for **41RR**, **41RS**, **42RS**, **43RS** and **44RS** are ascribed to impurities carried through from the previous synthetic steps. The introduction of electron-deficient substituents at the *N*1- and C6-positions resulted in epimerisation at the C6-position (Fig 13, entries 6–10). The diastereomeric ratio was determined from ^1^H NMR spectra. The diastereomeric mixture could be separated by silica column chromatography and **45SS** and **45SR** were isolated as single enantiomers (Fig 13, entries 11–12).

**Fig 14:**
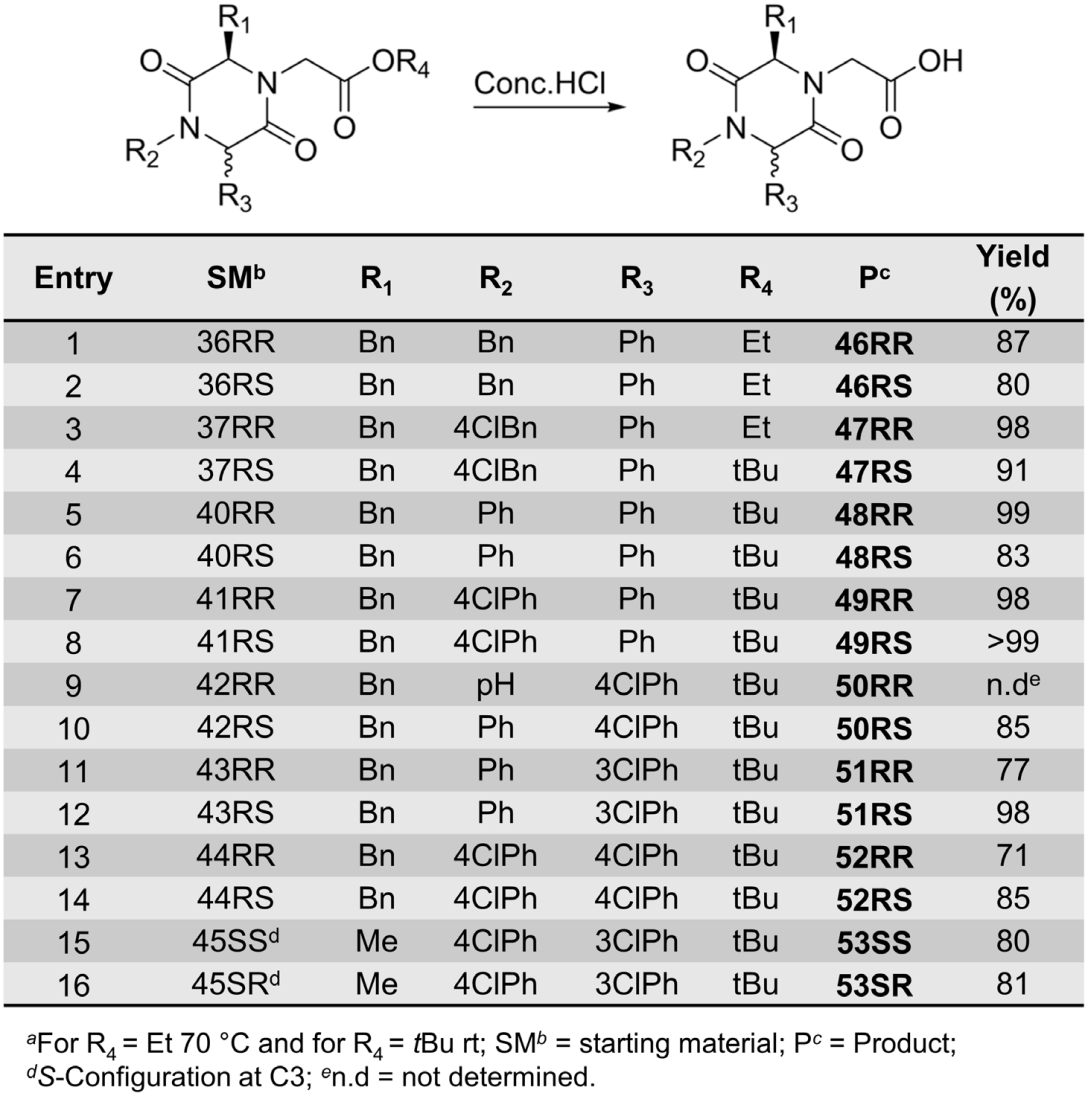
Ester hydrolysis of non-spiro-DKPs afforded acids 46-53.^*a*^

**Fig 15:**
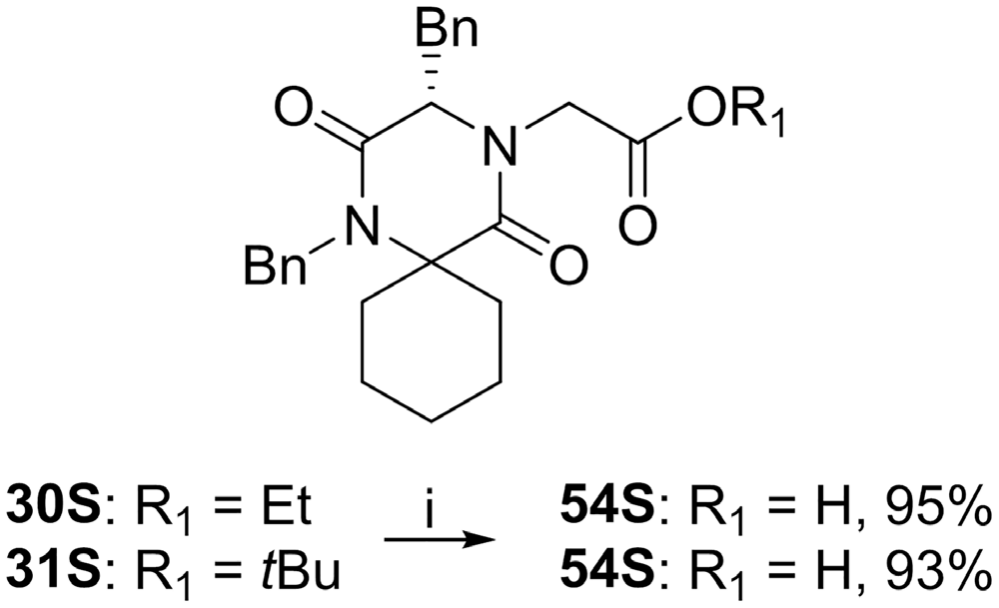
Ester hydrolysis of 30S and 31S. Reagents and reaction conditions: i) Conc. HCl (aq.) for R_1_ = Et, 70°C o.n. For R_1_ = *t*Bu, r.t. o.n.

A selection of 2,5-DKP esters were then further reacted to introduce other functionalities at the *N*4-positon. The esters were first hydrolysed to the corresponding carboxylic acids (Figs 14 and 15). The hydrolysis was initially tested on **36RR** using LiOH in THF/water (1:1). However, this reaction resulted in epimerisation at the C6-position. Acid-catalysed hydrolysis of the ethyl ester using HCl at room temperature gave the desired product without epimerisation, but the reaction was rather slow and full conversion was not achieved. Instead, the reaction was performed in aqueous concentrated HCl at 70°C. However, for the *tert*-butyl ester, the hydrolysis could be run at room temperature. All products were obtained in very good to excellent yields (Figs 14 and 15).

**Fig 16:**
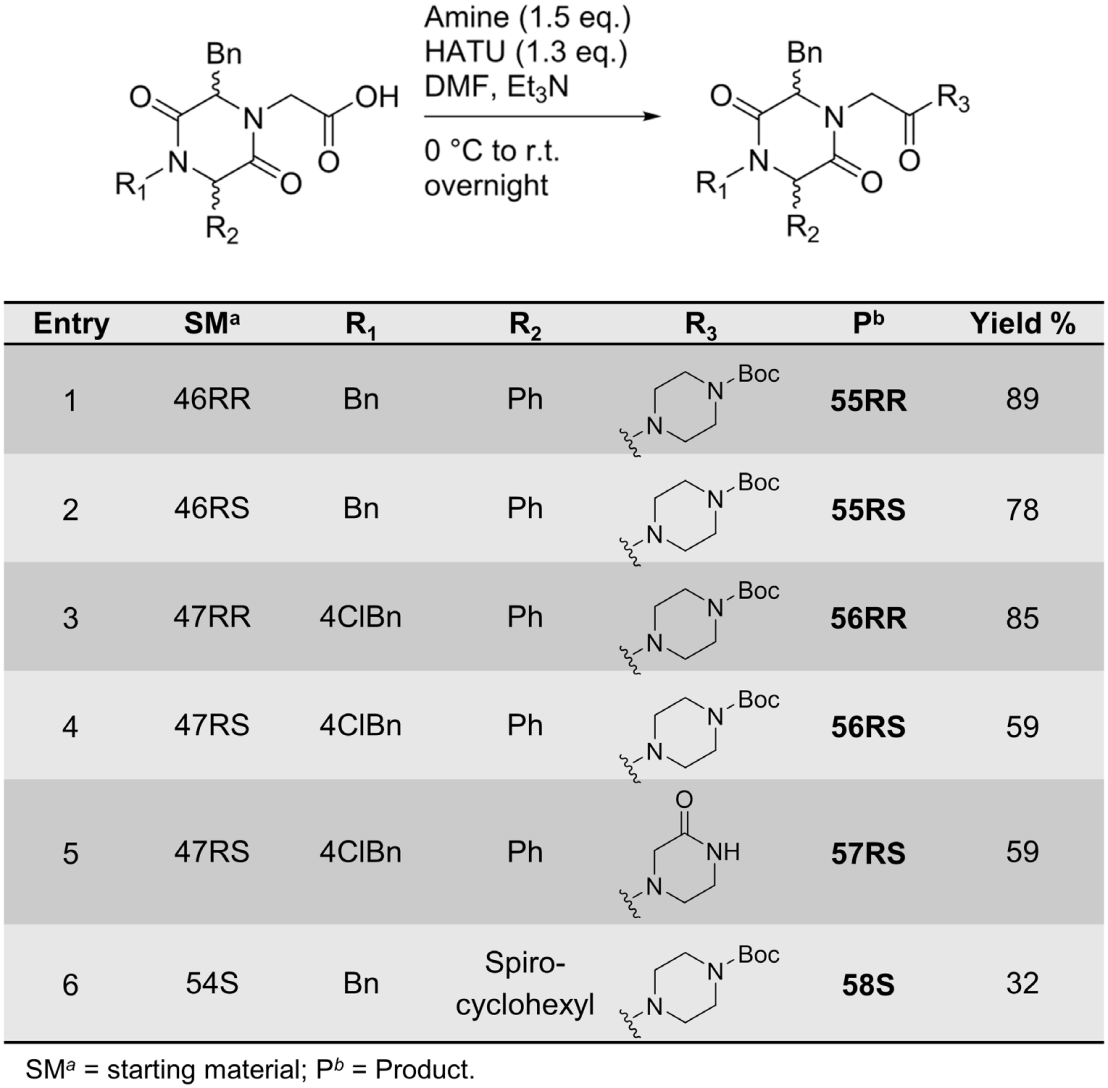
Amidation of 2,5-DKPs afforded 55–58.

The carboxylic acids **46RR**, **46RS**, **47RR**, **47RS** and **54S** were then further functionalised by amidation using 1-Boc-piperazine and 2-oxopiperazine (Fig 16). The coupling reactions were performed using HATU and Et_3_N in DCM affording the target compounds in good yields.

**Fig 17:**
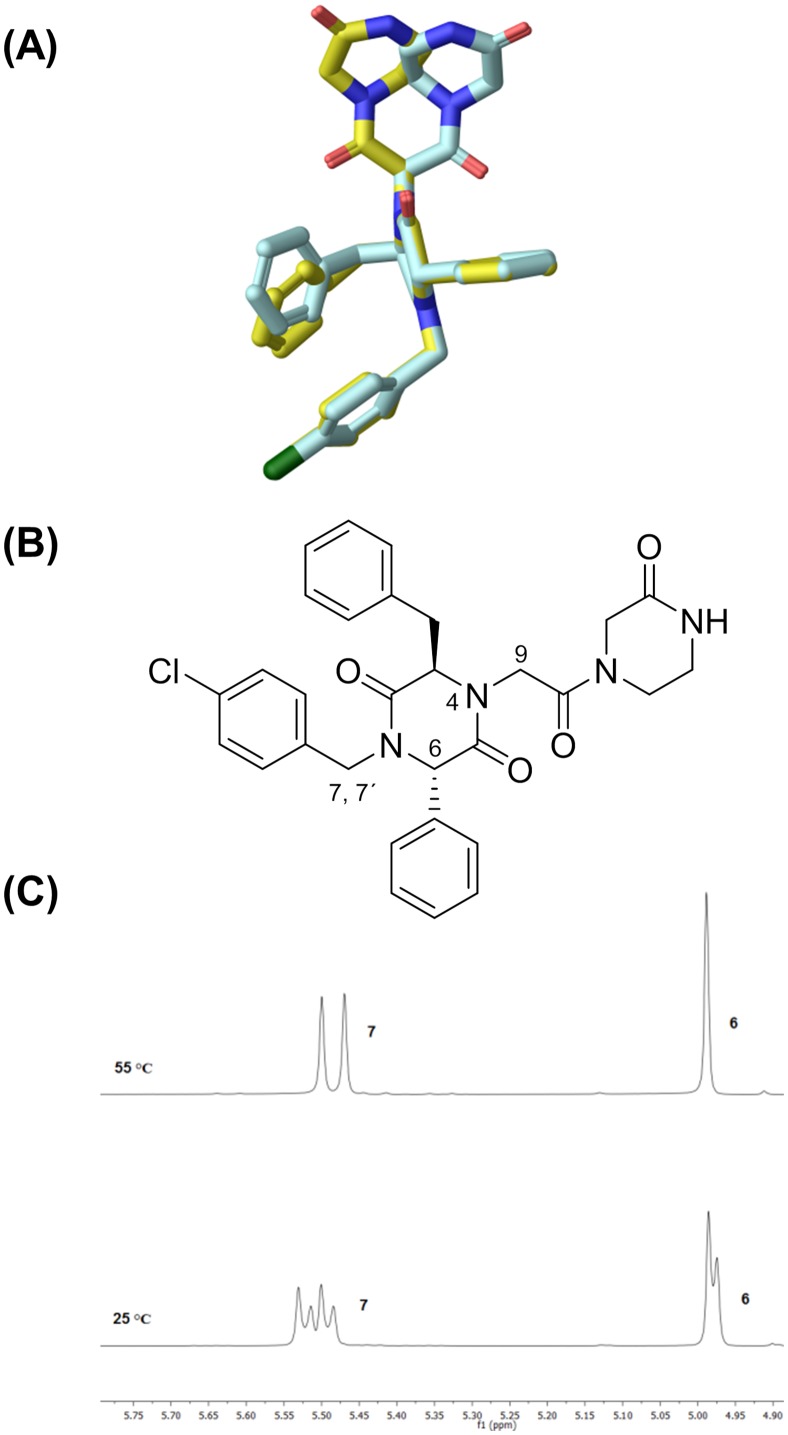
Conformational analysis of 57RS. (**A**) Model of two low energy conformations of **57RS**; (**B**) Chemical structure of **57RS** with atom numbers; (**C**) ^1^H NMR signals from H7 and H6 of **57RS** at 25°C and 55°C.

Interestingly, double peaks were observed for several signals in both ^1^H and ^13^C NMR spectra for **57RS**. Hindered rotation around the *N*4-C9 bond resulting in two different low energy conformations of **57RS** at room temperature in solution could be responsible for the double peaks observed. Conformational analysis of **57RS** indicated two major conformations, with the 2-oxopiperazine moiety pointing in opposite directions (Fig 17A).

NMR experiments were then conducted to demonstrate that **57RS** can adopt two low energy conformations. When two low energy conformers exist, the resulting double peaks will coalescence at elevated temperatures [39, 40] since heating will facilitate the rotation around the bonds. At 25°C and 55°C, a clear difference was observed in the ^1^H and ^13^C NMR spectra, confirming that **57RS** adopts two low energy conformations at 25°C (Fig 17) (See S1 Information for full ^1^H and ^13^C spectra).

**Fig 18:**
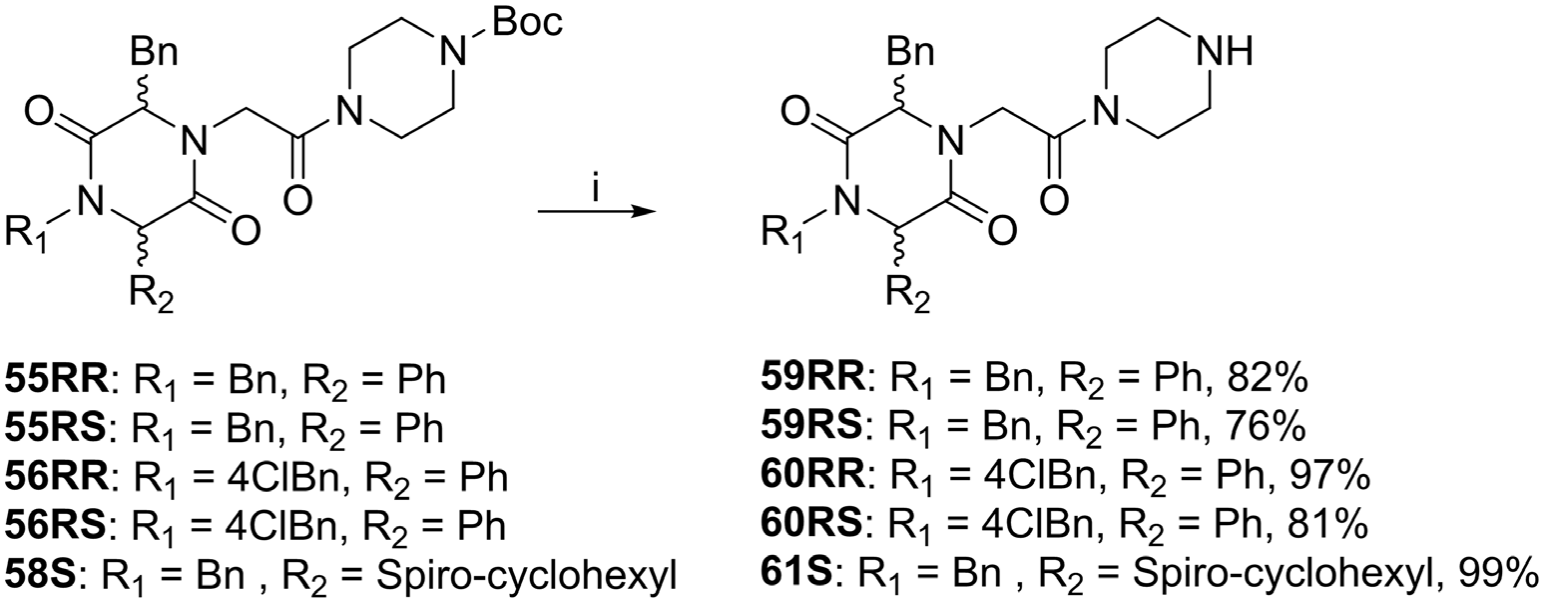
Boc-deprotection of 55–56 and 58S. Reagents and reaction conditions: i) TFA:DCM (1:1 v/v), 1h, r.t.

Treatment of **55RR**, **55RS**, **56RR**, **56RS** and **58S** with TFA:DCM (1:1) for one hour resulted in the complete removal of the Boc-group and afforded the target compounds in good to excellent yields (76–99%) (Fig 18).

**Fig 19:**
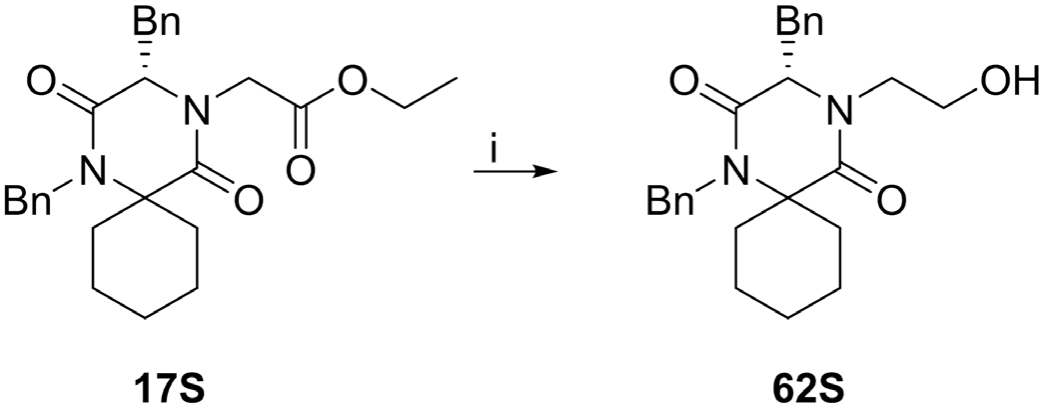
Reduction of 17S. Reagents and reaction conditions: i) NaBH_4_ (3.0 eq.), EtOH, r.t., 5 days.

Strategies for the introduction of an alcohol at *N*4 were also explored. First reduction of the ester functionality was investigated and treatment of **17S** with NaBH_4_ as a reducing agent afforded **62S** in 61% yield (Fig 19).

Reduction of **36RR** and **36RS** using NaBH_4_ in EtOH resulted in epimerisation of the product at the C6-position according to ^1^H NMR spectra. Attempts to reduce the carboxylic acid, **46RR**, using BH_3_•DMS failed and LCMS analysis of the reaction mixture revealed unidentified mass ions, epimerisation of the starting material was also observed in ^1^H NMR spectra.

### Biological evaluation of type II inhibitors

**Fig 20:**
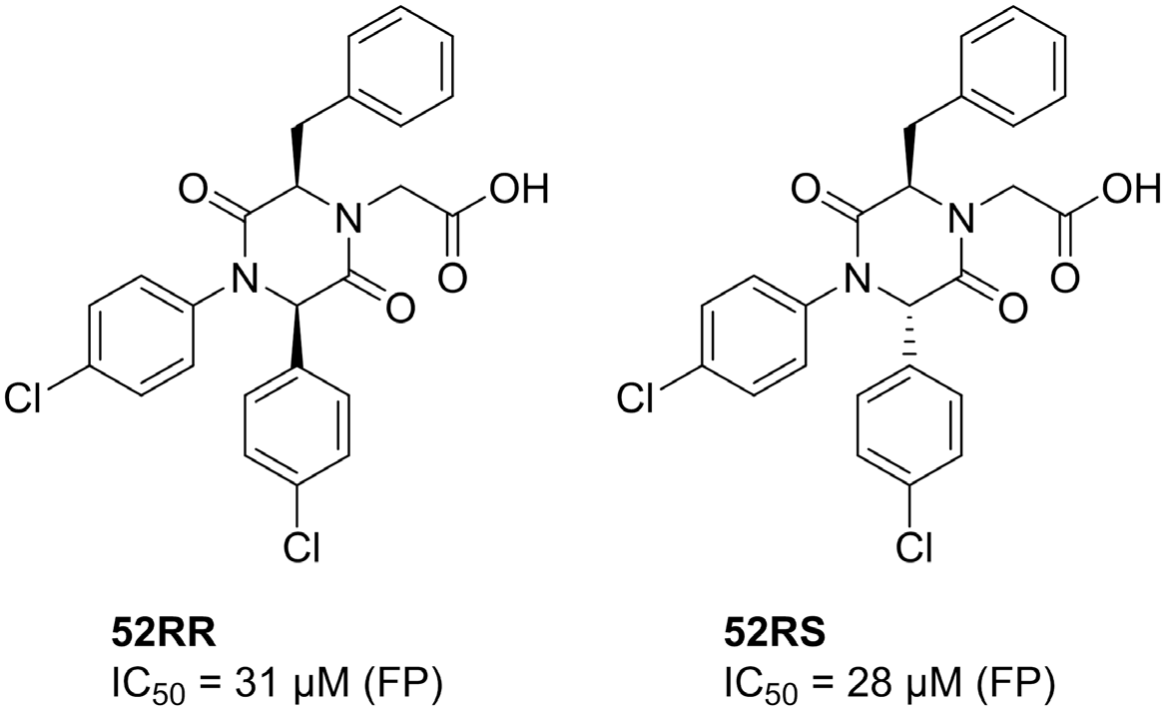
2,5-DKPs as inhibitors of the MDM2-p53 interaction.

To evaluate the 2,5-DKPs as potential MDM2-p53 inhibitors, the same FP-assay [34] was used as for the type III inhibitors. Out of 54 compounds evaluated, two compounds, **52RR** and **52RS**, were found to be active in the FP-assay, displaying IC_50_ values of 31 (95% CI [16.31, 60.49]) and 28 μM 95% CI [10.96, 71.43]), respectively (Fig 20) (see S1 Information for dose response curve of FP assay).

To confirm binding to the target, surface plasmon resonance (SPR) was used to study interactions of **52RR** and **52RS** with MDM2 [41, 42]. Plotting the steady state responses at different inhibitor concentrations yielded partial binding saturation curves from which K_D_ values of 155 (± 4) and 140 (± 3) for **52RR** and **52RS**, respectively, were calculated. These high values could be due to poor solubility of the inhibitors (see S1 Information for details).

The disparity between the competition potency seen in the FP assay and the binding affinity seen by SPR suggests that the apparent competitive inhibition in the FP assay may be due to compound or protein aggregation rather than direct competition. Likewise, the relatively poor affinity may be due to suboptimal solubility in the SPR buffer.

Therefore, to further validate the results from the FP-assay, WaterLOGSY [43] experiments were conducted that confirmed binding of **52RR** and **52RS** to MDM2 (see S1 Information for details). This suggests that the competitive potency measured in the FP experiment is accurate.

With only a few active compounds, it is very difficult to establish reasonable SARs. However, the results indicate the importance of two 4-chlorophenyl substituents in the *N*1- and C6-positions for binding to the target, since all other variants did not give any activity in the FP-assay. Analogous with the results obtained, chloro-substituted phenyls interacting with the Trp- and Phe-pockets of MDM2 have been shown by others to improve inhibitory potency. [15, 44] In addition, these results indicate that the presence of a carboxylic acid moiety at *N*4 is important for activity, since the ester analogues, **44RR** and **44RS**, were inactive in the FP-assay. Furthermore, the stereochemistry of the tested compounds does not seem to be of major importance as similar IC_50_ values were obtained for both compounds. It should be noted that the active compounds were tested as diastereomeric mixtures: 4.3:1 and 1.7:1 for **52RR** and **52RS**, respectively. In addition, the results follow the observation that many of the published inhibitors of the p53-MDM2 interaction are typically less extended than the model p53 α-helix, hence type II-like inhibitors.

## Conclusions

Two types of 2,5-DKPs inhibitors (type II and III) have been designed and synthesised as PPI inhibitors targeting the MDM2-p53 interaction. The first series of spiro-DKPs (type III inhibitors) were designed to directly mimic the topography of the α-helix in the MDM2-p53 binding interface. However, when these compounds were evaluated in an FP-assay for their inhibitory activity against the MDM2-p53 interaction, all spiro-DKPs were found to be inactive at the tested concentrations (up to 100 μM). Re-evaluation of the original design focusing on structure-based design, using the p53 binding pocket on MDM2 as a template, resulted in new series of type II inhibitors (spiro-2-DKPs and non-spiro-DKPs). An efficient strategy was developed for the preparation of tetrasubstituted spiro-2-DKPs and non-spiro-DKPs, using the Ugi protocol and alkylation reactions as the key steps in the synthesis. Two structurally-related compounds, **52RR** and **52RS**, showed inhibition at micromolar concentration (31 μM and 28 μM) in the FP-assay. Binding of **52RR** and **52RS** to MDM2 could be confirmed by SPR measurements and by WaterLOGSY experiments.

These results indicate that 2,5-DKPs could be used as a novel chemotype for the development of a new class of MDM2-p53 inhibitors. The current study also illustrates the challenge using structure-based design in the development of MDM2-p53 interaction inhibitors which has also recently been highlighted by others [45].

## Supporting Information

S1 Information Describes synthesis and characterisation including Proton (^1^H) and Carbon (^13^C) NMR spectra of all compounds synthesised . In addition, experimental procedures for the biological evaluation and conformational analysis procedure are included. A specific table of contents can be located in this document.
(DOCX)

## References

[1] Wade M, Li CY, Wahl GM. MDM2, MDMX and p53 in oncogenesis and cancer therapy. Nat. Rev. Cancer 2013; 13: 83–96. 10.1038/nrc3430 23303139 PMC4161369

[2] Vousden KH, Lu X. Live or let die: the cell's response to p53. Nat. Rev. Cancer 2002; 2: 594–604. 12154352 10.1038/nrc864

[3] Stiewe T. The p53 family in differentiation and tumorigenesis. Nat. Rev. Cancer 2007; 7: 165–168. 17332760 10.1038/nrc2072

[4] Toledo F, Wahl GM. Regulating the p53 pathway: in vitro hypotheses, in vivo veritas Nat. Rev. Cancer 2006; 6: 909–923. 17128209 10.1038/nrc2012

[5] Brown JC, Lain S, Verma CS, Fersht AR, Lane DP. Awakening guardian angels: drugging the p53 pathway Nat. Rev. Cancer 2009; 9: 862–873. 10.1038/nrc2763 19935675

[6] Feki A, Irminger-Finger I. Mutational spectrum of p53 mutations in primary breast and ovarian tumors. Crit. Rev. Oncol. Hematol. 2004; 52: 103–116. 15501075 10.1016/j.critrevonc.2004.07.002

[7] 7. Nigro JM, Baker SJ, Preisinger AC, Jessup JM, Hosteller R, Cleary K, et al Mutations in the p53 gene occur in diverse human tumour types. Nature 1989; 342: 705–708. 2531845 10.1038/342705a0

[8] Momand J, Jung D, Wilczynski S, Niland J. The MDM2 gene amplification database. Nucleic Acids Res. 1998; 26: 3453–3459. 9671804 10.1093/nar/26.15.3453 PMC147746

[9] Freedman DA, Wu L, Levine AJ. Functions of the MDM2 oncoprotein. Cell. Mol. Life Sci. 1999; 55: 96–107. 10065155 10.1007/s000180050273 PMC11146946

[10] Shangary S, Wang S. Targeting the MDM2-p53 Interaction for Cancer Therapy. Clin. Cancer. Res. 2008; 14: 5318–5324. 18765522 10.1158/1078-0432.CCR-07-5136 PMC2676446

[11] Kussie PH, Gorina S, Marechal V, Elenbaas B, Moreau J, Levine AJ, et al Structure of the MDM2 oncoprotein bound to the p53 tumor suppressor transactivation domain. Science 1996; 274: 948–953. 8875929 10.1126/science.274.5289.948

[12] Aeluri M, Chamakuri S, Dasari B, Guduru SKR, Jimmidi R, Jogula S, et al Small molecule modulators of protein-protein interactions: selected case studies. Chem. Rev. 2014; 114: 4640–4694. 10.1021/cr4004049 24673632

[13] Azzarito V, Long K, Murphy NS, Wilson AJ. Inhibition of α-helix-mediated protein-protein interactions using designed molecules. Nat. Chem. 2013; 5: 161–173. 10.1038/nchem.1568 23422557

[14] Vassilev LT, Vu BT, Graves B, Carvajal D, Podlaski F, Filipovic Z, et al In vivo activation of the p53 pathway by small-molecule antagonists of MDM2. Science 2004; 303: 844–848. 14704432 10.1126/science.1092472

[15] Sun D, Li Z, Rew Y, Gribble M, Bartberger MD, Beck HP, et al Discovery of AMG 232, a potent, selective, and orally bioavailable MDM2-p53 inhibitor in clinical development. J. Med. Chem. 2014; 57: 1454–1472. 10.1021/jm401753e 24456472

[16] Zhao Y, Yu S, Sun W, Liu L, Lu J, McEachern D, et al A potent small-molecule inhibitor of the MDM2-p53 interaction (MI-888) achieved complete and durable tumor regression in mice. J. Med. Chem. 2013; 56: 5553–5561. 10.1021/jm4005708 23786219 PMC3880646

[17] Orner BP, Ernst JT, Hamilton AD. Toward proteomimetics: terphenyl derivatives as structural and functional mimics of extended regions of an alpha-helix. J. Am. Chem. Soc. 2001; 123: 5382–5383. 11457415 10.1021/ja0025548

[18] Yin H, Lee GI, Park HS, Payne GA, Rodriguez JM, Sebti SM, et al Terphenyl-based helical mimetics that disrupt the p53/HDM2 interaction. Angew. Chem. Int. Ed. Engl. 2005; 44: 2704–2707. 15765497 10.1002/anie.200462316

[19] Biros SM, Moisan L, Mann E, Carella A, Zhai D, Reed JC, et al Heterocyclic alpha-helix mimetics for targeting protein-protein interactions. Bioorg. Med. Chem. Lett. 2007; 17: 4641–4645. 17555961 10.1016/j.bmcl.2007.05.075 PMC2699934

[20] Plante JP, Burnley T, Malkova B, Webb ME, Warriner SL, Edwards TA, et al Oligobenzamide proteomimetic inhibitors of the p53–*h*DM2 protein—protein interaction. Chem. Commun. 2009; 5091–5093. 10.1039/b908207g PMC2898631 20448956

[21] Lee JH, Zhang Q, Jo S, Chai SC, Oh M, Im W, et al Novel pyrrolopyrimidine-based α-helix mimetics: cell-permeable inhibitors of protein-protein interactions. J. Am. Chem. Soc. 2011; 133: 676–679. 10.1021/ja108230s 21171592 PMC3079198

[22] Aeluri M, Chamakuri S, Dasari B, Guduru SKR, Jimmidi R, Jogula S, et al Small Molecule Modulators of Protein—Protein Interactions: Selected Case Studies. Chem. Rev. 2014; 114: 4640–4694. 10.1021/cr4004049 24673632

[23] Milroy LG, Grossmann TN, Hennig S, Brunsveld L, Ottmann C. Modulators of Protein—Protein Interactions. Chem. Rev. 2014; 114: 4695–4748. 10.1021/cr400698c 24735440

[24] Rognan D. Rational design of protein-protein interaction inhibitors. Med. Chem. Commun. 2015; 6: 51–60.

[25] Tullberg M, Grøtli M, Luthman K. Synthesis of functionalized, unsymmetrical 1,3,4,6-tetrasubstituted 2,5-diketopiperazines. J. Org. Chem. 2007; 72: 195–199. 17194099 10.1021/jo0619635

[26] Tullberg M, Luthman K, Grøtli M. Microwave-assisted solid-phase synthesis of 2,5-diketopiperazines: solvent and resin dependence. J. Comb. Chem. 2006; 8: 915–922. 17096581 10.1021/cc0600876

[27] Tullberg M., Grøtli M., Luthman K.. Efficient synthesis of 2,5-diketopiperazines using microwave assisted heating. Tetrahedron 2006; 62: 7484–7491.

[28] Jam F, Tullberg M, Luthman K, Grøtli M. Microwave assisted synthesis of spiro-2,5- diketopiperazines. Tetrahedron 2007; 63: 9881–9889.

[29] Borthwick AD. 2,5-Diketopiperazines: Synthesis, Reactions, Medicinal Chemistry, and Bioactive Natural Products. Chem. Rev. 2012; 112: 3641–3716. 10.1021/cr200398y 22575049

[30] Park JD, Lee KJ, Kim DH. A new inhibitor design strategy for carboxypeptidase A as exemplified by N-(2-chloroethyl)-N-methylphenylalanine. Bioorg. Med.Chem. 2001; 9: 237–243. 11249116 10.1016/s0968-0896(00)00239-x

[31] Fieser LF, Fieser M, Reagents for Organic Synthesis; John Wiley & Sons, Inc.: New York, 1967; Vol. 1, pp 191.

[32] El-Faham A, Albericio F. Peptide Coupling Reagents, More than a Letter Soup. Chem. Rev. 2011; 111: 6557–6602. 10.1021/cr100048w 21866984

[33] Burkhardt ER, Coleridge BM. Reductive amination with 5-ethyl-2-methylpyridine borane. Tetrahedron Lett. 2008; 49: 5152–5155.

[34] Reed D, Shen Y, Shelat AA, Arnold LA, Ferreira AM, Zhu F, et al Identification and characterization of the first small molecule inhibitor of MDMX. J. Biol. Chem. 2010; 285: 10786–10796. 10.1074/jbc.M109.056747 20080970 PMC2856285

[35] Gonzalez-Lopez de Turiso F, Sun D, Rew Y, Bartberger MD, Beck HP, Canon J, et al Rational design and binding mode duality of MDM2-p53 inhibitors. J. Med. Chem. 2013; 56: 4053–4070. 10.1021/jm400293z 23597064

[36] Michelsen K, Jordan JB, Lewis J, Long AM, Yang E, Rew Y, et al Ordering of the N-terminus of human MDM2 by small molecule inhibitors. J. Am. Chem. Soc. 2012; 134: 17059–17067. 10.1021/ja305839b 22991965

[37] Rew Y, Sun D, Gonzalez-Lopez De Turiso F, Bartberger MD, Beck HP, Canon J, et al Structure-based design of novel inhibitors of the MDM2-p53 interaction. J. Med. Chem. 2012; 55: 4936–4954. 10.1021/jm300354j 22524527

[38] Nishizawa R, Nishiyama T, Hisaichi K, Minamoto C, Murota M, Takaoka Y, et al Discovery of 4-[4-({(3R)-1-butyl-3-[(R)-cyclohexyl(hydroxy)methyl]-2,5-dioxo-1,4,9-triazaspiro[5.5]undec-9-yl}methyl)phenoxy]benzoic acid hydrochloride: a highly potent orally available CCR5 selective antagonist. Bioorg. Med. Chem. 2011; 19: 4028–4042. 21658961 10.1016/j.bmc.2011.05.022 PMC7604827

[39] Mutulis F, Erdélyi M, Mutule I, Kreicberga J, Yahorava S, Yahorau A, et al 2-(p-Hydroxybenzyl)indoles—Side Products Formed Upon Cleavage of Indole Derivatives from Carboxylated Wang Polymer—an NMR Study. Molecules 2003; 8: 728–734.

[40] Gasparro FP, Kolodny NH. NMR determination of the rotational barrier in N,N-dimethylacetamide. A physical chemistry experiment. J. Chem. Educ.1977; 54: 258–261.

[41] Myszka DG. Improving biosensor analysis. J. Mol. Recogn. 1999; 12: 279–284. 10.1002/(SICI)1099-1352(199909/10)12:5<279::AID-JMR473>3.0.CO;2-3 10556875

[42] Papalia G, Myszka D. Exploring minimal biotinylation conditions for biosensor analysis using capture chips. Anal. Biochem. 2010; 403: 30–35. 10.1016/j.ab.2010.03.044 20371356

[43] Dalvit C, Fogliatto G, Stewart A, Veronesi M, Stockman B. WaterLOGSY as a method for primary NMR screening: practical aspects and range of applicability. *J*. *Biomol*. *NMR*, 2001; 21: 349–359. 11824754 10.1023/a:1013302231549

[44] Fry DC, Wartchow C, Graves B, Janson C, Lukacs C, Kammlott U, et al Deconstruction of a nutlin: dissecting the binding determinants of a potent protein-protein interaction inhibitor. ACS Med. Chem. Lett. 2013; 4: 660–665. 10.1021/ml400062c 24900726 PMC4027557

[45] Bharatham N, Bharatham K, Shelat AA, Bashford D. Ligand Binding Mode Prediction by Docking: Mdm2/Mdmx Inhibitors as a Case Study. J. Chem. Inf. Model. 2013; 54: 648–659. 10.1021/ci4004656 PMC4753531 24358984

