# Supplementary material for: Design, Synthesis and Evaluation of 2,5-Diketopiperazines as Inhibitors of the MDM2-p53 Interaction
Source: PLoS One. 2015 Oct 1;10(10):e0137867. doi: 10.1371/journal.pone.0137867 (PMC4591261; doi:10.1371/journal.pone.0137867)
Supplement: S1 Information — . In addition, experimental procedures for the biological evaluation and conformational analysis procedure are included. A specific table of contents can be located in this document. (DOCX) [file pone.0137867.s001.docx]

Supplementary Information

Design, Synthesis and Evaluation of 2,5-Diketo-piperazines as Inhibitors of the MDM2/p53 Interaction

Mariell Pettersson,^a^ Maria Quant,^a^ Jaeki Min,^b^ Luigi Iconaru,^c^ Richard W. Kriwacki,^c^ M. Brett Waddell,^d^ R. Kiplin Guy,^b^ Kristina Luthman,^a^ Morten Grøtli^a^

^a^Department of Chemistry and Molecular Biology, University of Gothenburg, 412 96 Gothenburg, Sweden. E-mail: grotli@chem.gu.se.

^b^Department of Chemical Biology and Therapeutics, St. Jude Children’s Research Hospital, 262 Danny Thomas Place, Memphis, Tennessee 38105, United States

^c^Department of Structural Biology, St. Jude Children’s Research Hospital, 262 Danny Thomas Place, Memphis, Tennessee 38105, United States

^d^Molecular Interaction Analysis Shared Resource, St. Jude Children’s Research Hospital, 262 Danny Thomas Place, Memphis, Tennessee 38105, United States

^*^Corresponding author (grotli@chem.gu.se)

Experimental details, ^1^H and ^13^C NMR spectra, conformational analysis, fluorescence polarization assay procedure and WaterLOGSY procedure.

Table of Content

[Experimental 3](#_Toc425242512)

[Fluorescence Polarization (FP) Assay 33](#_Toc425242513)

[WaterLOGSY measurements 34](#_Toc425242514)

[Surface plasmon resonance (SPR) measurements 35](#_Toc425242515)

[Molecular modelling 37](#_Toc425242516)

[^1^H and ^13^C NMR Spectra 37](#_Toc425242517)

[Reference 129](#_Toc425242518)

# Experimental

**General considerations**

All commercial chemicals were used without prior purification. When dry solvent were needed DCM was distilled from calcium hydride and THF was distilled from sodium and benzophenone. Microwave reactions were performed using a Biotage Initiator reactor with fixed hold time. All reaction were monitored by TLC (Merck silica gel 60 F_254_) and analysed under UV (254 nm) or LCMS analysis. LCMS analysis was performed on a API SCIEX 150 EX Perkin Elmer ESI-MS (30 eV) connected to a Perkin Elmer gradient pump system and a C8 column (Gemini) using acetonitrile and MilliQ-water with 1% formic acid as mobile phases with a gradient of 5 to 95% acetonitrile over 4 min. Column chromatography was performed by flash chromatography (wet-packed silica, 0.04–0.063 mm) or by automated column chromatography on a Biotage SP-4 instrument using pre-packed silica columns. Analytical high-performance liquid chromatography (HPLC) analysis was carried out on a Waters separation module 2690 connected to a Waters photodiode array detector 996 using an Atlantis^®^ 5 μm C18 AQ (250×4.6 mm) column. Preparative HPLC was carried out on a Waters 600 controller connected to a Waters 2487 Dual λ Absorbance detector using an Atlantis^®^ Prep T3 5 μm C-18 (250×19 mm) column. HRMS analysis was performed on a Waters LCTp XE mass spectrometer with an Acquity UPLC BEH C18 (pH 10) or an Acquity UPLC CSH C18 (pH 3) column eluting with a gradient of 5-95% acetonitrile in MQ-water. Optical rotation was measured on a Perkin-Elmer 341LC Polarimeter. ^1^H and ^13^C NMR spectra were obtained at 400 and 100 MHz respectively, using a Varian 400/54 spectrometer. The solvent peak was used as reference.

**General procedure A: Reductive amination**

Following a published procedure^1^ with some modifications, 8-amino-1,4-dioxaspiro[4.5]decane-8-carboxylic acid (1 eq.) was suspended in dry methanol (0.05 M) under N_2_(g). Et_3_N (1.2 eq.) and aldehyde (1.2-1.5 eq.) was added. The mixture was stirred for 10 min and then sodium cyanoborohydride (1.0 eq.) was added. The reaction was left at r.t. overnight. The solvent, Et_3_N and excess aldehyde was removed under reduced pressure. The obtained oil/residue was suspended in 0.5 M HCl in methanol (0.2 M) and stirred at 50 °C for 2.5 h. The reaction mixture was diluted with methanol, if observed, insoluble material was filtered off. The solvent was removed under reduced pressure and co-evaporated with toluene. The product was isolated as the hydrochloride salt observed as white solids. The product was identified by LCMS analysis and was used in the next step without further characterization or purification.

**General procedure B: Synthesis of dipeptide**

The carboxylic acid (1.0 eq.) was dissolved in dry DMF (0.03 M). HATU (2.0 eq.) and amine (1.7-2.0 eq.) was added. The vial was capped and flushed with N_2_(g), DIPEA (6-12 eq.) was added in one portion and the reaction mixture was directly heated in the micro wave reactor at 60 °C for 30 min. The solvent was evaporated, DCM and distilled H_2_O (2:1) was added to the residue. The phases were separated and the organic phase was washed with 0.5 M HCl (aq.), 15% NaOH (aq.) and brine. The organic phase was dried over MgSO_4_, filtered and the solvent was removed under reduced pressure. The crude product was purified by automated flash column chromatography. Although, some impurities could be detected in ^1^H NMR spectra the product was used in the next step without further purification.

**General procedure C: Synthesis of 2,5-DKPs via the Ugi reaction.**

Following a published procedure^7^ with some modifications, the amino acid was dissolved in dry methanol (0.26-0.30 M), the flask was capped and flushed with N_2_. The amine (1 eq.), ketone (1 eq.) and isocyanide (1 eq.) were added in that order at room temperature in an oven dried round bottom flask under N_2_(g). The reaction was heated to 55 °C until full consumption of the starting material was observed by LCMS analysis, typically overnight. Conc. HCl was added (25 v% of the solvent) and the reaction was heated to 55 °C. LCMS analysis showed full conversion to the corresponding unprotected Ugi-product after typical reaction time of 1-3 h. The solvent was removed under reduced pressure. The obtained white solids or yellow oil was dissolved in distilled water. Sat. NaHCO_3_ (aq.) was added whereupon a white precipitate was observed. The water phase was extracted with ethyl acetate (x3). The combined organic phases were dried over Na_2_SO_4_, filtered and the solvent was removed under reduced pressure upon which a light yellow oil was obtained. Toluene and 1.25 M acetic acid (1:1 ratio) was added to the oil (0.26 M) and the mixture was heated to 80 °C until no starting material could be observed by LCMS analysis (2-4 h). The reaction was diluted with ethyl acetate (6 times the amount of solvent volume), the organic phase was washed with a small amount of water, sat. NaHCO_3_ (aq.) and brine, dried over Na_2_SO_4_, filtered and the solvent was removed under reduced pressure. The crude product was purified using automated flash column chromatography using pentane and ethyl acetate mixtures or DCM and methanol mixtures. When anilines were used as the amino component, purification of the product failed and the product was used in the next step without further purification.

**General procedure D: Amide alkylation**

The 1,3,6-trisubstituted-2,5-diketopiperazine (1 eq.) was dissolved in dry DCM (0.04 M) in an oven dried round-bottomed flask or microwave vial. The flask was capped and flushed with N_2_ (g). BEMP (2 eq.) was added followed by the electrophile (1.5-2.1 eq.) and the reaction was stirred at r.t. overnight or until LCMS analysis showed full consumption of the starting material. The reaction mixture was extracted with 0.5 M NaOH (aq.) and 0.5 M HCl (aq.). The combined organic phases were washed with brine, dried over Na_2_SO_4_ and the solvent was removed under reduced pressure. The crude product was purified using automated flash column chromatography using pentane and ethyl acetate mixtures.

**General procedure E: Ester hydrolysis**

The 1,3,4,6-tetrasubstituted-2,5-diketopiperazine and conc. HCl was stirred at 70 °C or r.t. until LCMS analysis showed full consumption of the starting material. The reaction mixture was diluted with distilled water to around 3M and extracted with ethyl acetate (x4). The organic phase was washed with brine, dried over Na_2_SO_4_ and the solvent was removed under reduced pressure. The crude was co-evaporated with chloroform (x3) and then dried under high vacuum.

**General procedure F: Peptide coupling**

The carboxylic acid (1 eq.) and amine (1.5 eq.) were dissolved in dry DMF (0.06-0.1M) in an oven dried microwave vial. HATU (1.3 eq.) was added and the vial was capped and flushed with N_2_. The solution was cooled to 0 °C and Et_3_N (3 eq.) was added. The reaction was stirred at 0 °C for 15 min then allowed to reach r.t. overnight. The reaction mixture was diluted with ethyl acetate (same amount as solvent volume), washed with 0.25 M HCl (aq.), sat. NaHCO_3_ (aq.) and brine. The organic phase was dried over Na_2_SO_4_ and the solvent was removed under reduced pressure. The crude product was purified using automated flash column chromatography using pentane and ethyl acetate mixtures.

**General procedure G: Boc-deprotection**

The 1,3,4,6-tetrasubstituted-2,5-diketopiperazine (1 eq.) was dissolved in DCM (0.1 M) in a vial. TFA (0.1 M) was added and the reaction was stirred at r.t. for 1 h. The solvent was removed by bubbling N_2_ through the vial then under reduced pressure. The crude was dissolved in DCM (10 ml), washed with sat. NaHCO_3_, (aq.) distilled water and brine. The organic phase was dried over Na_2_SO_4_ and the solvent was removed under reduced pressure then dried under high vacuum.

**8-Benzylamino-1,4-dioxaspiro[4.5]decane-8-carboxylic acid**

Following general procedure A for the reductive amination from 8-amino-1,4-dioxaspiro[4.5]decane-8-carboxylic acid (500 mg, 2.5 mmol), Et_3_N (0.42 ml, 3.0 mmol), benzaldehyde (0.30 ml, 3.0 mmol) and sodium cyanoborohydride (156 mg, 2.5 mmol) in dry methanol (50 ml). 8-N-benzyl-amino-1,4-dioxaspiro[4.5]decane-8-carboxylic acid was identified by LCMS analysis and was used in the next step without further characterization or purification. LRMS *m/z* [M + H]^+^ calculated for C_16_H_21_NO_4_: 292.2. Found: 292.2.

**Methyl-8-benzylamino-1,4-dioxaspiro[4.5]decane-8-carboxylate (2)**

8-Benzylamino-1,4-dioxaspiro[4.5]decane-8-carboxylic acid (815 mg. 2.5 mmol) was dissolved in methanol and toluene (45 ml 1:3), trimethylsilyldiazomethane (4.0 ml, 8.0 mmol) was added. After 1 h the reaction mixture was light yellow and clear. The excess of trimethylsilyldiazomethane was quenched by the addition of a few drops acetic acid, until a colourless and clear reaction mixture was observed. The solvent was removed under reduced pressure and the crude material was suspended in DCM and 1M NaOH was added. The water phase was extracted with DCM (x2) and the combined organic phases were dried over MgSO_4_, filtered and the solvent was removed under reduced pressure. The crude product was purified by automated flash column chromatography (10% ethyl acetate in pentane) providing **2** as white solids (414 mg, 55%). ^1^H NMR (CDCl_3_): δ 7.38 – 7.26 (m, 4H), 7.28 – 7.19 (m, 1H), 3.98 – 3.92 (m, 4H), 3.73 (s, 3H), 3.58 (s, 2H), 2.13 – 1.92 (m, 4H), 1.89 – 1.76 (m, 2H), 1.63 – 1.53 (m, 2H); ^13^C NMR (CDCl_3_): δ 176.7, 140.6, 128.5, 128.4, 127.1, 108.6, 64.4, 64.3, 61.0, 51.9, 48.3, 30.6, 30.4.

**8-Benzylamino-1,4-dioxaspiro[4.5]decane-8-*N*-((*S*)-1-methoxycarbonyl-2-phenylethyl)carboxamido (4)**

Compound **4** was synthesised following general procedure B from 8-benzylamino-1,4-dioxaspiro[4.5]decane-8-carboxylic acid (200 mg, 0.61 mmol), HATU (464 mg, 1.22 mmol), L-phenylalanine methyl ester hydrochloride (263 mg, 1.22 mmol) and DIPEA (1.26 ml, 7.31 mmol.) in dry DMF (18 ml). The crude product was purified by automated flash column chromatography (40% ethyl acetate in heptane). Compound **4** was isolated as a yellow oil (159 mg, 58%) and used in the next step whiteout further purifications although some small impurities was identified in ^1^H NMR spectra. ^1^H NMR (CDCl_3_): δ 7.84 (d, *J* 8.5 Hz, 1H), 7.32 – 7.14 (m, 8H), 7.13 – 7.05 (m, 2H), 4.90 (ddd, *J* 8.5, 6.9, 5.5 Hz, 1H), 3.96 – 3.93 (m, 4H), 3.71 (s, 3H), 3.52 (d, *J* 11.9 Hz, 1H), 3.42 (d, *J* 11.9 Hz, 1H), 3.24 – 3.16 (m, 1H), 3.09 (dd, *J* 14.0, 6.9 Hz, 1H), 2.29 – 2.18 (m, 1H), 2.10 – 2.00 (m, 1H), 1.86 – 1.53 (m, 6H); ^13^C NMR (CDCl_3_): δ 175.8, 172.4, 136.2, 129.3, 128.7, 128.7, 128.4, 127.4, 127.2, 107.9, 64.5, 60.2, 52.8, 52.4, 47.6, 38.0, 31.3, 30.7, 30.6, 29.1.

**8-*iso*Butylamino-1,4-dioxaspiro[4.5]decane-8-carboxylic acid**

8-*iso*Butylamino-1,4-dioxaspiro[4.5]decane-8-carboxylic acid was synthesised following general procedure A from 8-Amino-1,4-dioxaspiro[4.5]decane-8-carboxylic acid (250 mg, 1.25 mmol), Et_3_N (0.21 ml, 1.49 mmol), isobutyraldehyde (0.17 ml, 1.87 mmol) was added and sodium cyanoborohydride (78 mg, 1.24 mmol) in dry methanol (25 ml). 8-N-isobutyl-amino-1,4-dioxaspiro[4.5]decane-8-carboxylic acid was identified by LCMS analysis and was used in the next step without further characterization or purification. LRMS *m/z* [M + H]^+^ calculated for C_13_H_23_NO_4_: 258.2. Found: 258.5.

**8-*iso*Butylamino-1,4-dioxaspiro[4.5]decane-8-*N*-((*S*)-1-methoxycarbonyl-2-phenylethyl)carboxamido (5)**

Compound **5** was synthesised following general procedure B from 8-*iso*Butylamino-1,4-dioxaspiro[4.5]decane-8-carboxylic acid (365 mg, 1.24 mmol), HATU (944 mg 2.48 mmol), L-phenylalanine methyl ester hydrochloride (536 mg, 2.48 mmol) and DIPEA (2.6 ml, 15.0 mmol) in dry DMF (18 ml). The crude product was purified by automated flash column chromatography (50% ethyl acetate in heptane) to provide **5** as as a yellow oil (354 mg, 68%) and used in the next step without further purifications although some small impurities was identified by NMR. ^1^H NMR (CDCl_3_): δ 7.75 (d, *J* 8.4 Hz, 1H), 7.33 – 7.18 (m, 3H), 7.16 – 7.07 (m, 2H), 4.83 (ddd, *J* 8.4, 7.1, 5.6 Hz, 1H), 3.93 (s, 4H), 3.71 (s, 3H), 3.18 (dd, *J* 14.0, 5.6 Hz, 1H), 3.05 (dd, *J* 14.0, 7.1 Hz, 1H), 2.19 – 1.95 (m, 4H), 1.78 – 1.63 (m, 3H), 1.57 – 1.46 (m, 4H), 0.82 (dd, *J* 6.7, 5.4 Hz, 6H); ^13^C NMR (CDCl_3_): δ 176.2, 172.3, 136.3, 129.3, 128.7, 127.1, 108.0, 64.4, 59.8, 52.9, 52.3, 50.8, 38.0, 30.8, 30.6, 30.5, 29.35, 29.25, 20.63, 20.56.

**8-{[(*tert*-Butoxy)carbonyl]amino}-1,4-dioxaspiro[4.5]decane-8-carboxylic acid**

8-Amino-1,4-dioxaspiro[4.5]decane-8-carboxylic acid (500 mg, 2.5 mmol, 1.0 eq.) was dissolved in 3M NaOH and 1,4-dioxane (1:2, 45 ml, pH ~12) and slightly cooled on an ice bath. Boc_2_O (2700 mg, 12.4 mmol, 5 eq.) was added and the reaction was stirred at 0 °C and slowly allowed to reach r.t. After 3.5 h withe slurry was obtained and the pH was adjusted to 12 with 3M NaOH (aq.) which dissolved the slurry. The mixture was stirred at r.t. overnight. Starting material could be observed by LCMS analysis. The pH was again readjusted to 12 with 3M NaOH (aq.) and additional Boc_2_O was added (540 mg, 1.0 eq.). The reaction was stirred for additional 12 h, whereupon LCMS analysis showed full consumption of starting material. The water phase was acidified to around pH 5 with 25% citric acid (aq,). The water phase was extracted with ethyl acetate (x5), after every extraction the pH was checked and adjusted to around 5. The combined organic phases were dried over Na_2_SO_4_, filtered and the solvent was removed under reduced pressure. ^1^H NMR on the crude product showed that the product contained acetic acid (probably from hydrolysis of ethyl acetate under the acidic conditions). The product was co-evaporated with heptane and ethyl acetate to remove the acetic acid. The product was isolated in quantitative yields as white solids. ^1^H NMR (CDCl_3_): δ 3.94 (s, 4H), 2.18 – 2.07 (m, 4H), 1.73 – 1.70 (m, 4H), 1.43 (s, 9H); ^13^C NMR (CDCl_3_): δ 178.9, 155.9, 107.7, 80.9, 64.5, 64.4, 58.1, 30.5, 30.3, 28.4.

**8-{[(*tert*-Butoxy)carbonyl]amino}-1,4-dioxaspiro[4.5]decane-8-*N*-((*S*)-1-methoxycarbonyl-2-phenylethyl)carboxamido (6)**

Compound **6** was synthesised following general procedure B from 8-{[(*tert*-butoxy)carbonyl]amino}-1,4-dioxaspiro[4.5]decane-8-carboxylic acid (100 mg, 0.33 mmol), HATU (252 mg, 0.66 mmol), L-phenylalanine methyl ester hydrochloride (143 mg, 0.66 mmol) and DIPEA (0.35 ml, 2.0 mmol) in dry DMF (11 ml). The crude product was purified by automated flash column chromatography (50% ethyl acetate in heptane) to provide **6** as white solids (130 mg, 85%). ^1^H NMR (CDCl_3_): δ 7.29 – 7.20 (m, 3H), 7.14 – 7.09 (m, 2H), 4.84 (dt, J 7.6, 5.9 Hz, 1H), 4.66 (s, 1H), 3.93 (s, 4H), 3.68 (s, 3H), 3.17 – 3.04 (m, 2H), 2.21 – 1.96 (m, 4H), 1.74 – 1.58 (m, 4H), 1.41 (s, 9H); ^13^C NMR (CDCl_3_): δ 173.7, 172.1, 154.9, 136.3, 129.4, 128.6, 127.1, 107.7, 80.5, 64.5, 64.4, 58.5, 53.5, 52.3, 38.2, 30.4, 30.4, 30.0, 28.4.

**(3*S*)-1,3-Dibenzyl-9,9-dihydroxy-1,4-diazaspiro[5.5]undecane-2,5-dione (7)**

Compound **4** (100 mg, 0.22 mmol) was mixed with distilled H_2_O (10 ml) in a microwave vial, the vial was sealed and heated in the microwave reactor for 90 min at 160 °C. The reaction mixture was transferred to a separation funnel and extracted with ethyl acetate (x5). The combined organic phases were washed with 1M NaOH (aq.) and brine, dried over MgSO_4_ and filtered. The solvent was removed under reduced pressure. The crude product was purified by automated flash column chromatography (50% ethyl acetate in heptane) to provide **7** as white solids (7 mg, 8%). ^1^H NMR (CDCl_3_): δ 7.49 – 7.44 (m, 2H), 7.37 – 7-33 (m, 2H), 7.30 – 7.24 (m, 4H), 7.20 – 7.17 (m, 2H), 4.67 (dd, *J* 5.8, 2.0 Hz, 1H), 3.96 (d, *J* 11.8 Hz, 1H), 3.87 (d, *J* 11.8 Hz, 1H), 3.70 (dd, *J* 13.8, 5.8 Hz, 1H), 3.31 (dd, *J* 13.8, 2.0 Hz, 1H), 2.15 (ddd, *J* 12.2, 10.8, 3.4 Hz, 1H), 1.98 – 1.61 (m, 5H), 1.56 – 1.46 (m, 1H), 0.07 – 0.00 (m, 1H); ^13^C NMR (CDCl_3_): δ 171.5, 171.3, 140.7, 135.4, 130.1, 128.7, 128.6, 128.5, 127.9, 127.3, 93.0, 59.0, 56.2, 48.3, 34.6, 33.0, 30.2, 29.6, 28.9; LRMS *m/z* [M + H]^+^ calculated for C_23_H_24_N_2_O_3_: 377.2. Found: 377.7.

**(3*S*)-3-Benzyl-1-*iso*butyl-9,9-dihydroxy-1,4-diazaspiro[5.5]undecane-2,5-dione (8)**

A solution of **5** (124 mg, 0.30 mmol) in acetone/1 M HCl (1:1 v/v, 40 ml) was heated at 55 °C for 2 days and then stirred at r.t. for additional two days. LCMS analysis showed product and in addition the starting material with both the ester and acetal hydrolysed. The acetone was removed under reduced pressure and the water was basified with 1M NaOH and extracted with ethyl acetate (x3). The organic phase was dried over MgS0_4_, filtered and the solvent was removed under reduced pressure. The crude product was purified by automated flash column chromatography (50% ethyl acetate in heptane) to provide **8** as a colourless oil (19 mg, 18%). ^1^H NMR (CDCl_3_): δ 7.33 – 7.22 (m, 3H), 7.21 – 7.11 (m, 2H), 4.64 (dd, *J* 5.7, 2.1 Hz, 1H), 3.67 (dd, *J* 13.8, 5.7 Hz, 1H), 3.28 (dd, *J* 13.8, 2.1 Hz, 1H), 2.57 – 2.42 (m, 2H), 2.18 – 2.09 (m, 1H), 1.94 – 1.83 (m, 2H), 1.78 – 1.58 (m, 5H), 1.50 – 1.38 (m, 1H), 0.97 (dd, *J* 6.6, 2.6 Hz, 6H), 0.08 – 0.00 (m, 1H); ^13^C NMR (CDCl_3_): δ 171.7, 171.3, 135.3, 130.1, 128.6, 127.8, 92.9, 58.5, 56.1, 51.3, 34.6, 33.0, 30.2, 29.5, 29.4, 28.7, 20.9, 20.8; HRMS *m/z* [M + H]^+^ calculated for C_20_H_28_N_3_O_4_: 361.2127. Found: 361.2115.

**(3*S*)-3-Benzyl-1,4-diazaspiro[5.5]undecane-2,5,9-trione (9)**

A mixture of **6** (108 mg, 0.23 mmol) and distilled H_2_O (9 ml) in a sealed microwave vial was heated in the microwave reactor at 160 °C for 30 min. The reaction mixture was transferred to a separation funnel and extracted with ethyl acetate (x5). The combined organic phases dried over MgSO_4_ and filtered. The solvent was removed under reduced pressure and **9** was obtained as colourless solid (27 mg, 40%). ^1^H NMR (DMF-*d*_7_): δ 8.40 (s, 1H), 8.18 (s, 1H), 7.36 – 7.20 (m, 5H), 4.42 (td, *J* 4.4, 2.1 Hz, 1H), 3.27 (dd, *J* 13.5, 4.1 Hz, 1H), 3.11 (dd, *J* 13.5, 4.8 Hz, 1H), 2.66 – 2.55 (m, 1H), 2.49 – 2.42 (m, 1H), 2.40 – 2.16 (m, 3H), 2.03 – 1.96 (m, 1H), 1.56 – 1.48 (m, 1H), 1.00 – 0.91 (m, 1H). ^13^C NMR (DMF-*d*_7_): δ 209.1, 170.0, 166.9, 136.8, 130.9, 128.5, 127.1, 56.4, 56.3, 39.2, 36.4, 36.3, 35.9, 35.7.

**4-*N*-[(1-Methoxycarbonyl-2-(*S*)-phenylethyl)carboxamido]-4-(*tert*-butoxycarbonylamino)-cyclohexanone (11)**

Compound **11** was synthesised following general procedure B from 1-{[(*tert*-Butoxy)carbonyl]amino}-4-oxocyclohexanecarboxylic acid (100 mg, 0.39 mmol), HATU (295 mg, 0.77 mmol), L-phenylalanine methyl ester hydrochloride (139 mg, 0.65 mmol) and DIPEA (0.40 ml, 2.33 mmol) in dry DMF (11 ml). The crude product was purified by automated flash column chromatography (50% ethyl acetate in heptane) to provide **11** as white solids (122 mg, 75%). ^1^H NMR (CDCl_3_): δ 7.29 – 7.19 (m, 3H), 7.13 – 7.09 (m, 2H), 4.85 (ddd, *J* 7.8, 6.8, 5.7 Hz, 1H), 3.69 (s, 3H), 3.15 (dd, *J* 13.9, 5.7 Hz, 1H), 3.05 (dd, *J* 13.9, 6.8 Hz, 1H), 2.53 – 2.14 (m, 8H), 1.41 (s, 9H); ^13^C NMR (CDCl_3_): δ 209.4, 172.9, 171.9, 154.9, 135.9, 129.2, 128.5, 127.1, 81.0, 58.0, 53.3, 52.3, 38.0, 36.6, 36.5, 32.5, 32.3, 28.2.

**4-*N*-[(1-Methoxycarbonyl-2-(*S*)-*iso*butylethyl)carboxamido]-4-(*tert*-butoxycarbonylamino)- cyclohexanone (12)**

Compound **12** was synthesised following general procedure B from 1-(*tert*-butoxycarbonylamino)-4-oxocyclohexanecarboxylic acid (250 mg, 0.97 mmol), HATU (739 mg, 1.94 mmol), L-leucine methyl ester hydrochloride (265 mg, 1.46 mmol) and DIPEA (1 ml, 5.83 mmol) in dry DMF (11 ml). The crude product was purified by automated flash column chromatography (40% ethyl acetate in heptane) to provide **12** as white solids (256 mg, 69%). ^1^H NMR (CDCl_3_): δ 4.87 (d, *J* 4.0 Hz, 1H), 4.59 (ddd, *J* 9.1, 7.9, 4.6 Hz, 1H), 3.72 (s, 3H), 2.60 – 2.34 (m, 6H), 2.34 – 2.22 (m, 2H), 1.71 – 1.53 (m, 3H), 1.46 (s, 9H), 0.94 (dd, *J* 6.1, 3.6 Hz, 6H); ^13^C NMR (CDCl_3_): δ 209.3, 173.5, 173.0, 155.1, 58.1, 52.4, 51.0, 41.5, 36.9, 36.7, 33.1, 32.3, 28.4, 24.9, 23.0, 21.9.

**(3*S*)-3-Benzyl-1,4-diazaspiro[5.5]undecane-2,5,9-trione (9)**

Distilled H_2_O (12 ml) was added to **11** (132 mg, 0.32 mmol) in a microwave vial and the reaction was heated in the microwave reactor at 160 °C for 70 min. The water was removed under reduced pressure and co-evaporated with ethyl acetate several times. **9** was isolated as white solids (89 mg, 99%). ^1^H and ^13^C NMR in accordance with **9** above.

**(3*S*)-3-*iso*Butyl-1,4-diazaspiro[5.5]undecane-2,5,9-trione (13)**

A mixture of **12** (215 mg, 0.56 mmol) and distilled H_2_O (20 ml) in a sealed microwave vial was heated in the microwave reactor at 160 °C for 70 min. LCMS analysis showed full consumption of the starting material. The mixture was extracted with ethyl acetate (x4), the phases were separate, combined organic phase were dried over MgSO_4_, filtered and the solvent was removed under reduced pressure. Compound **13** was isolated as white solids (112 mg, 79%). ^1^H NMR (DMF-*d*_7_). 8.44 (s, 1H), 8.17 (s, 1H), 4.04 (ddd, *J* 7.7, 5.0, 2.2 Hz, 1H), 2.74 – 2.63 (m, 2H), 2.57 – 2.47 (m, 2H), 2.46 – 2.35 (m, 2H), 2.18 – 2.06 (m, 2H), 2.02 – 1.94 (m, 1H), 1.79 (ddd, *J* 13.6, 8.4, 5.0 Hz, 1H), 1.66 (ddd, *J* 13.6, 7.7, 5.8 Hz, 1H), 0.94 (dd, *J* 6.6, 3.7 Hz, 6H); ^13^C NMR (DMF-*d*_7_): δ 210.0, 171.4, 169.8, 57.3, 54.2, 44.4, 37.3, 37.3, 36.8, 36.0, 25.1, 23.7, 22.6.

**(3*S*)-3-Benzyl-9-benzylamino-1,4-diazaspiro[5.5]undecane-2,5-dione (14)**

Benzylamine (18 µl, 0.17 mmol) and glacial acetic acid (20 µl, 0.35 mmo.) was added to a mixture of **13** (42 mg, 0.15 mmol) in dry methanol (1.2 ml). The mixture was cooled to 0 °C with an ice bath. PEMB (23 µl, 0.154 mmol) was added and the reaction mixture was slowly allowed to reach r.t. After 3.5 h there was still starting material present according to LCMS analysis. Additional benzylamine (9 µl, 0.09 mmol) was added and the reaction was left at r.t. overnight. The solvent was removed under reduced pressure and the residue was dissolved in DCM, washed with 0.5 M NaOH (aq.) and brine, dried over MgSO_4_, filtered and the solvent was removed under reduced pressure. The crude product was purified on the HPLC to provide **15** as white solids (16 mg, 22%) and as the diastereomeric mixture. ^1^H NMR (CD_3_OD): δ 7.46 – 7.41 (m), 7.31 – 7.17 (m), 4.36 (t, *J* 4.25 Hz), 4.30 (dd, *J* 4.8, 3.6 Hz), 4.17 (s), 4.13 (s), 3.33 – 3.26 (m), 3.24 (d, *J* 3.6 Hz), 3.12 – 2.91 (m), 2.32 – 2.19 (m), 2.12 – 1.57 (m), 1.55 – 1.25 (m), 0.98 – 0.90 (m), 0.45 – 0.39 (m), 0.14 – 0.08 (m); ^13^C NMR (CD_3_OD): δ 170.3, 170.2, 167.4, 167.3, 135.3, 135.2, 131.3, 131.2, 130.4, 130.4, 129.47, 129.46, 129.3, 129.2, 128.91, 128.86, 128.11, 128.09, 126.93, 126.89, 56.5, 55.97, 55.95, 55.7, 55.3, 55.2, 54.3, 38.8, 38.7, 34.8, 34.3, 33.4, 24.8, 24.5, 22.3, 22.2; HRMS *m/z* [M + H]^+^ calculated for C_23_H_27_N_3_O_2_: 378.2181. Found: 378.2217.

**(3*S*)-3-Benzyl-9-*iso*butylamino-1,4-diazaspiro[5.5]undecane-2,5-dione (15)**

Isobutyl amine (80 µl, 0.80 mmol), glacial acetic acid (60 µl, 1.05 mmol) and PEMB (62 µl, 0.41 mmol) were added to a solution of **13** (113 mg, 0.40 mmol) in methanol (2 ml). The reaction mixture was stirred at r.t. overnight. The solvent was removed under reduced pressure and the residue was dissolved in DCM, washed with 0.5 M NaOH (aq) and brine. The crude material was isolated as off white solids/oil. The crude product was purified by HPLC to provide **16** as white solids (13 mg, 10%), as a diastereomeric mixture. ^1^H NMR (CD_3_OD): δ 7.32 – 7.17 (m), 4.37 (t, *J* 4.2 Hz), 4.31 (dd, *J* 4.8, 3.6 Hz), 3.37 – 3.23 (m), 3.07 – 2.90 (m), 2.83 (d, *J* 7.2 Hz), 2.79 (d, *J* 7.2 Hz), 2.29 – 2.18 (m), 2.13 – 1.78 (m), 1.73 – 1.41 (m), 1.36 – 1.25 (m), 1.01 (t, *J* 6.8 Hz), 0.98 – 0.90 (m), 0.44 – 0.38 (m), 0.14 – 0.09 (m); ^13^C NMR (CD_3_OD): δ 171.7, 171.6, 168.8, 168.7, 136.71, 136.69, 131.81, 131.78, 129.5, 129.5, 128.3, 128.3, 57.9, 57.4, 57.2, 57.1, 55.8, 53.3, 52.7, 40.2, 40.1, 36.3, 35.9, 34.8, 34.8, 27.5, 27.5, 26.1, 25.8, 23.5, 23.4, 20.3, 20.3; HRMS *m/z* [M + H]^+^ calculated for C_20_H_29_N_3_O_2_: 344.2338. Found: 344.2357.

**(3*S*)-3-Benzylamino-9-*iso*butyl-1,4-diazaspiro[5.5]undecane-2,5-dione (16)**

Benzylamine (90 µl, 0.82 mmol, 2.15 eq.) and glacial acetic acid (0.55 µl, 0.96 mmol, 2.5 eq.) were added to a solution of **14** (97 mg, 0.38 mmol, 1.0 eq.) dry methanol (3 ml). PEMB (60 µl, 0.40 mmol, 1.05 eq.) was added at r.t. The reaction mixture was stirred at r.t. overnight. LCMS analysis showed full consumption of the starting material. The solvent was removed under reduced pressure and the obtained residue was dissolved in DCM, washed with 0.5 M NaOH (aq.) and brine, dried over MgSO_4_ and the solvent was removed under reduced pressure. A fraction of the crude material was purified by HPLC and **16** was obtained as white solids (30 mg, 23%). ^1^H NMR (CDCl_3_): δ 7.35 – 7.28 (m), 7.27 – 7.20 (m), 3.99 – 3.95 (m), 3.81 (s), 3.76 (s), 2.83 – 2.76 (m), 2.63 – 2.55 (m), 2.41 – 2.31 (m), 2.13 – 1.95 (m), 1.89 – 1.71 (m), 1.67 – 1.57 (m), 1.54 – 1.35 (m), 0.96 (dd, *J* 12.5, 6.1 Hz), 0.91 – 0.82 (m), 0.77 – 0.67 (m); ^13^C NMR (CDCl_3_): δ 171.6, 171.5, 169.33, 169.27, 168.64, 168.58, 140.7, 140.5, 128.6, 128.5, 128.17, 127.16, 127.0, 57.7, 57.6, 57.5, 57.4, 54.4, 53.2, 53.11, 53.08, 52.9, 51.5, 51.3, 51.0, 43.0, 42.8, 34.2, 33.83, 33.75, 32.3, 32.1, 31.8, 29.8, 29.5, 29.1, 27.3, 27.3, 26.8, 26.4, 26.2, 24.4, 23.4, 22.9, 21.3, 14.3, 14.3; HRMS *m/z* [M + H]^+^ calculated for C_20_H_29_N_3_O_2_: 344.2338. Found: 344.2359.

**(3*S*)-1,3-Dibenzyl-1,4-diazaspiro[5.5]undecane-2,5-dione (17S)**

Compound **17S** was synthesised using general procedure C from Boc-L-Phe-OH (345 mg, 1.3 mmol), benzylamine (142 µl, 1.3 mmol), cyclohexanone (135 µl, 1.3 mmol) and 2-morpholinoethyl isocyanide (180 µl, 1.3 mmol). The first step was run in dry methanol (5 ml) for 21 h at 55 °C. Conc HCl (1 ml) was added for the Boc-deprotection (55 °C, overnight). The cyclisation was run in 1.25 M acetic acid and toluene (1:1, 5 ml) at 80 °C for 1 h. Purification by automated flash column chromatography (50% ethyl acetate in pentane) provided **17S** as a light yellow foam (250 mg, 53%). ^1^H NMR (CDCl_3_): δ 7.43 – 7.19 (m, 8H), 7.21 – 7.09 (m, 2H), 5.62 (s, 1H), 4.92 (d, *J* 16.1 Hz, 1H), 4.63 (d, *J* 16.1 Hz, 1H), 4.32 (ddd, *J* 9.7, 3.7, 1.2 Hz, 1H), 3.58 (dd, *J* 14.0, 3.7 Hz, 1H), 2.93 (dd, *J* 14.0, 9.7 Hz, 1H), 2.19 – 2.08 (m, 1H), 1.94 – 1.89 (m, 1H), 1.73 – 1.55 (m, 7H), 1.16 – 0.92 (m, 1H); ^13^C NMR (CDCl_3_): δ 171.1, 168.2, 138.3, 136.0, 129.8, 129.0, 128.5, 127.4, 126.9, 126.4, 62.9, 55.5, 45.5, 39.5, 33.7, 33.0, 24.5, 22.9, 22.5; HRMS *m/z* [M + H]^+^ calculated for C_23_H_26_N_2_O_2_: 363.2072. Found: 363.2064; $\alpha_{D}^{20}$ = -75.4 (c 0.1, CH_2_Cl_2_).

**(3*R*)-1,3-Dibenzyl-1,4-diazaspiro[5.5]undecane-2,5-dione (17R)**

Compound **17R** was synthesised using general procedure C from Boc-D-Phe-OH (345 mg, 1.3 mmol), benzylamine (142 µl, 1.3 mmol), cyclohexanone (135 µl, 1.3 mmol) and 2-morpholinoethyl isocyanide (180 µl, 1.3 mmol). The first step was run in dry methanol (7.2 ml) for 20 h at 55 °C. Conc HCl (1.9 ml) was added for the Boc-deprotection (55 °C for 3 h). The cyclisation was run in 1.25 M acetic acid and toluene (1:1, 7 ml) at 80 °C for 4 h. Purification by flash column chromatography (40% ethyl acetate in pentane) provided **17R** as a thick, yellow oil (384 mg, 56%). ^1^H NMR (CDCl_3_): δ 7.40 – 7.20 (m, 8H), 7.17 – 7.13 (m, 2H), 5.64 (s, 1H), 4.92 (d, *J* 16.2 Hz, 1H), 4.63 (d, *J* 16.1 Hz, 1H), 4.32 (ddd, *J* 9.7, 3.7, 1.2 Hz, 1H), 3.58 (dd, *J* 14.1, 3.6 Hz, 1H), 2.93 (dd, *J* 14.0, 9.6 Hz, 1H), 2.21 – 2.07 (m, 1H), 1.96 – 1.87 (m, 1H), 1.76 – 1.51 (m, 7H), 1.12 – 0.98 (m, 1H); ^13^C NMR (CDCl_3_): δ 170.7, 168.4, 138.4, 136.0, 129.7, 129.3, 128.7, 127.7, 127.1, 126.6, 63.1, 55.5, 45.60, 39.8, 33.8, 33.0, 24.6, 23.0, 22.6; HRMS *m/z* [M + H]^+^ calculated for C_23_H_26_N_2_O_2_: 363.2072. Found: 363.2080; $\alpha_{D}^{20}$ = 98.2 (c 0.1, CH_2_Cl_2_).

**(3*S*)-3-Benzyl-1-phenethyl-1,4-diazaspiro[5.5]undecane-2,5-dione (18S)**

Compound **18S** was synthesised using general procedure C from Boc-L-Phe-OH (50 mg, 0.19 mmol), phenethylamine (24 µl, 0.19 mmol), cyclohexanone (20 µl, 0.19 mmol) and 2-morpholinoethyl isocyanide (26 µl, 0.19 mmol). The first step was run in dry methanol (5 ml) 12 h at 55 °C. HCl in Methanol (1.25 M, 2 ml) was added for the Boc-deprotection (55 °C for 2.5 h). The cyclisation was run in 1.25 M acetic acid and toluene (1:1, 5 ml) at 80 °C for 2 h. Purification by automated flash column chromatography (50% ethyl acetate in pentane) provided **18S** as a colourless oil (25 mg, 61%). ^1^H NMR (CDCl_3_): δ 7.37 – 7.19 (m, 10H), 6.02 (s, 1H), 4.26 (ddd, *J* 8.6, 3.7, 1.5 Hz, 1H), 3.65 (ddd, 13.6, 11.0, 5.8, 1H), 3.50 (ddd, 13.6, 10.8, 5.5, 1H), 3.43 (dd, *J* 13.9, 3.7 Hz, 1H), 2.96 (dd, *J* 13.9, 8.6 Hz, 1H), 2.84 (qdd, 12.9, 10.7, 5.6 Hz, 2H), 2.20 – 2.05 (m, 1H), 1.97 – 1.78 (m, 2H), 1.74 – 1.54 (m, 6H), 1.15 –1.04 (m, 1H); ^13^C NMR (CDCl_3_): δ 170.6, 166.9, 138.9, 135.8, 129.7, 129.0, 128.8, 128.6, 127.4, 126.5, 62.3, 55.5, 44.7, 40.1, 35.9, 33.6, 32.8, 24.4, 22.7, 22.5; HRMS *m/z* [M + H]^+^ calculated for C_24_H_28_N_2_O_2_: 377.2229. Found: 377.2232; $\alpha_{D}^{20}$ = -21.4 (c 0.1, CH_2_Cl_2_).

**(3*S*)-3-Benzyl-1-(4-chlorobenzyl)-1,4-diazaspiro[5.5]undecane-2,5-dione (19S)**

Compound **19S** was synthesised using general procedure C from Boc-L-Phe-OH (1.00 g, 3.8 mmol), 4-chlorobenzylamine (0.46 ml, 3.8 mmol), cyclohexanone (0.4 ml, 3.8 mmol) and 2-morpholinoethyl isocyanide (0.53 ml, 3.8 mmol). The first step was run in dry methanol (13 ml) overnight at 55 °C. Conc. HCl (12 M, 3.5 ml) was added for the Boc-deprotection (55 °C for 4 h). The cyclisation was run in 1.25 M acetic acid and toluene (1:1, 20 ml) at 80 °C for 2.5 h. Purification by automated flash column chromatography (10-50% ethyl acetate in pentane) failed to provide pure **19S**, one additional automated flash column (0-2.5% methanol in DCM) provided **19S** as a light yellow foam (650 mg, 43%).^1^H NMR (CDCl_3_): δ 7.40 – 7.29 (m, 3H), 7.28 – 7.24 (m, 4H), 7.10 – 7.05 (m, 2H), 5.70 (s, 1H), 4.86 (d, *J* 16.2 Hz, 1H), 4.57 (d, *J* 16.2 Hz, 1H), 4.32 (ddd, *J* 9.3, 3.7, 1.2 Hz, 1H), 3.53 (dd, *J* 14.0, 3.7 Hz, 1H), 2.95 (dd, *J* 14.0, 9.3 Hz, 1H), 2.21 – 2.08 (m, 1H), 1.94 – 1.85 (m, 1H), 1.75 – 1.50 (m, 7H), 1.14 – 0.97 (m, 1H). ^13^C NMR (CDCl_3_): δ 170.5, 168.4, 136.9, 135.9, 132.9, 129.7, 129.3, 128.9, 128.1, 127.7, 63.1, 55.5, 45.1, 39.7, 33.8, 33.1, 24.6, 23.0, 22.6; HRMS *m/z* [M + H]^+^ calculated for C_23_H_25_ClN_2_O_2_: 397.1683. Found: 397.1665; $\alpha_{D}^{20}$ = -73.6 (c 0.1, CH_2_Cl_2_).

**(3*S*)-1-Benzyl-3-(*tert*butoxycarbonyl-methyl)-1,4-diazaspiro[5.5]undecane-2,5-dione (20S)**

Boc-L-Asp(OtBu)-OH (120 mg, 0.42 mmol) was dissolved in dry methanol (2 ml), benzylamine (45 µl, 0.42 mmol), cyclohexanone (43 µl, 0.42 mmol) and 2-morpholinoethyl isocyanide (58 µl, 0.42 mmol) were added at r.t. under N_2_ (g) in an oven dried round bottomed flask. The reaction was stirred at 55 °C overnight followed by 48 h at r.t. The solvent was removed under reduced pressure and the obtained residue was dissolved in ethyl acetate, washed with 0.5 M HCl (aq.), sat. NaHCO_3_ (aq.), brine, dried over Na_2_SO_4_ and the solvent was removed under reduced pressure. The obtained residue was dissolved in dry DCM (0.5 ml), TFA (0.5 ml) was added at r.t. and the reaction was stirred for 2 h. 3 M KOH (aq.) was added until the water phase had a pH of above 12. The water phase was extracted with DCM (x3). The combined organic phases were dried over MgSO_4_, filtered and the solvent was removed under reduced pressure. The residue was suspended in 1.25 M acetic acid and toluene (1:1, 2 ml) and heated at 80 °C for 2 h. The reaction was allowed to reach r.t. and diluted with ethyl acetate (220 ml), the organic phase was washed with small amounts of water (2x30 ml), sat. NaHCO_3_ (40 ml) and brine, dried over Na_2_SO_4_, filtered and the solvent was removed under reduced pressure. The crude product was obtained as a yellow/white solids. The crude product was purified by automatic flash chromatography (10-15% ethyl acetate in pentane) to provide **20S** as a colourless oil (38 mg, 24%). ^1^H NMR (CDCl_3_): δ 7.32 – 7.26 (m, 2H), 7.25 – 7.15 (m, 3H), 6.41 (s, 1H), 4.86 (d, *J* 16.2 Hz, 1H), 4.65 (d, *J* 16.2 Hz, 1H), 4.44 (ddd, *J* 10.2, 3.0, 1.4 Hz, 1H), 3.21 (dd, *J* 17.3, 3.0 Hz, 1H), 2.62 (dd, *J* 17.4, 10.2 Hz, 1H), 2.16 (qt, *J* 13.1, 4.0 Hz, 1H), 2.00 – 1.86 (m, 2H), 1.79 – 1.54 (m, 6H), 1.48 (s, 9H), 1.08 (qt, *J* 12.7, 3.6 Hz, 1H); ^13^C NMR (CDCl_3_): δ 170.5, 170.5, 167.9, 138.3, 128.7, 127.1, 126.6, 82.3, 63.1, 51.5, 45.6, 39.4, 33.7, 33.4, 28.2, 24.6, 23.0, 22.6; HRMS *m/z* [M + H]^+^ calculated for C_22_H_30_N_2_O_4_: 387.2284. Found: 387.2263; $\alpha_{D}^{20}$ = -2.6 (c 0.1, CH_2_Cl_2_).

**(3*S*)-1-Benzyl-3-(methoxycarbonyl-methyl)-1,4-diazaspiro[5.5]undecane-2,5-dione (21S)**

Compound **21S** was synthesised using general procedure C from Boc-L-Asp(OtBu)-OH (240 mg, 0.83 mmol), benzylamine (0.1 ml, 0.92 mmol), cyclohexanone (0.1 ml, 0.96 mmol) and 2-morpholinoethyl isocyanide (0.12 ml, 0.86 mmol). The first step was run in dry methanol (13 ml) for 40 h at 55 °C. Conc. HCl (12 M, 3.5 ml) was added for the Boc-deprotection (55 °C for 6 h). *Note, all of the tertbutyl ester was converted to the methyl ester.* The cyclisation was run in 1.25 M acetic acid and toluene (1:1, 20 ml) at 80 °C for 2.5 h. Purification by automatic flash column chromatography (15-20% ethyl acetate in pentane) provided **21S** as a colourless oil (105 mg, 37%). ^1^H NMR (CDCl_3_): δ 7.33 – 7.26 (m, 2H), 7.24 – 7.16 (m, 3H), 7.09 – 6.99 (m, 1H), 4.89 (d, *J* 16.2 Hz, 1H), 4.62 (d, *J* 16.2 Hz, 1H), 4.48 (dd, *J* 8.8, 3.8, 1H), 3.73 (s, 3H), 3.22 (dd, *J* 17.2, 3.8 Hz, 1H), 2.77 (dd, *J* 17.2, 8.8 Hz, 1H), 2.22 – 2.11 (m, 1H), 2.04 – 1.86 (m, 2H), 1.78 – 1.54 (m, 6H), 1.14 – 0.99 (m, 1H); ^13^C NMR (CDCl_3_): δ 171.3, 171.1, 167.9, 138.2, 128.7, 127.1, 126.5, 63.1, 52.3, 51.3, 45.6, 37.7, 33.6, 33.1, 24.6, 23.0, 22.6; HRMS *m/z* [M + H]^+^ calculated for C_19_H_24_N_2_O_4_: 345.1814. Found: 345.1777; $\alpha_{D}^{20}$ = -25.9 (c 0.1, CH_2_Cl_2_).

**1,3-Dibenzyl-6-phenylpiperazine-2,5-dione (22RR and 22RS)**

Compounds **22RR** and **22RS** were synthesised following general procedure C from Boc-D-Phe-OH (502 mg, 1.89 mmol), benzylamine (200 µl, 1.82 mmol), benzaldehyde (190 µl, 1.87 mmol) and 2-morpholinoethyl isocyanide (260 µl, 1.87 mmol). The first step was run in dry methanol (7.3 ml) overnight at 55 °C. Conc. HCl (2 ml) was added for the Boc-deprotection (55 °C for 3 h). The cyclisation was run in 1.25 M acetic acid and toluene (1:1, 10 ml) at 80 °C for 3.5 h. Purification by automated flash column chromatography (50% ethyl acetate in pentane) provided **22RR** (109 mg, 16%) and **22RS** (160 mg, 23%) as off white/yellow solids in a total yield of 39%. **22RR**: ^1^H NMR (CDCl_3_): δ 7.41 – 7.32 (m, 3H), 7.31 – 7.20 (m, 10H), 7.08 – 6.87 (m, 2H), 6.70 (s, 1H,), 5.53 (d, *J* 14.8 Hz, 1H), 4.68 (s, 1H), 4.45 (dd, *J* 8.0, 3.8 Hz, 1H,), 3.46 (dd, *J* 14.1, 3.9 Hz, 1H), 3.45 (d, *J* 14.8 Hz, 1H), 3.13 (dd, *J* 14.1, 8.0 Hz, 1H); ^13^C NMR (CDCl_3_): δ 166.5, 166.1, 135.5, 135.4, 135.1, 129.9, 129.4, 129.0, 129.0, 128.9, 128.4, 128.0, 127.5, 127.0, 62.8, 55.3, 47.3, 39.2; HRMS *m/z* [M+H]^+^ calculated for C_24_H_22_N_2_O_2_: 371.1759. Found 371.1742; $\alpha_{D}^{20}$ = 58.0 (c 0.1, CH_2_Cl_2_). **22RS**: ^1^H NMR (CDCl_3_): δ 7.31 – 7.09 (m, 11H), 7.01 – 6.97 (m, 2H), 6.81 – 6.77 (m, 2H), 5.49 (d, *J* 14.6 Hz, 1H), 4.74 (s, 1H), 4.41 – 4.36 (m,1H), 3.34 (d, *J* 14.6 Hz, 1H), 3.21 – 3.08 (m, 2H); ^13^C NMR (CDCl_3_): δ 166.3, 165.2, 135.6, 135.5, 135.2, 130.1, 129.1, 129.0, 128.9, 128.8, 128.7, 128.2, 127.3, 127.3, 62.3, 57.0, 47.2, 40.6; HRMS *m/z* [M+H]^+^ calculated for C_24_H_22_N_2_O_2_: 371.1759. Found: 371.1742; $\alpha_{D}^{20}$ = 98.8 (c 0.1, CH_2_Cl_2_).

**3-Benzyl-1-(4-chlorobenzyl)-6-phenylpiperazine-2,5-dione (23RR and 23RS)**

Benzaldehyde (382 µl, 3.77 mmol) and 4-chloro benzylamine (460 µl, 3.77 mmol) was added to a solution of dry methanol (13 ml) and molecular sieves. The flask was capped and flushed with N_2_ and stirred for 3 h at r.t. Boc-D-Phe-OH (1.00 g, 3.77 mmol) followed by 2-morpholinoethyl isocyanide (523 µl, 3.77 mmol) were added and the reaction was heated to 55 °C for 3 days. The molecular sieves were filtered off and conc. HCl (5 ml) was added. The reaction mixture was heated to 55 °C for 4 h. The solvent was evaporated and the obtained oil was dissolved in distilled water (25 ml) and sat. NaHCO_3_ (25 ml) was added and a white precipitation was observed. The aqueous phase was extracted with ethyl acetate (3 x 30 ml) and the combined organic phases were dried over Na_2_SO_4_, filtered and the solvent was removed under reduced pressure. 1.25 M acetic acid (aq.) and toluene (1:1, 40 ml) were added to the oil and the reaction mixture was heated to 80 °C overnight. The solution was diluted with ethyl acetate (30 ml) and the aqueous phase was extracted with ethyl acetate (x2). The combined organic phases were washed with distilled water, NaHCO_3_ (aq.), and brine. The organic phase was dried over Na_2_SO_4_, filtered and the solvent was removed under reduced pressure. Purification by automated flash column chromatography (40-75% ethyl acetate in pentane) provided **23RR** (226 mg, 15%) and **23RS** (206 mg, 14%) as yellow solids in a total yield of 29%. **23RR**: ^1^H NMR (CDCl_3_): δ 7.43 – 7.22 (m, 12H), 6.95 – 6.87 (m, 2H), 6.41(*br* s, 1H), 5.44 (d, *J* 14.9 Hz, 1H), 4.67 (s, 1H), 4.47 (dd, *J* 8.3, 3.7 Hz, 1H), 3.53 – 3.45 (m, 2H), 3.12 (dd, *J* 14.1, 8.1 Hz, 1H); ^13^C NMR (CDCl_3_): δ 166.30, 166.2, 135.3, 135.1, 134.0, 133.7, 129.9, 129.8, 129.5, 129.2, 129.1, 128.8, 127.7, 126.9, 63.1, 55.2, 47.0, 39.3; HRMS *m/z* [M+H]^+^ calculated for C_24_H_21_ClN_2_O_2_: 405.1370. Found: 405.1362; $\alpha_{D}^{20}$ = 86.6 (c 0.1, CH_2_Cl_2_). **23RS**: ^1^H NMR (CDCl_3_): δ 7.31 – 7.01 (m, 13H), 6.84 – 6.78 (m, 2H), 5.39 (d, *J* 14.7 Hz, 1H), 4.75 (s, 1H), 4.43 – 4.41 (m, 1H), 3.42 (d, *J* 14.7 Hz, 1H), 3.20 – 3.15 (m, 2H); ^13^C NMR (CDCl_3_): δ 166.0, 165.3, 135.5, 135.3, 134.1, 133.8, 130.2, 130.1, 129.2, 129.07, 129.06, 128.9, 127.4, 127.3, 62.6, 57.0, 46.8, 40.6; HRMS *m/z* [M+H]^+^ calculated for C_24_H_21_ClN_2_O_2_: 405.1370. Found: 405.1362; $\alpha_{D}^{20}$ = 66.2 (c 0.1, CH_2_Cl_2_).

**3-Benzyl-1,6-diphenylpiperazine-2,5-dione (24RR and 24RS)**

Compounds **24RR** and **24RS** were synthesised following general procedure C from Boc-D-Phe-OH (500 mg, 1.89 mmol), anline (179 µl, 1.88 mmol), benzaldehyde (191 µl, 1.88 mmol) and 2-morpholinoethyl isocyanide (260 µl, 1.88 mmol). The first step was run in dry methanol (6.5 ml) for 48 h at 55 °C. Conc. HCl (12 M, 1.5 ml) was added for the Boc-deprotection (55 °C, overnight). The cyclisation was run in 1.25 M acetic acid and toluene (1:1, 8 ml) at 80 °C overnight. Purification by automated flash column chromatography (5% methanol in DCM) provided **24RR** (97 mg, 14%) and **24RS** (97 mg, 14%) as off white solids in a total yield of 28%. **24RR**: ^1^H NMR (CDCl_3_): δ 7.31 – 7.12 (m, 13H), 7.01 – 6.96 (m, 2H), 6.35 (s, 1H), 5.07 (s, 1H), 4.38 (dd, *J* 8.6, 3.9 Hz, 1H), 3.47 (dd, *J* 14.2, 3.9 Hz, 1H), 3.02 (dd, *J* 14.2, 8.6 Hz, 1H); ^13^C NMR (CDCl_3_): δ 167.2, 166.5, 139.9, 135.50, 135.47, 129.8, 129.3, 129.23, 129.21, 128.9, 127.7, 127.6, 126.8, 126.3, 67.8, 55.5, 38.7; HRMS *m/z* [M + H]^+^ calculated for C_23_H_20_N_2_O_2_: 357.1603. Found: 357.1572; $\alpha_{D}^{20}$ = 156.2 (c 0.1, CH_2_Cl_2_). **24RS**: ^1^H NMR (CDCl_3_): δ 6.35 (s, 1H), 7.40 – 6.99 (m, 13H), 6.74 – 6.67 (m, 2H), 5.30 (s, 1H), 4.56 – 4.48 (m, 1H), 3.25 (dd, *J* 13.6, 6.8 Hz, 1H), 3.13 (dd, *J* 13.6, 4.1 Hz, 1H); ^13^C NMR (CDCl_3_): δ 166.8, 165.2, 139.5, 136.2, 135.7, 130.4, 129.2, 129.0, 128.7, 128.5, 127.7, 127.6, 127.3, 126.9, 67.1, 57.2, 40.2; HRMS *m/z* [M + H]^+^ calculated for C_23_H_20_N_2_O_2_: 357.1603. Found: 357.1600; $\alpha_{D}^{20}$ = 28.8 (c 0.1, CH_2_Cl_2_).

**3-Benzyl-1-(4-chlorophenyl)-6-phenylpiperazine-2,5-dione (25RR and 25RS)**

Compounds **25RR** and **25RS** was synthesised following general procedure C from Boc-D-Phe-OH (500 mg, 1.89 mmol), 4-chloroaniline (241 mg, 1.88 mmol), benzaldehyde (200 mg, 1.88 mmol) and 2-morpholinoethyl isocyanide (0.26 ml, 1.88 mmol). The first step was run in dry methanol (6.7 ml) for 48 h at 55 °C. Conc. HCl (12 M, 1.5 ml) was added for the Boc-deprotection (55 °C for 3 h). The cyclisation was run in 1.25 M acetic acid and toluene (1:1, 8 ml) at 80 °C for 12 h. Purification by automated column chromatography (50-80% ethyl acetate in pentane) provided **25RR** (50 mg, 7%) as a yellow solid and **25RS** (52 mg, 7%) as a yellow oil in a total yield of 14%. **25RR** ^1^H NMR (CDCl_3_): δ 7.39 – 7.22 (m, 12H), 7.00 – 6.94 (m, 2H), 6.38 (s, 1H), 5.06 (s, 1H), 4.45 (dd, *J* 8.5, 3.9 Hz, 1H), 3.52 (dd, *J* 14.2, 3.9 Hz, 1H), 3.08 (dd, *J* 14.2, 8.5 Hz, 1H); ^13^C NMR (CDCl_3_): δ 166.8, 166.3, 138.1, 135.2, 135.1, 133.2, 129.7, 129.4, 129.3, 129.2, 129.0, 127.7, 127.5, 126.6, 67.5, 55.4, 38.7; LRMS *m/z* [M + H]^+^ calculated for C_23_H_19_ClN_2_O_2_: 391.1. Found: 391.6. **25RS** ^1^H NMR (CDCl_3_): δ 7.39 – 7.14 (m, 8H), 7.13 – 7.07 (m, 2H), 6.99 – 6.94 (m, 2H), 6.76 – 6.70 (m, 2H), 5.25 (s, 1H), 4.53 – 4.48 (m, 1H), 3.19 (dd, *J* 5.6, 4.2 Hz, 2H); ^13^C NMR (CDCl_3_): δ 166.3, 165.2, 137.8, 135.8, 135.4, 133.4, 130.2, 129.4, 129.0, 128.8, 128.7, 128.2, 127.4, 127.4, 66.9, 57.2, 40.3; LRMS *m/z* [M + H]^+^ calculated for C_23_H_19_ClN_2_O_2_: 391.1. Found: 391.6.

**3-Benzyl-6-(4-chlorophenyl)-1-phenylpiperazine-2,5-dione (26RR and 26RS)**

Compounds **26RR** and **26RS** was synthesised following general procedure C from Boc-D-Phe-OH (500 mg, 1.88 mmol), aniline (0.17 ml, 1.88 mmol), 4-chlorobenzaldehyde (265 mg, 1.89 mmol) and 2-morpholinoethyl isocyanide (0.26 ml, 1.88 mmol). The first step was run in dry methanol (6.5 ml) for 48 h at 55 °C. Conc. HCl (12 M, 1.5 ml) was added for the Boc-deprotection (55 °C for 3 h). The cyclisation was run in 1.25 M acetic acid and toluene (1:1, 8 ml) at 80 °C for 16 h. Purification by automatic flash column chromatography (30-80% ethyl acetate in pentane) provided **26RR** (33 mg, 5%) as an yellow solid and **26RS** (62 mg, 8%) as a yellow foam/oil, in a total yield of 13%. **26RR:** ^1^H NMR (CDCl_3_): δ 7.40 – 7.22 (m, 12H), 7.06 – 7.02 (m, 2H), 6.35 (s, 1H), 5.08 (s, 1H), 4.46 (dd, *J* 8.5, 3.9 Hz, 1H), 3.54 (dd, *J* 14.2, 3.9 Hz, 1H), 3.11 (dd, *J* 14.2, 8.5 Hz, 1H); ^13^C NMR (CDCl_3_): δ 166.8, 166.3, 139.6, 135.3, 135.0, 134.1, 129.8, 129.44, 129.41, 129.3, 128.3, 127.83, 127.81, 126.3, 67.1, 55.6, 39.0; LRMS *m/z* [M + H]^+^ calculated for C_23_H_19_ClN_2_O_2_: 391.1. Found: 391.7. **26RS:** ^1^H NMR (CDCl_3_): δ 7.59 – 7.49 (m, 1H), 7.44 – 7.37 (m, 1H), 7.35 – 7.27 (m, 4H), 7.24 – 7.18 (m, 1H), 7.16 – 7.12 (m, 2H), 7.08 (d, *J* 6.9 Hz, 2H), 7.02 – 6.92 (m, 2H), 6.50 (d, *J* 8.2 Hz, 2H), 5.25 (s, 1H), 4.57 – 4.54 (m, 1H), 3.33 (dd, *J* 13.6, 6.3 Hz, 1H), 3.12 (dd, *J* 13.7, 4.0 Hz, 1H); ^13^C NMR (CDCl_3_): δ 166.1, 164.8, 139.0, 135.4, 134.7, 134.4, 130.5, 129.3, 128.99, 128.96, 128.8, 127.8, 127.5, 126.9, 66.3, 57.0, 40.1; LRMS *m/z* [M + H]^+^ calculated for C_23_H_19_ClN_2_O_2_: 391.1. Found: 391.7.

**3-Benzyl-6-(3-chlorophenyl)-1-phenylpiperazine-2,5-dione (27RR and 27RS)**

Compounds **27RR** and **27RS** was synthesised following general procedure C from Boc-D-Phe-OH (500 mg, 1.88 mmol), aniline (0.17 ml, 1.88 mmol), 3-chlorobenzaldehyde (265 mg, 1.89 mmol) and 2-morpholinoethyl isocyanide (0.26 ml, 1.88 mmol). The first step was run in dry methanol (6.5 ml) for 48 h at 55 °C. Conc. HCl (12 M, 1.5 ml) was added for the Boc-deprotection (55 °C for 3 h). The cyclisation was run in 1.25 M acetic acid and toluene (1:1, 8 ml) at 80 °C for 16 h. Purification by automatic flash column chromatography (30-80% ethyl acetate in pentane) provided **27RR** (56 mg, 8%) as an yellow solid and **27RS** (74 mg, 10%) as a yellow oil, in a total yield of 18%. **27RR:** ^1^H NMR (CDCl_3_): δ 7.40 – 7.18 (m, 12H), 7.07 – 7.01 (m, 2H), 6.57 (br s, 1H), 5.06 (s, 1H), 4.48 (dd, *J* 8.2, 3.9 Hz, 1H), 3.52 (dd, *J* 14.2, 3.9 Hz, 1H), 3.13 (dd, *J* 14.1, 8.2 Hz, 1H); ^13^C NMR (CDCl_3_): δ 166.6, 166.2, 139.5, 137.6, 135.3, 135.2, 130.4, 129.9, 129.4, 129.2, 129.2, 127.83, 127.78, 127.1, 126.3, 125.1, 67.1, 55.5, 38.9. **27RS** ^1^H NMR (CDCl_3_): δ 7.41 – 6.98 (m, 12H), 6.94 – 6.91 (m, 1H), 6.59 – 6.42 (m, 1H), 5.29 (s, 1H), 4.53 – 5.50 (m, 1H), 3.72 (s, 1H), 3.30 – 3.13 (m, 1H); ^13^C NMR (CDCl_3_): δ 166.0, 165.1, 139.1, 138.0, 135.3, 134.5, 130.2, 129.9, 129.3, 128.9, 128.8, 128.0, 127.9, 127.7, 126.8, 125.5, 66.4, 57.1, 40.4.

**1-(4-Chlorophenyl)-6-(3-chlorophenyl)-3-methyl-piperazine-2,5-dione (29SS and 29SR)**

Compounds **29SS** and **29SR** were synthesised following general procedure C from Boc-Ala-OH (1.00 g, 5.29 mmol), 4-chloroaniline (674 mg, 5.29 mmol), 3-chlorobenzaldehyde (602 µl, 5.29 mmol) and 2-morpholinoethyl isocyanide (729 µl, 5.29 mmol). The first step was run in dry methanol (20 ml) for 3 days at 55 °C. Conc. HCl (12 M, 4 ml) was added for the Boc-deprotection (55 °C for 3 h). The cyclisation was run in 1.25 M acetic acid and toluene (1:1, 8 ml) at 80 °C for 12 h. Purification by automatic flash column chromatography (0-80% ethyl acetate in pentane) provided **29SS** (25 mg) as an colourless oil and **29SR** (22 mg) as a colourless oil, in a total yield of 3%. **29SS** ^1^H NMR (CDCl_3_): δ 7.44 – 7.43 (m, 1H), 7.38 – 7.27 (m, 5H), 7.19 – 7.12 (m, 2H), 6.87 (br s, 1H), 5.34 (s, 1H), 4.21 (q, *J* 6.8 Hz, 1H), 1.58 (d, *J* 6.8 Hz, 2H); ^13^C NMR (CDCl_3_): δ 167.7, 167.0, 138.4, 136.3, 135.6, 133.1, 130.6, 129.5, 129.3, 129.0, 126.8, 126.3, 124.2, 121.0, 67.7, 50.0, 17.4; HRMS m/z [M + H]+ calculated for C17H14Cl2N2O4: 349.0510. Found: 349.0500. **29SR** ^1^H NMR (CDCl_3_): δ 7.43 – 7.37 (m, 1H), 7.34 – 7.28 (m, 5H), 7.14 – 7.08 (m, 2H), 6.79 (br s, 1H), 5.33 (s, 1H), 4.36 (qd, *J* 7.1, 3.1 Hz, 1H), 1.59 (d, *J* 7.2 Hz, 3H); ^13^C NMR (CDCl_3_): δ 167.0, 165.6, 137.8, 137.8, 135.2, 133.5, 130.3, 129.6, 129.2, 127.6, 127.3, 125.0, 66.5, 52.1, 22.1; HRMS *m/z* [M + H]^+^ calculated for C_17_H_14_Cl_2_N_2_O_4_: 349.0510. Found: 349.0508.

**(3*S*)-1,3-Dibenzyl-4-(ethoxycarbonyl-methyl)-1,4-diazaspiro[5.5]undecane-2,5-dione (30S)**

Compound **30S** was synthesised following general procedure D from **17S** (400 mg, 1.10 mmol), BEMP (0.64 ml, 2.21 mmol), ethyl bromoacetate (0.25 ml, 2.25 mmol). The reaction was stirred at r.t. overnight. Purification by automatic flash chromatography (10-20% ethyl acetate in pentane) provided **30S** as a thick colourless oil (451 mg, 91%). ^1^H NMR (CDCl_3_): δ 7.35 – 7.12 (m, 10H), 5.00 (d, *J* 16.1 Hz, 1H), 4.47 – 4.44 (m, 1H), 4.42 (d, 17.0, 1H), 4.34 (d, *J* 16.1 Hz, 1H), 4.19 (qd, *J* 7.2, 1.1 Hz, 2H), 3.49 (d, *J* 17.0 Hz, 1H), 3.34 (dd, *J* 14.2, 5.8 Hz, 1H), 3.25 (dd, *J* 14.2, 4.3 Hz, 1H), 2.24 – 2.10 (m, 1H), 1.88 – 1.79 (m, 1H), 1.65 – 1.46 (m, 4H), 1.43 – 1.32 (m, 2H), 1.27 (t, *J* 7.1 Hz, 3H), 1.05 – 0.91 (m, 1H), 0.89 – 0.79 (m, 1H); ^13^C NMR (CDCl_3_): δ 169.9, 168.3, 167.0, 138.1, 136.2, 129.9, 128.9, 128.6, 127.4, 126.9, 126.3, 62.6, 62.5, 61.6, 47.2, 45.9, 38.7, 34.6, 33.5, 24.4, 23.1, 22.3, 14.2; HRMS *m/z* [M + H]^+^ calculated for C_27_H_32_N_2_O_4_: 449.2440. Found: 449.2459; $\alpha_{D}^{20}$ = -13.4 (c 0.1, CH_2_Cl_2_).

**(3*S*)-1,3-Dibenzyl-4-(*tert*butoxycarbonyl-methyl)-1,4-diazaspiro[5.5]undecane-2,5-dione (31S)**

Compound **31S** was synthesised following general procedure D from **17S** (305 mg, 0.84 mmol), BEMP (0.5 ml, 1.73 mmol) and *tert*-butyl bromoacetate (0.25 ml, 1.70 mmol). The reaction was stirred at r.t. overnight. Purification by automatic flash chromatography (10-20% ethyl acetate in pentane) provided **31S** as a colourless foam (397 mg, 99%). ^1^H NMR (CDCl_3_): δ 7.32 – 7.18 (m, 6H), 7.17 – 7.12 (m, 4H), 4.95 (d, *J* 16.1 Hz, 1H), 4.45 (t, *J* 4.8 Hz, 1H), 4.41 (d, *J* 16.8 Hz, 1H), 4.33 (d, *J* 16.1 Hz, 1H), 3.42 (d, *J* 16.8 Hz, 1H), 3.36 (dd, *J* 14.2, 5.2 Hz, 1H), 3.21 (dd, *J* 14.2, 4.4 Hz, 1H), 2.23 – 2.12 (m, 1H), 1.86 – 1.78 (m, 1H), 1.66 – 1.50 (m, 4H), 1.46 (s, 9H), 1.37 – 1.26 (m, 2H), 1.02 – 0.90 (m, 1H), 0.69 – 0.64 (m, 1H); ^13^C NMR (CDCl_3_): δ 169.6, 167.2, 167.0, 138.1, 136.2, 130.0, 128.9, 128.5, 127.4, 126.8, 126.4, 82.2, 62.5, 62.2, 47.8, 45.9, 38.4, 34.3, 33.6, 28.2, 24.3, 23.0, 22.3; HRMS *m/z* [M + H]^+^ calculated for C_29_H_36_N_2_O_4_: 477.2753. Found: 477.2740; $\alpha_{D}^{20}$ = -16.1 (c 0.1, CH_2_Cl_2_).

**(3*R*)-1,3-Dibenzyl-4-(ethoxycarbonyl-methyl)-1,4-diazaspiro[5.5]undecane-2,5-dione (30R)**

Compound **30R** was synthesised following general procedure D from **17R** (150 mg, 0.41 mmol), BEMP (0.24 ml, 0.83 mmol) and ethyl bromoacetate (0.1 ml, 0.9 mmol). The reaction was stirred at r.t. overnight. Purification by automatic flash chromatography (15-20% ethyl acetate in pentane) provided **30R** as colourless oil (161 mg, 87%). ^1^H NMR (CDCl_3_): δ 7.32 – 7.12 (m, 10H), 4.99 (d, *J* 16.1 Hz, 1H), 4.48 – 4.38 (m, 2H), 4.33 (d, *J* 16.2 Hz, 1H), 4.18 (qd, *J* 7.2, 1.2 Hz, 2H), 3.49 (d, *J* 17.1 Hz, 1H), 3.33 (dd, *J* 14.2, 5.7 Hz, 1H), 3.24 (dd, *J* 14.1, 4.3 Hz, 1H), 2.25 – 2.11 (m, 1H), 1.86 – 1.80 (m, 1H), 1.66 – 1.45 (m, 4H), 1.43 – 1.31 (m, 2H), 1.26 (t, *J* 7.2 Hz, 3H), 1.04 – 0.90 (m, 1H), 0.86 – 0.76 (m, 1H); ^13^C NMR (CDCl_3_): δ 169.8, 168.2, 166.9, 138.0, 136.1, 129.9, 128.8, 128.5, 127.4, 126.8, 126.3, 62.5, 62.4, 61.5, 47.1, 45.8, 38.6, 34.5, 33.4, 24.3, 23.0, 22.2, 14.1; HRMS *m/z* [M + H]^+^ calculated for C_27_H_32_N_2_O_4_: 449.2440. Found: 449.2433; $\alpha_{D}^{20}$ = 24.6 (c 0.1, CH_2_Cl_2_).

**(3*S*)-1,3-Dibenzyl-4-(ethoxy-carbonyl)-1,4-diazaspiro[5.5]undecane-2,5-dione (32S)**

Compound **32S** was synthesised following general procedure D from **17S** (150 mg, 0.41 mmol), BEMP (0.24 ml, 0.83 mmol) and ethyl chloroformate (0.08 ml, 0.84 mmol) in dry DCM (11 ml). The reaction was stirred at r.t. overnight. Purification by automatic flash column chromatography (15-20% ethyl acetate in pentane) provided **32S** as colourless oil (160 mg, 89%). ^1^H NMR (CDCl_3_): δ 7.32 – 7.16 (m, 6H), 7.19 – 7.06 (m, 4H), 5.08 (t, *J* 4.8 Hz, 1H), 4.79 (d, *J* 15.9 Hz, 1H), 4.33 (d, *J* 15.8 Hz), 4.34 – 4.21(m), (3H), 3.34 (d, *J* 4.7 Hz, 2H), 1.91 – 1.63 (m, 4H), 1.4 – 1.68 (m, 1H), 1.53 – 1.47 (m, 1H), 1.40 – 1.35 (m, 1H), 1.31 (t, *J* 7.1 Hz, 3H), 1.31 – 1.21 (m, 1H) 0.94 (qt, *J* 13.3, 3.9 Hz, 1H), 0.65 – 0.56 (m, 1H); ^13^C NMR (CDCl_3_): δ 170.0, 166.7, 152.9, 137.9, 135.4, 130.9, 128.7, 128.5, 127.4, 127.0, 126.6, 64.5, 63.7, 59.8, 45.9, 39.5, 34.4, 32.8, 24.1, 22.2, 22.1, 14.2; HRMS *m/z* [M + H]^+^ calculated for C_26_H_30_N_2_O_4_: 435.2284. Found: 435.2294; $\alpha_{D}^{20}$ = 17.2 (c 0.1, CH_2_Cl_2_).

**(3*S*)-1,3-Dibenzyl-4-allyl-1,4-diazaspiro[5.5]undecane-2,5-dione (33S)**

Compound **17S** (165 mg, 0.45mmol), BEMP (0.26 ml, 0.89 mmol) and allyl bromide (0.12 ml, 1.42 mmol) dissolved in dry DMF (4.5 ml). The reaction was heated in the micro wave reactor for 45 min at 150 °C. The solvent was removed under reduced pressure, co-evaporated with ethyl acetate. The crude product was purified with automated flash column chromatography (10% ethyl acetate in pentane) provided **33S** as an colourless oil (133 mg, 73%). ^1^H NMR (CDCl_3_): δ 7.33 – 7.08 (m, 10H), 5.79 (dddd, *J* 17.1, 10.2, 7.4, 4.5 Hz, 1H), 5.26 (dq, *J* 10.2, 1.3 Hz, 1H), 5.18 (dq, *J* 17.1, 1.2 Hz, 1H), 4.97 (d, *J* 16.0 Hz, 1H), 4.77 (ddt, *J* 15.2, 4.5, 1.7 Hz, 1H), 4.43 (t, *J* 4.7 Hz, 1H), 4.23 (d, *J* 16.0 Hz, 1H), 3.38 (ddt, *J* 15.2, 7.4, 1.2 Hz, 1H), 3.33 – 3.18 (m, 2H), 2.33 – 2.15 (m, 1H), 1.80 – 1.43 (m, 5H), 1.37 – 1.17 (m, 2H), 1.01 – 0.89 (m, 1H), 0.49 – 0.33 (m, 1H); ^13^C NMR (CDCl_3_): δ 169.2, 167.3, 138.2, 136.2, 131.9, 130.2, 128.9, 128.5, 127.4, 126.9, 126.4, 118.6, 62.7, 60.0, 46.7, 45.9, 37.8, 34.2, 34.1, 24.4, 23.2, 22.3; HRMS *m/z* [M + H]^+^ calculated for C_26_H_30_N_2_O_2_: 403.2385. Found: 403.2391; $\alpha_{D}^{20}$ = -60.2 (c 0.1, CH_2_Cl_2_).

**(3*S*)-1,4-Dibenzyl-3-(methoxycarbonyl-methyl)-1,4-diazaspiro[5.5]undecane-2,5-dione (34S)**

A solution of **21S** (63 mg, 0.18 mmol), BEMP (0.11 ml, 0.38 mmol), benzylbromide (0.043 ml, 0.36 mmol) and KI (61 mg, 0.37 mmol) was stirred at r.t. for 19 h. The reaction mixture was diluted with ethyl acetate (50 ml) and washed with distilled water (5 x 100 ml), brine, dried over Na_2_SO_4_ and filtered. The solvent was removed under reduced pressure and the crude product was purified by automatic flash chromatography (15-20% ethyl acetate in pentane) providing **34S** as an colourless oil (52 mg, 60%). ^1^H NMR (CDCl_3_): δ 7.38 – 7.18 (m, 10H), 5.12 (d, *J* 15.3 Hz, 1H), 4.86 (d, *J* 16.2 Hz, 1H), 4.65 (d, *J* 16.2 Hz, 1H), 4.44 (dd, *J* 5.7, 4.5 Hz, 1H), 4.28 (d, *J* 15.2 Hz, 1H), 3.64 (s, 3H), 3.02 – 2.87 (m, 2H), 2.27 – 2.11 (m, 1H), 2.09 – 1.87 (m, 3H), 1.84 – 1.59 (m, 5H), 1.19 – 1.05 (m, 1H); ^13^C NMR (CDCl_3_): δ 170.5, 169.6, 167.6, 138.2, 136.3, 129.0, 128.6, 127.9, 127.8, 127.0, 126.5, 62.9, 55.8, 52.2, 47.7, 45.9, 37.3, 34.7, 33.9, 24.5, 23.0, 22.7; HRMS *m/z* [M + H]^+^ calculated for C_26_H_30_N_2_O_4_: 435.2284. Found: 435.2287; $\alpha_{D}^{20}$ = 2.3 (c 0.1, CH_2_Cl_2_).

**(3*R,*6*R*)-1,3-dibenzyl-4-(ethoxycarbonyl-methyl)-6-phenylpiperazine-2,5-dione (35RR)**

Compound **35RR** was synthesised following procedure D from **22RR** (377 mg, 1.02 mmol), BEMP (0.59 ml, 2.04 mmol), bromoacetate (0.17 ml, 1.53 mmol) in dry DCM (26 ml). The reaction was stirred at r.t. overnight. Purification by automated flash column chromatography (25% ethyl acetate in pentane) provided **35RR** as a colourless oil (434 mg, 93%). ^1^H NMR (CDCl_3_): δ 7.37 – 7.13 (m, 11H), 7.10 – 7.06 (m, 2H), 6.77 – 6.67 (m, 2H), 5.27 (d, *J* 14.7 Hz, 1H), 4.87 (d, *J* 17.3 Hz, 1H), 4.74 – 4.71 (m, 1H), 4.25 (s, 1H), 4.13 (q, *J* 7.1 Hz, 2H), 3.71 (d, *J* 17.3 Hz, 1H), 3.52 (dd, *J* 14.5, 3.7 Hz, 1H), 3.37 (d, *J* 14.7 Hz, 1H), 3.25 (dd, *J* 14.4, 4.3 Hz, 1H), 1.20 (t, *J* 7.2 Hz, 3H); ^13^C NMR (CDCl_3_): δ 167.8, 166.0, 164.8, 136.6, 134.65, 134.63, 129.9, 128.94, 128.85, 128.83, 128.82, 128.5, 128.2, 127.7, 127.4, 62.1, 61.7, 60.9, 46.9, 45.4, 37.0, 14.1; HRMS *m/z* [M+H]^+^ calculated for C_28_H_28_N_2_O_4_: 457.2127. Found: 457.2144; $\alpha_{D}^{20}$ = 18.9 (c 0.1, CH_2_Cl_2_).

**(3*R*,6*S*)-1,3-Dibenzyl-4-(ethoxycarbonyl-methyl)-6-phenylpiperazine-2,5-dione (35RS)**

Compound **35RS** was synthesised following procedure D from **22RS** (204 mg, 0.551 mmol), BEMP (320 µl, 1.11 mmol), ethyl bromoacetate (100 µl, 0.902 mmol). The reaction was stirred at r.t. for 24 h. Purification by automated flash column chromatography (20% ethyl acetate in pentane) provided **35RS** as a colourless oil (202 mg, 80%). ^1^H NMR (CDCl_3_): δ 7.37 – 7.24 (m, 9H), 7.23 – 7.19 (m, 2H), 7.14 – 7.10 (m, 2H), 7.05 – 7.00 (m, 2H), 5.65 (d, *J* 14.8 Hz, 1H), 5.05 (s, 1H), 4.41 (dd, *J* 8.7, 3.9 Hz, 1H), 4.28 (d, *J* 17.4 Hz, 1H), 4.19 – 4.06 (m, 2H), 3.64 (d, *J* 14.9 Hz, 1H), 3.31 (dd, *J* 13.8, 3.9 Hz, 1H), 3.00 (dd, *J* 13.8, 3.9 Hz), 2.97 (d, *J* 17.4 Hz) (2H), 1.19 (t, *J* 7.1 Hz, 3H); ^13^C NMR (CDCl_3_): δ 167.8 , 166.3 , 165.7 , 136.1 ,135.1, 134.8 , 129.6 , 129.14, 129.12, 129.0, 128.6 , 128.2 , 128.1 , 127.6 , 126.5 , 63.8 , 62.2 , 61.6 , 47.9 , 47.5 , 39.6 , 14.1; HRMS *m/z* [M+H]^+^ calculated for C_28_H_28_N_2_O_4_: 457.2127. Found: 457.2127; $\alpha_{D}^{20}$ = 70.5 (c 0.1, DCM).

**(3*R*,6*R*)-3-Benzyl-1-(4-chlorobenzyl)-4-(ethoxycarbonyl-methyl)-6-phenylpiperazine-2,5-dione (36RR)**

Compound **36RR** was synthesised following procedure D from **23RR** (50.4 mg, 0.125 mmol), BEMP (71.0 µl, 0.245 mmol) and ethyl bromoacetate (21.0 µl, 0.189 mmol) in dry DCM (3.2 ml). The reaction was stirred at r.t. for 23 h. Purification by automated flash column chromatography (20-25% ethyl acetate in pentane) provided **36RR** as a colourless oil (32.0 mg, 52%). ^1^H NMR (CDCl_3_): δ 7.35 – 7.29 (m, 4H), 7.27 – 7.19 (m, 2H), 7.16 – 7.06 (m, 6H), 6.62 – 6.57 (m, 2H), 5.10 (d, *J* 14.8 Hz, 1H), 4.90 (d, *J* 17.3 Hz, 1H), 4.70 (t, *J* 3.9 Hz, 1H), 4.17 – 4.11 (m, 3H), 3.72 (d, *J* 17.3 Hz, 1H), 3.48 (dd, *J* 14.4, 3.8 Hz, 1H), 3.39 (d, *J* 14.8 Hz, 1H), 3.24 (dd, *J* 14.4, 4.2 Hz, 1H), 1.21 (t, *J* 7.1 Hz, 3H); ^13^C NMR (CDCl_3_): δ 167.8, 166.0, 165.0, 136.4, 134.6, 133.6, 133.4, 130.2, 130.1, 129.1, 129.03, 128.97, 128.7, 128.3, 127.6, 62.4, 61.8, 61.1, 46.4, 45.5, 37.1, 14.2; HRMS *m/z* [M+H]^+^ calculated for C_28_H_27_ClN_2_O_4_: 491.1737. Found: 491.1743; $\alpha_{D}^{20}$ = 44.9 (c 0.1, DCM).

**(3*R,*6*S*)-3-Benzyl-1-(4-chlorobenzyl)-4-(*tert*-butoxy-carbonyl-methyl)-6-phenylpiperazine-2,5-dione (37RS)**

Compound **37RS** was synthesised following procedure D from **23RS** (211 mg, 0.521 mmol), BEMP (300 µl, 1.04 mmol) and *t*-butyl- bromoacetate (150 µl, 1.02 mmol). The reaction was stirred at r.t. overnight. Purification by flash column chromatography (10-20% ethyl acetate in pentane) provided **37RS** as a colourless oil (259 mg, 96%). ^1^H NMR (CDCl_3_): δ 7.34 – 7.23 (m, 8H), 7.16 – 7.13 (m, 2H), 7.04 – 7.00 (m, 4H), 5.49 (d, *J* 14.9 Hz, 1H), 4.99 (s, 1H), 4.40 (dd, *J* 8.1, 4.1 Hz, 1H), 4.26 (d, *J* 17.3 Hz, 1H), 3.63 (d, *J* 14.9 Hz, 1H), 3.27 (dd, *J* 13.9, 4.1 Hz, 1H), 3.03 (dd, *J* 13.9, 8.1 Hz, 1H), 2.92 (d, *J* 17.2 Hz, 1H), 1.39 (s, 9H); ^13^C NMR (CDCl_3_): δ 166.7, 166.2, 165.3, 136.0, 134.7, 133.9, 133.7, 129.7, 129.6, 129.10, 129.07, 129.0, 128.6, 127.5, 126.6, 82.5, 63.4, 62.5, 48.0, 47.3, 39.3, 28.0; HRMS *m/z* [M+H]^+^ calculated for C_30_H_31_ClN_2_O_4_: 519.2050. Found: 519.2043; $\alpha_{D}^{20}$ = 54.8 (c 0.1, CH_2_Cl_2_).

**(3*R*,6*R*)-1,3-Dibenzyl-4-(3-ethyloxy-carbonyl-prop-2-enyl)-6-phenylpiperazine-2,5-dione (38RR)**

Compound **38RR** was synthesised following procedure D from **22RR** (67.0 mg, 0.181 mmol), BEMP (57.0 µl, 0.20 mmol) and ethyl-4-bromocrotonate (50.0 µl, 0.36 mmol). The reaction was stirred at r.t. for 3 days. Purification by automated flash chromatography (10-50% ethyl acetate in pentane) provided **38RR** (35 mg, 40%) as a colourless oil. ^1^H NMR (CDCl_3_): δ 7.38 – 7.11 (m, 9H), 7.09 – 7.00 (m, 4H), 6.79 – 6.71 (m, 3H), 5.91 (ddd, *J* 15.7, 2.0, 1.3 Hz, 1H), 5.20 (d, *J* 14.5 Hz, 1H), 4.94 (ddd, *J* 16.5, 4.5, 2.0 Hz, 1H), 4.55 (dd, *J* 4.4, 3.5 Hz, 1H), 4.19 (q, *J* 7.1 Hz, 2H), 4.07 (s, 1H), 3.68 (ddd, *J* 16.5, 7.2, 0.8 Hz, 1H), 3.42 (dd, *J* 14.3, 3.6 Hz, 1H). 3.41 (d, *J* 14.5 Hz), 3.25 (dd, *J* 14.3, 4.4 Hz) (2H)*.*1.29 (t, *J* 7.14 Hz, 3H); ^13^C NMR (CDCl_3_): δ 165.9, 165.4, 165.3, 140.6, 136.65, 134.69, 134.3, 130.1, 129.2, 129.00, 128.95, 128.6, 127.90, 127.87, 127.6, 124.4, 62.4, 60.8, 60.1, 47.1, 44.3, 37.1, 14.3, HRMS *m/z* [M+H]^+^ calculated for C_30_H_30_N_2_O_4_: 483.2284. Found: 483.2301; $\alpha_{D}^{20}$ = 44.8 (c 0.1, CH_2_Cl_2_).

**(*3R,6S*)-1,3-Dibenzyl-4-(3-ethyloxy-carbonyl-prop-2-enyl)-6-phenylpiperazine-2,5-dione (38RS)**

Compound **38RR** was synthesised following procedure D from **22RS** (84 mg, 0.23 mmol), BEMP (130 µl, 0.449 mmol) and ethyl-4-bromocrotonate (47.0 µl, 0.341 mmol). The reaction was stirred at r.t. for 2 days. Purification by automated flash column chromatography (10-20% ethyl acetate in pentane) provided **38RS** as an colourless oil (79 mg, 73%). ^1^H NMR (CDCl_3_): δ 7.37 – 7.23 (m, 9H), 7.20 – 7.13 (m, 4H), 7.03 – 6.99 (m, 2H), 6.59 (ddd, *J* 15.7, 6.6, 4.4 Hz, 1H), 5.64 – 5.56 (m, 2H), 5.03 (s, 1H), 4.49 (ddd, *J* 16.8, 4.5, 2.0 Hz, 1H), 4.27 (dd, *J* 8.7, 4.1 Hz, 1H), 4.20 – 4.14 (m, 2H), 3.60 (d, *J* 14.7 Hz, 1H), 3.29 (dd, *J* 13.9, 4.1 Hz, 1H), 2.95 – 2.89 (m, 2H), 1.28 (t, *J* 7.1 Hz, 3H); ^13^C NMR (CDCl_3_): δ 166.7, 165.5, 165.2, 140.6, 136.2, 135.2, 134.6, 129.6, 129.22, 129.17, 129.12, 128.7, 128.6, 128.3, 127.6, 126.6, 123.7, 62.3, 62.2, 60.7, 48.1, 46.0, 40.0, 14.4; HRMS *m/z* [M+H]^+^ calculated for C_30_H_30_N_2_O_4_: 483.2284. Found: 483.2295; $\alpha_{D}^{20}$ = 75.6 (c 0.1, CH_2_Cl_2_).

**(3*R*,6*R*)-4-Allyl-1,3-dibenzyl-6-phenylpiperazine-2,5-dione (39RR)**

Compound **39RR** was synthesised following procedure D from **22RR** (248 mg, 0.670 mmol), BEMP (390 µl 1.35 mmol) and allyl bromide (100 µl, 1.18 mmol). The reaction was stirred at r.t. for 22 h. Purification by flash column chromatography (10-50% ethyl acetate in pentane) provided **39RR** as a colourless oil (263 mg, 96%). ^1^H NMR (CDCl_3_): δ 7.34 – 7.06 (m, 9H), 7.09 – 6.98 (m, 4H), 6.69 – 6.63 (m, 2H), 5.68 (dddd, *J* 17.2, 10.1, 8.0, 4.5 Hz, 1H), 5.28 – 5.21 (m, 2H), 5.15 (d, *J* 14.6 Hz, 1H), 4.83 (ddt, *J* 15.1, 4.6, 1.7 Hz, 1H), 4.58 (dd, *J* 4.4, 3.4 Hz, 1H), 4.03 (s, 1H), 3.50 (ddd, *J* 15.2, 8.0, 1H), 3.38 (dd, *J* 3.5, 14.2 Hz 3.34 (d, *J* 14.2 Hz) (2H), 3.24 (dd, *J* 14.2, 4.4 Hz, 1H); ^13^C NMR (CDCl_3_): δ 165.6, 165.5, 137.1, 134.7, 134.6, 131.4, 130.1, 129.1, 129.0, 128.8, 128.8, 128.5, 127.9, 127.7, 127.4, 119.7, 62.3, 59.3, 46.9, 46.1, 36.6; HRMS *m/z* [M+H]^+^ calculated for C_27_H_26_N_2_O_3_: 411.2072. Found: 411.2079; $\alpha_{D}^{20}$ = 73.8 (c 0.1, CH_2_Cl_2_).

**(3R,6S)-4-Allyl-1,3-dibenzyl-6-phenylpiperazine-2,5-dione (39RS)**

Compound **39RS** was synthesised following procedure D from **22RS** (242 mg, 0.653 mmol), BEMP (380 µl, 1.31 mmol) and allyl bromide (90.0 µl, 1.06 mmol). The reaction was stirred at r.t. for 20 h. Purification by flash column chromatography (10-50% ethyl acetate in pentane) provided **39RS** as a colourless oil (249 mg, 93%). ^1^H NMR (CDCl_3_): δ 7.36 – 7.22 (m, 9H), 7.17 – 7.13 (m, 2H), 7.09 – 7.05 (m, 4H), 5.64 – 5.53 (m), 5.57 (d, *J* 14.7 Hz) (2H), 5.15 (ddt, *J* 10.1, 1.4, 1.2 Hz, 1H), 5.00 (s, 1H), 4.94 (ddt, *J* 17.2, 1.8, 1.2 Hz, 1H), 4.53 (ddt, *J* 15.4, 4.5, 1.7 Hz, 1H), 4.37 (dd, *J* 7.8, 4.4 Hz, 1H), 3.56 (d, *J* 14.6 Hz, 1H), 3.27 (dd, *J* 13.9, 4.5 Hz, 1H), 2.99 (dd, *J* 13.9, 7.8 Hz, 1H), 2.84 (dd, *J* 15.3, 7.7 Hz, 1H); ^13^C NMR (CDCl_3_): δ 166.6, 164.9, 136.3, 135.3, 134.9, 131.3, 129.7, 129.1, 129.00, 128.97, 128.6, 128.5, 128.2, 127.4, 126.7, 119.1, 62.4, 61.0, 47.8, 47.4, 39.4; HRMS *m/z* [M+H]^+^ calculated for C_27_H_26_N_2_O_3_: 411.2072. Found: 411.2063; $\alpha_{D}^{20}$ = 107.1 (c 0.1, CH_2_Cl_2_).

**(3*R*,6*R*)-3-Benzyl-1,6-diphenyl-4-(*tert*-butoxy-carbonyl-methyl)-piperazine-2,5-dione (40RR)**

Compound **40RR** was synthesised following procedure D from **24RR** (50 mg, 0.14 mmol), BEMP (0.081 ml, 0.28 mmol) and *tert*-butyl bromoacetate (0.041 ml, 0.28 mmol). The reaction was stirred at r.t. for 17 h. Purification by automated flash column chromatography (10-20% ethyl acetate in pentane) provided **40RR** as white solids (62 mg, 94%). ^1^H NMR (CDCl_3_): δ 7.48 – 7.37 (m, 3H), 7.26 – 7.19 (m, 2H), 7.19 – 7.04 (m, 6H), 7.02 – 6.94 (m, 2H), 6.66 – 6.59 (m, 2H), 4.95 (d, *J* 17.1 Hz, 1H), 4.71 (t, *J* 3.4, 1H), 4.21 (s, 1H), 3.73 (d, *J* 17.1 Hz, 1H), 3.46 (dd, *J* 14.1, 3.5 Hz, 1H), 3.25 (dd, *J* 14.0, 4.1 Hz, 1H), 1.50 (s, 9H); ^13^C NMR (CDCl_3_): δ 167.0, 166.3, 165.0, 138.5, 136.9, 134.9, 130.5, 129.0, 128.8, 128.7, 128.4, 128.3, 127.9, 127.5, 82.8, 65.62 61.9, 46.2, 37.4, 28.2; HRMS *m/z* [M + H]^+^ calculated for C_29_H_30_N_2_O_4_: 471.2284. Found: 471.2245; $\alpha_{D}^{20}$ = 67.1 (c 0.1, CH_2_Cl_2_).

**(3*R*,6*S*)-3-Benzyl-1,6-diphenyl-4-(*tert*-butoxy-carbonyl-methyl)-piperazine-2,5-dione (40RS)**

Compound **40RS** was synthesised following procedure D from **24RS** (50 mg, 0.14 mmol), BEMP (0.081 ml, 0.28 mmol) and *tert*-butyl bromoacetate (0.041 ml, 0.28 mmol). The reaction was stirred at r.t. for 17 h. Purification by automated flash column chromatography (10-20% ethyl acetate in pentane) provided **40RS** as a colourless oil (62 mg, 94%). ^1^H NMR (CDCl_3_): δ 7.36 – 7.20 (m, 11H), 7.17 – 7.12 (m, 2H), 7.08 – 7.04 (m, 2H), 5.58 (s, 1H), 4.45 (dd, *J* 8.6, 4.1 Hz, 1H), 4.32 (d, *J* 17.2 Hz, 1H), 3.26 (dd, *J* 13.9, 4.1 Hz, 1H), 2.95 (dd, *J* 13.8, 8.6 Hz, 1H), 2.92 (d, *J* 17.3 Hz, 1H), 1.44 (s, 9H); ^13^C NMR (CDCl_3_): δ 166.9, 166.3, 165.9, 140.0, 136.3, 136.0, 129.7, 129.3, 129.2, 129.0, 128.6, 127.6, 127.4, 126.8, 125.8, 82.6, 66.8, 64.3, 48.2, 39.2, 28.1; HRMS *m/z* [M + H]^+^ calculated for C_29_H_30_N_2_O_4_: 471.2284. Found: 471.2296; $\alpha_{D}^{20}$ = 20.1 (c 0.1, CH_2_Cl_2_).

**(3*R*,6*R*)-3-Benzyl-6-(4-chlorophenyl)-1-phenyl-4-(*tert*-butoxy-carbonyl-methyl)-piperazine-2,5-dione (41RR)**

Compound **41RR** was synthesised following procedure D from **25RR** (50 mg, 0.13 mmol) BEMP (0.074 ml, 0.26 mmol) and *tert*-butyl bromoacetate (0.04 ml, 0.27 mmol). The reaction was stirred at r.t. for 20 h. Purification by automated flash column chromatography (10-20% ethyl acetate in pentane) provided **41RR** as white solids (39 mg, 60%). ^1^H NMR (CDCl_3_): δ 7.48 – 7.37 (m, 3H), 7.23 – 7.17 (m, 2H), 7.15 – 7.08 (m, 5H), 7.00 – 6.94 (m, 2H), 6.57 – 6.50 (m, 2H), 4.93 (d, *J* 17.1 Hz, 1H), 4.69 (t, *J* 3.8 Hz, 1H), 4.13 (s, 1H), 3.73 (d, *J* 17.1 Hz, 1H), 3.43 (dd, *J* 14.0, 3.5 Hz, 1H), 3.24 (dd, *J* 14.0, 4.0 Hz, 1H), 1.49 (s, 9H); ^13^C NMR (CDCl_3_): δ 166.8, 165.9, 164.9, 136.9, 136.5, 134.7, 133.1, 130.4, 128.93, 128.92, 128.7, 128.5, 128.5, 128.4, 127.9, 82.8, 65.4, 61.8, 46.2, 37.4, 28.1; HRMS *m/z* [M + H]^+^ calculated for C_29_H_29_ClN_2_O_4_: 505.1894. Found: 505.1910; $\alpha_{D}^{20}$ = 84.7 (c 0.1, CH_2_Cl_2_).

**(3*R*,6*S*)-3-Benzyl-6-(4-chlorophenyl)-1-phenyl-4-(*tert*-butoxy-carbonyl-methyl)-piperazine-2,5-dione (41RS)**

Compound **41RS** was synthesised following procedure D from **25RS** (52 mg, 0.13 mmol), BEMP (0.077 ml, 0.26 mmol) and *tert*-butyl bromoacetate (0.04 ml, 0.27 mmol). The reaction was stirred at r.t. for 20 h. Purification by automated flash column chromatography (10-20% ethyl acetate in pentane) provided **41RS** as a colourless oil (36 mg, 54%). ^1^H NMR (CDCl_3_): δ 7.35 – 7.24 (m, 8H), 7.21 – 7.15 (m, 2H), 7.08 – 7.01 (m, 4H), 5.51 (s, 1H), 4.45 (dd, *J* 8.3, 4.1 Hz, 1H), 4.34 (d, *J* 17.2 Hz, 1H), 3.24 (dd, *J* 13.9, 4.2 Hz, 1H), 2.97 (dd, 14.0, 8.4 Hz, 1H), 2.96 (d, *J* 17.2 Hz, 1H), 1.43 (s, 9H); ^13^C NMR (CDCl_3_): δ 166.8, 166.1, 165.5, 138.3, 135.9, 135.5, 132.9, 129.6, 129.3, 129.1, 128.9, 128.6, 127.6, 127.0, 126.7, 82.6, 66.5, 63.9, 47.9, 38.9, 28.0; HRMS *m/z* [M + H]^+^ calculated for C_29_H_29_ClN_2_O_4_: 505.1894. Found: 505.1884.

**(3*R*,6*R*)-3-Benzyl-6-(3-chlorophenyl)-1-phenyl-4-(*tert*-butoxy-carbonyl-methyl)-piperazine-2,5-dione (42RR)**

Compound **42RR** was synthesised following procedure D from **26RR** (35 mg, 0.09 mmol), BEMP (0.052 ml, 0.18 mmol) and *tert*-butyl bromoacetate (0.027 ml, 0.18 mmol). The reaction was stirred at r.t. for 17 h. Purification by automated flash column chromatography (10-20% ethyl acetate in pentane) provided **42RR** as a colourless oil (39 mg, 86%). ^1^H NMR (CDCl_3_): δ 7.48 – 7.38 (m, 3H), 7.23 – 7.05 (m, 7H), 6.93 – 6.86 (m, 2H), 6.63 – 6.58 (m, 2H), 4.93 (d, *J* 17.1 Hz, 1H), 4.67 (dd, *J* 4.2, 3.7, 1H), 4.13 (s, 1H), 3.71 (d, *J* 17.1 Hz, 1H), 3.45 (dd, *J* 14.0, 3.4 Hz, 1H), 3.22 (dd, *J* 14.0, 4.1 Hz, 1H), 1.50 (s, 9H); ^13^C NMR (CDCl_3_): δ 166.9, 165.9, 165.0, 138.3, 135.6, 134.8, 134.2, 130.6, 130.1, 129.1, 129.0, 128.7, 128.0, 127.7, 127.4, 83.0, 64.8, 62.0, 46.4, 37.6, 28.2; HRMS *m/z* [M + H]^+^ calculated for C_29_H_29_ClN_2_O_4_: 505.1894. Found: 505.1880; $\alpha_{D}^{20}$ = 94.8 (c 0.1, CH_2_Cl_2_).

**(3*R*,6*S*)-3-Benzyl-6-(4-chlorophenyl)-1-phenyl-4-(*tert*-butoxy-carbonyl-methyl)-piperazine-2,5-dione (42RS)**

Compound **42RS** was synthesised following procedure D from **26RS** (62 mg, 0.16 mmol), BEMP (0.09 ml, 0.32 mmol) and *tert*-butyl bromoacetate (0.047 ml, 0.32 mmol). The reaction was stirred at r.t. for 17 h. Purification by automated flash column chromatography (10-20% ethyl acetate in pentane) provided **42RS** as a colourless oil (56 mg, 70%). Diastereomeric ratio of 3:33:1, determined from ^1^H NMR spectra. ^1^H NMR (CDCl_3_): δ 7.41 – 7.28 (m, 6H), 7.20 – 7.10 (m, 6H), 6.79 – 6.74 (m, 2H), 5.47 (s, 1H), 4.53 (dd, *J* 6.9, 4.2 Hz, 1H), 4.43 (d, *J* 17.1 Hz, 1H), 3.27 (dd, *J* 14.1, 4.3 Hz, 1H), 3.24 – 3.14 (m, 2H), 1.46 (s, 9H); ^13^C NMR (CDCl_3_): δ 166.7, 165.4, 165.2, 139.4, 135.7, 134.4, 130.0, 129.3, 129.2, 128.87, 128.86, 128.5, 127.7, 127.6, 126.1, 82.7, 66.0, 63.4, 47.6, 38.5, 28.0; HRMS *m/z* [M + H]^+^ calculated for C_29_H_29_ClN_2_O_4_: 505.1894. Found: 505.1873.

**(3*R*,6*R*)-3-Benzyl-6-(3-chlorophenyl)-1-phenyl-4-(*tert*-butoxy-carbonyl-methyl)-piperazine-2,5-dione (43RR)**

Compound **43RR** was synthesised following procedure D from **29RR** (54 mg, 0.14 mmol), BEMP (0.08 ml, 0.28 mmol) and *tert*-butyl bromoacetate (0.041 ml, 0.28 mmol). The reaction was stirred at r.t. for 17 h. Purification by automated flash column chromatography (10-20% ethyl acetate in pentane) provided **43RR** as a yellow oil (65 mg, 93%). Diastereomeric ratio of 5:1, determined from ^1^H NMR spectra. ^1^H NMR (CDCl_3_): δ 7.50 – 7.37 (m, 3H), 7.26 – 7.02 (m, 7H), 6.96 (t, *J* 1.7 Hz, 1H), 6.88 (dt, *J* 7.0, 1.7 Hz, 1H), 6.66 – 6.56 (m, 2H), 4.92 (d, *J* 17.0 Hz, 1H), 4.69 (t, *J* 3.7 Hz, 1H), 4.12 (s, 1H), 3.72 (d, *J* 17.0 Hz, 1H), 3.46 (dd, *J* 14.0, 3.4 Hz, 1H), 3.23 (dd, *J* 14.0, 4.1 Hz, 1H), 1.51 (s, 9H); ^13^C NMR (CDCl_3_): δ 166.7, 165.6, 164.9, 138.7, 138.1, 134.6, 134.1, 130.5, 129.5, 129.0, 128.89, 128.86, 128.4, 127.9, 127.6, 127.3, 126.8, 82.9, 64.8, 61.9, 46.3, 37.4, 28.1; HRMS *m/z* [M + H]^+^ calculated for C_29_H_29_ClN_2_O_4_: 505.1894. Found: 505.1892.

**(3*R*,6*S*)-3-Benzyl-6-(3-chlorophenyl)-1-phenyl-4-(*tert*-butoxy-carbonyl-methyl)-piperazine-2,5-dione (43RS)**

Compound **43RS** was synthesised following procedure D from **27RS** (72 mg, 0.18 mmol), BEMP (0.11 ml, 0.38 mmol) and *tert*-butyl bromoacetate (0.054 ml, 0.37 mmol). The reaction was stirred at r.t. for 18 h. Purification by automated flash column chromatography (10-20% ethyl acetate in pentane) provided **43RS** as a yellow oil (63 mg, 68%). Diastereomeric ratio of 2.8:1, determined from ^1^H NMR spectra. ^1^H NMR (CDCl_3_): δ 7.39 – 7.29 (m, 5H), 7.27 – 7.03 (m, 8H), 6.85 – 6.82 (m, 1H), 5.54 (s, 1H), 4.48 (dd, *J* 7.9, 4.2 Hz, 1H), 4.36 (d, *J* 17.2 Hz, 1H), 3.28 (dd, *J* 13.9, 4.3 Hz, 1H), 3.04 (dd, 14.0, 8.0, 1H) 3.06 (d, *J* 17.2 Hz, 1H), 1.45 (s, 9H); ^13^C NMR (CDCl_3_): δ 166.8, 165.9, 165.2, 139.6, 138.0, 135.9, 134.9, 130.1, 129.7, 129.4, 129.2, 128.9, 127.9, 127.6, 127.4, 125.9, 124.9, 82.7, 66.2, 63.9, 48.1, 39.1, 28.1; HRMS *m/z* [M + H]^+^ calculated for C_29_H_29_ClN_2_O_4_: 505.1894. Found: 505.1891.

**3-Benzyl-1,6-(4-chlorophenyl)-piperazine-2,5-dione (28RR and 28RS)**

Compounds **28RR** and **28SS** were synthesised following procedure C from Boc-D-Phe-OH (250 mg, 0.94 mmol), 4-chloroaniline (120 mg, 0.94 mmol), 4-chlorobenzaldehyde (133 mg, 0.94 mmol) and 2-morpholinoethyl isocyanide (140 µl, 1.00 mmol) and purification by automatic flash column chromatography (50-80% ethyl acetate in pentane) provided **28RR** (17 mg, 4%) as an yellow solid and **28RS** (14 mg, 3.5%), as a yellow oil, in a total yield of 7.5%. Products was confirmed by LCMS analysis and used in the next step without further purification.

**(3*R*,6*R*)-3-Benzyl-1,6-(di-4-chlorophenyl)-4-(*tert*-butoxy-carbonyl-methyl)-piperazine-2,5-dione (44RR)**

Compound **44RR** was synthesised following procedure D from **28RR** (28 mg, 0.066 mmol), BEMP (0.038 ml, 0.13 mmol) and *tert*-butyl bromoacetate (0.019 ml, 0.13 mmol). The reaction was stirred at r.t. for 17 h. Purification by automated flash column chromatography (10-20% ethyl acetate in pentane) provided **44RR** as a yellow solid (31 mg, 87%). Diastereomeric ratio of 4.3:1, determined from ^1^H NMR spectra. ^1^H NMR (CDCl_3_): δ 7.47 – 7.35 (m, 3H), 7.20 – 7.07 (m, 6H), 6.93 – 6.82 (m, 2H), 6.59 – 6.46 (m, 2H), 4.91 (d, *J* 17.1 Hz, 1H), 4.66 (t, *J* 3.7 Hz, 1H), 4.06 (s, 1H), 3.71 (d, *J* 17.1 Hz, 1H), 3.43 (dd, *J* 14.0, 3.4 Hz, 1H), 3.26 – 3.15 (m, 1H), 1.50 (s, 9H); ^13^C NMR (CDCl_3_): δ 166.9, 165.7, 165.1, 136.8, 135.3, 134.7, 134.5, 133.5, 130.6, 130.0, 129.3, 129.1, 128.9, 128.7, 128.1, 83.1, 64.7, 62.1, 46.4, 37.6, 28.2; HRMS *m/z* [M + H]^+^ calculated for C_29_H_28_Cl_2_N_2_O_4_: 539.1504. Found: 539.1483.

**(3*R*,6*S*)-3-Benzyl-1,6-(di-4-chlorophenyl)-4-(*tert*-butoxy-carbonyl-methyl)-piperazine-2,5-dione (44RS)**

Compound **44RS** was synthesised following procedure D from **28RS** (24 mg, 0.056 mmol), BEMP (0.033 ml, 0.11 mmol) and *tert*-butyl bromoacetate (0.017 ml, 0.11 mmol). The reaction was stirred at r.t. for 17 h. Purification by automated flash column chromatography (10-20% ethyl acetate in pentane) provided **44RS** (11 mg, 36%) as a yellow solid. Diastereomeric ratio of 1.7:1, determined from ^1^H NMR spectra. ^1^H NMR (CDCl_3_): δ 7.43 – 7.33 (m, 3H), 7.20 – 7.02 (m, 8H), 6.70 – 6.64 (m, 2H), 5.41 (s, 1H), 4.52 (dd, *J* 6.7, 4.3 Hz, 1H), 4.44 (d, *J* 17.1 Hz, 1H), 3.28 – 3.16 (m, 2H), 3.23 (d, *J* 17.2 Hz, 1H), 1.46 (s, 9H); ^13^C NMR (CDCl_3_): δ 166.8, 165.5, 165.1, 137.9, 135.7, 134.7, 134.2, 133.5, 130.1, 129.6, 129.3, 129.1, 128.7, 128.6, 127.6, 83.0, 66.1, 63.3, 47.6, 38.5, 28.2; HRMS *m/z* [M + H]^+^ calculated for C_29_H_28_Cl_2_N_2_O_4_: 539.1504. Found: 539.1484.

**(3*S*,6*S*)-3-Methyl-4-(*tert*-butoxy-carbonyl-methyl)-1-(4-chlorophenyl)-6-(3-chlorophenyl) piperazine-2,5-dione (45SS)**

Compound **45SS** was synthesised following procedure D from **29SS** (25 mg, 0.07 mmol), BEMP (41 µl, 0.14 mmol) and *tert*-butyl bromoacetate (21 µl, 0.14 mmol). The reaction was stirred at r.t. for 20 h. Purification by automated flash column chromatography (10-25% ethyl acetate in pentane) provided **45SS** as a colourless oil (12 mg, 37%). The other diastereoisomer were isolated as a colourless oil (14 mg, 43%). ^1^H NMR (CDCl_3_): δ 7.44 – 7.38 (m, 1H), 7.36 – 7.27 (m, 5H), 7.14 (d, *J* 8.8 Hz, 2H), 5.46 (s, 1H), 4.57 (d, *J* 17.6 Hz, 1H), 4.37 (q, *J* 7.0 Hz, 1H), 3.83 (d, *J* 17.6 Hz, 1H), 1.60 (d, *J* 7.0 Hz, 3H), 1.38 (s, 9H); ^13^C NMR (CDCl_3_): δ 167.2, 166.2, 138.2, 136.6, 135.3, 133.1, 130.4, 129.5, 129.4, 129.1, 127.0, 126.9, 124.8, 82.6, 66.7, 54.6, 45.1, 27.9, 15.6.

**(3*S*,6*R*)-3-Methyl-4-(*tert*-butoxy-carbonyl-methyl)-1-(4-chlorophenyl)-6-(3-chlorophenyl) piperazine-2,5-dione (45SR)**

Compound **45SR** was synthesised following procedure D from **29SR** (21 mg, 0.06 mmol), BEMP (35 µl, 0.12 mmol) and *tert*-butyl bromoacetate (18 µl, 0.12 mmol). The reaction was stirred at r.t. for 20 h. Purification by automated flash column chromatography (10-25% ethyl acetate in pentane) provided **45SR** as a colourless oil (16 mg, 55%). The other diastereoisomer was isolated as a colourless oil (8 mg. 28%). ^1^H NMR (CDCl_3_): δ 7.50 – 7.46 (m, 1H), 7.36 – 7.28 (m, 5H), 7.23 – 7.18 (m, 2H), 5.47 (s, 1H), 4.25 (q, *J* 7.3 Hz, 1H), 4.20 (d, *J* 17.1 Hz, 1H), 3.78 (d, *J* 17.1 Hz, 1H), 1.53 (d, *J* 7.2 Hz, 3H), 1.43 (s, 9H); ^13^C NMR (CDCl_3_): δ 167.4, 166.7, 164.2, 137.9, 137.7, 135.2, 133.2, 130.3, 129.5, 129.1, 127.0, 126.9, 124.6, 82.8, 66.2, 58.1, 47.3, 27.9, 19.1.

**(3*R*,6*R*)-1,3-Dibenzyl-4-(carboxy-methyl)-6-phenylpiperazine-2,5-dione (46RR)**

Compound **46RR** was synthesised using general procedure E from **36RR** (144 mg, 0.315 mmol) and conc. HCl (12 M, 8 ml). The reaction was stirred at 70 °C overnight. The crude material was co-evaporated with chloroform to provide **46RR** as an off white solid (117 mg, 87%). ^1^H NMR (CDCl_3_): δ 8.67 (br s, 1H), 7.29 – 7.07 (m, 11H), 7.05 – 7.00 (m, 2H), 6.73 – 6.68 (m, 2H), 5.21 (d, *J* 14.6 Hz, 1H), 4.81 (d, *J* 17.2 Hz, 1H), 4.74 (s, 1H), 4.16 (s, 1H), 3.69 (d, *J* 17.4 Hz, 1H), 3.42 (d, *J* 13.4 Hz, 1H), 3.38 (d, *J* 14.6 Hz, 1H), 3.17 (d, *J* 13.4 Hz, 1H); ^13^C NMR (CDCl_3_): δ 171.0, 166.4, 165.5, 136.2, 134.5, 134.4, 130.0, 128.99, 128.95 (2 carbons), 128.94, 128.6, 128.5, 127.9, 127.5, 62.2, 61.1, 47.1, 45.5, 37.2; HRMS m/z [M+H]^+^ calculated for C_26_H_24_N_2_O_4_: 429.1814. Found: 429.1814; $\alpha_{D}^{20}$ = 5.0 (c 0.1, CH_2_Cl_2_).

**(*3R,6S*)-1,3-dibenzyl-4-(carboxy-methyl)-6-phenylpiperazine-2,5-dione (46RS)**

Compound **46RS** was synthesised using general procedure E from **36RS** (60.0 mg, 0.131 mmol) and conc. HCl (12 M, 5 ml). The reaction was stirred at 70 °C for 4.5 h and then left at r.t. overnight. The crude material was co-evaporated with chloroform to provide **46RS** as a colourless oil (45 mg, 80%). ^1^H NMR (CDCl_3_): δ 7.40 – 7.33 (m, 3H), 7.30 – 7.22 (m, 6H), 7.19 – 7.10 (m, 4H), 7.05 – 6.96 (m, 2H), 5.63 (d, *J* 15.0 Hz, 1H), 5.08 (s, 1H), 4.43 (dd, *J* 9.1, 3.7 Hz, 1H), 4.34 (d, *J* 17.6 Hz, 1H), 3.71 (d, *J* 15.0 Hz, 1H), 3.30 (dd, *J* 13.8, 3.7 Hz, 1H), 2.95 – 2.87 (m, 2H); ^13^C NMR (CDCl_3_): δ 171.9, 167.0, 166.2, 135.9, 134.8, 134.6, 129.6, 129.27, 129.26, 129.1, 128.8, 128.2, 128.0, 127.7, 126.5, 63.8, 62.4, 48.3, 47.4, 39.6; HRMS *m/z* [M+H]^+^ calculated for C_26_H_24_N_2_O_4_: 429.1814. Found: 429.1803;$\alpha_{D}^{20}$ = 91.4 (c 0.1, CH_2_Cl_2_).

**(*3R,6R*)-3-Benzyl-1-(4-chlorobenzyl)-4-(carboxy-methyl)-6-phenylpiperazine-2,5-dione (47RR)**

Compound **47RR** was synthesised using general procedure E from **37RR** (117 mg, 0.225 mmol) and conc. HCl (12 M, 6.2 ml). The reaction was stirred at 70 °C for 2 h. The crude material was co-evaporated with chloroform to provide **47RR** as an colourless oil (102 mg, 98%). ^1^H NMR (CDCl_3_): δ 10.15 (s, 1H), 7.42 – 6.91 (m, 12H), 6.65 – 6.63 (m, 2H), 5.03 (d, *J* 14.1 Hz, 1H), 4.87 – 4.70 (m, 2H), 4.07 (s, 1H), 3.78 – 3.63 (m, 1H), 3.50 – 3.31 (m, 2H), 3.22 – 3.11 (m, 1H); ^13^C NMR (CDCl_3_): δ 171.0, 166.2, 165.6, 135.9, 134.2, 133.6, 133.0, 130.2, 130.1, 129.03, 128.97, 128.90, 128.6, 128.4, 127.6, 62.3, 61.2, 46.6, 45.7, 37.2; HRMS *m/z* [M+H]^+^ calculated for C_26_H_23_ClN_2_O_4_: 463.1424. Found: 463.1376; $\alpha_{D}^{20}$ = 33.3 (c 0.1, CH_2_Cl_2_).

**(*3R,6S*)-3-Benzyl-1-(4-chlorobenzyl)-4-(carboxy-methyl)-6-phenylpiperazine-2,5-dione (47RS)**

Compound **47RS** was synthesised following general procedure E from **37RS** (260 mg, 0.501 mmol) and conc. HCl (12 M, 12 ml). The reaction was stirred at 70 °C for 2 h. The crude material was co-evaporated with chloroform to provide **47RS** as white solids (210 mg, 91 %). ^1^H NMR (CDCl_3_): δ 9.96 (br s, 1H), 7.38 – 6.95 (m, 14H), 5.50 (d, *J* 14.9 Hz, 1H), 5.08 (s, 1H), 4.47 – 4.26 (m, 2H), 3.74 (d, *J* 14.8 Hz, 1H), 3.30 – 3.24 (m, 1H), 2.97 – 2.88 (m, 2H); ^13^C NMR (CDCl_3_): δ 171.4, 167.1, 166.0, 135.6, 134.3, 133.9, 133.3, 129.6, 129.4, 129.3, 129.2, 129.1, 128.9, 127.7, 126.4, 63.6, 62.6, 47.9, 47.3, 39.3; HRMS *m/z* [M+H]^+^ calculated for C_26_H_23_ClN_2_O_4_: 463.1424. Found: 463.1413; $\alpha_{D}^{20}$ = 81.0 (c 0.1, CH_2_Cl_2_).

**(3*R*,6*R*)-3-Benzyl-1,6-(diphenyl)-4-(carboxy-methyl)-piperazine-2,5-dione (48RR)**

Compound **48RR** was synthesised following general procedure E from **40RR** (53 mg, 0.11 mmol) and conc. HCl (12 M, 3 ml). The reaction was stirred at r.t. for 22 h. Compound **61RR** was isolated as a white foam (46 mg, 99%). ^1^H NMR (CDCl_3_): δ 7.48 – 7.41 (m, 3H), 7.23 – 7.18 (m, 2H), 7.15 -7.07 (m, 6H), 7.01 – 6.93 (m, 2H), 6.61 (d, *J* 7.4 Hz, 2H), 6.42 (br s, 1H), 4.92 (d, *J* 16.4 Hz, 1H), 4.77 (br s, 1H), 4.18 (s, 1H), 3.81 (d, *J* 16.5 Hz, 1H), 3.43 (d, *J* 13.4 Hz, 1H), 3.17 (d, *J* 13.5 Hz, 1H); ^13^C NMR (CDCl_3_): δ 170.9, 166.6, 165.4, 138.1, 136.4, 134.5, 130.5, 129.0, 128.8, 128.7, 128.4, 128.4, 128.0, 127.6, 127.4, 65.6, 62.1, 45.9, 37.6; HRMS *m/z* [M + H]^+^ calculated for C_25_H_22_N_2_O_4_: 415.1658. Found: 415.1619; $\alpha_{D}^{20}$ = 60.8 (c 0.1, CH_2_Cl_2_).

**(3*R*,6*S*)-3-Benzyl-1,6-(diphenyl)-4-(carboxy-methyl)-piperazine-2,5-dione (48RS)**

Compound **48RS** was synthesised following general procedure E from **40RS** (55 mg, 0.12 mmol) and conc. HCl (12 M, 3 ml). The reaction was stirred at r.t. for 22 h. Compound **48RS** was isolated as a colourless oil (40 mg, 83%). ^1^H NMR (CDCl_3_): δ 7.35 – 7.29 (m, 5H), 7.27 – 7.18 (m, 5H), 7.11 – 6.99 (m, 5H), 5.63 (s, 1H), 4.58 – 4.54 (m, 1H), 4.46 (dd, *J* 17.6, 1.5 Hz, 1H), 3.27 (dd, *J* 13.9, 4.1 Hz, 1H), 3.16 – 3.07 (m, 1H), 3.07 – 2.99 (m, 1H); ^13^C NMR (CDCl_3_): δ 171.0, 166.4, 139.5, 135.7, 135.4, 129.8, 129.2, 129.2, 128.8, 128.6, 127.7, 127.6, 126.9, 126.1, 66.6, 63.7, 47.0, 38.8. The 2,5-DKPs carbonyls have the same chemical shift, confirmed by HMBC; HRMS *m/z* [M + H]^+^ calculated for C_25_H_22_N_2_O_4_: 415.1658. Found: 415.1616; $\alpha_{D}^{20}$ = 17.6 (c 0.1, CH_2_Cl_2_).

**(3*R*,6*R*)-3-Benzyl-6-(4-chlorophenyl)-1-phenyl-4-(carboxy-methyl)-piperazine-2,5-dione (49RR)**

Compound **49RR** was synthesised following general procedure E from **41RR** (32 mg, 0.063 mmol) and conc. HCl (12 M, 6 ml). The reaction was stirred at r.t. for 25 h. Compound **49RR** was isolated as a white foam (28 mg, 98%). ^1^H NMR (CDCl_3_): δ 7.50 – 7.37 (m, 3H), 7.23 – 7.16 (m, 2H), 7.16 – 7.09 (m, 5H), 6.95 (dd, *J* 6.7, 2.8 Hz, 2H), 6.58 – 6.48 (m, 2H), 4.93 (d, *J* 17.2 Hz, 1H), 4.74 (m, 1H), 4.13 (s, 1H), 3.84 (d, *J* 17.2 Hz, 1H), 3.43 (dd, *J* 14.1, 3.3 Hz, 1H), 3.19 (dd, *J* 14.0, 3.8 Hz, 1H); ^13^C NMR (CDCl_3_): δ 171.4, 166.3, 165.2, 136.6, 136.1, 134.4, 133.4, 130.4, 129.0, 128.7, 128.6, 128.0, 65.4, 62.2, 45.9, 37.6; HRMS *m/z* [M + H]^+^ calculated for C_25_H_21_ClN_2_O_4_: 449.1268. Found: 449.1228; $\alpha_{D}^{20}$ = 51.1 (c 0.1, CH_2_Cl_2_).

**(3*R*,6*S*)-3-Benzyl-6-(4-chlorophenyl)-1-phenyl-4-(carboxy-methyl)-piperazine-2,5-dione (49RS)**

Compound **49RS** was synthesised following general procedure E from **41RS** (28 mg, 0.055 mmol) and conc. HCl (12 M, 6 ml). The reaction was stirred at r.t. for 25 h. Compound **49RS** was isolated as a white foam (quantitative yield). ^1^H NMR (CDCl_3_): δ 7.98 (s, 1H), 7.33 (dd, *J* 4.9, 2.2 Hz, 3H), 7.30 – 7.20 (m, 5H), 7.19 – 7.11 (m, 2H), 7.10 – 7.03 (m, 2H), 6.99 – 6.92 (m, 2H), 5.58 (s, 1H), 4.55 (dd, *J* 7.9, 4.1 Hz, 1H), 4.46 (d, *J* 17.5 Hz, 1H), 3.26 (dd, *J* 14.0, 4.0 Hz, 1H), 3.14 (d, *J* 17.5 Hz, 1H), 3.04 (dd, *J* 13.9, 7.9 Hz, 1H); ^13^C NMR (CDCl_3_): δ 171.2, 166.4, 166.3, 138.0, 135.6, 135.2, 133.4, 129.9, 129.6, 129.3, 129.1, 128.9, 127.9, 127.6, 127.0, 66.6, 63.8, 47.1, 38.9; HRMS *m/z* [M + H]^+^ calculated for C_25_H_21_ClN_2_O_4_: 449.1268. Found: 449.1244; $\alpha_{D}^{20}$ = 14.3 (c 0.1, CH_2_Cl_2_).

**(3*R*,6*S*)-3-Benzyl-6-(3-chlorophenyl)-1-phenyl-4-(carboxy-methyl)-piperazine-2,5-dione (50RS)**

Compound **50RS** was synthesised following general procedure E from **42RS** (45 mg, 0.09 mmol) and conc. HCl (12 M, 2.5 ml). The reaction was stirred at r.t. for 23 h. Compound **50RS** was isolated as a white foam (34 mg, 85%). Diastereomeric ratio of 3.33:1, determined from ^1^H NMR spectra. ^1^H NMR (CDCl_3_): δ 7.48 – 7.10 (m, 12H), 6.70 – 6.53 (m, 2H), 5.58 (s, 1H), 4.65 (t, *J* 5.2 Hz, 1H), 4.59 (d, *J* 17.5 Hz, 1H), 3.45 (d, *J* 17.3 Hz, 1H), 3.29 (d, *J* 5.1 Hz, 2H); ^13^C NMR (CDCl_3_): δ 170.5, 165.9, 165.7, 138.9, 135.2, 134.5, 134.0, 130.2, 129.4, 129.3, 128.9, 128.8, 127.89, 127.85, 126.5, 65.8, 62.9, 46.5, 38.1; HRMS *m/z* [M + H]^+^ calculated for C_25_H_21_ClN_2_O_4_: 449.1268. Found: 449.1250.

**(3*R*,6*R*)-3-Benzyl-6-(3-chlorophenyl)-1-phenyl-4-(carboxy-methyl)-piperazine-2,5-dione (51RR)**

Compound **51RR** was synthesised following general procedure E from **43RR** (64 mg, 0.13 mmol) and conc. HCl (12 M, 1.5 ml). The reaction was stirred at r.t. for 23 h. Compound **51RR** was isolated as white solids (44 mg, 77%). Diastereomeric ratio of 5:1, determined from ^1^H NMR spectra. ^1^H NMR (CDCl_3_): δ 7.53 – 7.37 (m, 3H), 7.23 – 6.97 (m, 8H), 6.85 – 6.83 (m, 1H), 6.68 – 6.55 (m, 2H), 4.95 (d, *J* 17.2 Hz, 1H), 4.75 (br t, *J* 3.7 Hz, 1H), 4.12 (s, 1H), 3.84 (d, *J* 17.3 Hz, 1H), 3.45 (d, *J* 14.0 Hz, 1H), 3.18 (d, *J* 14.0 Hz, 1H); ^13^C NMR (CDCl_3_): δ 171.1, 166.0, 165.2, 138.4, 137.8, 134.3, 134.2, 130.5, 129.6, 129.03, 128.96, 128.9, 128.6, 128.0, 127.8, 127.3, 126.9, 64.9, 62.1, 45.8, 37.6; HRMS *m/z* [M + H]^+^ calculated for C_25_H_21_ClN_2_O_4_: 449.1268. Found: 449.1259.

**(3*R*,6*S*)-3-Benzyl-6-(3-chlorophenyl)-1-phenyl-4-(carboxy-methyl)-piperazine-2,5-dione (51RS)**

Compound **51RS** was synthesised following general procedure E from **43RS** mp518 (60 mg, 0.12 mmol) and conc. HCl (12 M, 1.5 ml). The reaction was stirred at r.t. for 23 h. Compound **51RS** was isolated as white solids (52 mg, 98%). Diastereomeric ratio of 3:1, determined from ^1^H NMR spectra. ^1^H NMR (CDCl_3_): δ 7.70 (s, 1H), 7.39 – 7.28 (m, 5H), 7.25 – 7.05 (m, 8H), 6.76 – 6.56 (m, 1H), 5.61 (s, 1H), 4.59 (br s, 1H), 4.50 (d, *J* 17.4 Hz, 1H), 3.33 – 3.06 (m, 3H); ^13^C NMR (CDCl_3_): δ 170.9, 166.1, 165.8, 139.1, 137.4, 135.3, 134.7, 130.0, 129.8, 129.4, 129.2, 128.9, 128.0, 127.8, 127.5, 126.2, 125.1, 65.9, 63.4, 46.8, 38.7; HRMS *m/z* [M + H]^+^ calculated for C_25_H_21_ClN_2_O_4_: 449.1268. Found: 449.1240.

**(3*R*,6*R*)-3-Benzyl-1,6-(di-4-chlorophenyl)-4-(carboxy-methyl)-piperazine-2,5-dione (52RR)**

Compound **52RR** was synthesised following general procedure E from **44RR** (30 mg, 0.12 mmol) and conc. HCl (12 M, 1.5 ml). The reaction was stirred at r.t. for 24 h. Compound **52RR** was isolated as white solids (19 mg, 71%). Diastereomeric ratio of 4.2:1, determined from ^1^H NMR spectra. ^1^H NMR (CDCl_3_): δ 7.47 – 7.49 (m, 3H), 7.19 – 7.03 (m, 6H), 6.88 (d, *J* 8.0 Hz, 2H), 6.54 (d, *J* 8.3 Hz, 2H), 4.94 (d, *J* 16.8 Hz, 1H), 4.73 (s, 1H), 4.07 (s, 1H), 3.84 (d, *J* 17.0 Hz, 1H), 3.43 (d, *J* 13.4 Hz, 1H), 3.18 (d, *J* 13.9 Hz, 1H); ^13^C NMR (CDCl_3_): δ 170.9, 165.9, 165.4, 136.3, 134.7, 134.5, 134.2, 133.6, 130.4, 129.9, 129.2, 129.1, 128.8, 128.6, 128.1, 64.6, 62.2, 45.9, 37.7; HRMS *m/z* [M + H]^+^ calculated for C_25_H_20_Cl_2_N_2_O_4_: 483.0878. Found: 483.0878.

**(3*R*,6*S*)-3-Benzyl-1,6-(di-4-chlorophenyl)-4-(carboxy-methyl)-piperazine-2,5-dione (52RS)**

Compound **52RS** was synthesised following general procedure E from **44RS** (11 mg, 0.02 mmol) and conc. HCl (12 M, 1.5 ml). The reaction was stirred at r.t. for 24 h. Compound **52RS** was isolated as white solids (8.4 mg, 85%). Diastereomeric ratio of 1.6:1, determined from ^1^H NMR spectra. ^1^H NMR (CDCl_3_): δ 7.47 – 7.36 (m), 7.31 – 7.25 (m), 7.24 – 6.98 (m) (11H), 6.67 (d, *J* 8.2 Hz, 2H), 5.51 (s, 1H), 4.64 – 4.50 (m, 2H), 3.49 – 3.39 (m, 1H), 3.32 – 3.11 (m, 2H); ^13^C NMR (CDCl_3_): δ 170.5, 165.8, 165.7, 137.6, 135.3, 134.9, 133.8, 133.7, 130.6, 130.2, 129.7, 129.5, 129.2, 128.7, 127.8, 66.0, 63.4, 46.8, 38.5; HRMS *m/z* [M + H]^+^ calculated for C_25_H_20_Cl_2_N_2_O_4_: 483.0878. Found: 483.0853.

**(3*S*,6*S*)-1-(4-Chlorophenyl)-6-(3-chlorophenyl)-4-(carboxy-methyl)-3-methyl-piperazine-2,5-dione (53SS)**

Compound **53SS** was synthesised following general procedure E from **45SS** (5.7 mg, 0.012 mmol) and conc. HCl (12 M, 2 ml). The reaction was stirred at r.t. for 3 days. Compound **53SS** was isolated as a colourless oil (4 mg, 80%). ^1^H NMR (CDCl_3_): δ 7.39 – 7.38 (m, 1H), 7.35 – 7.28 (m, 4H), 7.23 – 7.20 (m, 1H), 7.15 – 7.09 (m, 2H), 5.46 (s, 1H), 4.65 (d, *J* 17.9 Hz, 1H), 4.40 (q, *J* 6.9 Hz, 1H), 4.02 (d, *J* 17.9 Hz, 1H), 1.63 (d, *J* 6.9 Hz, 3H); ^13^C NMR (CDCl_3_): δ 171.7, 166.8, 166.5, 137.9, 136.6, 135.3, 133.3, 130.4, 129.5, 129.3, 127.3, 127.0, 125.1, 66.6, 54.9, 44.2, 16.1; HRMS *m/z* [M + H]^+^ calculated for C_19_H_16_Cl_2_N_2_O_4_: 407.0565. Found: 407.0560; $\alpha_{D}^{20}$ = -25.2 (c 0.1, CHCl_3_).

**(3*S*,6*R*)-1-(4-Chlorophenyl)-6-(3-chlorophenyl)-4-(carboxy-methyl)-3-methyl-piperazine-2,5-dione (53SR)**

Compound **53SR** was synthesised following general procedure E from **45SR** (17 mg, 0.036 mmol) and conc. HCl (12 M, 2 ml). The reaction was stirred at r.t. for 3 days. Compound **53SR** was isolated as a colourless oil (12 mg, 81%). ^1^H NMR (CDCl_3_): δ 7.45 (s, 1H), 7.38 – 7.27 (m, 5H), 7.20 (d, *J* 8.7 Hz, 2H), 5.52 (s, 1H), 4.37 (d, *J* 17.4 Hz), 4.33 (q, *J* 7.2 Hz), (2H), 3.92 (d, *J* 17.5 Hz, 1H), 1.57 (d, *J* 7.1 Hz, 3H); ^13^C NMR (CDCl_3_): δ 171.0, 167.3, 164.7, 137.6, 137.3, 135.2, 133.5, 130.3, 129.6, 129.3, 127.4, 127.0, 124.8, 66.1, 58.0, 46.2, 19.1; HRMS *m/z* [M + H]^+^ calculated for C_19_H_16_Cl_2_N_2_O_4_: 407.0565. Found: 407.0559; $\alpha_{D}^{20}$ = 56.0 (c 0.1, CHCl_3_).

**(3*S*)-4-(Carboxy-methyl)-1,3-dibenzyl-1,4-diazaspiro[5.5]undecane-2,5-dione (54S)**

Compound **54S** was synthesised following general procedure E from **30S** (164 mg, 0.36 mmol) and conc. HCl (12 M, 8.7 ml). The reaction was stirred at 70 °C for 48 h. Compound **54S** was isolated as a colourless thick oil (144 mg, 95%). ^1^H NMR (CDCl_3_): δ 9.89 (s, 1H), 7.33 – 7.23 (m, 5H), 7.22 – 7.10 (m, 5H), 5.01 (d, *J* 16.1 Hz, 1H), 4.52 – 4.48 (m, 1 Hz, 1H), 4.44 (d, *J* 17.3 Hz, 1H), 4.35 (d, *J* 16.1 Hz, 1H), 3.47 (d, *J* 17.3 Hz, 1H), 3.33 (dd, *J* 14.2, 5.8 Hz, 1H), 3.27 (dd, *J* 14.2, 3.4 Hz, 1H), 2.23 – 2.12 (m, 1H), 1.88 – 1.80 (m, 1H), 1.66 – 1.44 (m, 4H), 1.46 – 1.33 (m, 2H), 1.02 – 0.93 (m 1H), 0.88 – 0.83 (m, 1H); ^13^C NMR (CDCl_3_): δ 172.6, 170.2, 167.4, 137.8, 136.0, 130.0, 129.0, 128.6, 127.6, 127.0, 126.3, 62.8, 62.6, 47.2, 46.0, 38.8, 34.7, 33.4, 24.3, 23.1, 22.3; HRMS *m/z* [M + H]^+^ calculated for C_25_H_28_N_2_O_4_: 421.2127. Found: 421.2107; $\alpha_{D}^{20}$ = -14.2 (c 0.1, DCM).

**(3*S*)-4-(Carboxy-methyl)-1,3-dibenzyl-1.4 diazaspiro[5.5]un-decane-2,5-dione (54S)**

Using general procedure E, **31S** (164 mg, 0.36 mmol) and conc. HCl (12 M, 8.7 ml) provided **54S** (144 mg, 93%) as a colourless thick oil. The reaction was run at r.t. ^1^H NMR and ^13^C NMR spectra in accordance with above.

**(3*R*,6*R*)-1,3-Dibenzyl-4-((4-*tert*-butoxycarbonyl)piperazine-1-yl)-carbonyl-methyl)-6-phenylpiperazine-2,5-dione (55RR)**

Compound **55RR** was synthesised following general procedure F from **46RR** (41 mg, 0.096 mmol), 1-(tert-butoxycarbonyl)piperazine (27 mg, 0.14 mmol), HATU (48 mg, 0.13 mmol) and Et_3_N (40 µl, 0.30 mmol). The reaction was stirred overnight at r.t. Purification by automated flash column chromatography (20% ethyl acetate in pentane) provided **55RR** as white solids (51 mg, 89%). ^1^H NMR (CDCl_3_): δ 7.37 – 7.15 (m, 11H), 7.13 – 7.06 (m, 2H), 6.77 – 6.70 (m, 2H), 5.28 (d, *J* 14.7 Hz, 1H), 5.06 (d, *J* 15.9 Hz, 1H), 4.85 (t, *J* 4.2 Hz, 1H), 4.28 (s, 1H), 3.62 (d, *J* 15.9 Hz, 1H), 3.58 – 3.53 (m, 1H), 3.52 – 3.19 (m, 10H), 1.46 (s, 9H); ^13^C NMR (CDCl_3_): δ 166.2, 165.2, 165.1, 154.5, 136.8, 135.4, 134.9, 129.9 (2C), 129.1, 128.91, 128.88, 128.6, 128.3, 127.7, 127.4, 80.6, 62.3, 60.8, 46.9, 44.8, 44.4, 41.9, 37.0, 28.5; HRMS *m/z* [M+H]^+^ calculated for C_35_H_40_N_4_O_5_: 597.3077. Found: 597.3083; $\alpha_{D}^{20}$ = -13.8 (c 0.1, CH_2_Cl_2_).

**(*3R,6S*)-1,3-Dibenzyl-4-((4-*tert*-butoxycarbonyl)piperazine-1-yl)-carbonyl-methyl)-6-phenylpiperazine-2,5-dione (55RS)**

Compound **55RS** was synthesised following general procedure F from **46RS** (127 mg, 0.30 mmol), 1-(*tert*-butoxycarbonyl)piperazine (85 mg, 0.45 mmol), HATU (148 mg, 0.40 mmol), Et_3_N (120 µl ml, 0.86 mmol) and purification by automated flash column chromatography (50-70% ethyl acetate in pentane) provided **55RS** as white solids (137 mg, 78%). ^1^H NMR (CDCl_3_): δ 7.35 – 7.24 (m, 9H), 7.24 – 7.19 (m, 2H), 7.12 – 7.04 (m, 4H), 5.61 (d, *J* 14.9 Hz, 1H), 5.02 (s, 1H), 4.54 – 4.40 (m, 2H), 3.65 (d, *J* 15.0 Hz, 1H), 3.59 – 3.43 (m, 2H), 3.39 – 3.32 (m, 3H), 3.31 – 3.36 (m, 2H), 3.14 – 3.07 (m, 1H), 3.05– 2.96 (m, 2H), 2.89 (d, *J* 16.1 Hz, 1H), 1.45 (s, 9H); ^13^C NMR (CDCl_3_): δ 166.4, 166.0, 165.1, 154.5, 136.8, 135.1, 135.0, 129.7, 129.2, 129.11, 129.06, 128.7, 128.3, 128.1, 127.5, 126.7, 80.6, 63.5, 62.3, 47.9, 46.3*,* 44.6, 42.0, 39.6, 28.5; HRMS *m/z* [M+H]^+^ calculated for C_35_H_40_N_4_O_5_: 597.3077. Found: 597.3087; $\alpha_{D}^{20}$ = 43.9 (c 0.1, CH_2_Cl_2_).

**(*3R,6R*)-3-Benzyl-1-(4-chlorobenzyl)-4-((4-*tert*-butoxycarbonyl)piperazine-1-yl)-carbonyl-methyl)-6-phenylpiperazine-2,5-dione (56RR)**

Compound **56RR** was synthesised following general procedure F from **47RR** (43 mg, 0.093 mmol), 1-(*tert*-butoxycarbonyl)piperazine (25.0 mg, 0.134 mmol), HATU (47 mg, 0.124 mmol) and Et_3_N (40 µl, 0.287 mmol). The reaction was stirred overnight at r.t. Purification by automated flash column chromatography (50-100% ethyl acetate in pentane) provided **56RR** as a colourless oil (50 mg, 85%). ^1^H NMR (CDCl_3_): δ 7.34 – 7.18 (m, 6H), 7.16 – 7.06 (m, 6H), 6.64 – 6.59 (m, 2H), 5.10 (d, *J* 14.7 Hz), 5.08 (d, *J* 15.6 Hz) (2H), 4.82 (t, *J* 4.0 Hz, 1H), 4.16 (s, 1H), 3.64 (d, *J* 15.9 Hz, 1H), 3.61 – 3.55 (m, 1H), 3.49 – 3.25 (m, 10H), 1.45 (s, 9H); ^13^C NMR (CDCl_3_): δ 166.1, 165.1, 165.1, 154.5, 136.5, 135.2, 133.53, 133.50, 130.1, 130.0, 129.1, 129.0, 128.9, 128.7, 128.4, 127.5, 80.5, 62.5, 60.9, 46.4, 44.8, 44.4, 42.0, 37.0, 28.5; HRMS *m/z* [M+H]^+^ calculated for C_35_H_39_ClN_4_O_5_: 631.2687. Found: 631.2717.

**(3*R*,6*S*)-3-Benzyl-1-(4-chlorobenzyl)-4-((4-*tert*-butoxycarbonyl)piperazine-1-yl)-carbonyl-methyl)-6-phenylpiperazine-2,5-dione (56RS)**

Compound **56RS** was synthesised following general procedure F from **47RS** (100 mg, 0.216 mmol), 1-(*tert*-butoxycarbonyl)piperazine (62 mg, 0. 32 mmol), HATU (109 mg, 0.287 mmol) and Et_3_N (90 µl, 0.65 mmol). The reaction was stirred overnight at r.t. Purification by automated flash column chromatography (50% ethyl acetate in pentane) provided **56RS** as a colourless oil (80 mg, 59%). ^1^H NMR (CDCl_3_): δ 7.35 – 7.23 (m, 8H), 7.19 – 7.14 (m, 2H), 7.09 – 7.02 (m, 4H), 5.52 (d, *J* 15.1 Hz, 1H), 4.98 (s, 1H), 4.48 (d, *J* 16.2 Hz), 4.45 – 4.42 (m) (2H), 3.66 (d, *J* 15.0 Hz, 1H), 3.59 – 3.41 (m, 2H), 3.39 – 3.27 (m, 5H), 3.14 – 3.07 (m, 1H), 3.02 – 2.94 (m, 2H), 2.83 (d, *J* 16.2 Hz, 1H), 1.45 (s, 9H); ^13^C NMR (CDCl_3_): δ 166.4, 165.9, 165.0, 154.5, 136.8, 135.0, 133.9, 133.7, 129.70, 129.65, 129.20, 129.17, 129.1, 128.7, 127.5, 126.6, 80.5, 63.6, 62.6, 47.5, 46.4, 44.5, 42.0, 39.6, 28.5; HRMS *m/z* [M+H]^+^ calculated for C_35_H_39_ClN_4_O_5_: 631.2687. Found: 631.2684; $\alpha_{D}^{20}$ = 54.0 (c 0.1, CH_2_Cl_2_).

**(*3R,6S*)-3-Benzyl-1-(4-chlorobenzyl)-4-(((3-oxo)-piperazine-1-yl)-carbonyl-methyl)-6-phenylpiperazine-2,5-dione (57RS)**

Compound **57RS** was synthesised following general procedure F from **47RS** (99 mg, 0.21 mmol), 2-oxopiperazine (32 mg, 0.32 mmol), HATU (106 mg, 0.279 mmol) and Et_3_N (90 µl, 0.65 mmol). The reaction was stirred overnight at r.t. Purification by flash column chromatography (5% methanol in DCM) provided **57RS** as a colourless oil (69 mg, 59%). ^1^H and ^13^C NMR recoded at 55 °C. ^1^H NMR (CDCl_3_): δ 7.37 – 7.30 (m, 3H), 7.29 – 7.23 (m, 5H), 7.18 – 7.10 (m, 4H), 7.07 – 7.02 (m, 2H), 5.48 (d, *J* 15.1 Hz, 1H), 4.99 (s, 1H), 4.47 – 4.40 (m, 2H), 4.26 – 3.54 (m), 3.70 (d, *J* 15.0 Hz) (5H), 3.41 – 3.18 (m, 3H), 2.95 (dd, *J* 13.8, 9.1 Hz, 1H), 2.86 (d, *J* 16.4 Hz, 1H); ^13^C NMR (CDCl_3_): δ 166.6, 166.0, 165.9, 165.3, 136.9, 135.2, 134.0, 133.9, 129.7, 129.3, 129.2, 129.1, 128.8, 127.6, 126.7, 63.9, 62.9, 47.8, 46.7, 40.8, 39.9, 39.1; HRMS *m/z* [M + H]^+^ calculated for C_30_H_29_ClN_4_O_4_: 545.1955. Found: 545.1924; $\alpha_{D}^{20}$ = 39.5 (c 0.1, CH_2_Cl_2_).

**(3*S*)-1,3-Dibenzyl-4-((4-*tert*-butoxycarbonyl)piperazine-1-yl)-carbonyl-methyl)-1,4-diazaspiro[5.5]undecane-2,5-dione (58S)**

Compound **58S** was synthesised following general procedure F from **54S** (71 mg, 0.17 mmol), 1-(*tert*-butoxycarbonyl)piperazine (48 mg, 0.25 mmol), HATU (85 mg, 0.22 mmol) and Et_3_N (70 µl, 0.3 mmol). The reaction was stirred overnight at r.t. Purification by automated flash column chromatography (50-60% ethyl acetate in pentane) provided **58S** as white solids (32 mg, 32%). ^1^H NMR (CDCl_3_): δ 7.34 – 7.23 (m, 5H), 7.25 – 7.15 (m, 5H), 5.00 (d, *J* 16.2 Hz, 1H), 4.54 – 4.45 (m, 2H), 4.41 (d, *J* 16.2 Hz, 1H), 3.57 – 3.48 (m, 2H), 3.47 – 3.32 (m, 6H), 3.28 (dd, *J* 14.2, 6.7 Hz, 1H), 3.26 – 3.10 (m, 2H), 2.26 – 2.09 (m, 1H), 1.95 – 1.86 (m, 1H), 1.68 – 1.55 (m, 2H), 1.53 – 1.43 (m), 1.47 (s), (12H), 1.14 – 1.11 (m, 1H), 1.06 – 0.94 (m, 1H); ^13^C NMR (CDCl_3_): δ 170.2, 167.5, 165.3, 154.6, 138.2, 137.2, 129.8, 128.9, 128.6, 127.3, 126.9, 126.4, 80.6, 62.7, 62.5, 46.3, 45.9, 44.5, 43.3, 42.0, 38.9, 35.1, 33.3, 28.5, 24.4, 23.2, 22.4; HRMS *m/z* [M + H]^+^ calculated for C_34_H_44_N_4_O_5_: 589.3390. Found: 589.3390; $\alpha_{D}^{20}$ = -10.6 (c 0.1, CH_2_Cl_2_).

**(3*R*,6*R*)-1,3-Dibenzyl-4-(piperazine-1-yl-carbonyl-methyl)-6-phenylpiperazine-2,5-dione (59RR)**

Compound **59RR** was synthesised following general procedure G from, **55RR** (17.0 mg, 0.0285 mmol), TFA (0.30 ml) in DCM (0.30 ml). Compound **59RR** was obtained as an off white solid (11.6 mg, 82%). ^1^H NMR (CDCl_3_): δ 7.37 – 7.16 (m, 11H), 7.12 – 7.06 (m, 2H), 6.77 – 6.70 (m, 2H), 5.27 (d, J 14.7 Hz, 1H), 5.04 (d, *J* 15.9 Hz, 1H), 4.85 (dd, *J* 4.6, 3.6 Hz, 1H), 4.26 (s, 1H), 3.65 (d, *J* 15.9 Hz, 1H), 3.62 – 3.57 (m, 1H), 3.56 – 3.52 (m, 1H), 3.48 (dd, *J* 15.6, 3.9 Hz, 1H), 3.41 – 3.30 (m, 4H), 2.89 – 2.71 (m, 4H); ^13^C NMR (CDCl_3_): δ 166.3, 165.04, 165.02, 136.8, 135.3, 134.9, 129.9, 129.1, 128.97, 128.95, 128.93, 128.91, 128.6, 128.4, 127.8, 127.4, 62.3, 60.8, 46.9, 45.5, 45.2, 45.1, 44.4, 42.1, 37.0; HRMS *m/z* [M+H]^+^ calculated for C_30_H_32_N_4_O_3_: 497.2552. Found: 497.2542; $\alpha_{D}^{20}$ = -9.7 (c 0.1, CH_2_Cl_2_).

**(3*R*,6*S*)-1,3-Dibenzyl-4-(piperazine-1-yl-carbonyl-methyl)-6-phenylpiperazine-2,5-dione (59RS)**

Compound **59RS** was synthesised following general procedure G from, **55RS** (60 mg, 0.10 mmol), and TFA (1 ml). Compound **59RS** was obtained as an off white solid (38 mg, 76%). ^1^H NMR (CDCl_3_): δ 7.34 – 7.25 (m, 9H), 7.23 – 7.19 (m, 2H), 7.10 – 7.03 (m, 4H), 5.59 (d, *J* 14.9 Hz, 1H), 5.00 (s, 1H), 4.55 – 4.45 (m), 4.48 (d, *J* 16.7 Hz) (2H), 3.68 – 3.46 (m), 3.63 (d, *J* 14.9 Hz) (3H), 3.35 (dd, *J* 13.9, 3.8 Hz, 1H), 3.21– 3.04 (m), 3.05 (dd, *J* 13.9, 8.5 Hz) (4H), 2.93 (d, *J* 16.2 Hz, 1H), 2.86 – 2.71 (m, 4H); ^13^C NMR (CDCl_3_): δ 166.3, 165.9, 164.9, 136.8, 135.2, 135.1, 129.8, 129.13, 129.07, 129.0, 128.6, 128.3, 128.0, 127.4, 126.7, 63.4, 62.4, 47.9, 46.2, 45.5, 45.3, 45.1, 42.4, 39.5; HRMS *m/z* [M+H]^+^ calculated for C_30_H_32_N_4_O_3_: 497.2552. Found: 497.2500; $\alpha_{D}^{20}$ = 78.8 (c 0.1, CH_2_Cl_2_).

**(3*R*,6*R*)-3-Benzyl-1-(4-chlorobenzyl)-4-(piperazine-1-yl-carbonyl-methyl)-carbonyl-methyl)-6-phenylpiperazine-2,5-dione (60RR)**

Compound **60RR** was synthesised following general procedure G from **56RR** (43.0 mg, 0.0681 mmol) and TFA (0.8 ml). Compound **60RR** was obtained as an off white solid (35 mg, 97%). ^1^H NMR (CDCl_3_): δ 7.40 – 7.12 (m, 12H), 6.67 – 6.63 (m, 2H), 5.15 (d, *J* 16.0 Hz, 1H), 5.14 (d, *J* 14.7 Hz, 1H), 4.89 – 4.86 (m, 1H), 4.19 (s, 1H), 3.69 (d, *J* 16.0 Hz, 1H), 3.64 – 3.52 (m, 2H), 3.49 (dd, *J* 14.5, 4.1 Hz, 1H), 3.42 (d, *J* 14.9 Hz), 3.42 – 3.32 (m) (4H), 2.89 – 2.78 (m, 4H); ^13^C NMR (CDCl_3_): δ 166.1, 165.2, 164.8, 136.6, 135.2, 133.6, 133.5, 130.2, 130.0, 129.1, 128.94, 128.90, 128.7, 128.4, 127.5, 62.5, 60.8, 46.3, 46.2, 45.7, 44.4, 43.2, 36.8; HRMS *m/z* [M+H]^+^ calculated for C_30_H_31_ClN_4_O_3_: 531.2163. Found: 531.2175; $\alpha_{D}^{20}$ = 24.4 (c 0.1, CH_2_Cl_2_).

**(3*R*,6*S*)-3-Benzyl-1-(4-chlorobenzyl)-4-(piperazine-1-yl-carbonyl-methyl)-carbonyl-methyl)-6-phenylpiperazine-2,5-dione (60RS)**

Compound **60RS** was synthesised following general procedure G from **56RS** (66 mg, 0.11 mmol) and TFA (1 ml). Compound **60RS** was obtained as a colourless oil (45 mg, 81%). ^1^H NMR (CDCl_3_): δ 7.34 – 7.22 (m, 8H), 7.18 – 7.14 (m, 2H), 7.08 – 7.02 (m, 4H), 5.50 (d, *J* 14.8 Hz, 1H), 4.98 (s, 1H), 4.50 (d, *J* 16.2 Hz, 1H), 4.45 (dd, *J* 8.7, 3.8 Hz, 1H), 3.65 (d, *J* 15.0 Hz, 1H), 3.59 – 3.42 (m, 2H), 3.32 (dd, *J* 13.8, 3.8 Hz, 1H), 3.14 – 2.96 (m), 2.99 (dd, *J* 13.8, 8.7 Hz) (3H), 2.85 (d, *J* 16.2 Hz, 1H), 2.80 – 2.62 (m, 4H); ^13^C NMR (CDCl_3_): δ 166.4, 165.8, 164.7, 136.7, 135.1, 133.8, 133.7, 129.74, 129.68, 129.2, 129.1, 128.7, 127.4, 126.6, 63.5, 62.6, 47.4, 46.3, 46.0, 45.9, 45.8, 43.2, 39.4; HRMS *m/z* [M+H]^+^ calculated for C_30_H_31_ClN_4_O_3_: 531.2163. Found: 531.2174; $\alpha_{D}^{20}$ = 25.5 (c 0.1, CH_2_Cl_2_).

**(3*S*)-1,3-Dibenzyl-4-(piperazine-1-yl-carbonyl-methyl)-1,4-diazaspiro[5.5]undecane-2,5-dione (61S)**

Compound **61S** was synthesised following general procedure G from **58S** (17.0 mg, 0.0285 mmol) and TFA (0.5 ml). Compound **61S** was obtained as a colourless oil/foam (17 mg, quantitative yield). ^1^H NMR (CDCl_3_): δ 7.33 – 7.23 (m, 5H), 7.22 – 7.18 (m, 5H), 4.99 (d, *J* 16.2 Hz, 1H), 4.53 (d, *J* 16.0 Hz, 1H), 4.49 (dd, *J* 6.3, 4.1 Hz, 1H), 4.41 (d, *J* 16.2 Hz, 1H), 3.61 – 3.55 (m, 2H), 3.44 (d, *J* 16.0 Hz, 1H), 3.40 – 3.24 (m, 2H), 3.27 – 3.18 (m, 2H), 2.86 –2.80 (m, 4H), 2.28 (s, 1H), 2.26 – 2.11 (m, 1H), 1.96 – 1.87 (m, 1H), 1.66 – 1.56 (m, 3H), 1.57 – 1.38 (m, 3H), 1.08 – 1.03 (m, 1H), 1.04 – 0.91 (m, 1H); ^13^C NMR (CDCl_3_): δ 170.1, 167.4, 165.0, 138.2, 137.2, 129.8, 128.9, 128.6, 127.3, 126.8, 126.5, 62.6, 62.4, 46.1, 45.9, 45.6, 43.0, 38.7, 35.0, 33.4, 24.4, 23.2, 22.5; HRMS *m/z* [M + H]^+^ calculated for C_29_H_36_N_4_O_3_: 489.2866. Found: 489.2876; $\alpha_{D}^{20}$ = -7.4 (c 0.1, CH_2_Cl_2_).

**(3*S*)-1,3-Dibenzyl-4-(2-hydroxyethyl)-1,4-diazaspiro[5.5]undecane-2,5-dione (62S)**

Compound **17S** (100 mg, 0.22 mmol) was dissolved in absolute ethanol (2 ml) and cooled to 0 °C on an ice bath under N_2_ (g). Sodium borohydride (13 mg, 0.34 mmol) was added and the reaction was stirred at 0 °C for 72 h. Additional NaBH_4_ (12 mg, 0.33 mmol) was added and the reaction was left at r.t. for additional 48 h. The reaction was quenched by the addition of sat. NH_4_Cl (aq.) and extracted with ethyl acetate. The crude product was purified by automatic flash chromatography (75% ethyl acetate in pentane) providing **62S** as a colourless oil (55 mg, 61%). *Note difficult to remove solvents from the product.* ^1^H NMR (CDCl_3_): δ 7.32 – 7.19 (m, 6H), 7.17 – 7.12 (m, 4H), 4.94 (d, *J* 16.0 Hz, 1H), 4.52 (t, *J* 4.6 Hz, 1H), 4.25 (d, *J* 16.0 Hz, 1H), 3.98 (ddd, *J* 14.2, 6.3, 3.4 Hz, 1H), 3.91 – 3.73 (m, 2H), 3.33 (dd, *J* 14.1, 4.7 Hz, 1H), 3.26 – 3.19 (m, 2H), 3.08 (s, 1H), 2.19 (qt, *J* 13.1, 4.3 Hz, 1H), 1.79 – 1.69 (m, 1H), 1.65 – 1.44 (m, 4H), 1.36 – 1.16 (m, 2H), 0.94 (qt, *J* 13.6, 4.3 Hz, 1H), 0.42 – 0.30 (m, 1H); ^13^C NMR (CDCl_3_): δ 171.2, 167.0, 138.1, 135.9, 130.2, 128.9, 128.6, 127.5, 127.0, 126.5, 62.7, 62.5, 61.7, 49.0, 46.0, 38.3, 34.2, 33.9, 24.3, 23.1, 22.2; HRMS *m/z* [M + H]^+^ calculated for C_25_H_30_N_2_O_3_: 407.2335. Found: 407.2341; $\alpha_{D}^{20}$ = -13.3 (c 0.1, CH_2_Cl_2_).

# Fluorescence Polarization (FP) Assay

The FP assay was performed in black 384-well microtiter plates at a final volume of 20 μL per well. Assay plates were prepared by addition of 20 μL of assay buffer (10 mM Tris (pH 8.0), 42.5 mM NaCl, and 0.0125% Tween-20) containing 1 μM GST-MDM2 (1 – 188) and Texas Red labeled wild-type p53 peptide (15 nM, amino acids 11 – 29: GSGSSQETFSDLWKLLPEN) to each well using a WellMate instrument (Matrix). For testing, a dilution series of small molecules (spanning 10 mM to 0.5 μM in DMSO, in 1:3-dilution) was added by direct addition to the assay plate by Biomek FX lab automation workstation (Beckman Coulter, Inc., Fullerton, CA) using pin transfer (100ss pins, V&P scientific) giving a test dilution series spanning 130 μM to 6.6 nM with a final concentration of 1.3 % DMSO. The assay mixture was incubated for 1 h at r.t. and the fluorescence polarization signal was measured on an EnVision multilabel plate reader fitted with a 555-nm excitation filter, 632-nm static and polarized filters, and a Texas Red FP dichroic mirror. Unlabelled WT-p53 peptide was used as a positive control, and DMSO was used as a negative control. Technical triplicate data was normalized to the positive (100% inhibition) and negative (0% inhibition) controls on the corresponding row of the 384-well plate (the percentage inhibition = 100 × (sample result – negative control)/(positive control mean – negative control)). Two to seven independent experiments of normalized data were combined into a data set, and then fit using a non-linear regression in GraphPad Prism with the formula log(inhibitor) vs. response – Variable slope (four parameters). Additionally the residuals of the curve fit were plotted to determine the fit of the theoretical curve. IC_50_ and 95% confidence intervals (CI) were determined from these graphs.

**Figure S1**. Fluorescence polarization-based MDM2 binding assay for DKPs. Inhibition curves for **52RR** (◇) (IC_50_ = 31.41 µM, 95% CI [16.31, 60.49]) and **52RS** (●) (IC_50_ = 27.99 µM, 95% CI [10.96, 71.43]). Wild-type p53 peptide (□, amino acids 11 – 29: GSGSSQETFSDLWKLLPEN, IC_50_ = 1.84 µM, 95% CI [1.38, 2.42]) and Nutlin-3a (▲) (IC_50_ = 0.80 µM, 95% CI [0.62, 1.02]were used as positive controls.

# WaterLOGSY measurements

All NMR experiments were performed at 298 K (25 °C) using a Bruker Avance 600 MHz spectrometer equipped with TCI cryogenic gradient probe. Compounds (20 mM stock solution in DMSO-D_6_) were added to buffer (25mM HEPES, pH7.2, 150 mM NaCl, 2mM TCEP) or buffer containing MDM2 protein (5 µM) to give final compound concentration of 200 µM. One-dimensional (1D) ^1^H- and WaterLOGSY (water-ligand observed via gradient spectroscopy)^8^ NMR spectra were recorded for compounds, protein, and compounds plus protein, respectively. NMR spectra were processed using Bruker Topspin software.

**Figure S2.** WaterLOGSY experiment. ^1^H NMR spectra and WaterLOGSY spectra of the MDM2 protein in the absence of the compound (A = ^1^H and C = WaterLOGSY) and in the presence of **52RR** (B = ^1^H and D = WaterLOGSY).

**Figure S3**. WaterLOGSY experiment. ^1^H NMR spectra and WaterLOGSY spectra of the MDM2 protein in the absence of the compound (A = ^1^H and C = WaterLOGSY) and in the presence of **52RS** (B = ^1^H and D = WaterLOGSY).

# Surface plasmon resonance (SPR) measurements

Biotinylation of MDM2

MDM2 was minimally biotinylated by reaction with EZ-Link Sulfo-NHS-LC-LC-Biotin (Thermo Scientific). The biotin reagent was added to the protein at a 0.5:1 molar ratio, and the reaction was incubated on ice for 3-4 hours. Unconjugated biotin was removed by processing the samples through two Zeba Spin Desalting Columns (Thermo Scientific) that had been equilibrated with storage buffer (20 mM Bis-Tris pH 6.5, 200 mM NaCl, 20 mM dithiothreitol, 20% glycerol). Bovine serum albumin (BSA) was added to the reaction at a final concentration of 0.1 mg/mL immediately prior to processing through the spin columns to improve recovery.^2^ The biotinylated protein was aliquotted, flash-frozen and stored at -80° C for use in subsequent binding experiments.

Kinetic/equilibrium affinity analysis of compound binding by surface plasmon resonance

SPR experiments were conducted at 20 °C using a SensiQ Pioneer optical biosensor (SensiQ Technologies). Neutravidin (Thermo Scientific) was covalently immobilized on a polysaccharide hydrogel-coated gold surface (COOH5 chip; SensiQ Technologies) using routine amine coupling chemistry in immobilization buffer (10 mM HEPES pH 7.4, 150 mM NaCl, 0.005% Tween20). Carboxyl groups on the hydrogel were activated with N-ethyl-N´-(3-dimethylaminopropyl) carbodiimide (EDC) and N-hydroxysuccinimide (NHS), and neutravidin was injected in 10 mM sodium acetate pH 5.0 until immobilization levels of ~3000-3400 RU were achieved. Remaining active sites were blocked by reaction with ethanolamine. The instrument was primed with binding buffer (20 mM Tris pH 8.0, 100 mM NaCl, 5 mM dithiothreitol, 0.005% Tween20, 5% glycerol, 5% DMSO), and biotinylated MDM2 was injected until ~1070 RU of protein was captured. Unoccupied biotin-binding sites of neutravidin on both the reference and protein surfaces were blocked with amine-PEG_2_-biotin (Thermo Scientific) to minimize potential non-specific binding by the compounds.^3^

Analytes were prepared in running buffer as a 2-fold dilution series starting at 200 µM for compounds 52RR and 52RS, at 11 µM for wild-type p53 peptide (SQETFSDLWKLLPEN) and at 500 nM for nutlin-3a, and were injected in triplicate at a flow rate of 150 μL/min. A series of buffer-only (blank) injections was included throughout the experiment to account for instrumental noise. The data were processed, double-referenced, solvent corrected and analyzed^4^ using the software package Qdat (version 2.5.5.12 , BioLogic Software). The equilibrium dissociation constants (*K*_D_) were determined by fitting the data to a 1:1 (Langmuir) interaction model. For compounds 52RR and 52RS, the *K*_D_s were determined by equilibrium affinity analysis as the concentration that gave 50% occupancy of the chip (i.e. Response = ½ Rmax). For wild-type p53 peptide and nutlin-3a, the *K*_D_s were determined by kinetic analysis as the quotient of the kinetic rate constants, *k*_d_/*k*_a_.

**Table S1.** Surface plasmon resonance results

| **Analyte** | ***k*_a_ (M^-1^s^-1^)** | ***k*_d_ (s^-1^)** | ***K*_D_ (µM)** | **Rmax (RU)** |
| --- | --- | --- | --- | --- |
| **52RR** | ND | ND | 155 (± 4)^a^ | 16.6 (± 0.2) |
| **52RS** | ND | ND | 140 (± 3)^a^ | 16.8 (± 0.2) |
| **WT p53 peptide** | 2.98 (± 0.01) x 10^5^ | 3.76 (± 0.01) x 10^-1^ | 1.26 (± 0.01)^b^ | 46.0 (± 0.1) |
| **Nutlin-3a** | 9.10 (± 0.01) x 10^5^ | 2.80 (± 0.01) x 10^-2^ | 0.0307 (±0.0001)^b^ | 28.4 (± 0.1) |

a = *K*_D_ determined by equilibrium affinity analysis as concentration at 50% occupancy of chip (i.e. Response = ½ Rmax)

b = *K*_D_ determined by kinetic analysis as quotient of kinetic rate constants, *k*_d_/*k*_a_


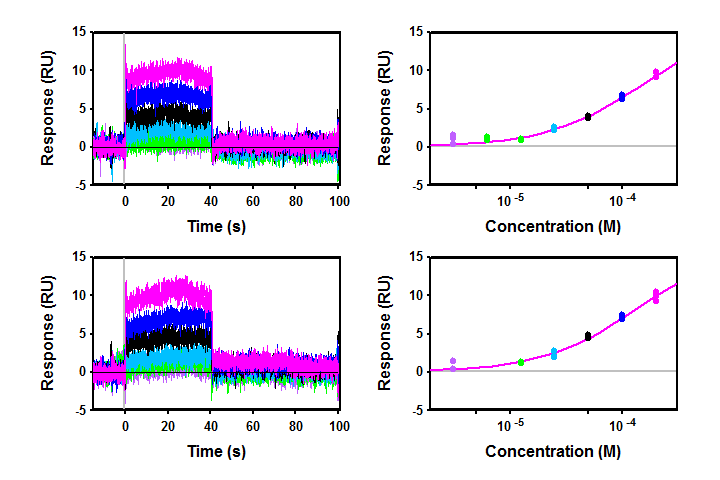


**Fig S4.** Surface plasmon resonance equilibrium affinity analyses. Sensorgrams (left) and binding isotherms (right) for compounds 52RR (top) and 52 RS (bottom). The data were fit to a 1:1 interaction model.


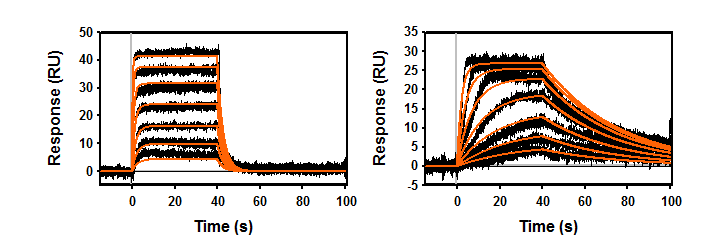


**Fig S5.** Surface plasmon resonance kinetic analyses. Sensorgrams and kinetic analyses for positive controls wild-type p53 peptide (left) and nutlin-3a (right). The data (black lines) were fit to a 1:1 interaction model (orange lines).

# Molecular modelling

Conformational analysis was performed using MacroModel^5^ in Maestro. The conformational search was performed using the OPLS-2005 force field and a Born water solvation model. The energy window for saving structures was set to 21 kJ/mol and a maximum atom deviation threshold of 0.5 Å. The torsional sampling mode (MCMM) was employed with 10000 steps per rotable bond. PRCG was used as the minimization method with a maximum of 10000 iterations. Protein crystal complexes were retrieved from the PDB database and prepared with the protein preparation wizard workflow in Maestro using default settings (minimization with OPLS2005 as forcefield converging heavy atoms to RMSD of 0.30Å)^6^ Grids for docking were prepared using Glide[11] in maestro. Grids were centred around the co-crystallized ligands with a box size of 20 Å. Prior to docking, all ligands were prepared using Ligprep[12] in maestro with OPLS_2005 as forcefield. Epik was used to generate possible ionization states at pH 7 ±2. Docking was performed in Glide with flexible ligands and added Epik penalties. Docking of structures generated by Ligprep was performed in Precision (XP) mode. Crystal complexes were compared using the protein structure alignment in Maestro. Alignments were made with protein backbone atoms and with all heavy atoms.

# ^1^H and ^13^C NMR Spectra


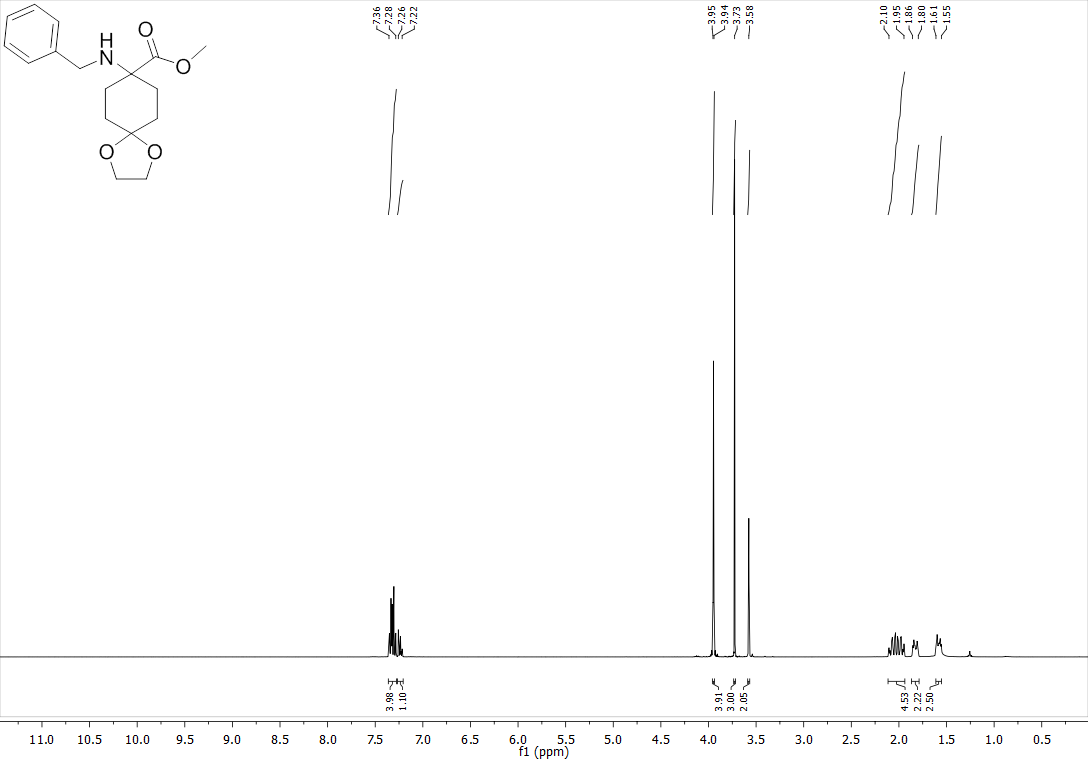


**Figure S6**. ^1^H NMR spectrum of compound **2**.


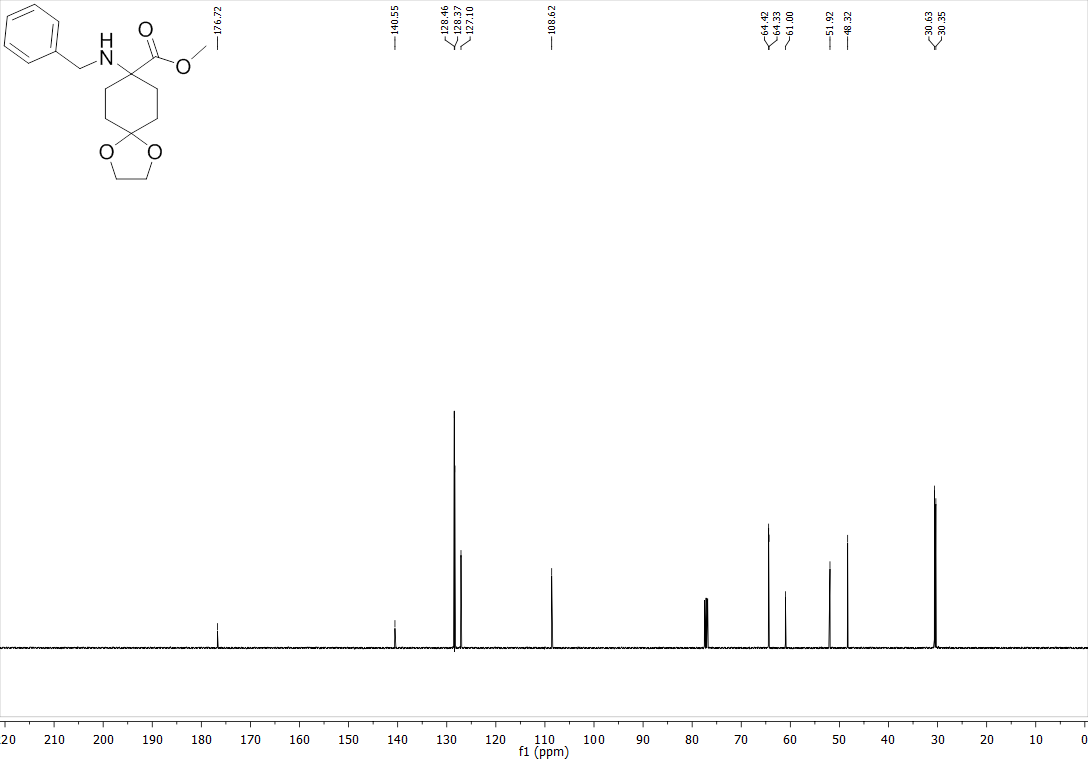


**Figure S7**. ^13^C NMR spectrum of compound **2**.


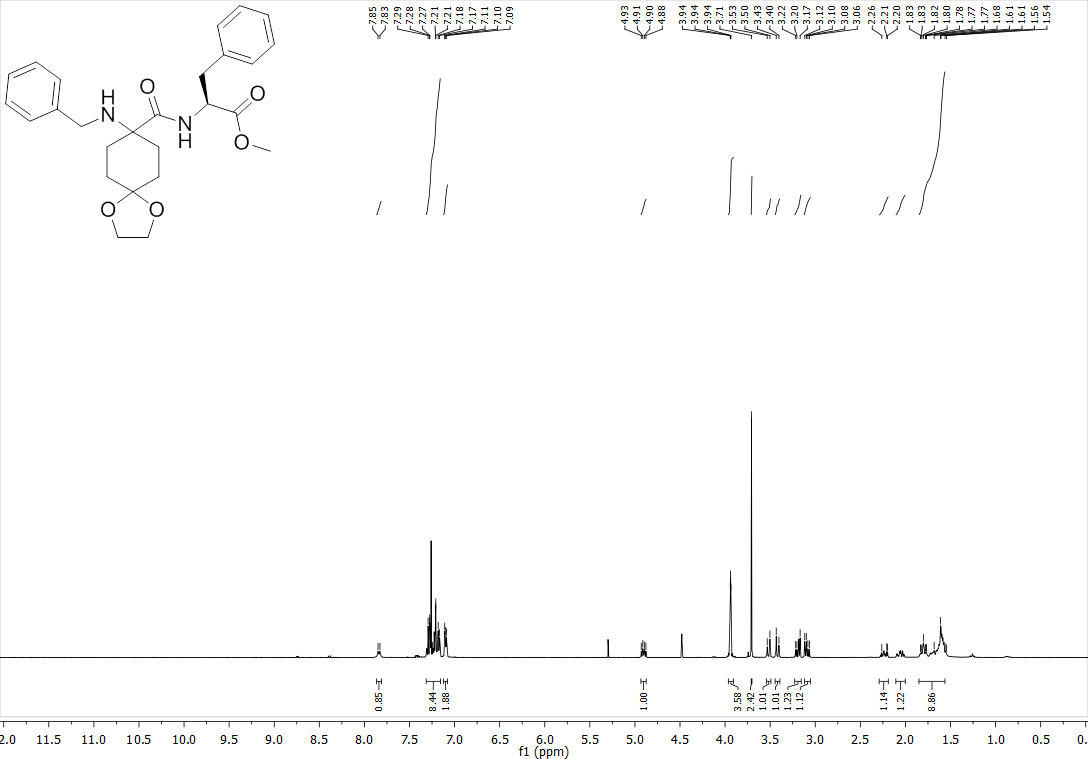


**Figure S8**. ^1^H NMR spectrum of compound **4**.


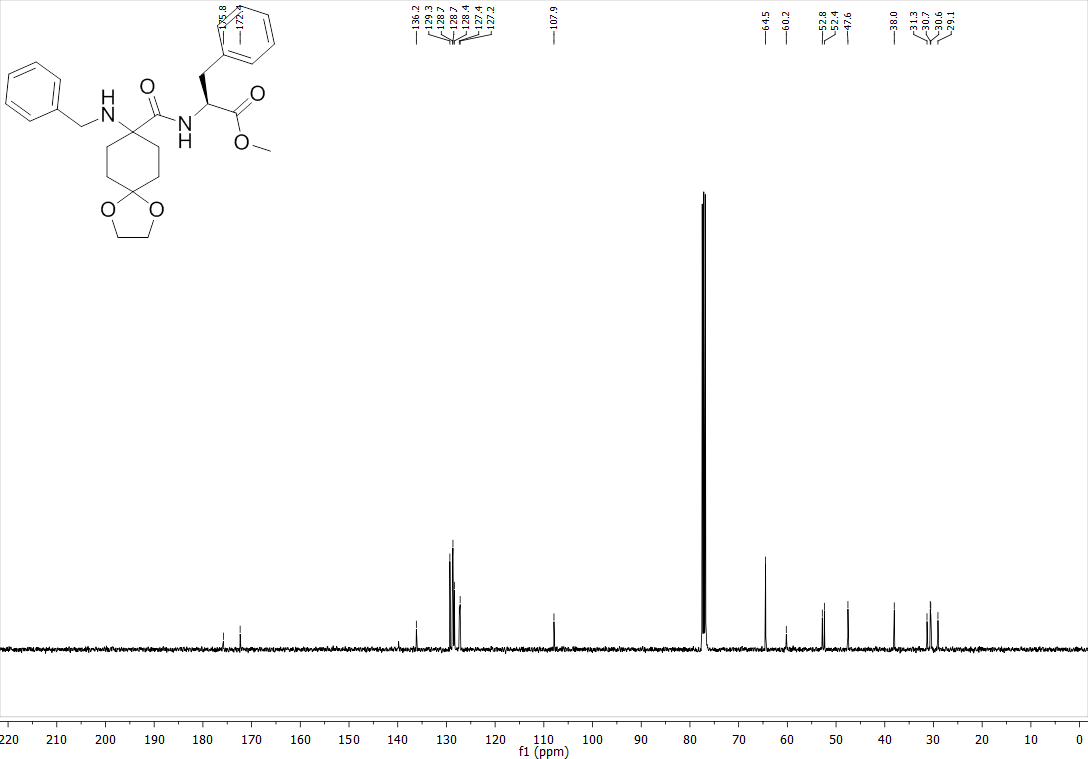


**Figure S9**. ^13^C NMR spectrum of compound **4**.


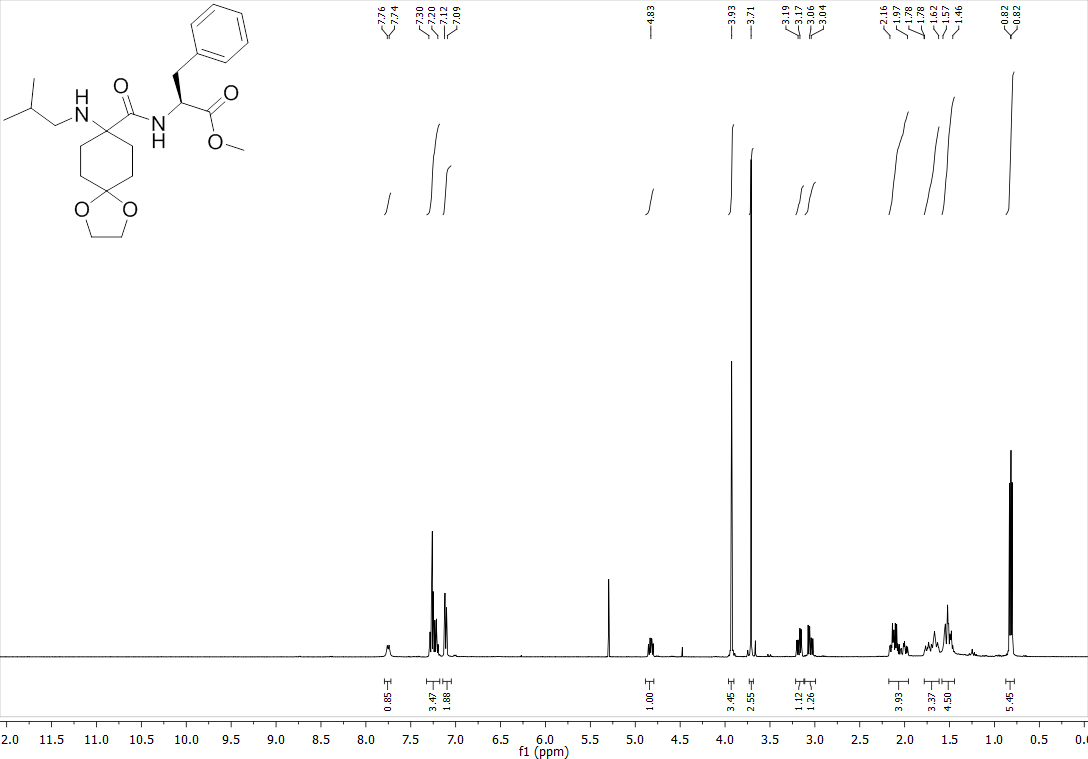


**Figure S10**. ^1^H NMR spectrum of compound **5**.


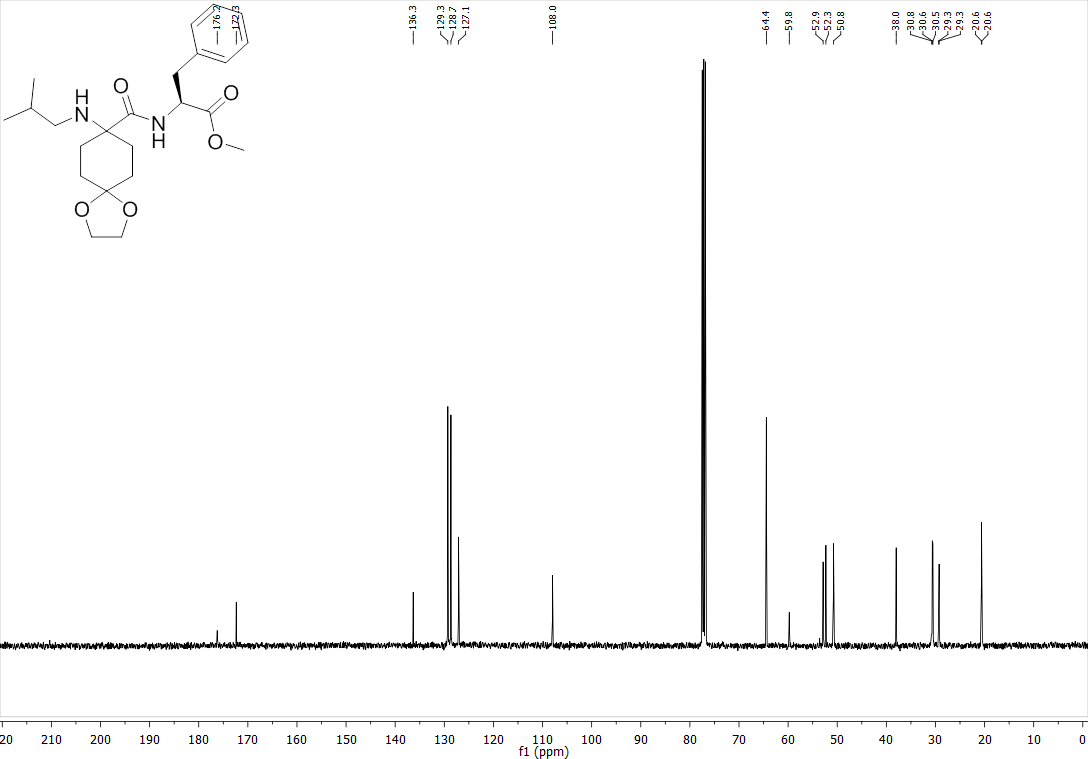


**Figure S11**. ^13^C NMR spectrum of compound **5**.


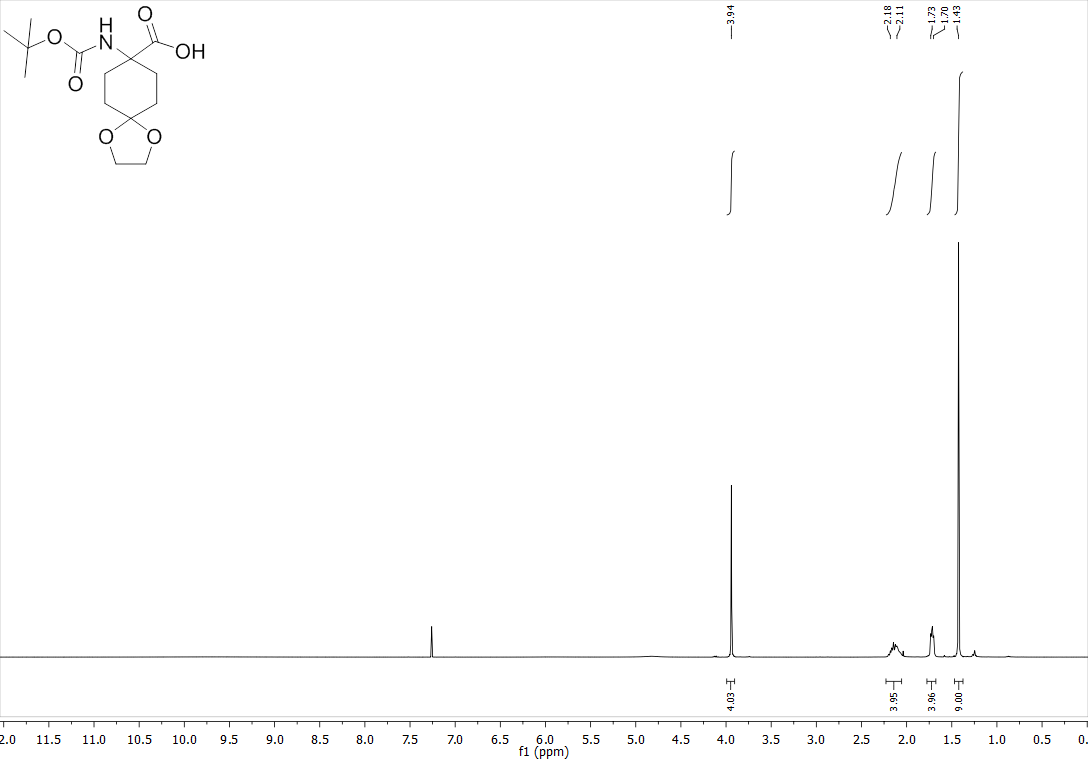


**Figure S12**. ^1^H NMR spectrum of compound 8-{[(*tert*-Butoxy)carbonyl]amino}-1,4-dioxaspiro[4.5]decane-8-carboxylic acid.


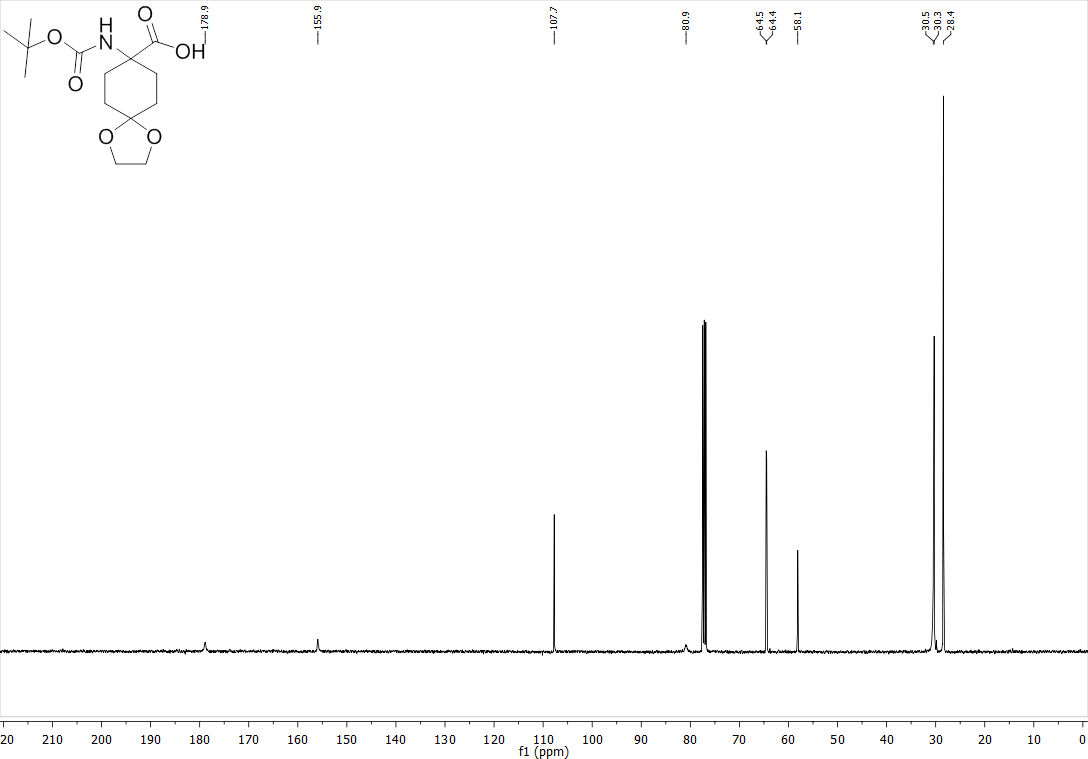


**Figure S13**. ^13^C NMR spectrum of compound 8-{[(*tert*-Butoxy)carbonyl]amino}-1,4-dioxaspiro[4.5]decane-8-carboxylic acid.


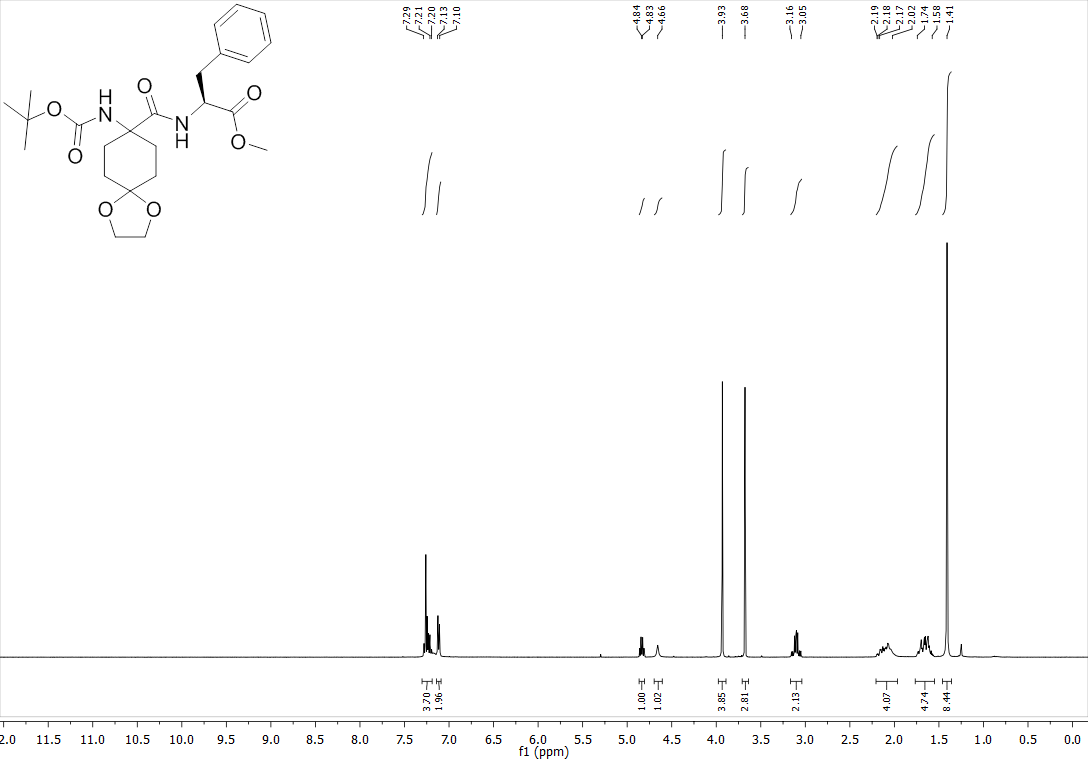


**Figure S14**. ^1^H NMR spectrum of compound **6**.


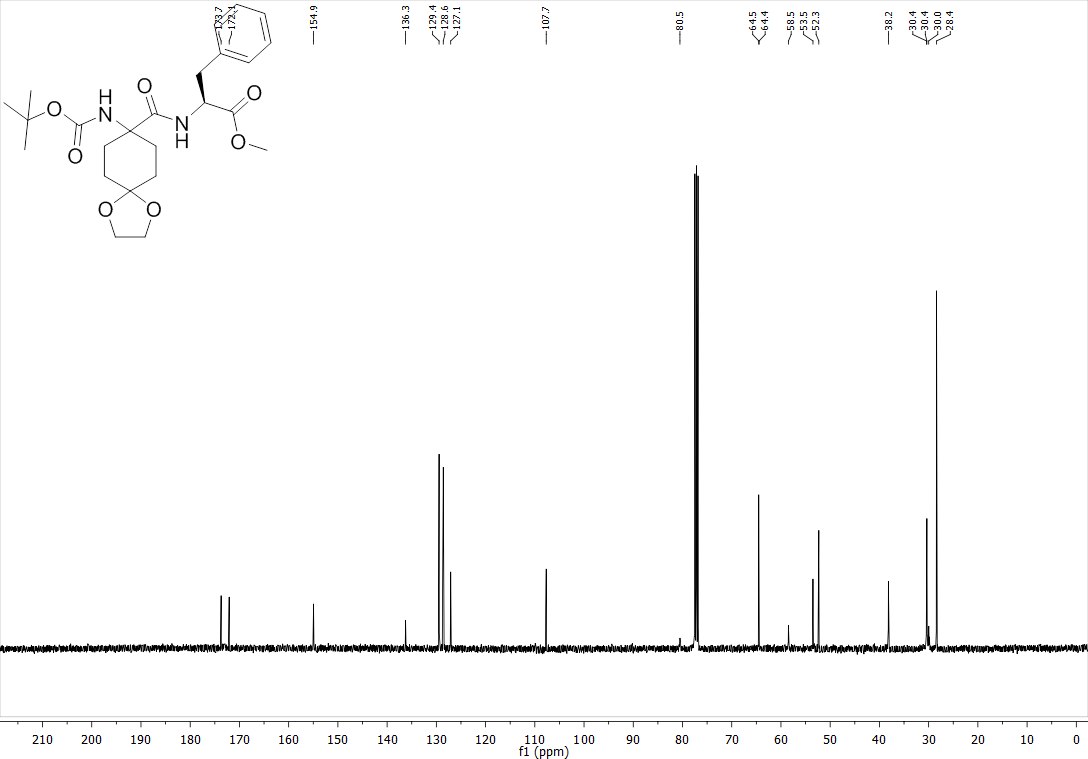


**Figure S15**. ^13^C NMR spectrum of compound **6**.


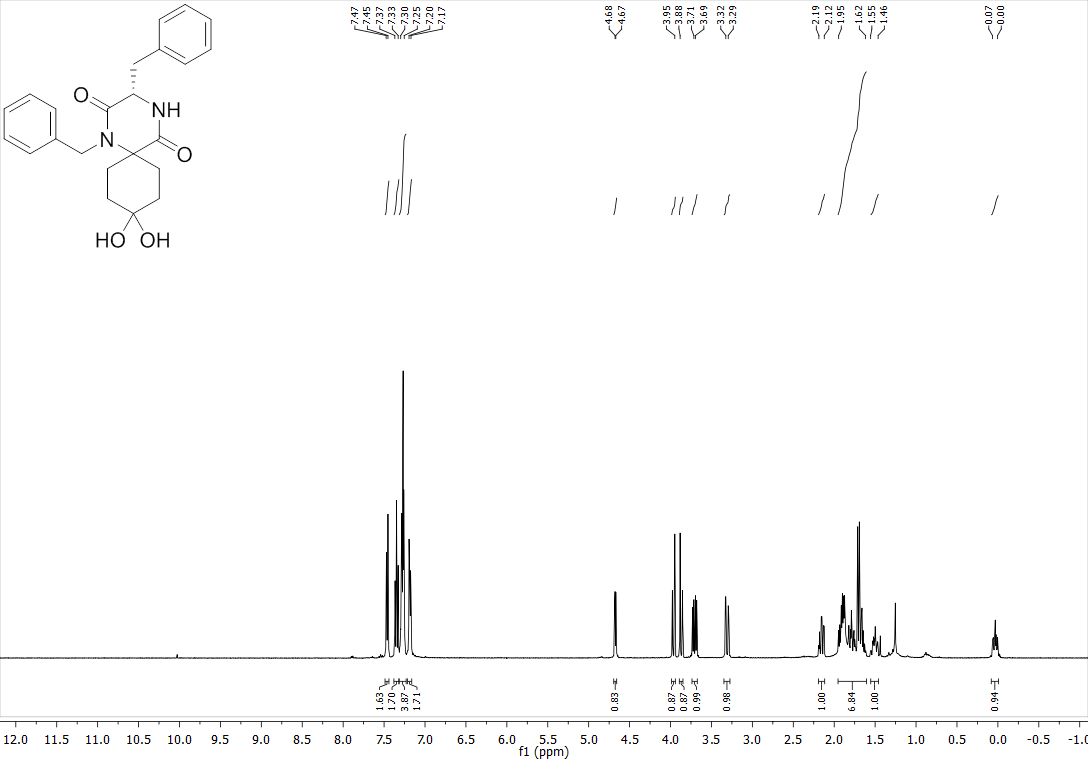


**Figure S16**. ^1^H NMR spectrum of compound **7**.


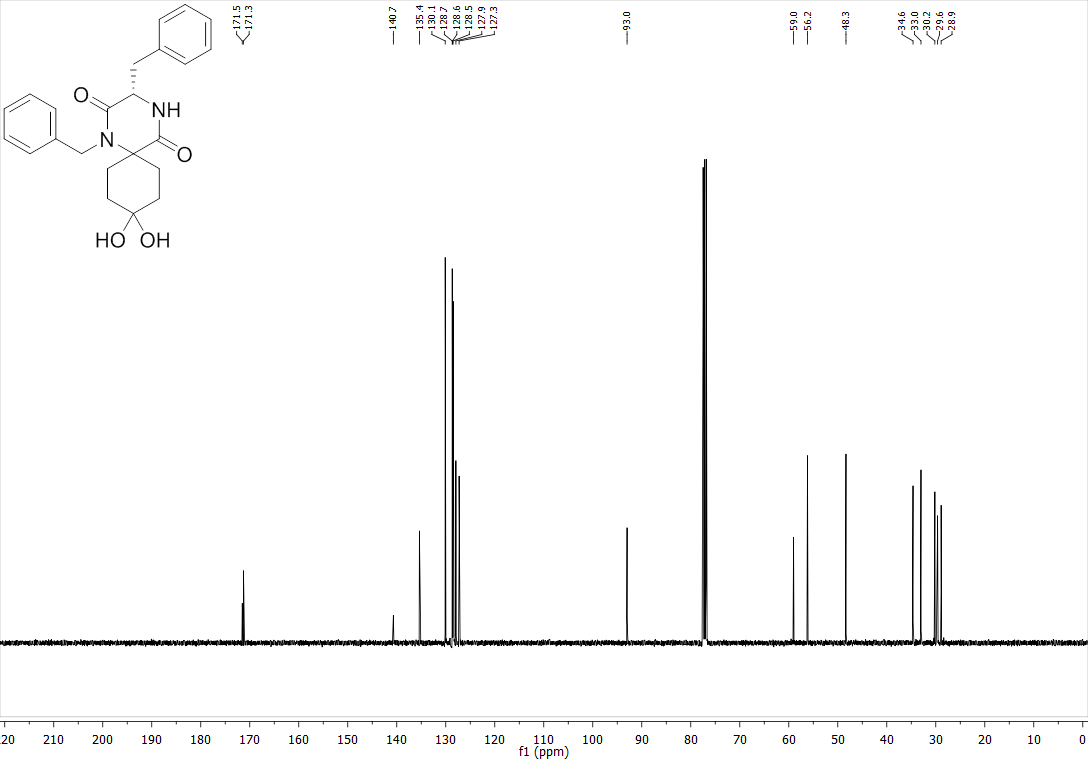


**Figure S17**. ^13^C NMR spectrum of compound **7**.


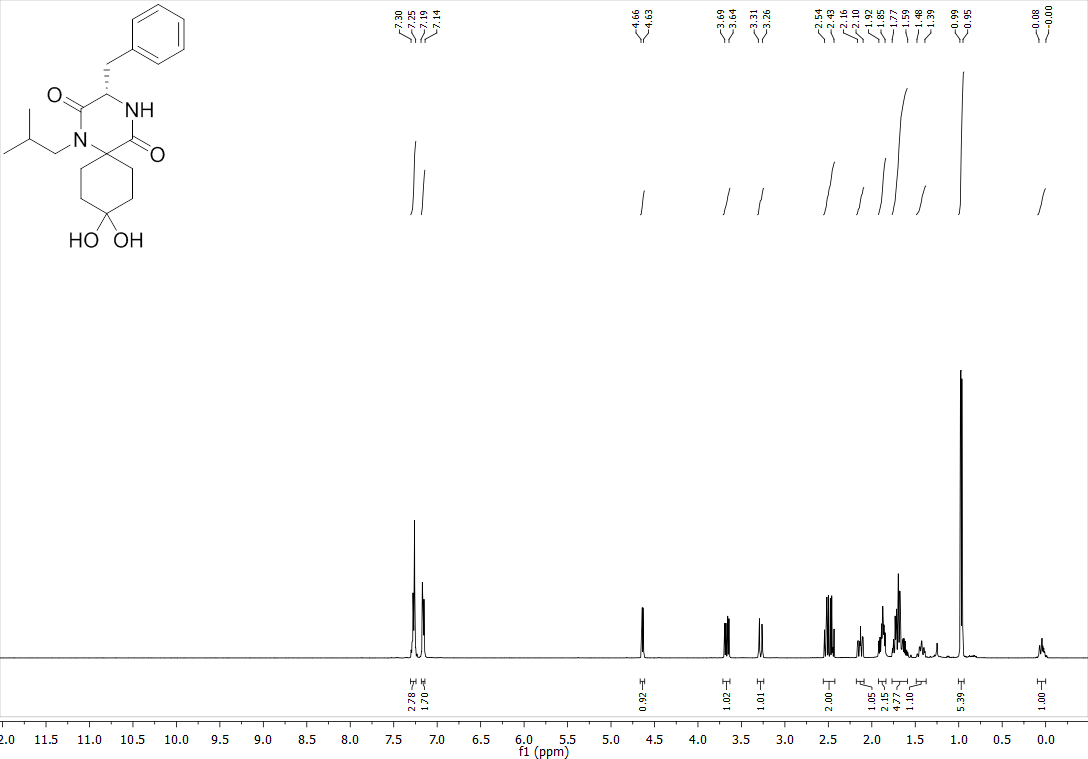


**Figure S18**. ^1^H NMR spectrum of compound **8**.


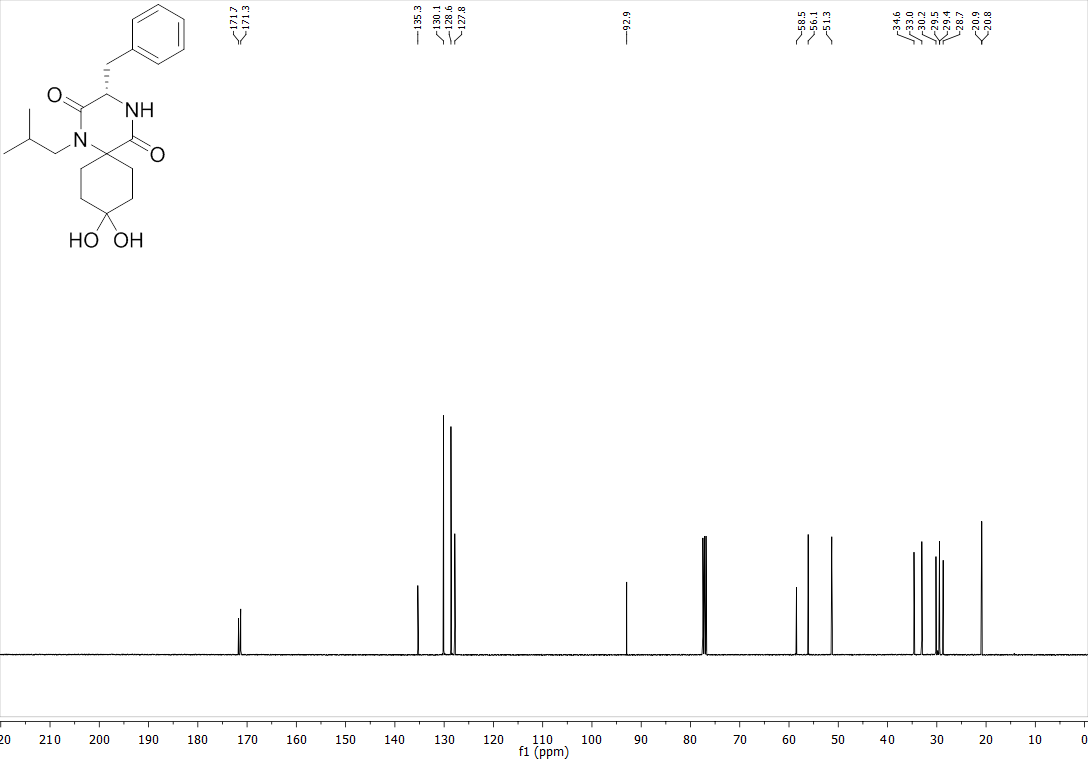


**Figure S19**. ^13^C NMR spectrum of compound **8**.


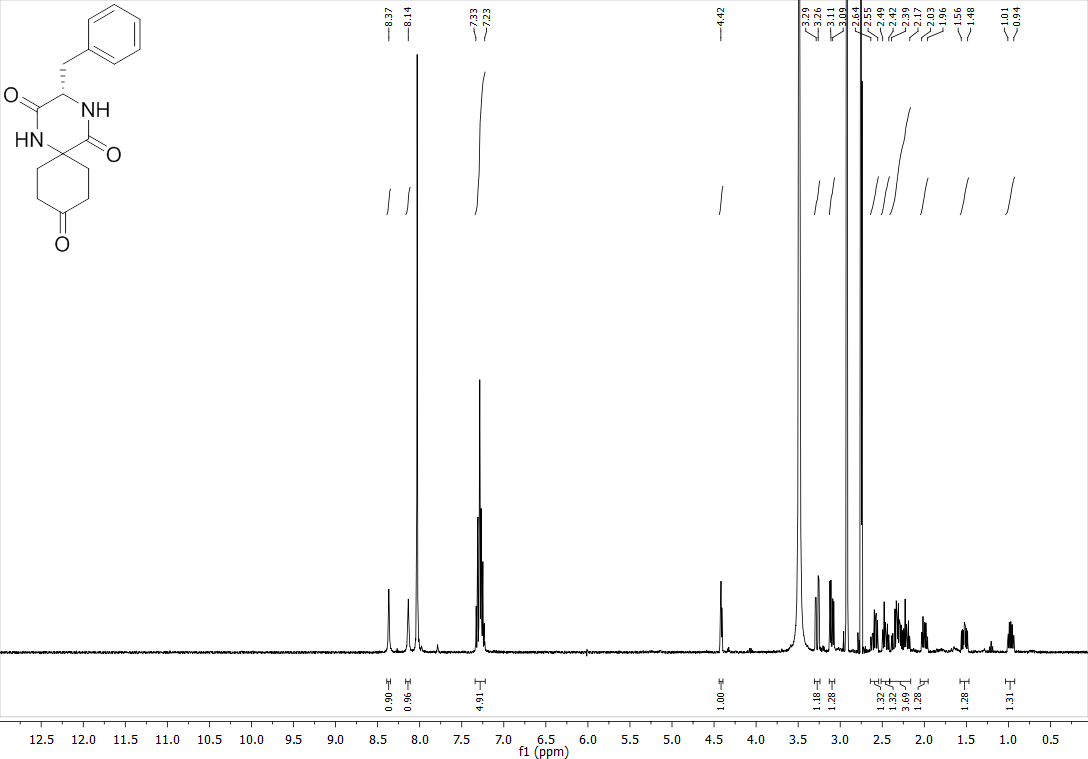


**Figure S20**. ^1^H NMR spectrum of compound **9**.


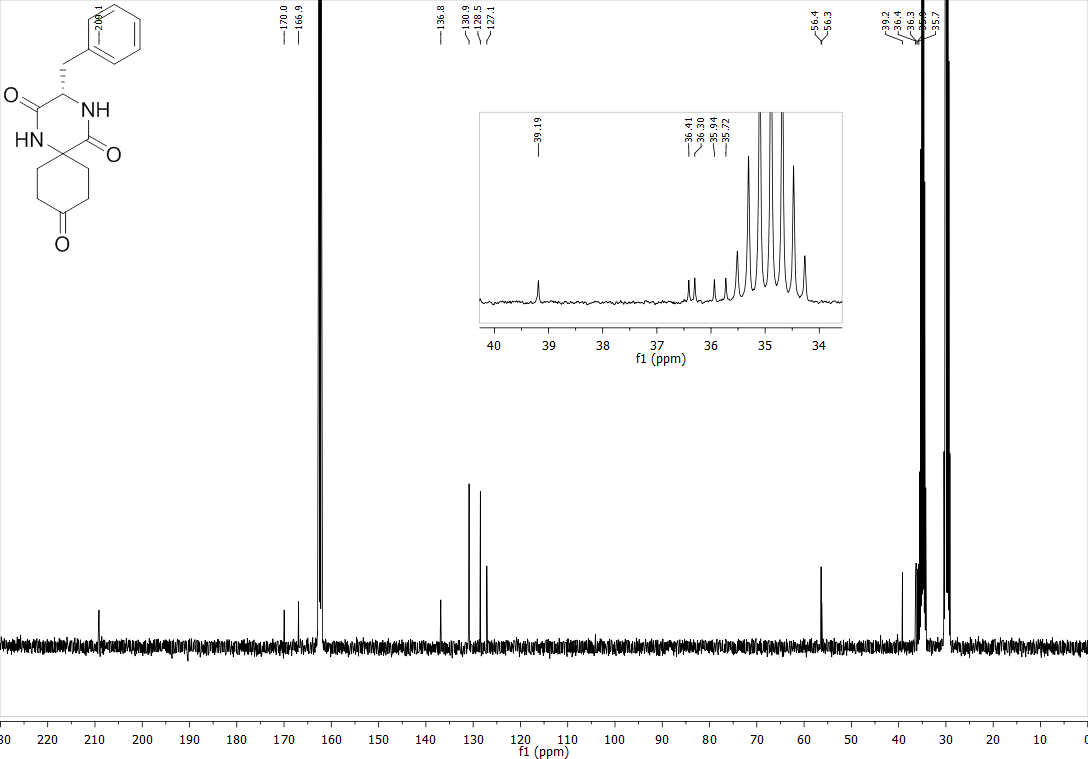


**Figure S21**. ^13^C NMR spectrum of compound **9**.

**
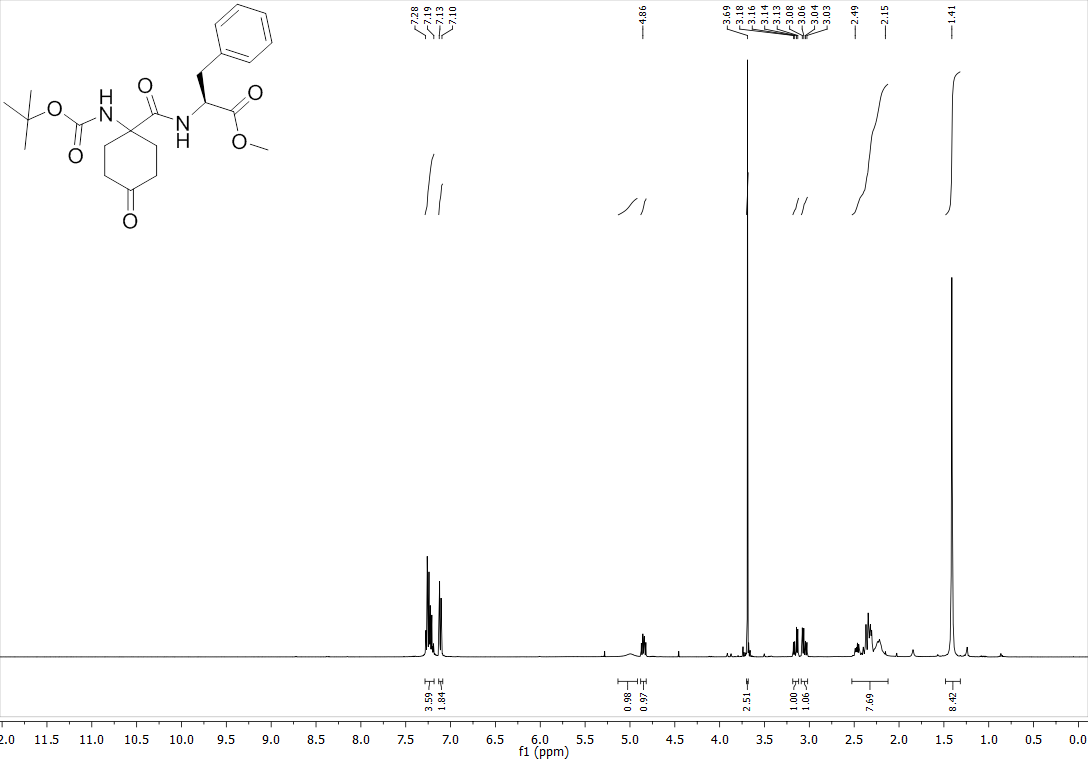
**

**Figure S22** ^1^H NMR spectrum of compound **11**.

**
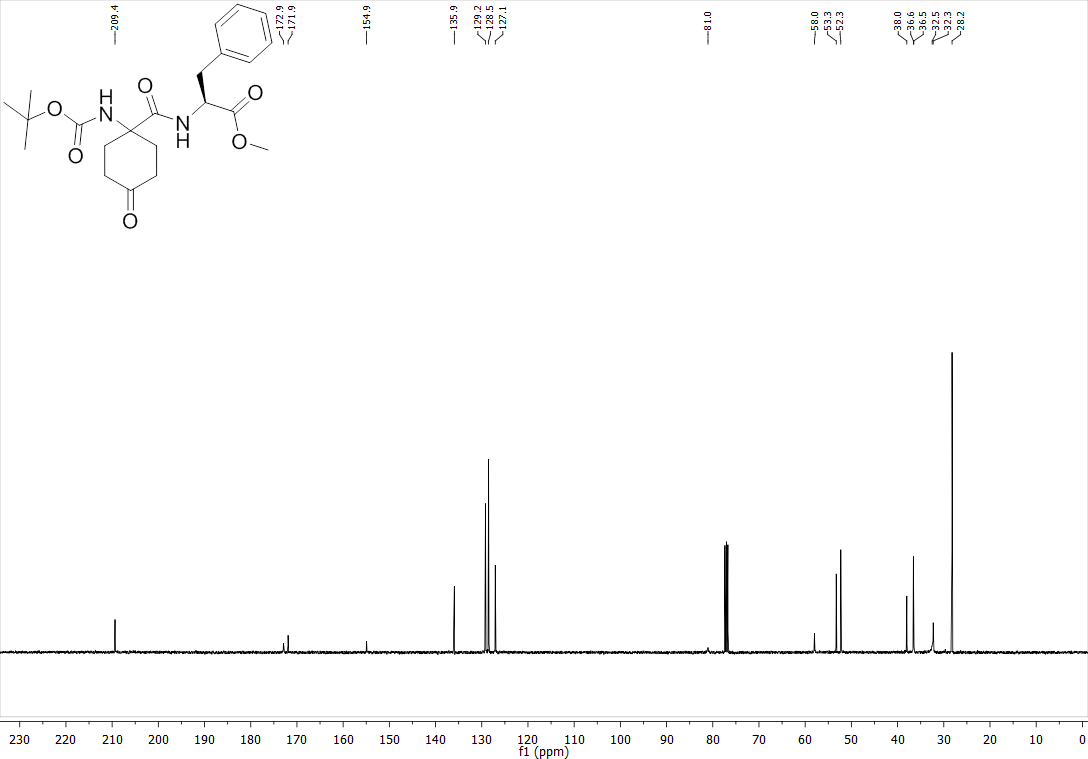
**

**Figure S23**. ^13^C NMR spectrum of compound **11**.


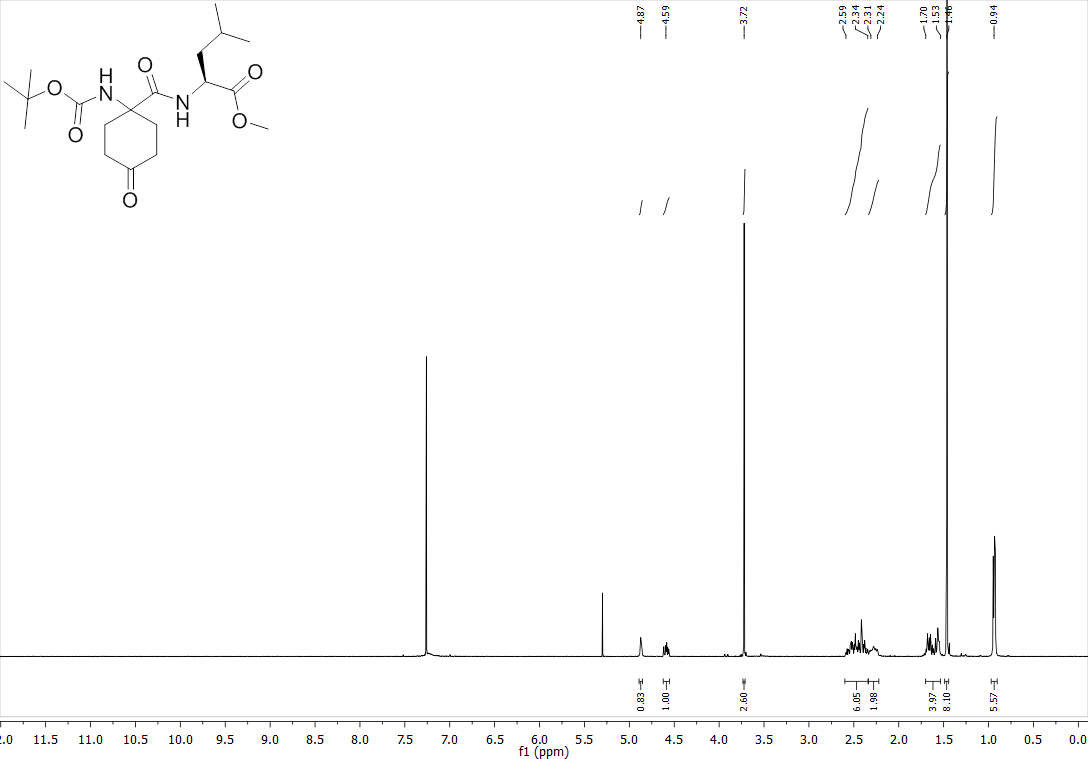


**Figure S24**. ^1^H NMR spectrum of compound **12**.


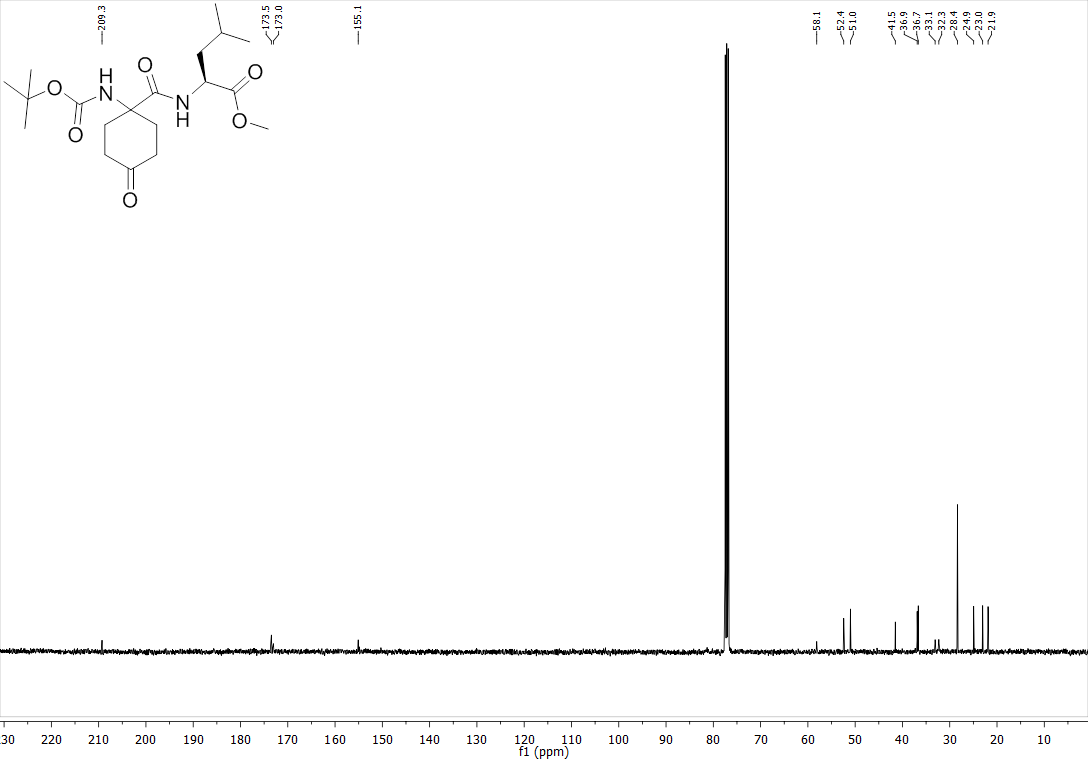


**Figure S25**. ^13^C NMR spectrum of compound **12**.


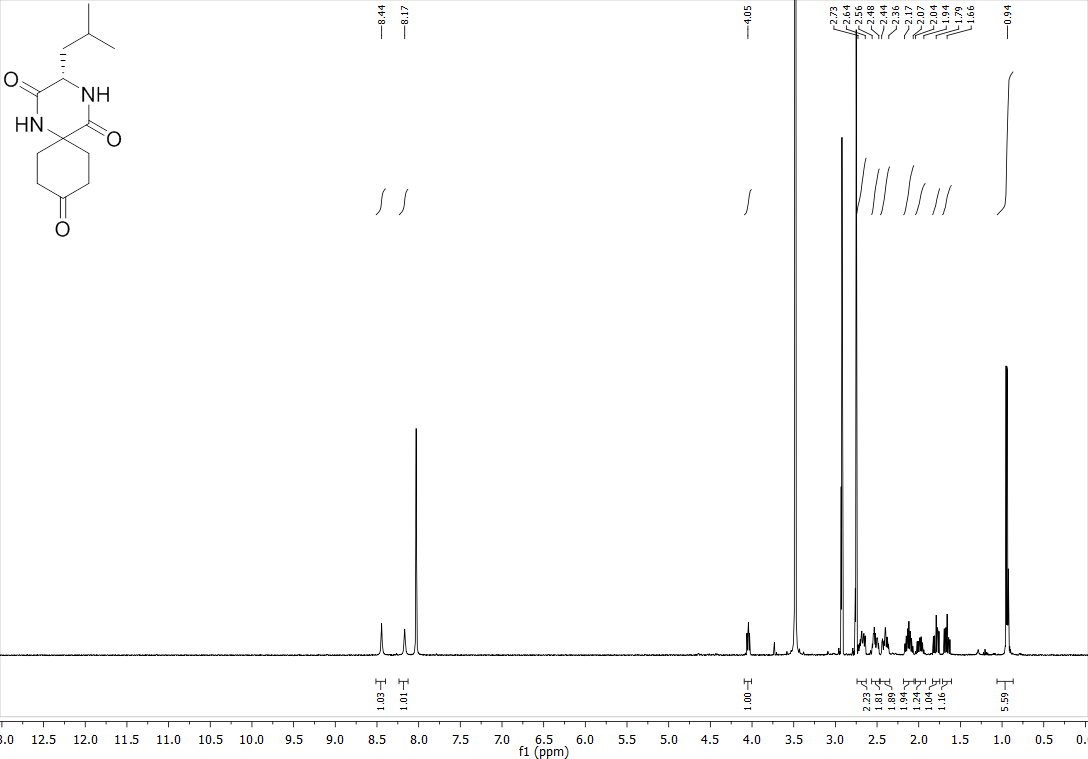


**Figure S26**. ^1^H NMR spectrum of compound **13**.

**
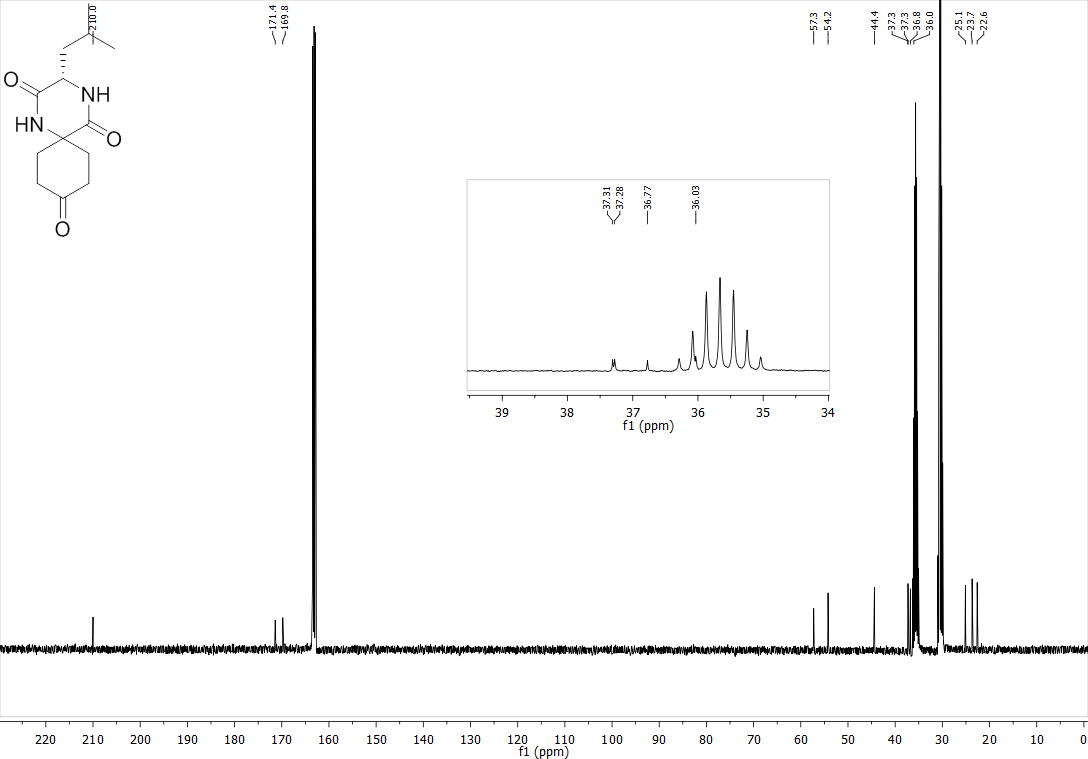
**

**Figure S27**. ^13^C NMR spectrum of compound **13**.


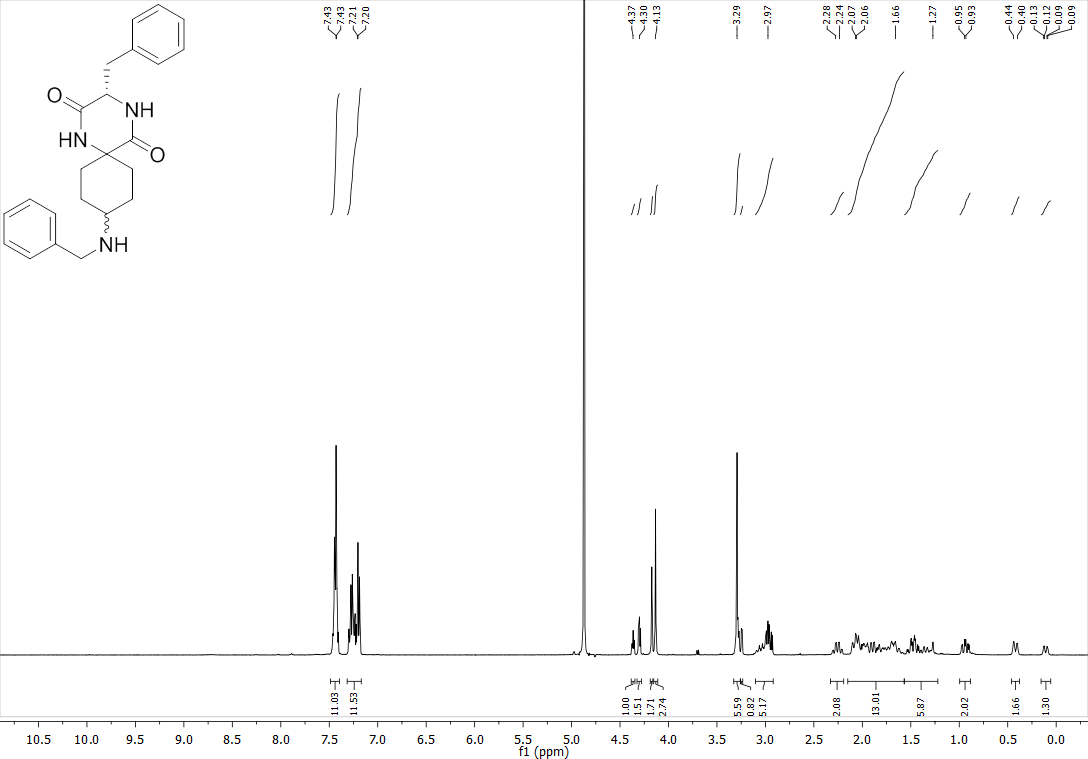


**Figure S28**. ^1^H NMR spectrum of compound **14**.


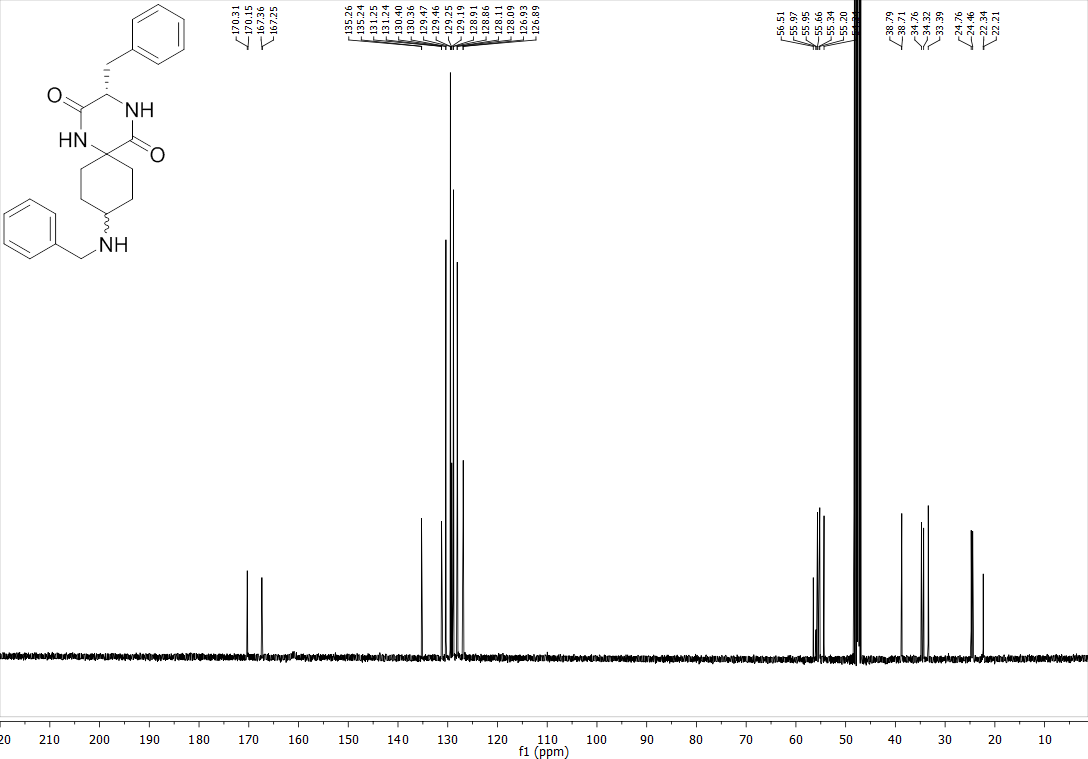


**Figure S29**. ^13^C NMR spectrum of compound **14**.


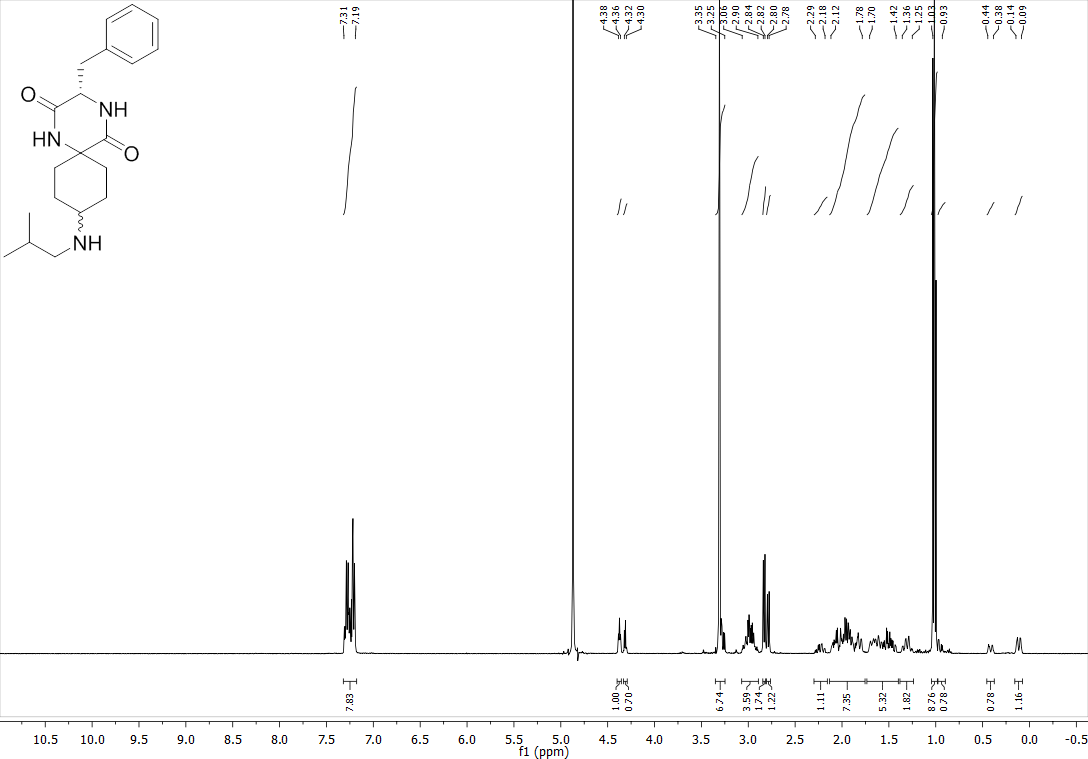


**Figure S30**. ^1^H NMR spectrum of compound **15**.


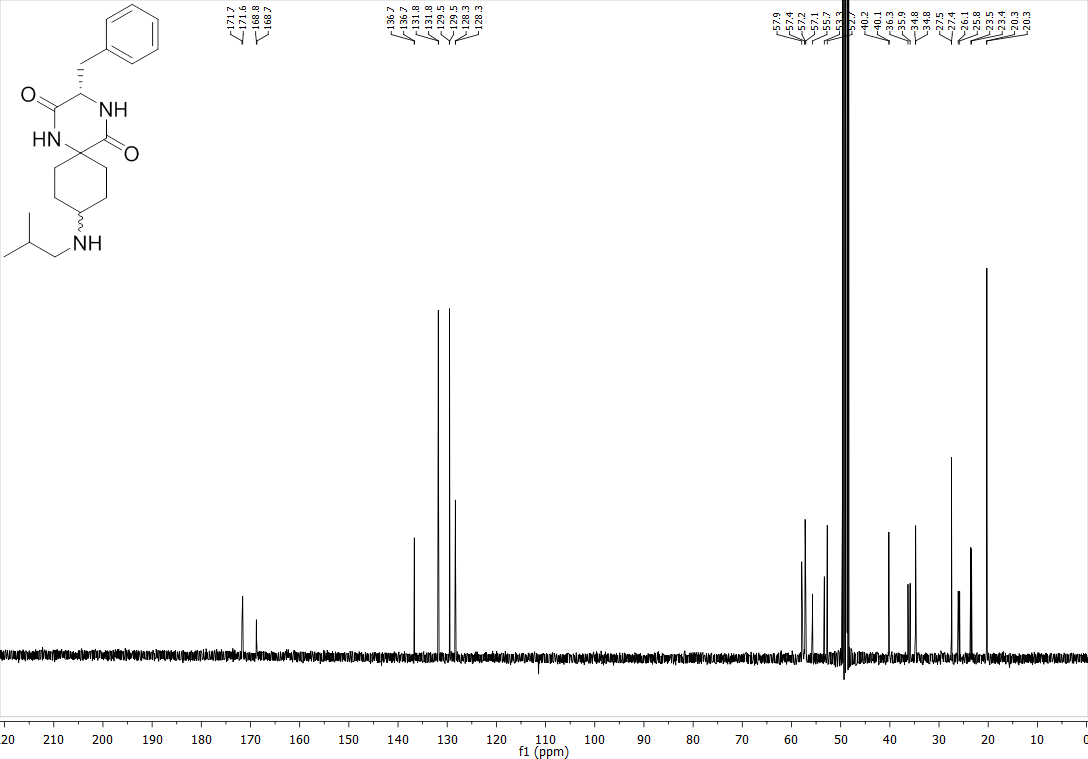


**Figure S31**. ^13^C NMR spectrum of compound **15**.


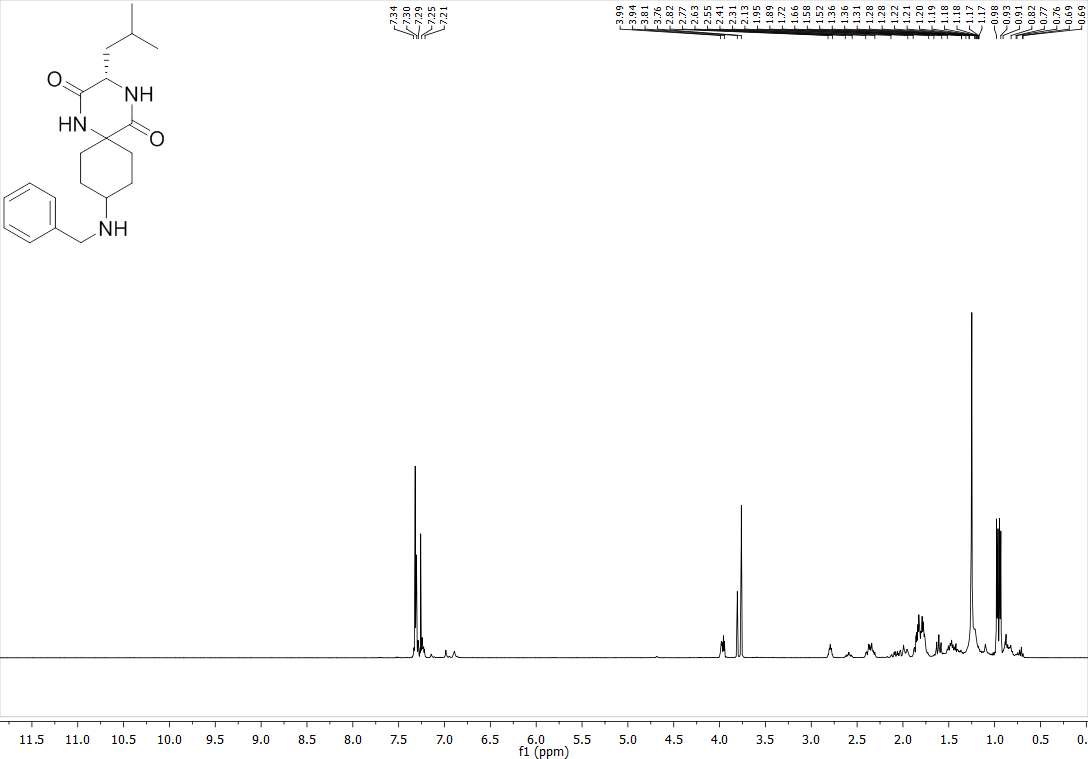


**Figure S32**. ^1^H NMR spectrum of compound **16**.


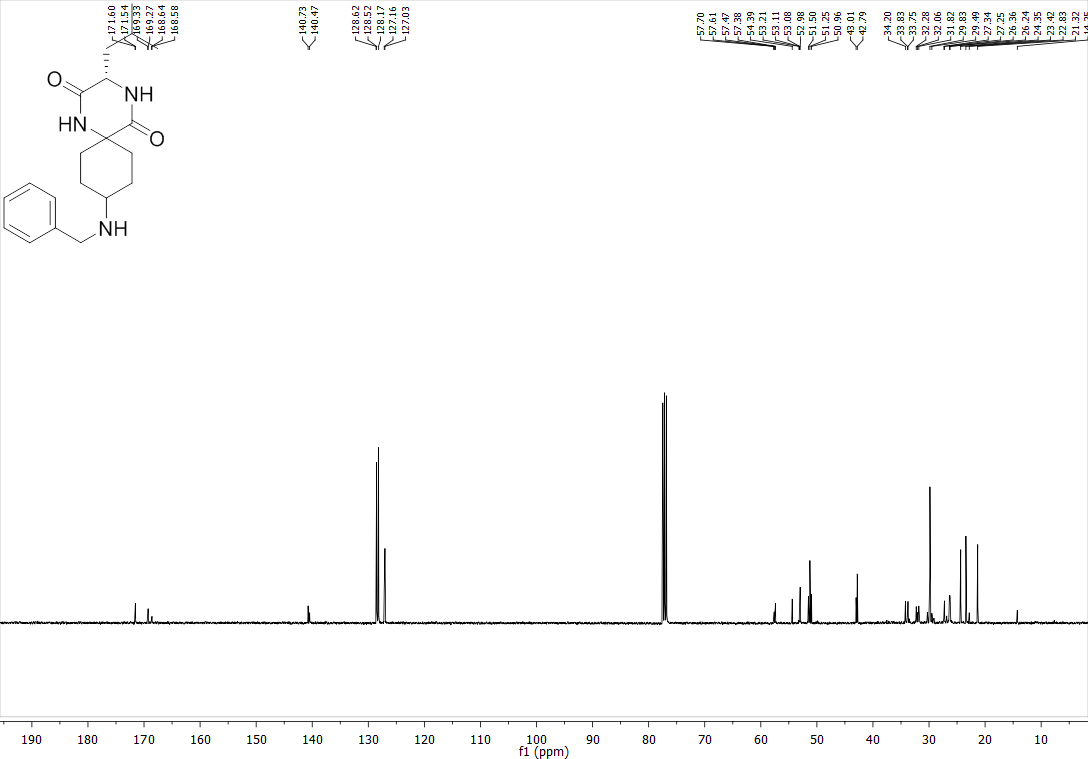


**Figure S33**. ^13^C NMR spectrum of compound **16**.


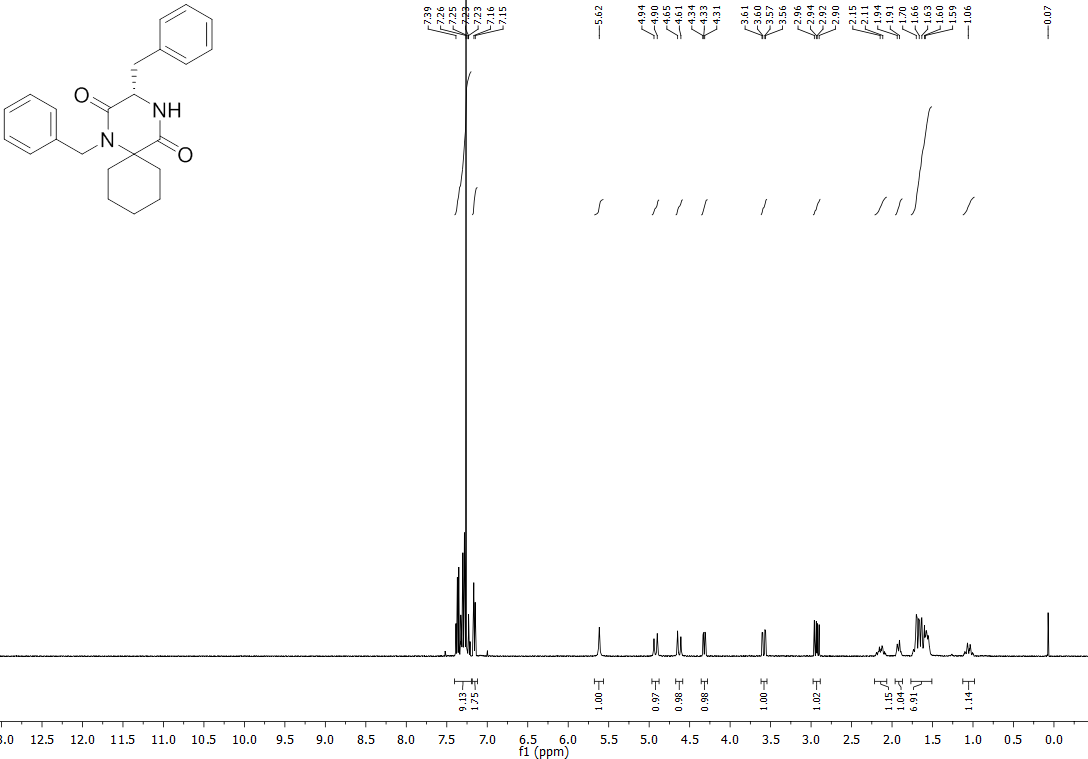


**Figure S34.** ^1^H NMR spectrum of compound **17S**.


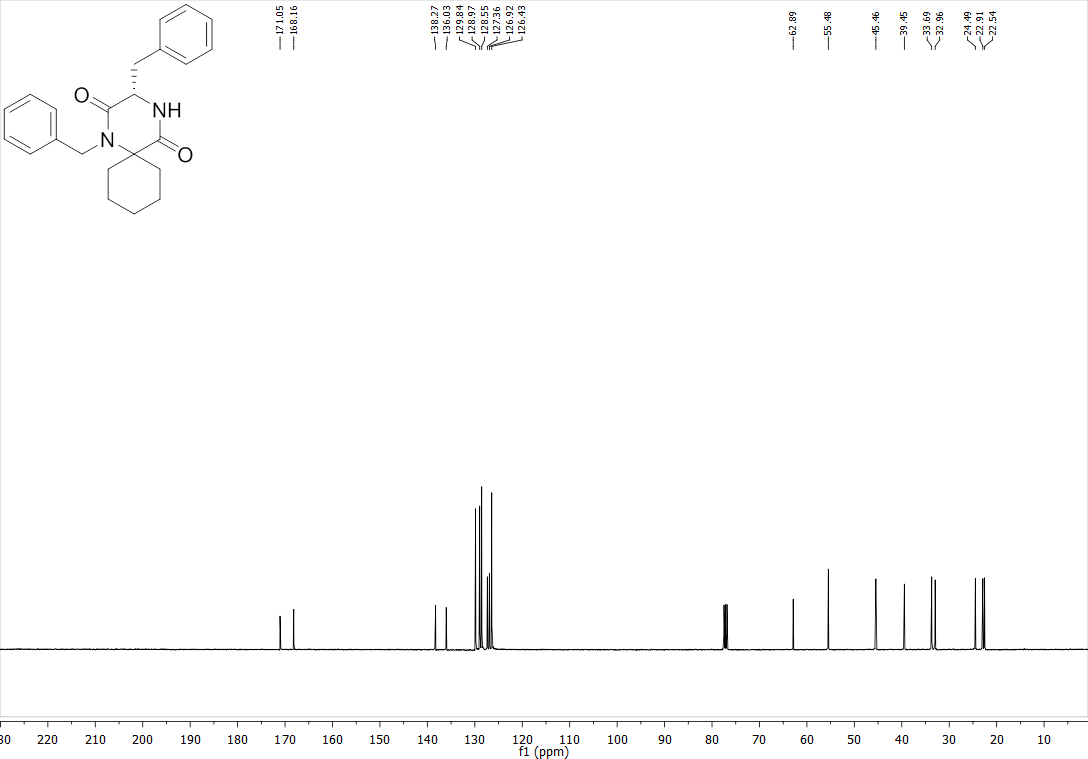


**Figure S35**. ^13^C NMR spectrum of compound **17S**.

**
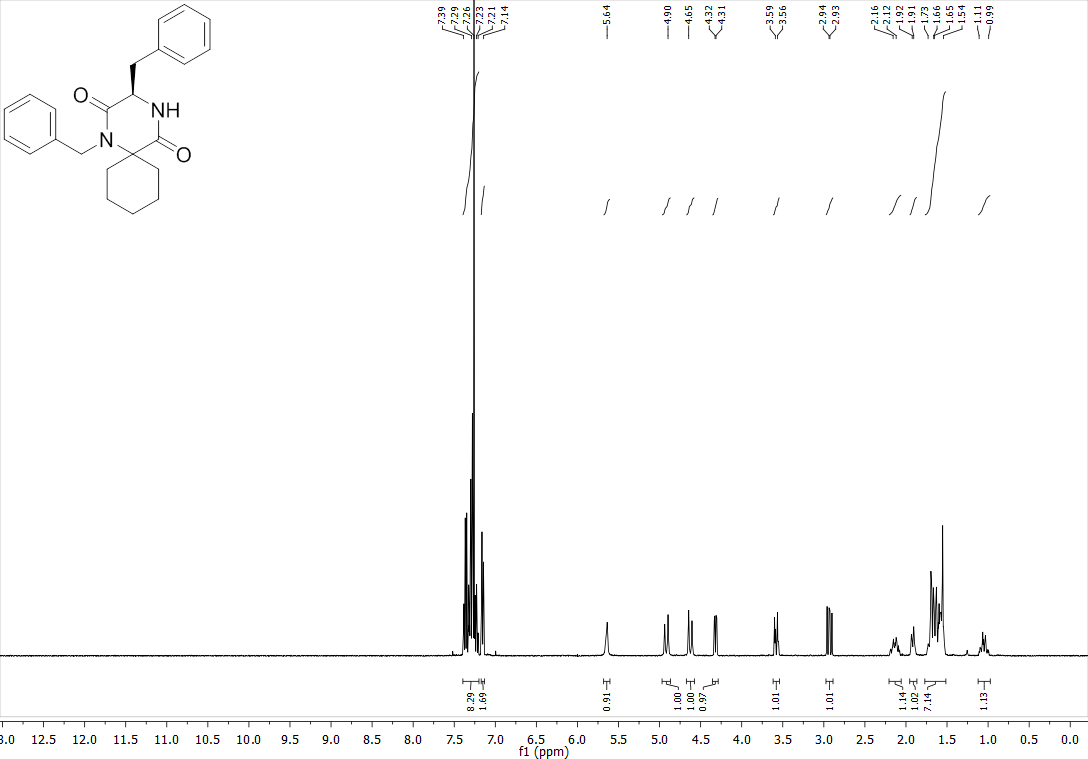
**

**Figure S36.** ^1^H NMR spectrum of compound **17R**.

**
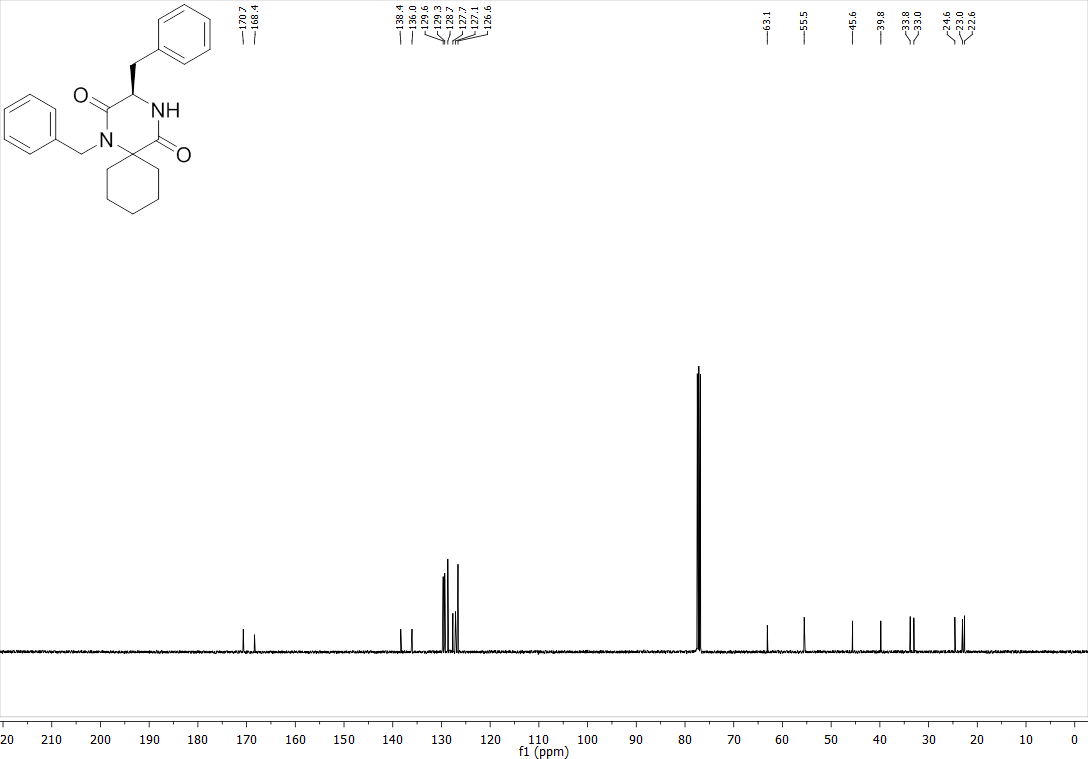
**

**Figure S37**. ^13^C NMR spectrum of compound **17R**.


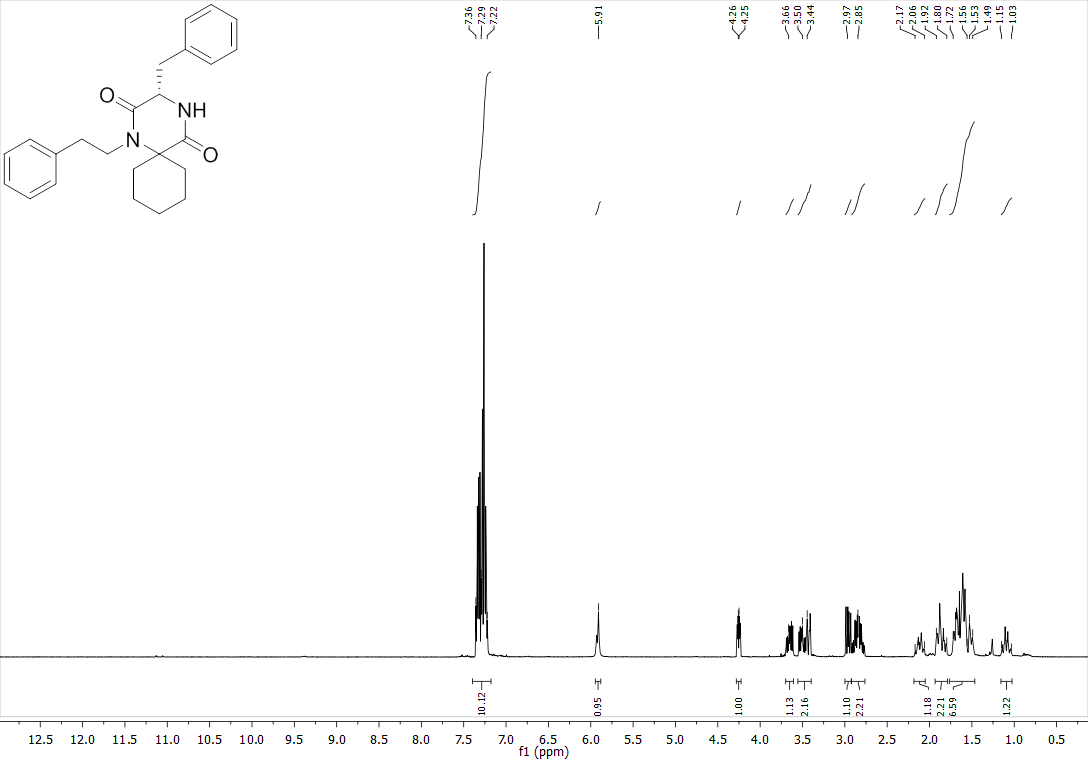


**Figure S38.** ^1^H NMR spectrum of compound **18S**.


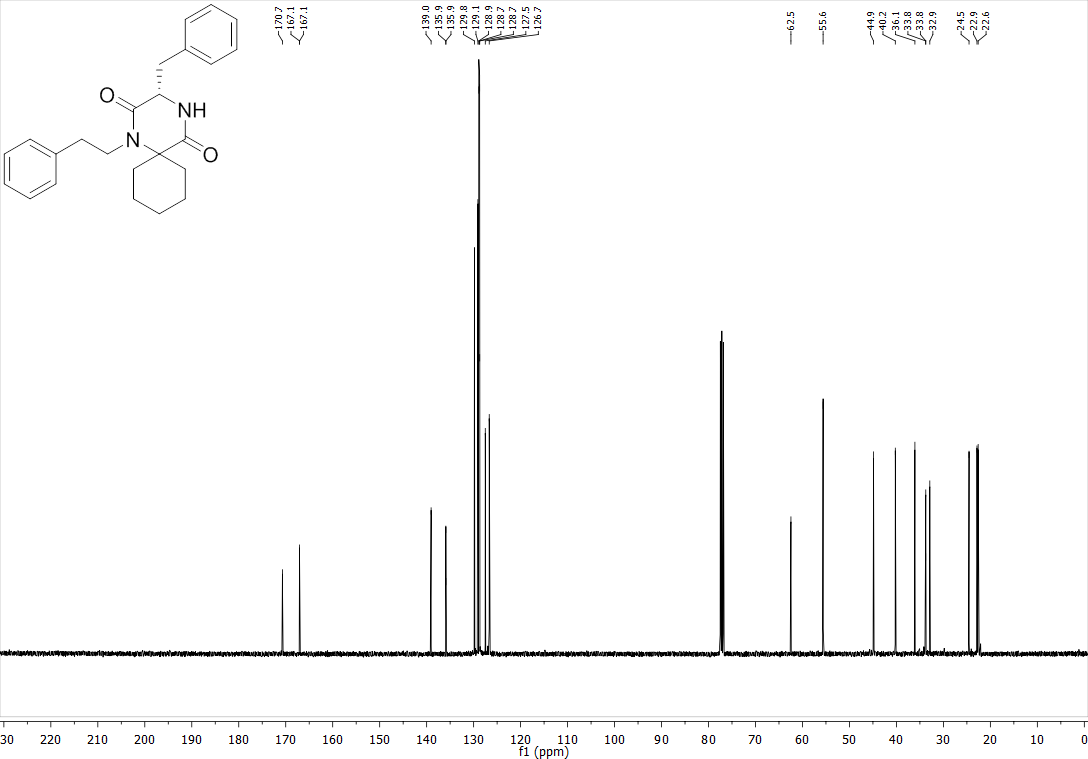


**Figure S39**. ^13^C NMR spectrum of compound **18S**.


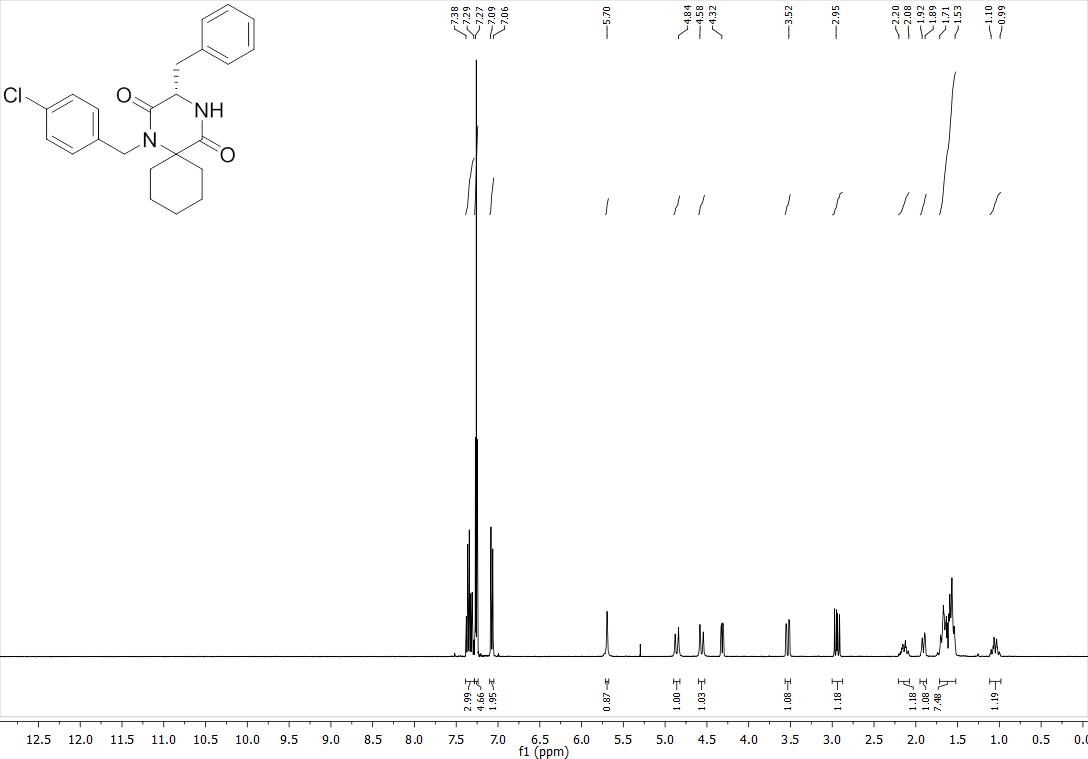


**Figure S40.** ^1^H NMR spectrum of compound **19S**.


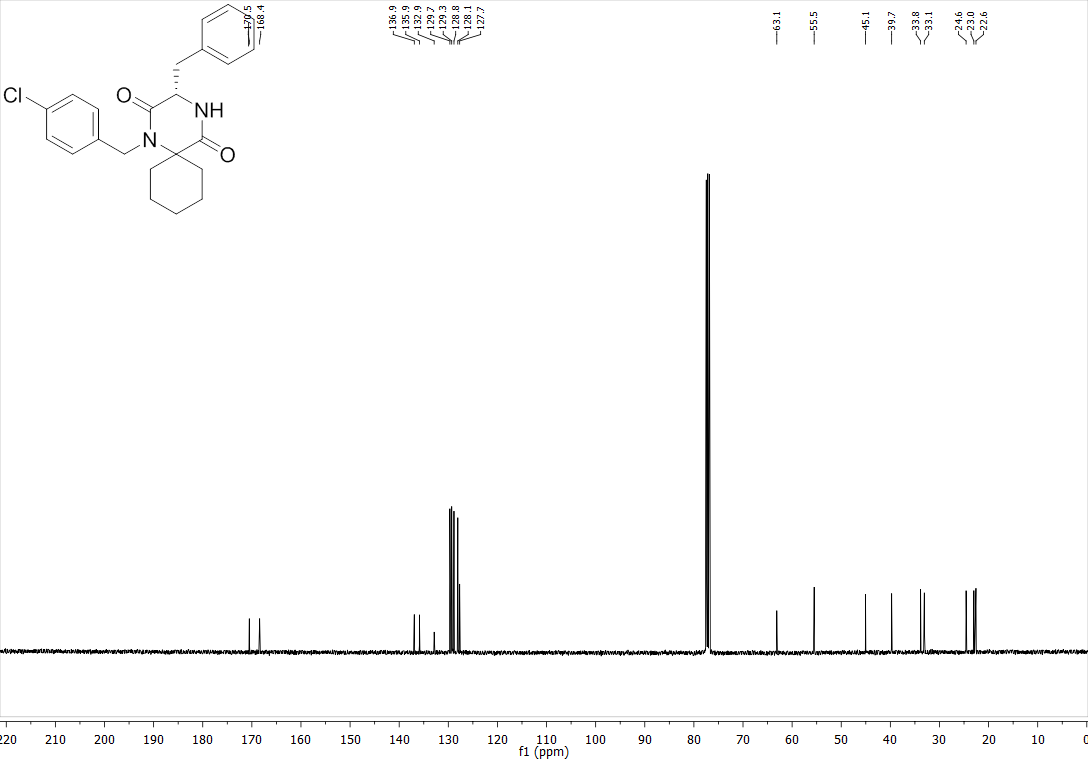


**Figure S41**. ^13^C NMR spectrum of compound **19S**.


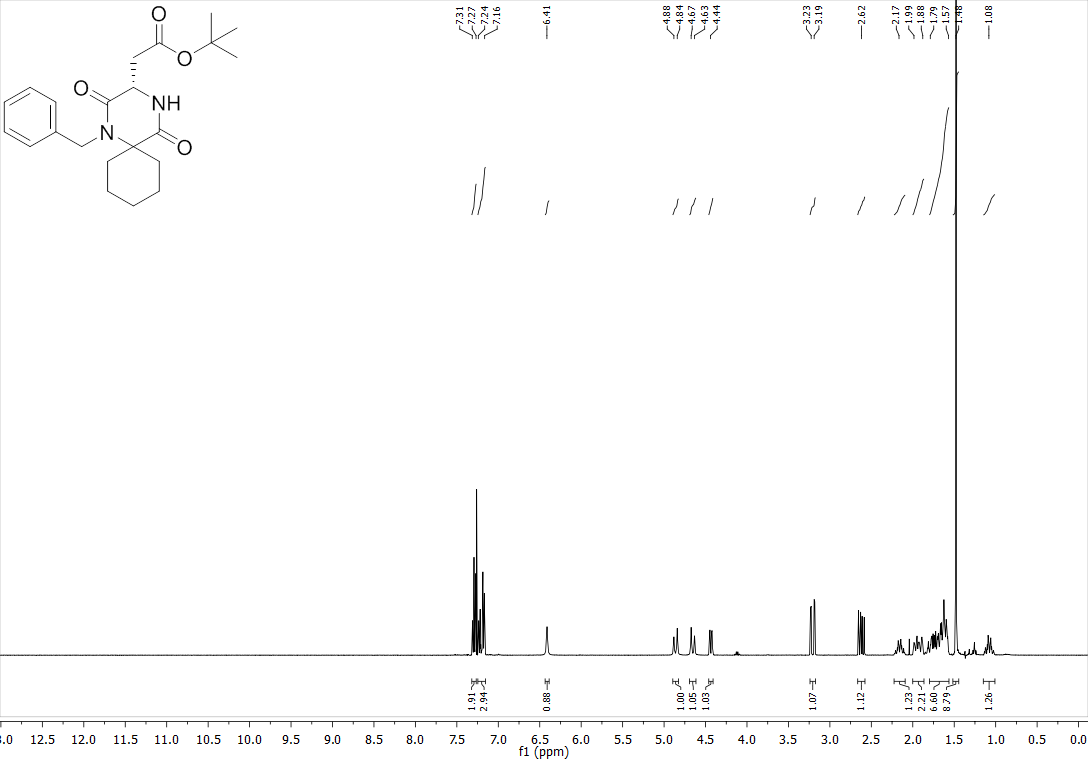


**Figure S42.** ^1^H NMR spectrum of compound **20S**.


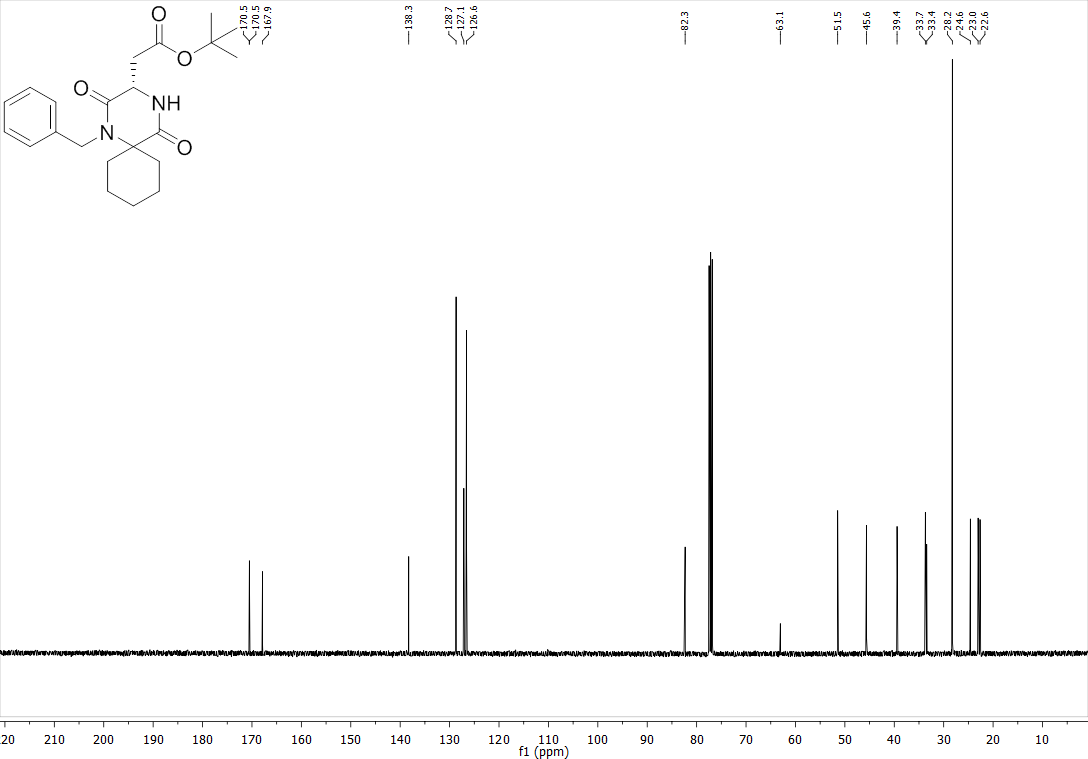


**Figure S43**. ^13^C NMR spectrum of compound **20S**.


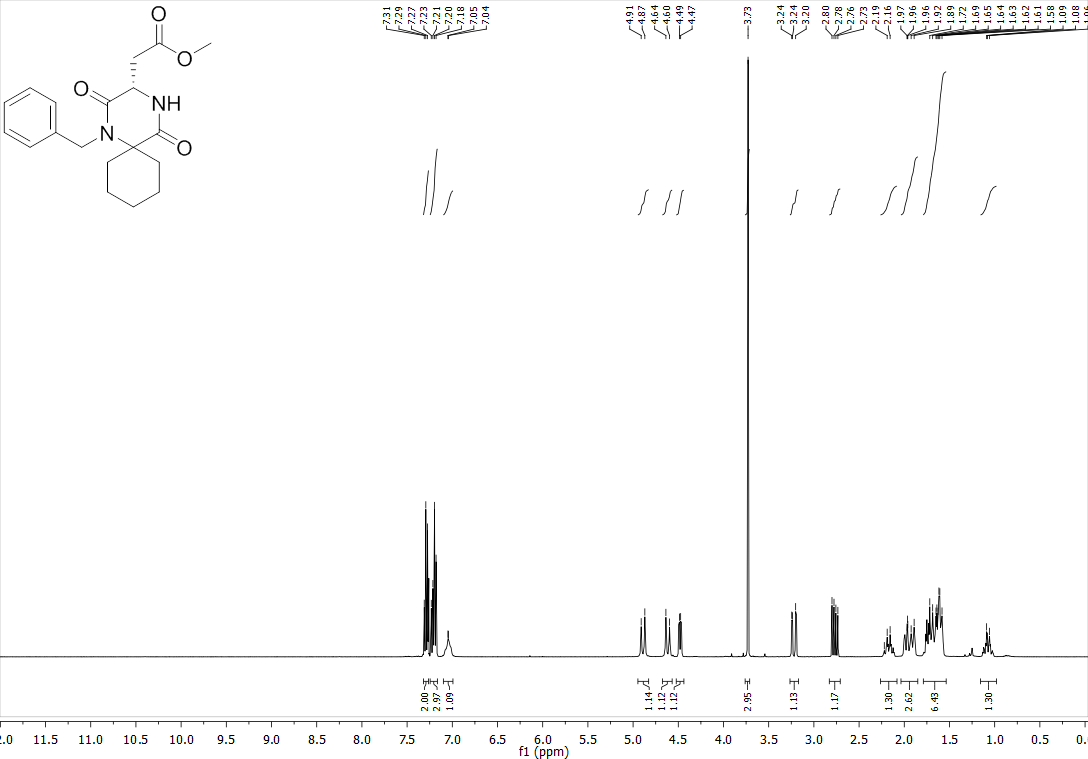


**Figure S44.** ^1^H NMR spectrum of compound **21S**.


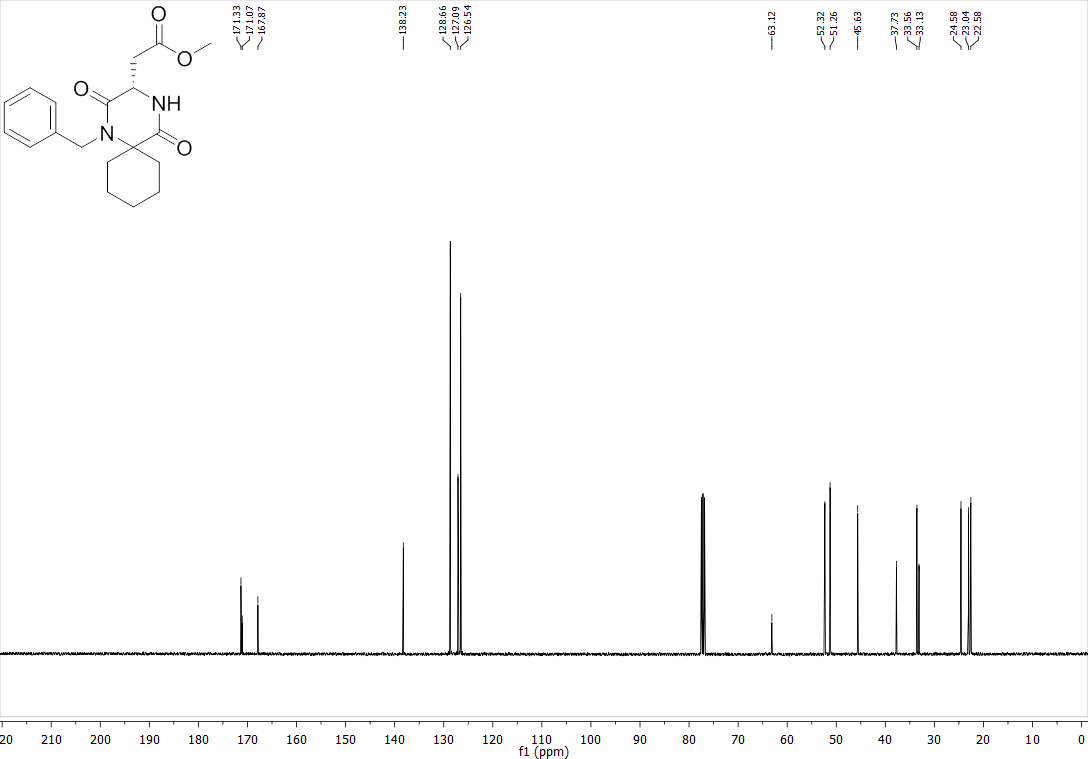


**Figure S45**. ^13^C NMR spectrum of compound **21S**.

**
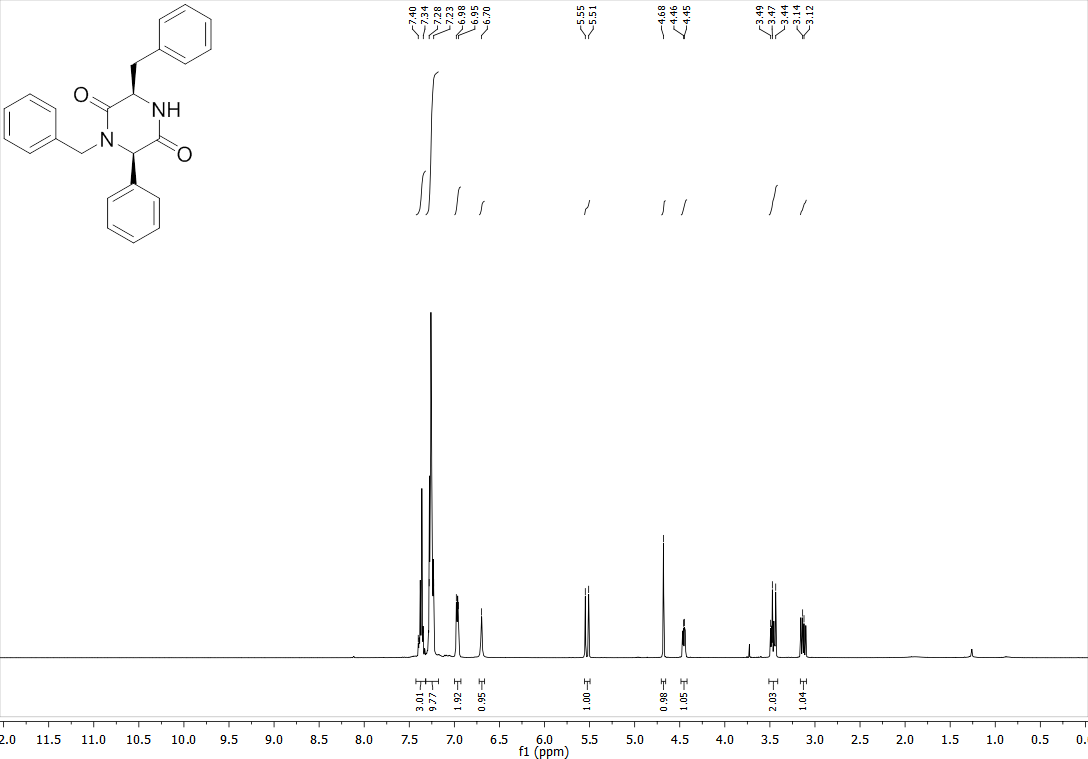
**

**Figure S46**. ^1^H NMR spectrum of compound **22RR**.

**
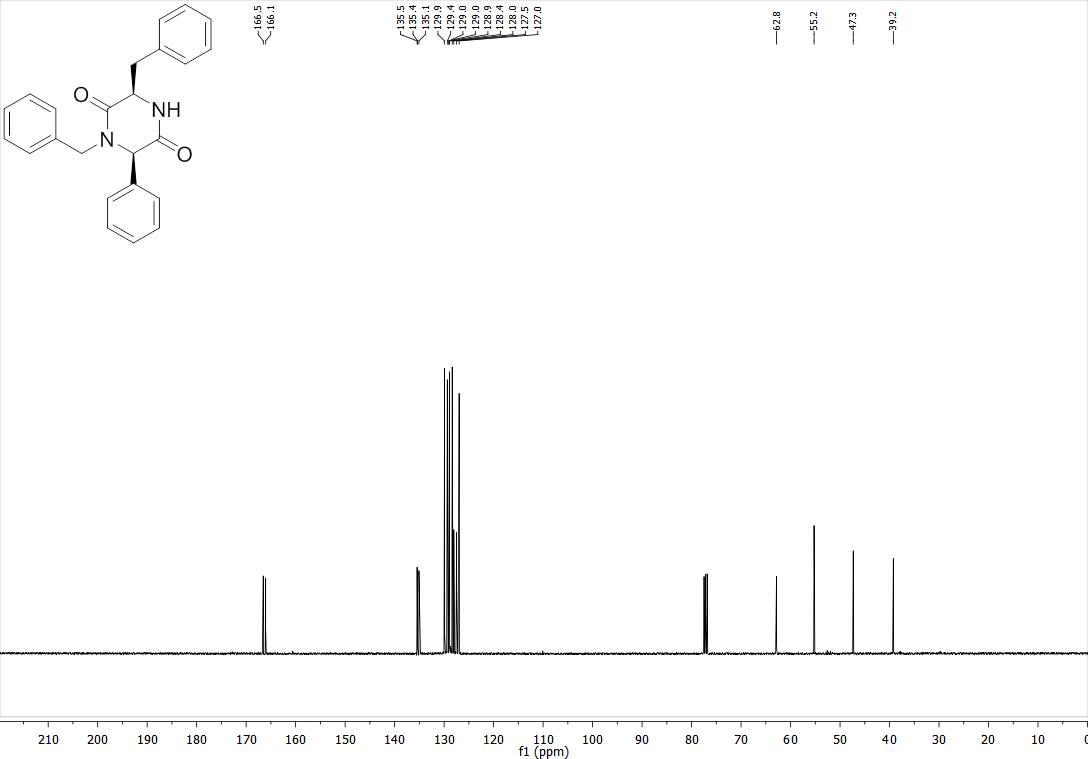
**

**Figure S47**. ^13^C NMR spectrum of compound **22RR**.

**Figure S48**. NOESY spectrum of compound **22RR**.

**
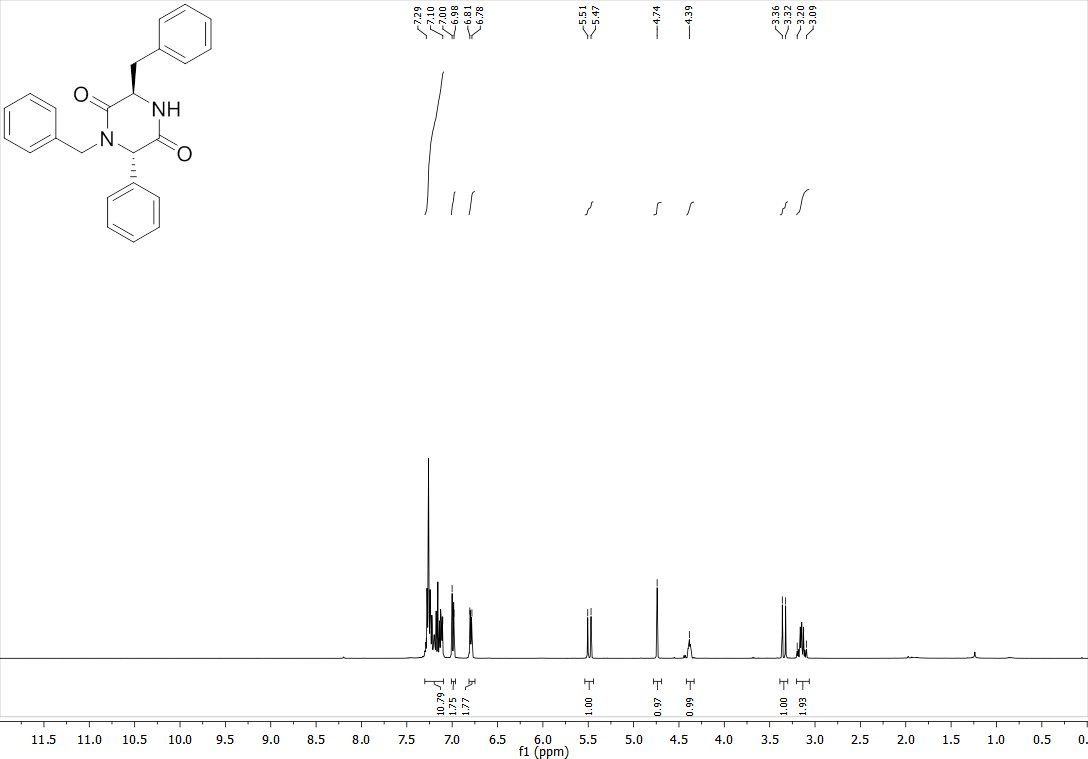
**

**Figure S49.** ^1^H NMR spectrum of compound **22RS**.

**
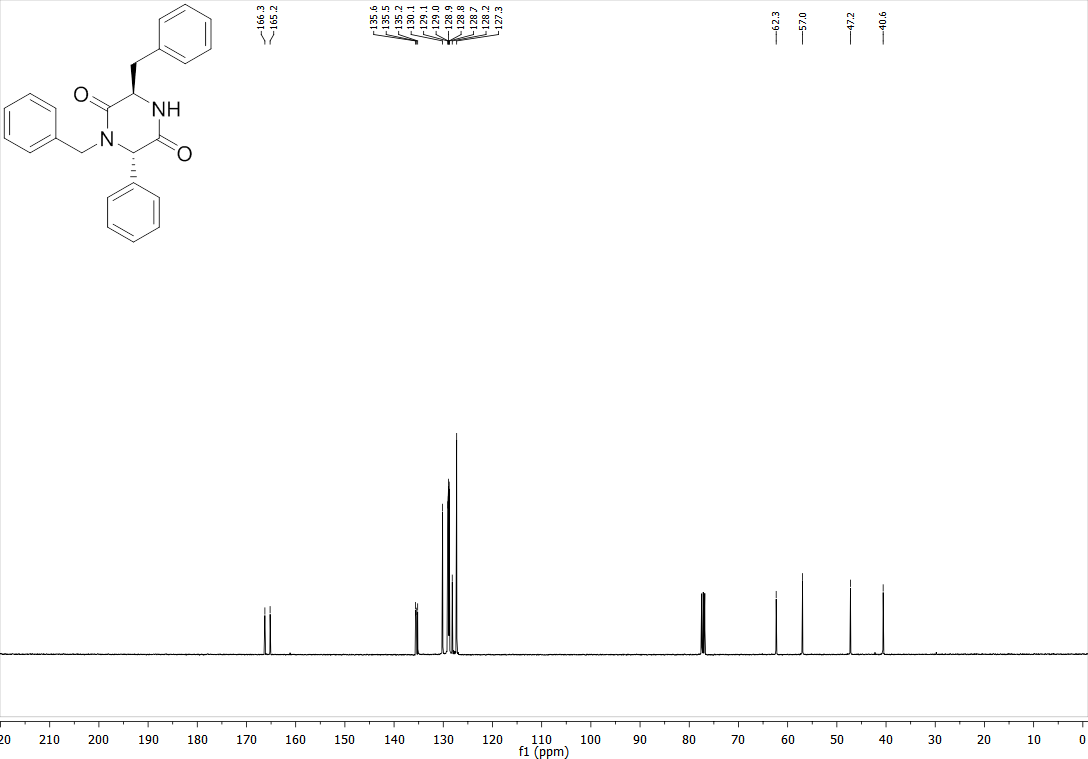
**

**Figure S50**. ^13^C NMR spectrum of compound **22RS**.

**Figure S51**. NOESY spectrum of compound **22RS**.


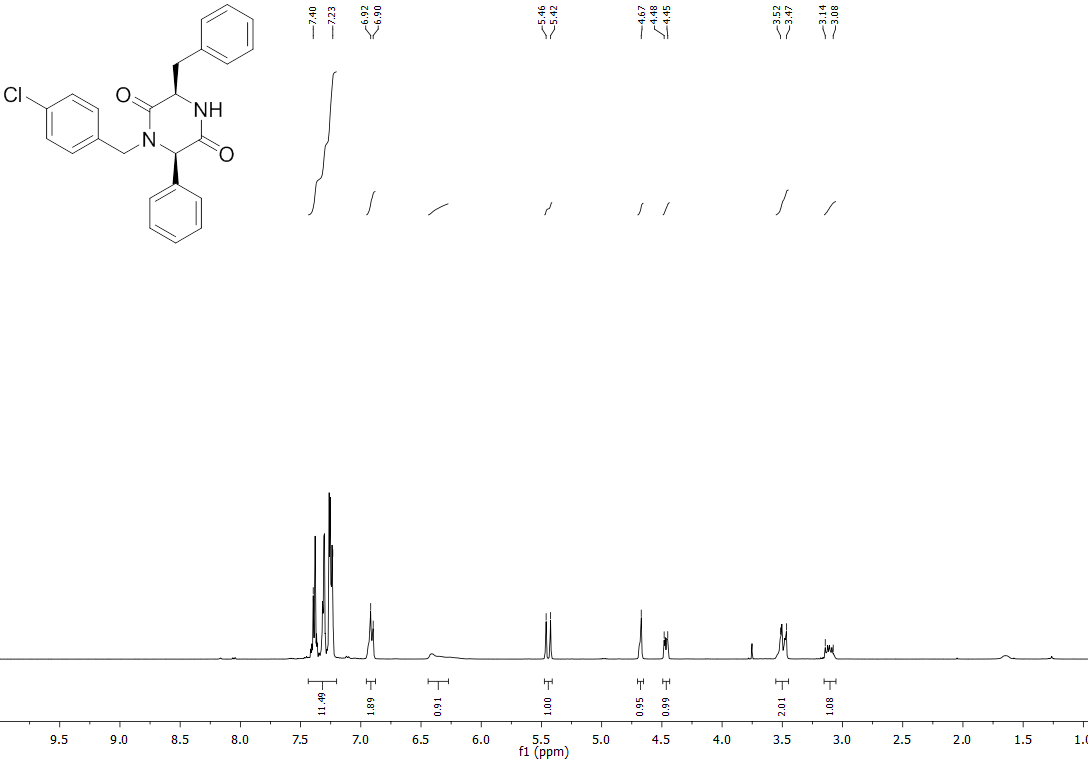


**Figure S52.** ^1^H NMR spectrum of compound **23RR**.

**
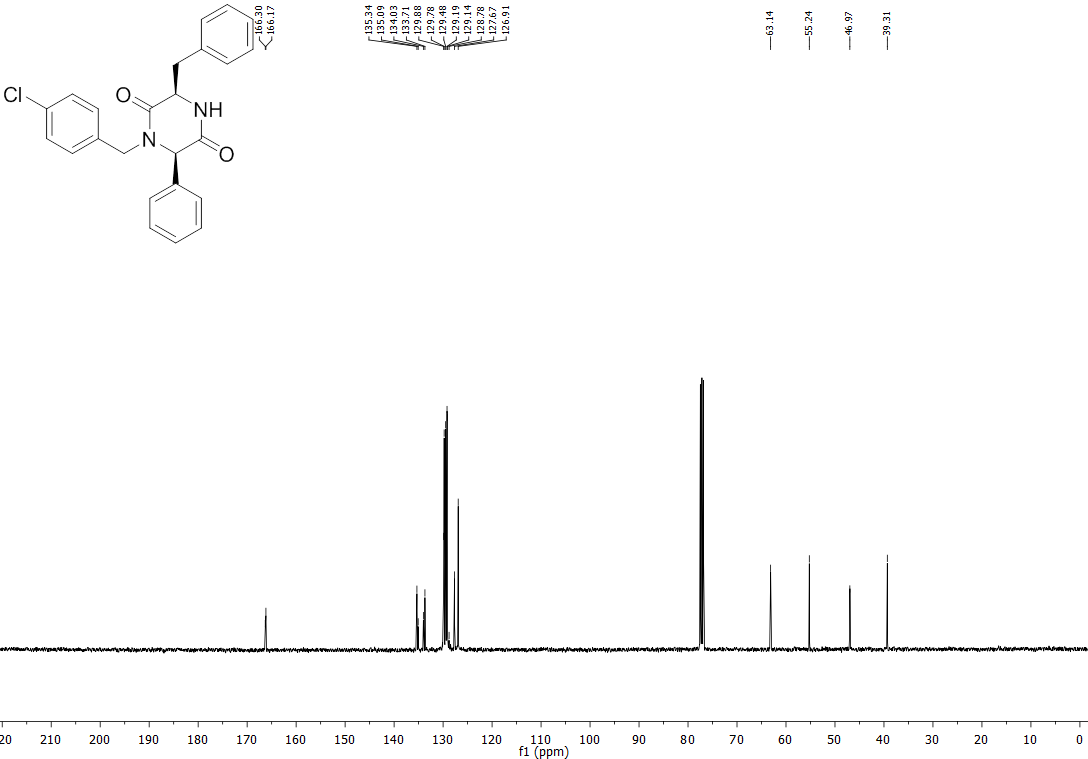
**

**Figure S53**. ^13^C NMR spectrum of compound **23RR**.

**
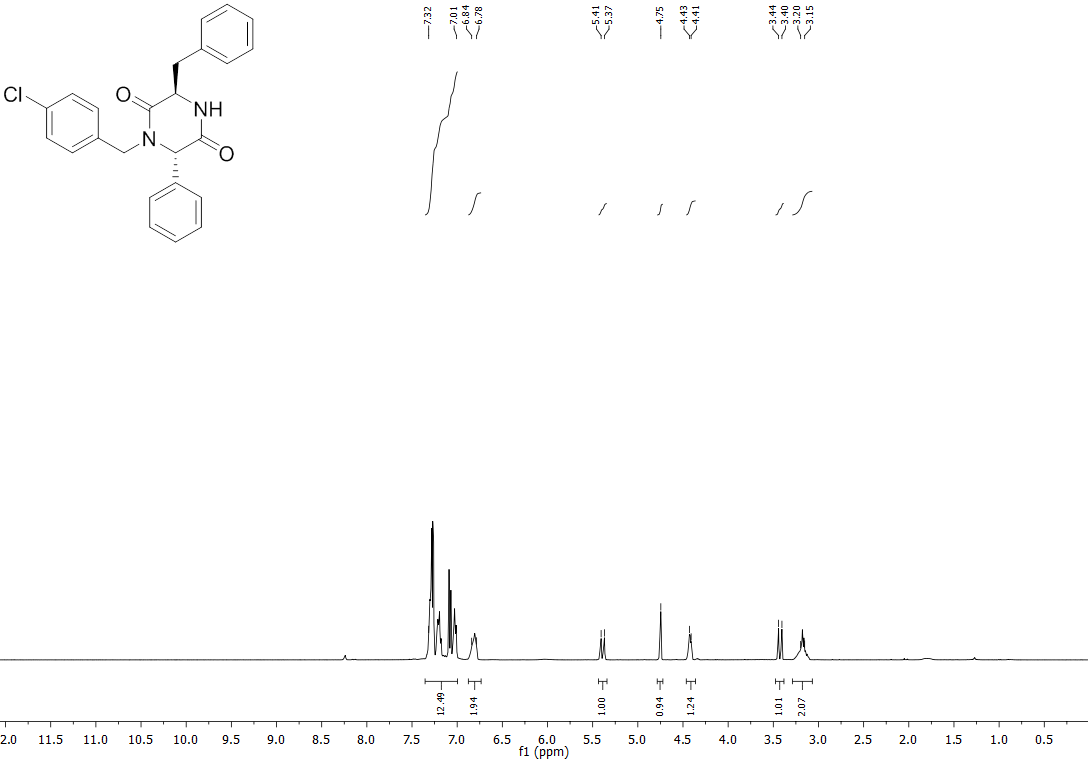
**

**Figure S54.** ^1^H NMR spectrum of compound **23RS**.

**
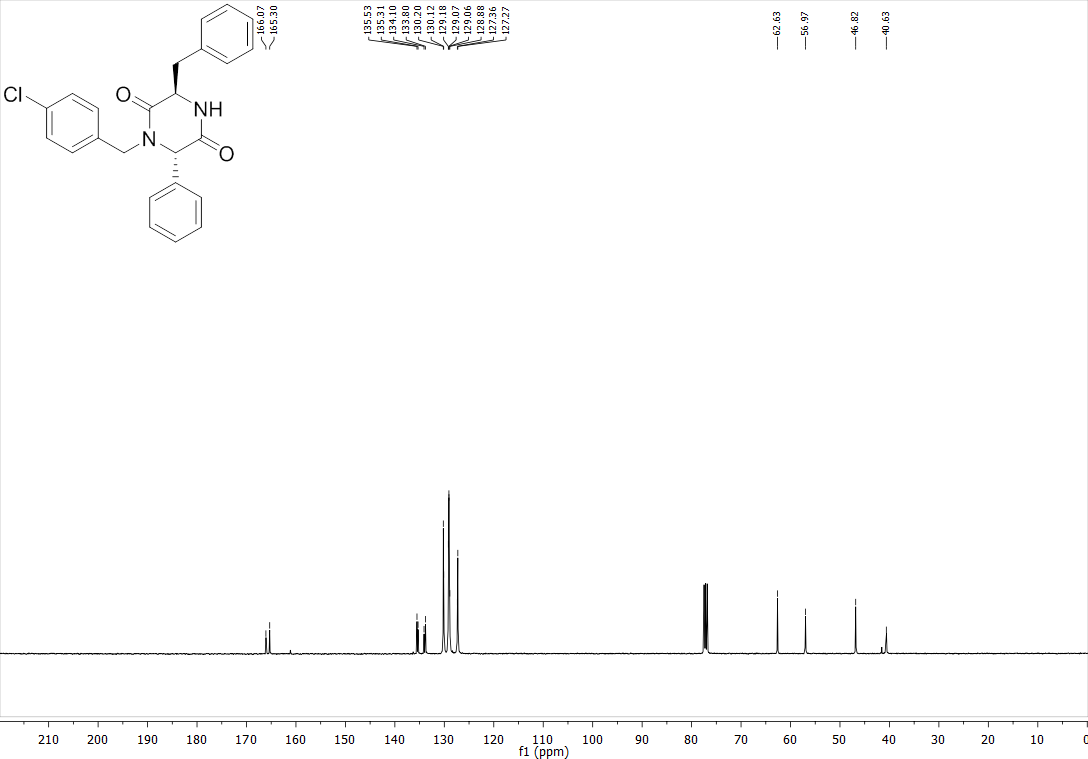
**

**Figure S55**. ^13^C NMR spectrum of compound **23RS**.


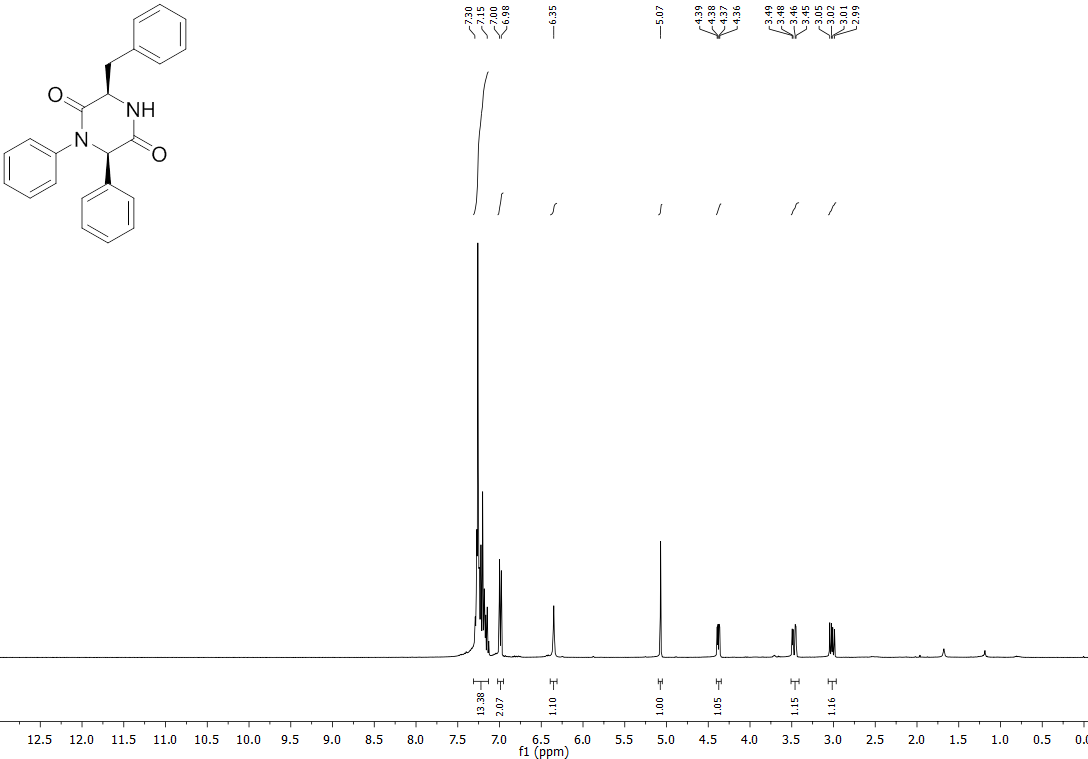


**Figure S56**. ^1^H NMR spectrum of compound **24RR**.


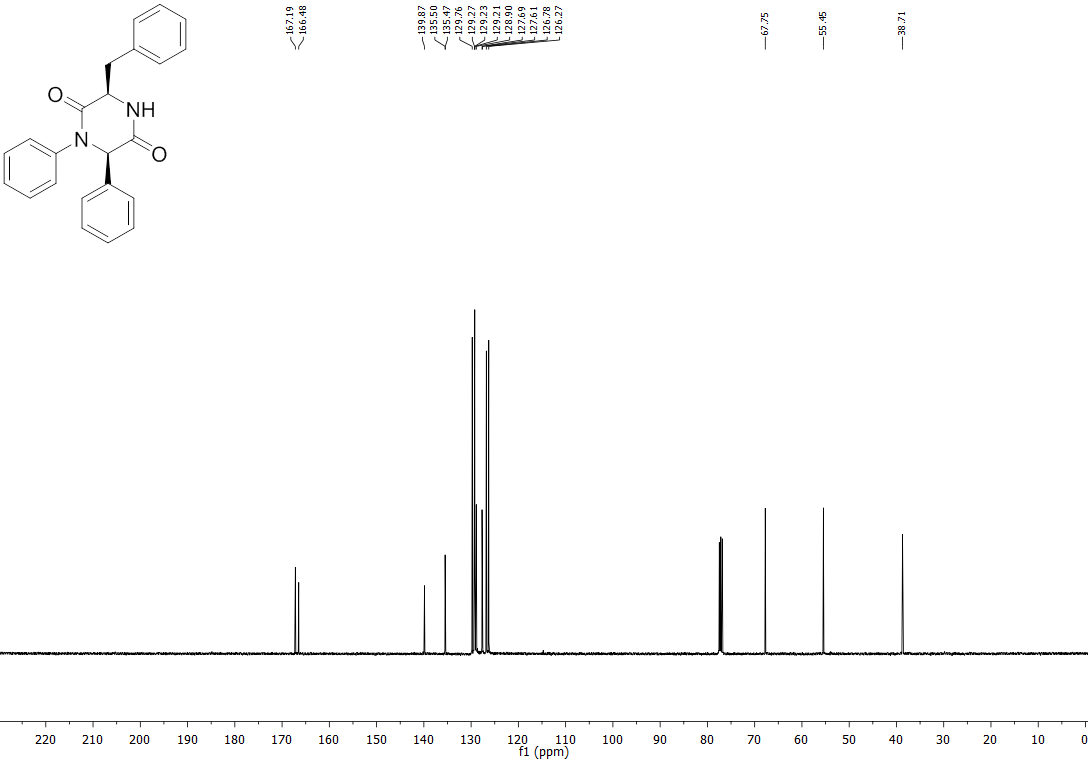


**Figure S57**. ^13^C NMR spectrum of compound **24RR**.


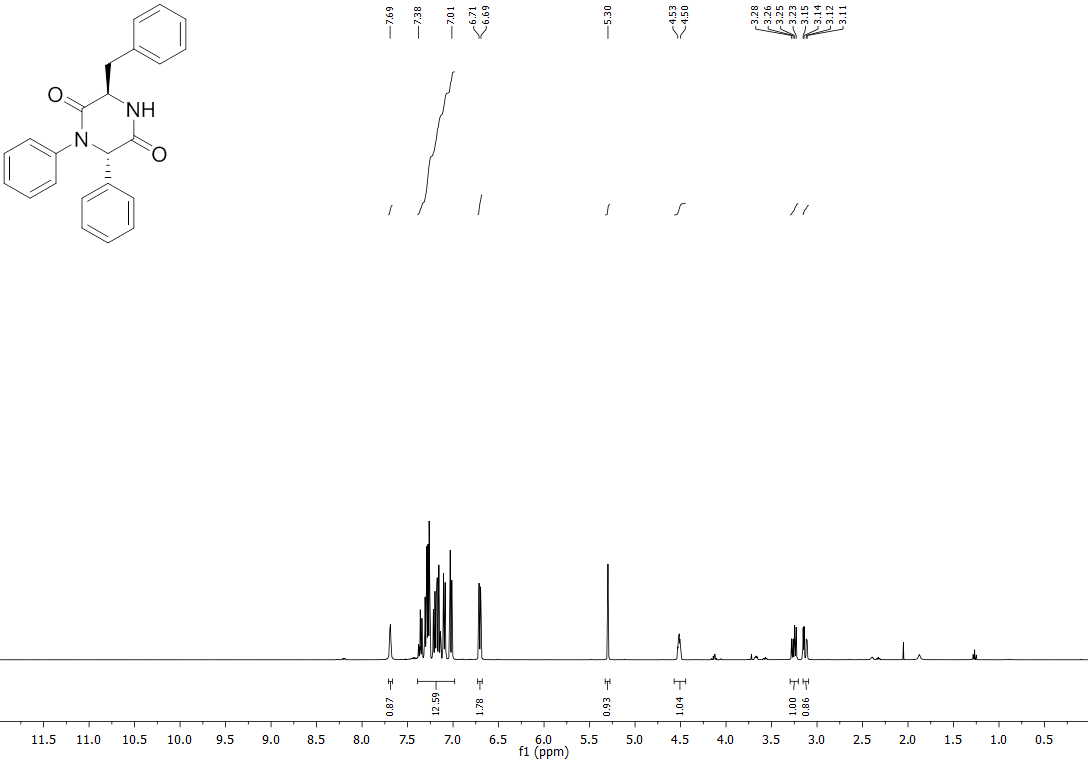


**Figure S58**. ^1^H NMR spectrum of compound **24RS**.


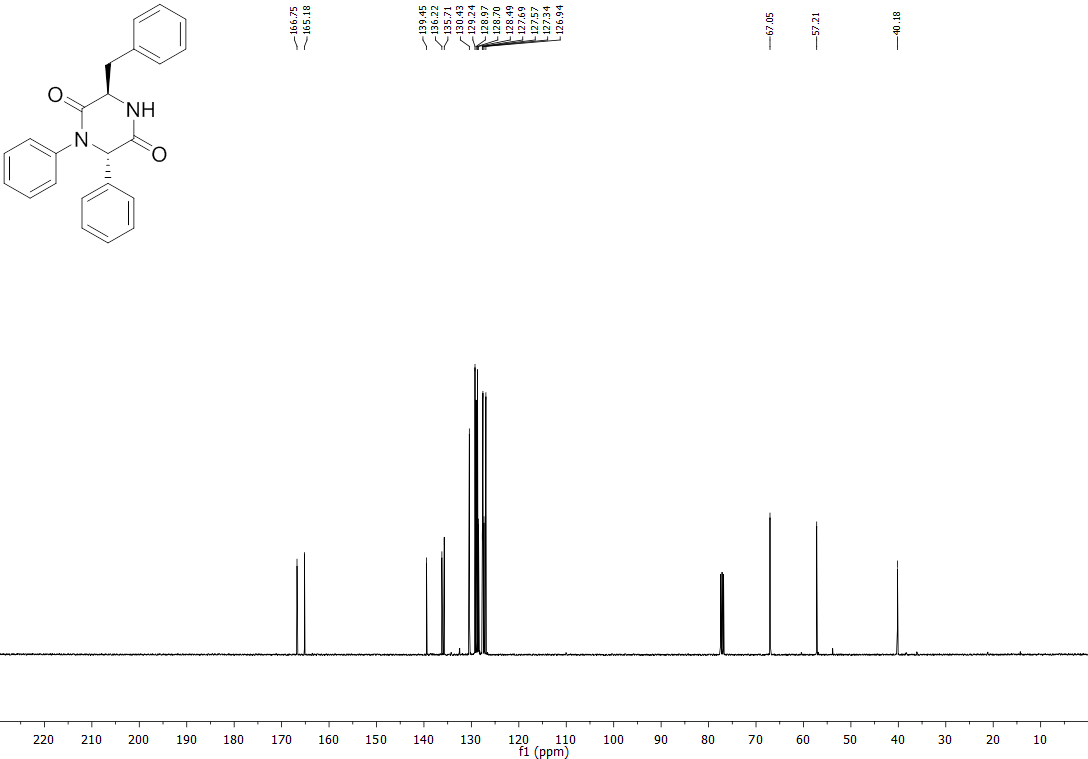


**Figure S59**. ^13^C NMR spectrum of compound **24RS**.


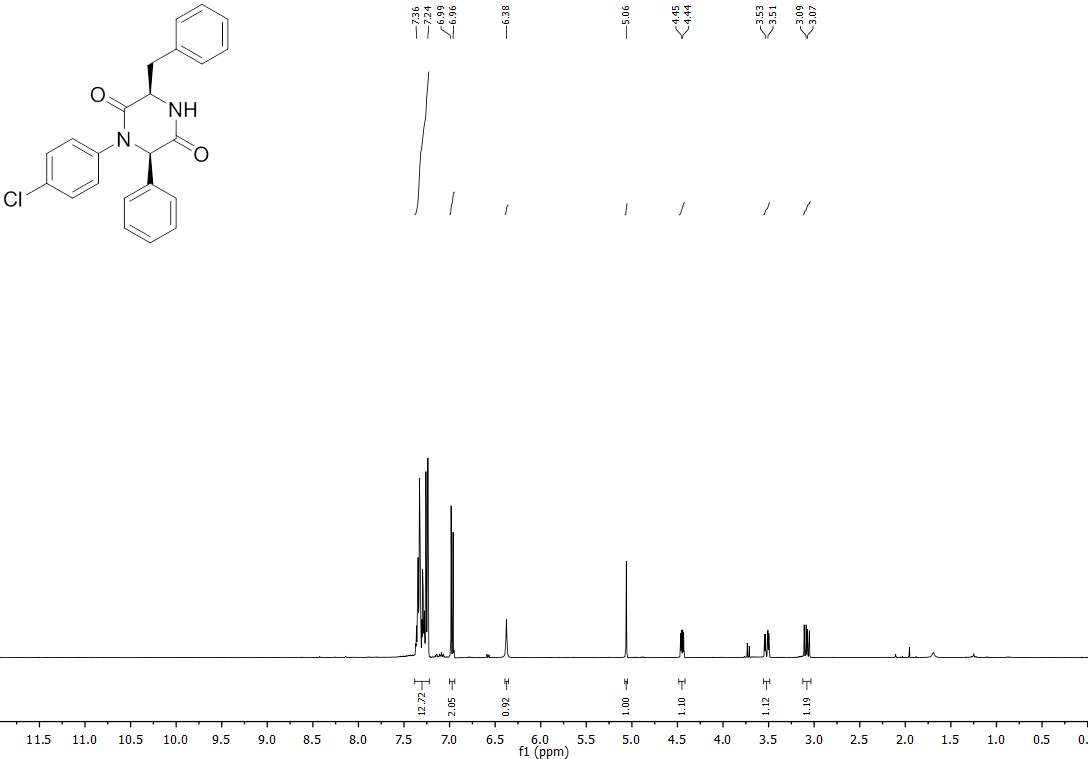


**Figure S60**. ^1^H NMR spectrum of compound **25RR**.


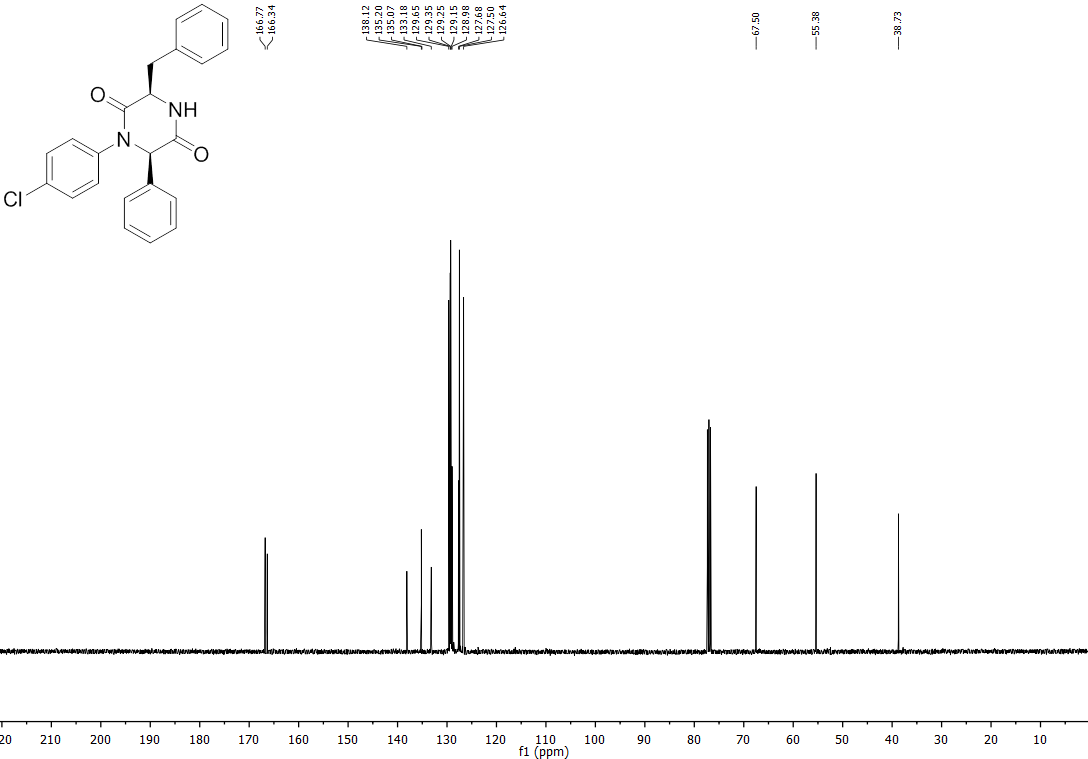


**Figure S61**. ^13^C NMR spectrum of compound **25RR**.


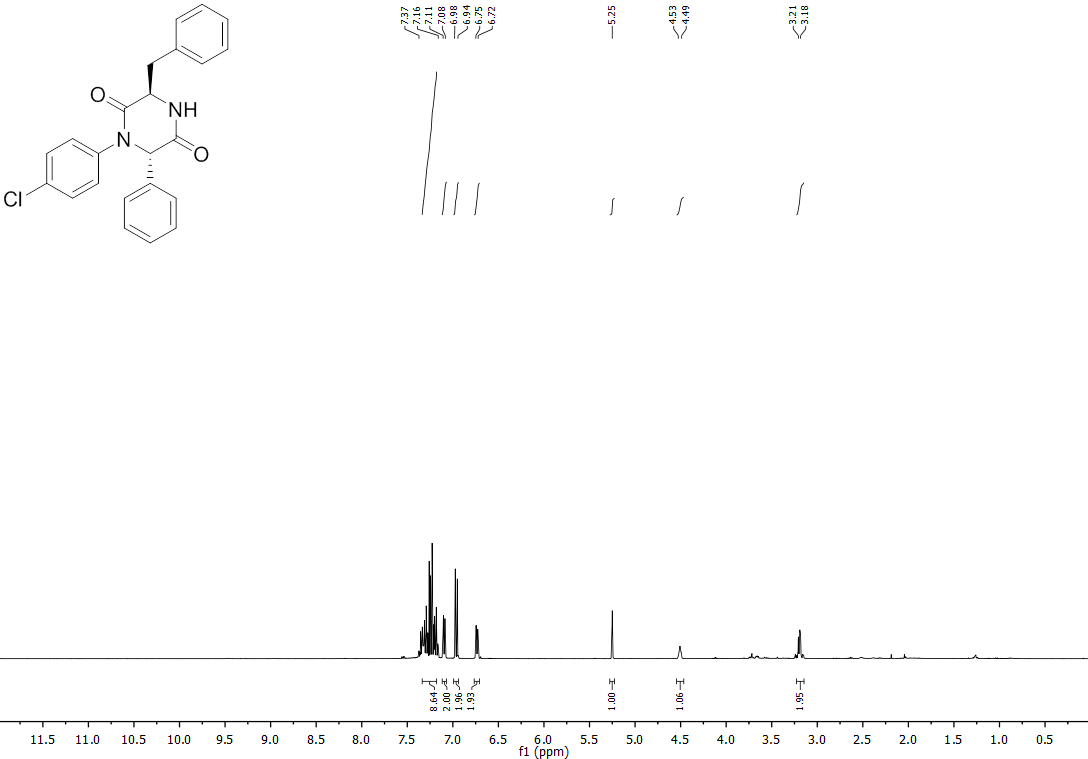


**Figure S62**. ^1^H NMR spectrum of compound **25RS**.


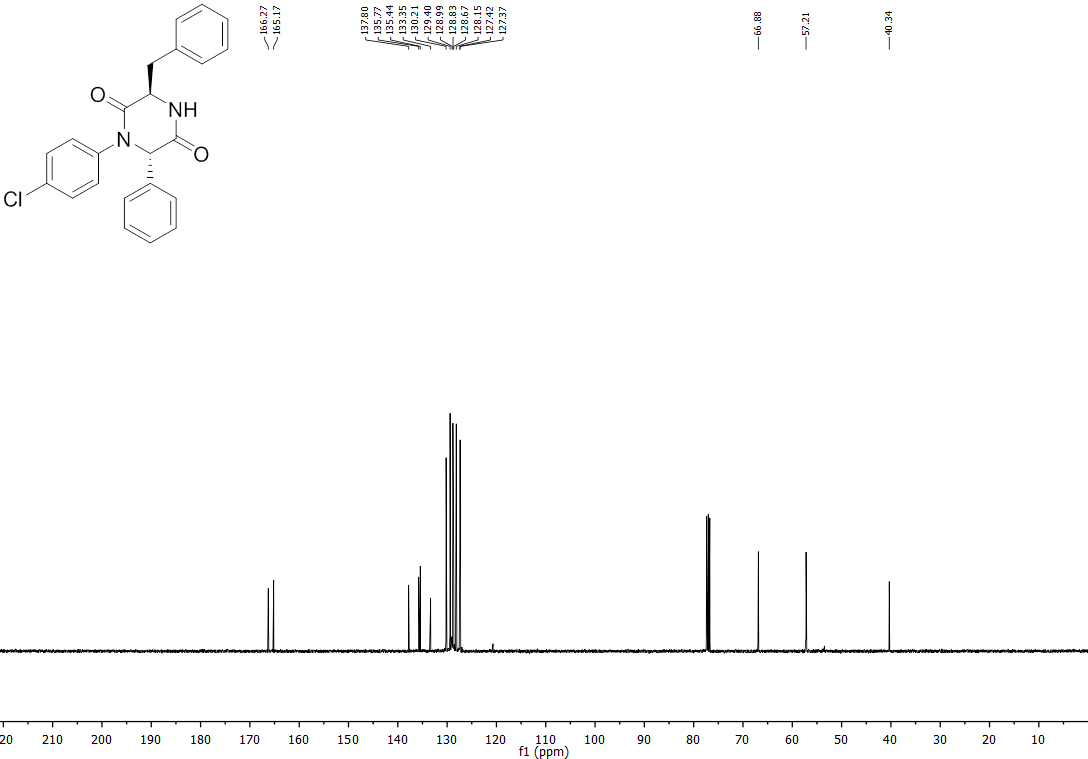


**Figure S63**. ^13^C NMR spectrum of compound **25RS**.


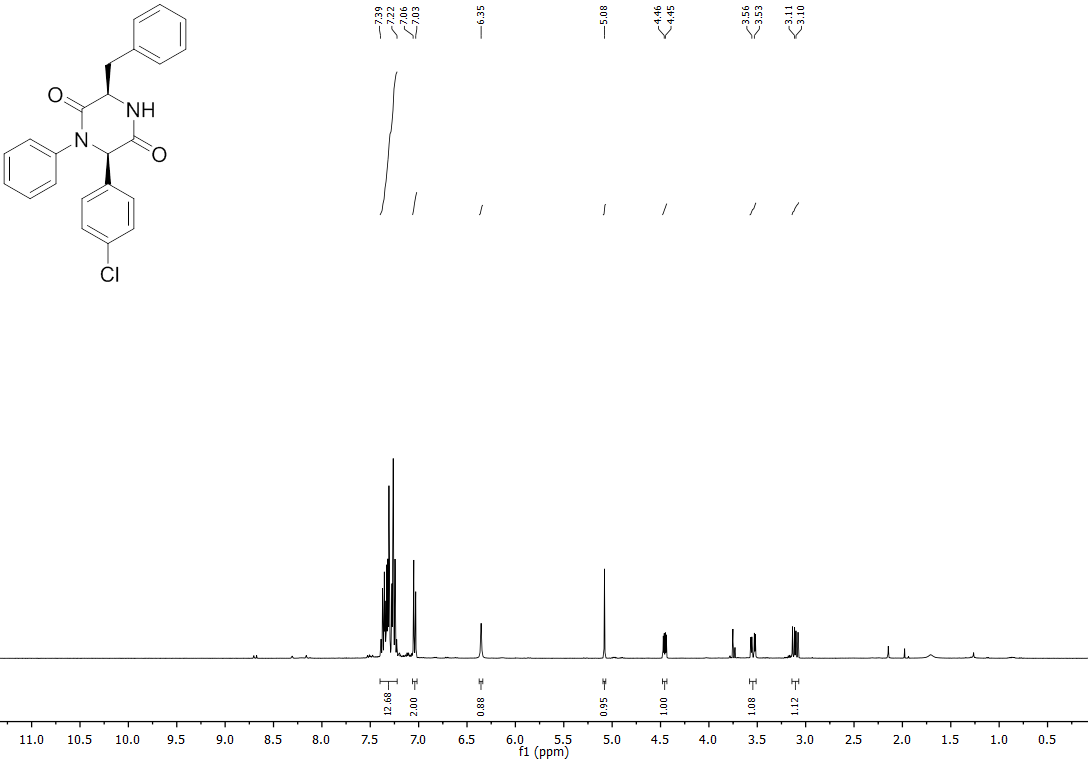


**Figure S64**. ^1^H NMR spectrum of compound **26RR**.


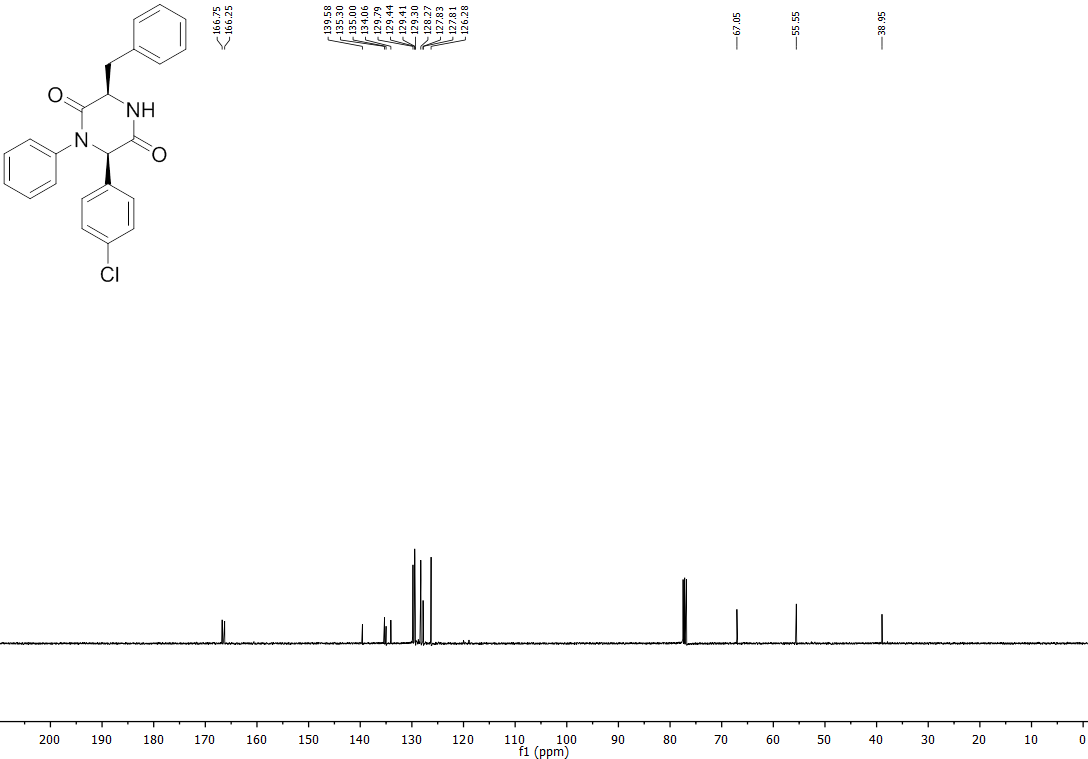


**Figure S65**. ^13^C NMR spectrum of compound **26RR**.


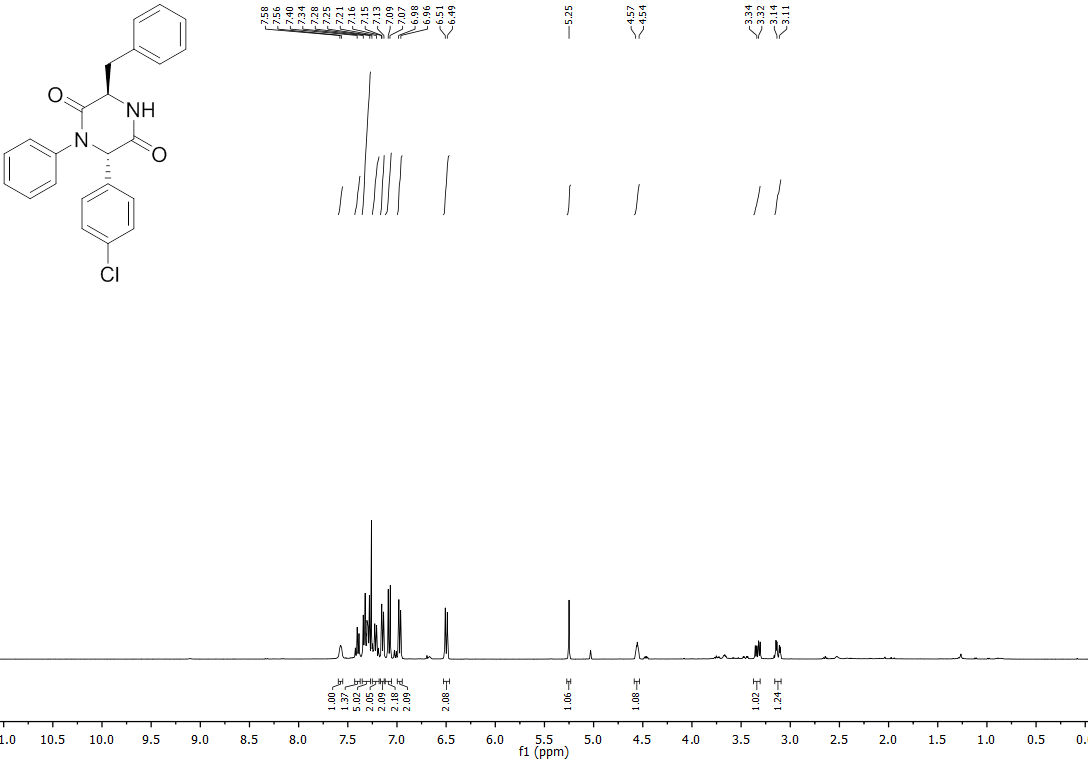


**Figure S66**. ^1^H NMR spectrum of compound **26RS**.


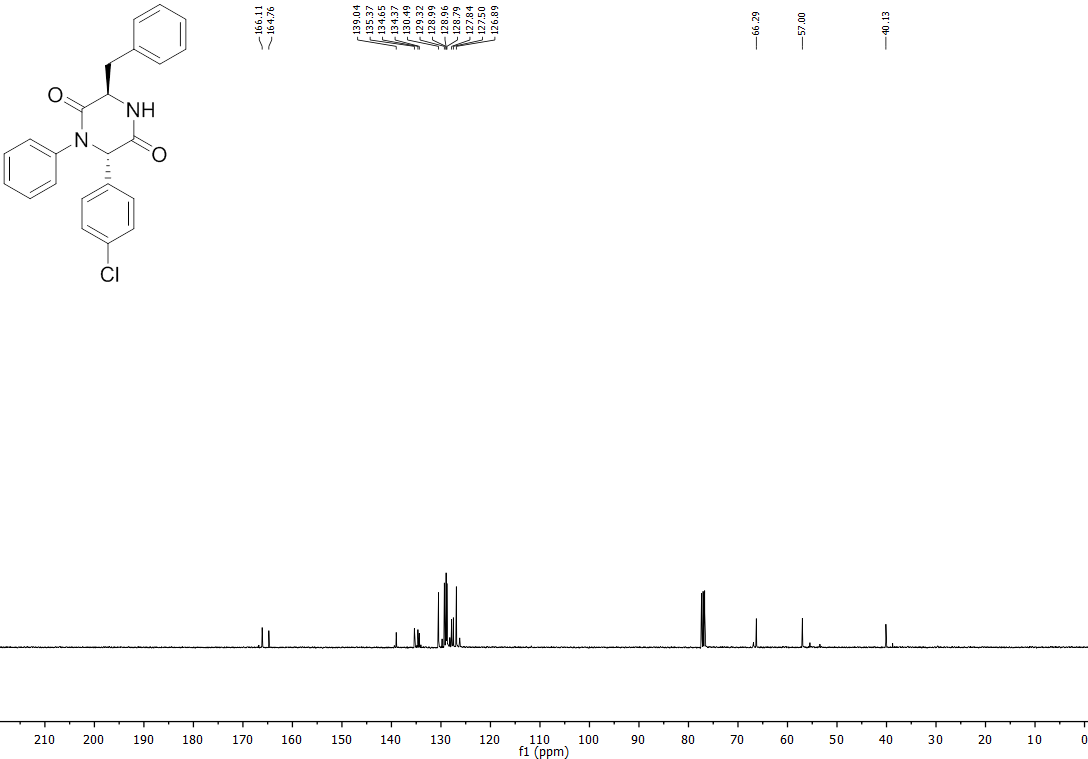


**Figure S67**. ^13^C NMR spectrum of compound **26RS**.


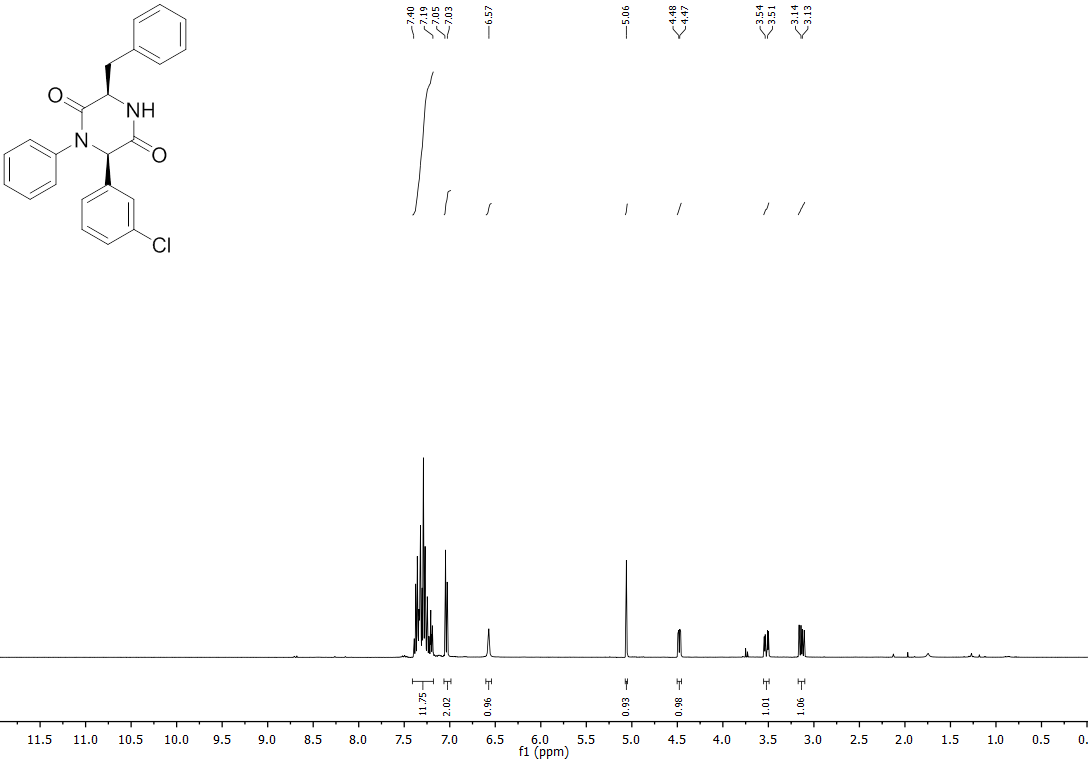


**Figure S68**. ^1^H NMR spectrum of compound **27RR**.


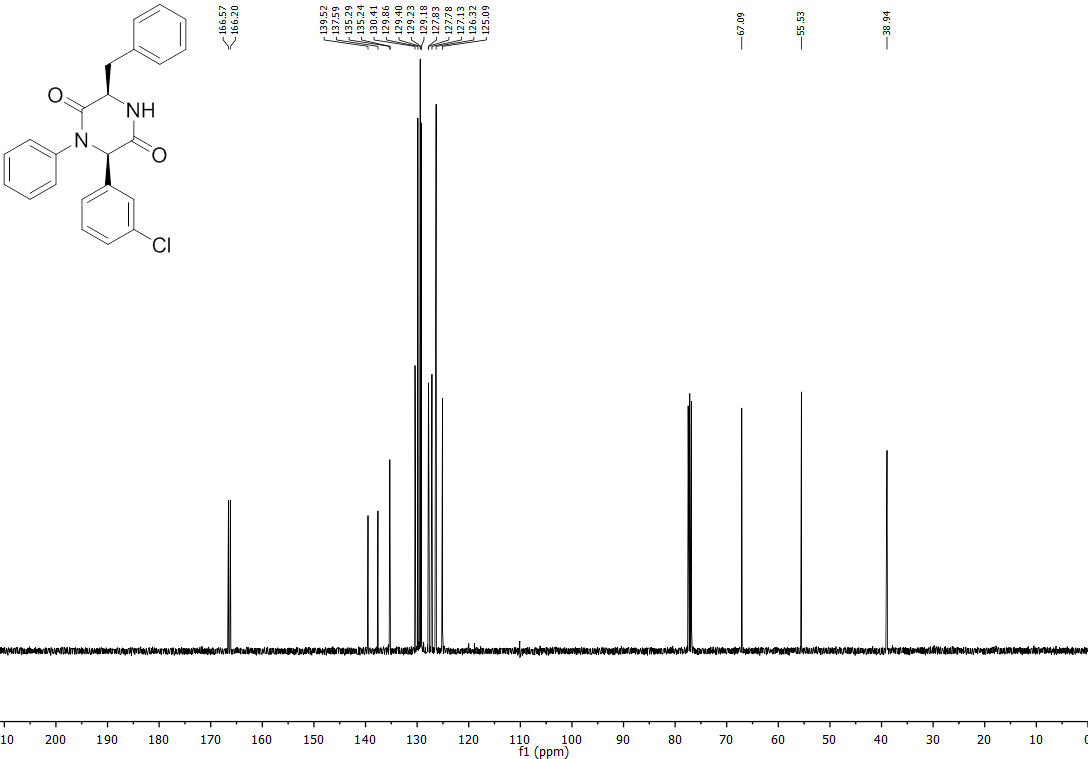


**Figure S69**. ^13^C NMR spectrum of compound **27RR**.


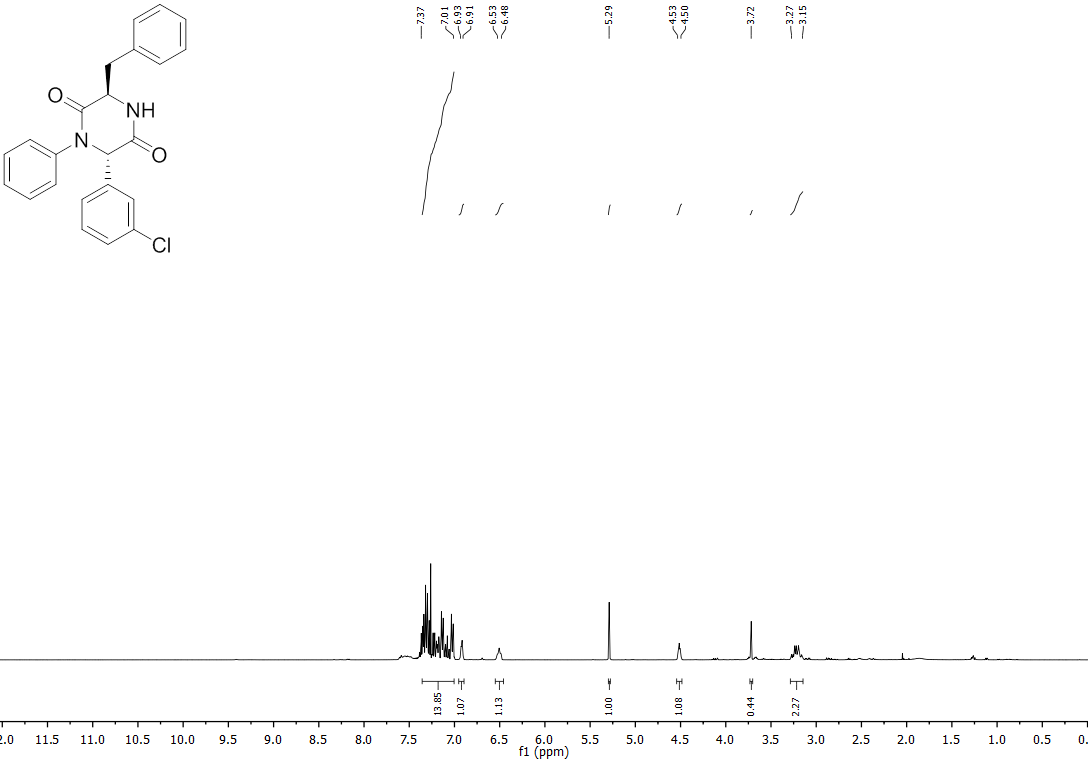


**Figure S70**. ^1^H NMR spectrum of compound **27RS**.


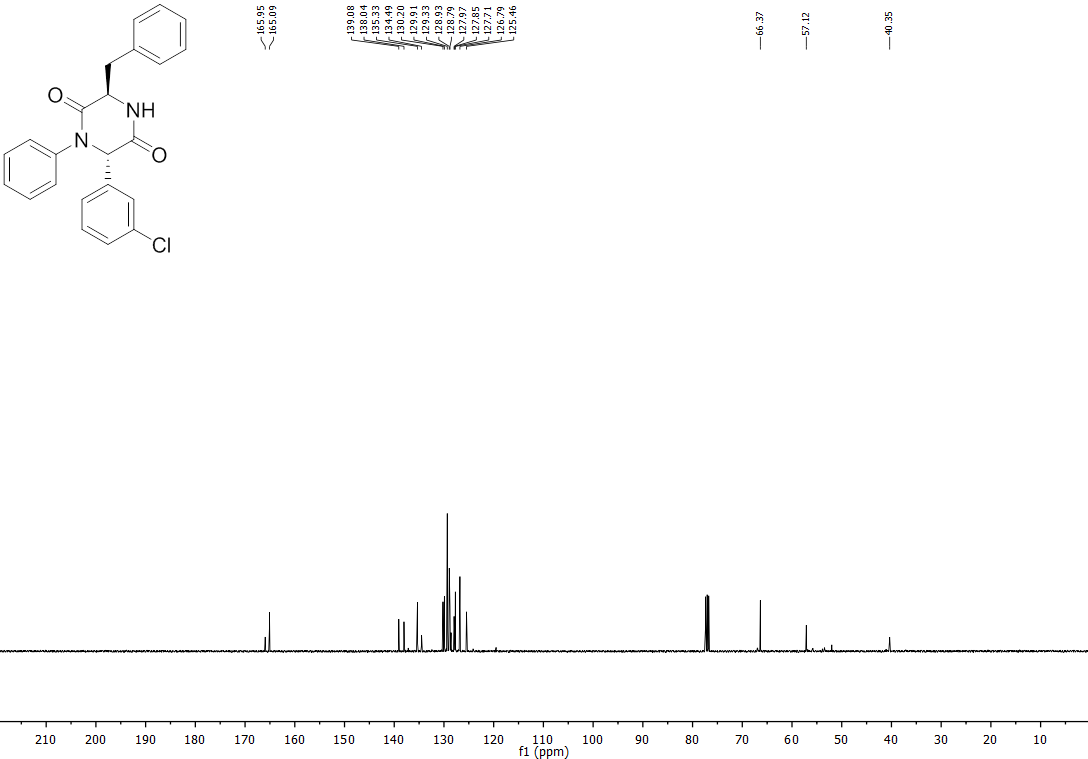


**Figure S71**. ^13^C NMR spectrum of compound **27RS**.


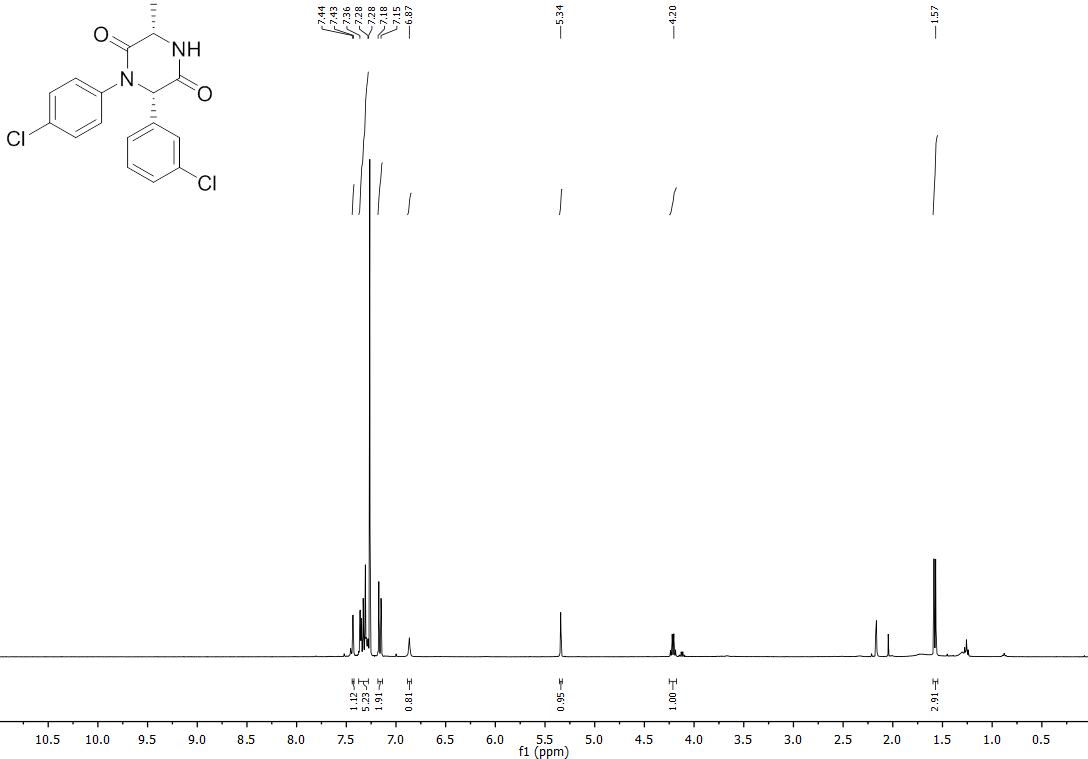


**Figure S72**. ^1^H NMR spectrum of compound **29SS**.


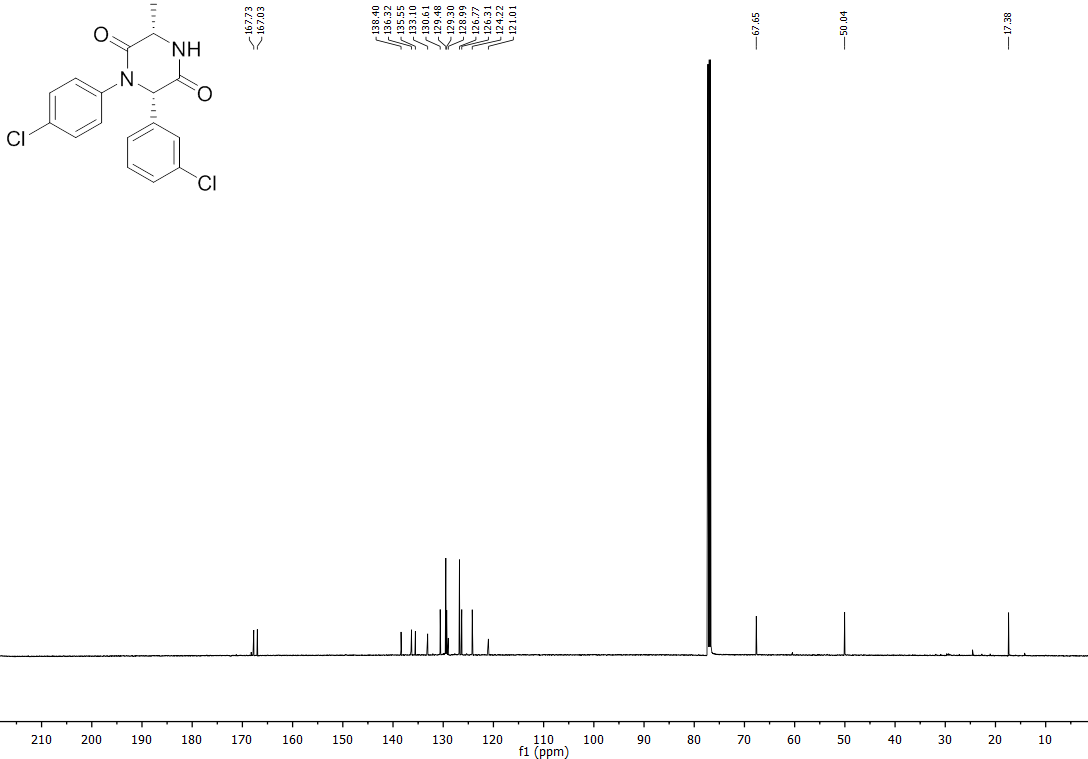


**Figure S73**. ^13^C NMR spectrum of compound **29SS**.

**
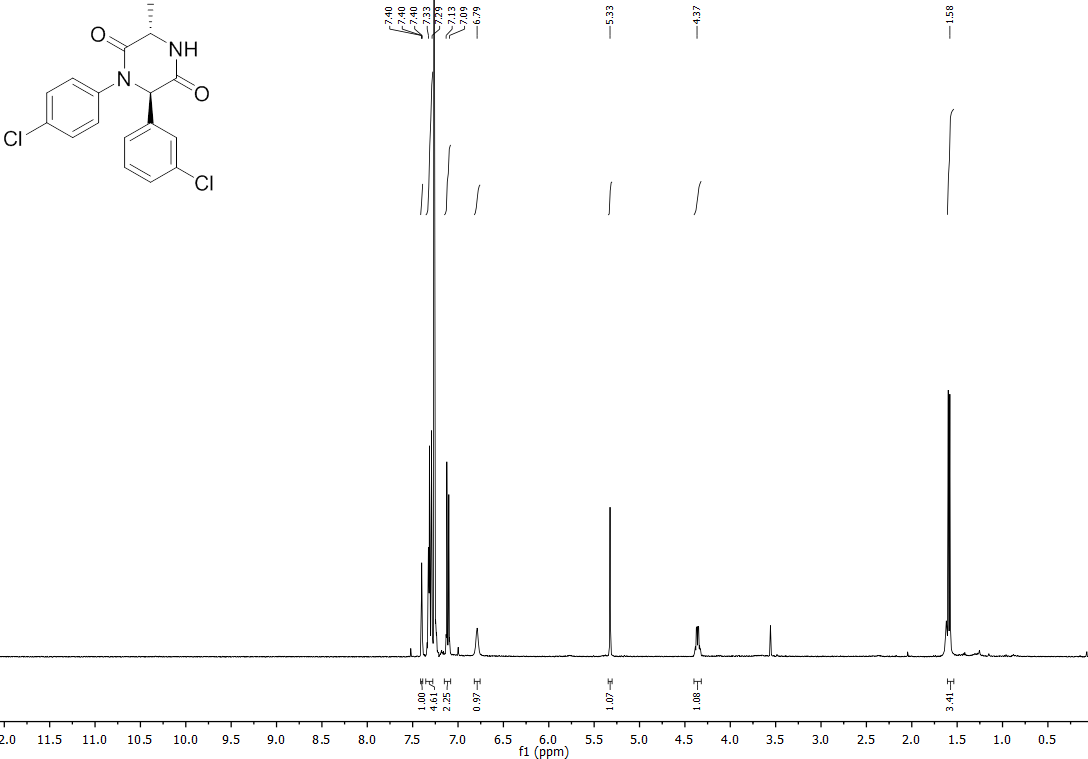
**

**Figure S74**. ^1^H NMR spectrum of compound **29SR**.

**
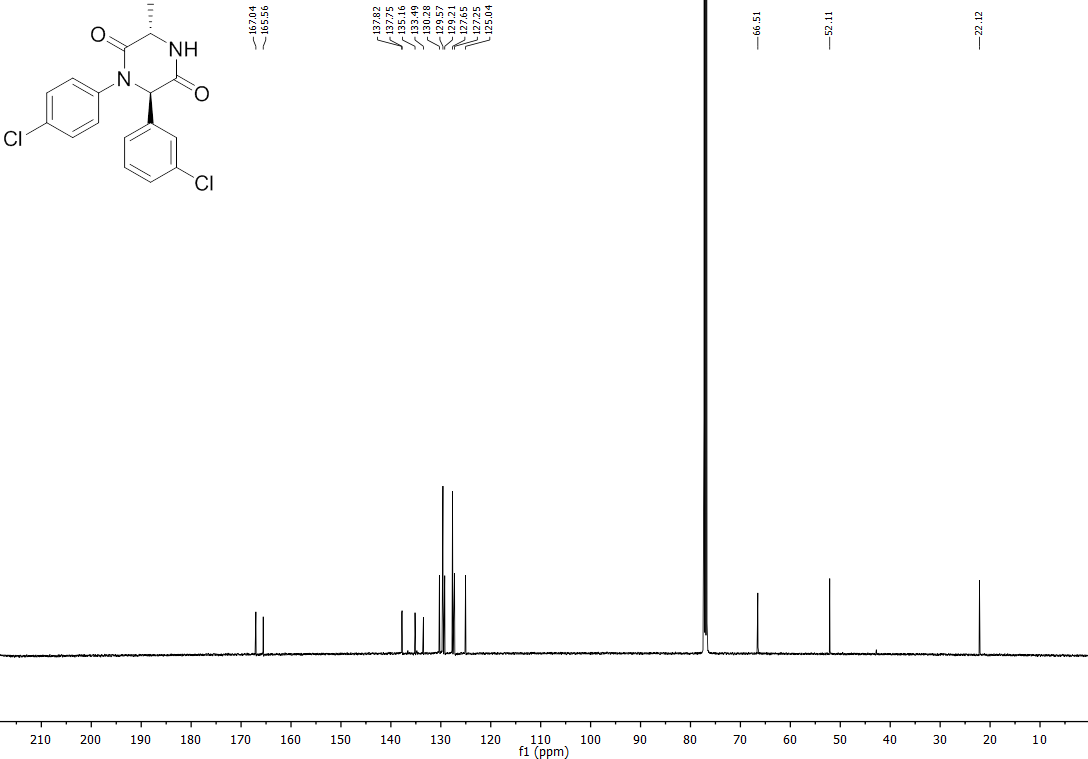
**

**Figure S75**. ^13^C NMR spectrum of compound **29SR**.


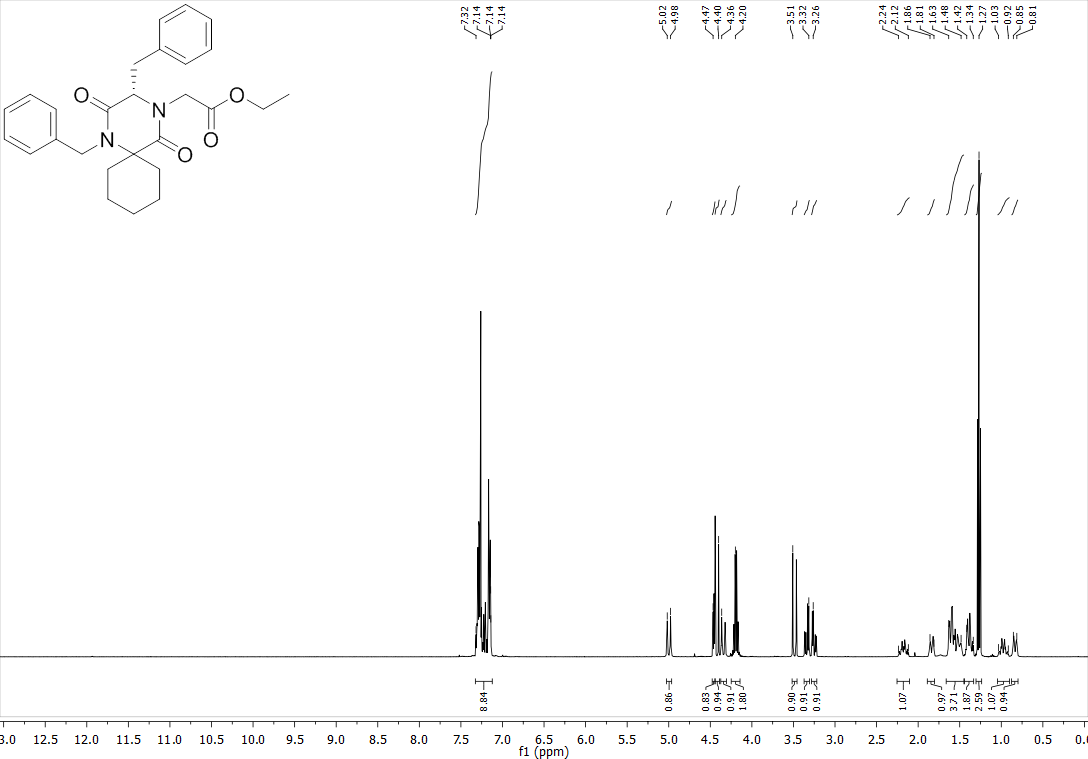


**Figure S76.** ^1^H NMR spectrum of compound **30S**.


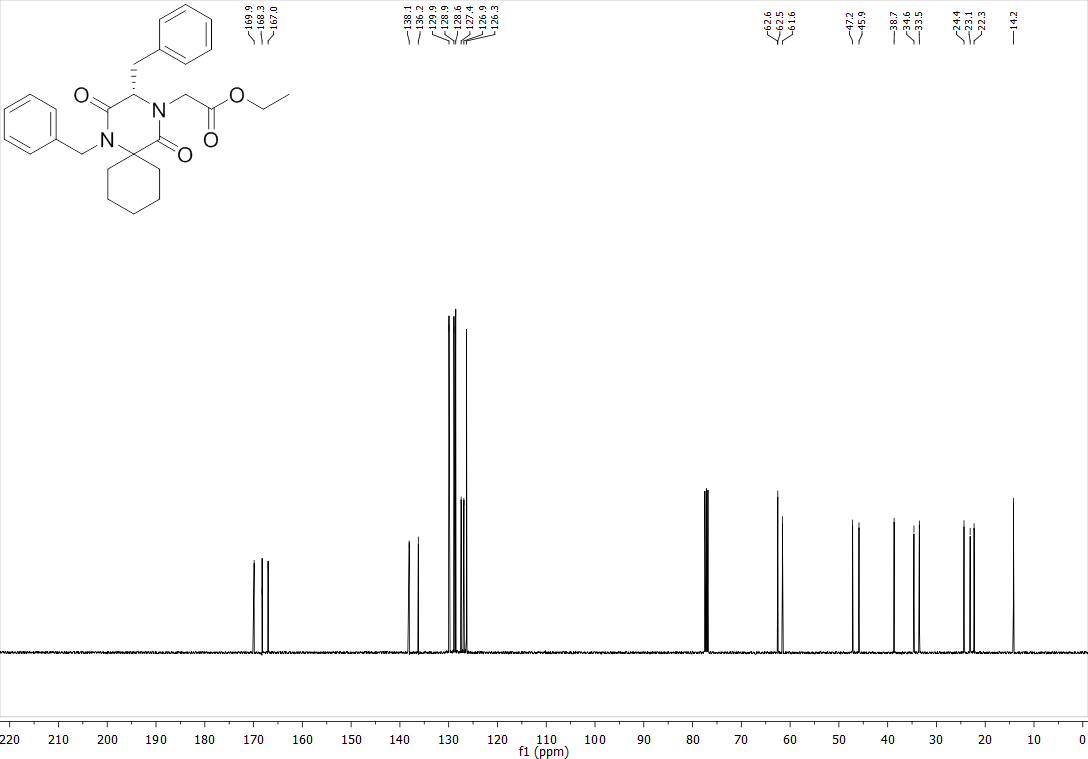


**Figure S77**. ^13^C NMR spectrum of compound **30S**.


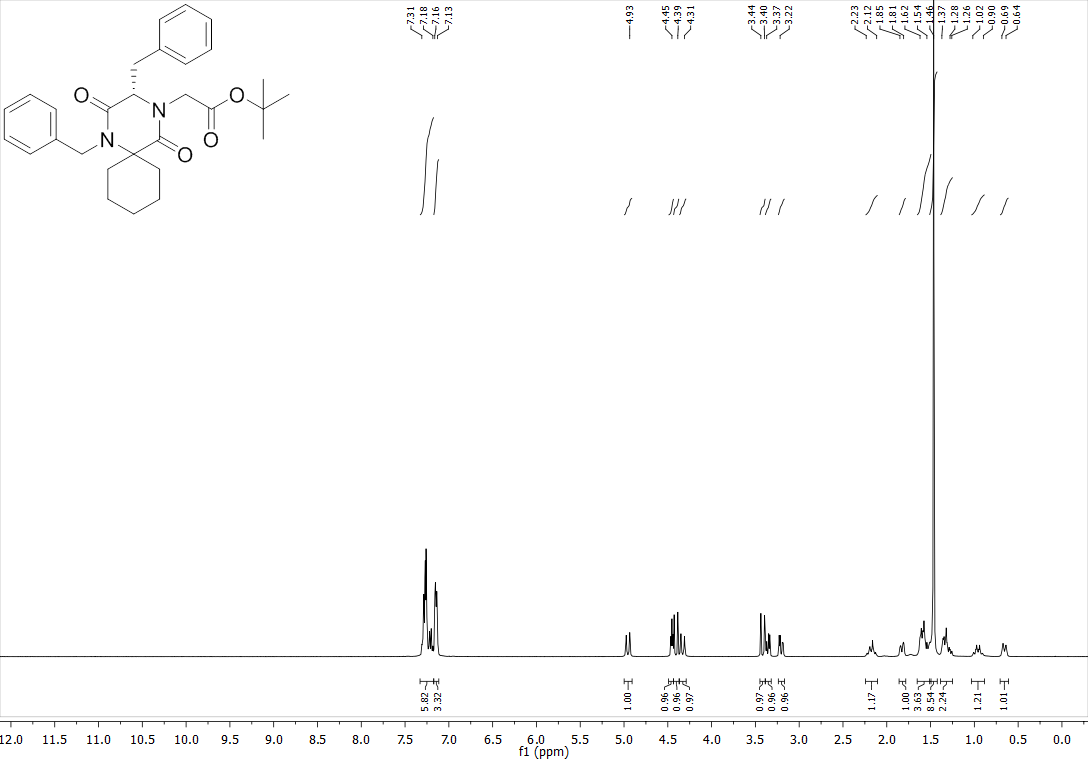


**Figure S78.** ^1^H NMR spectrum of compound **31S**.


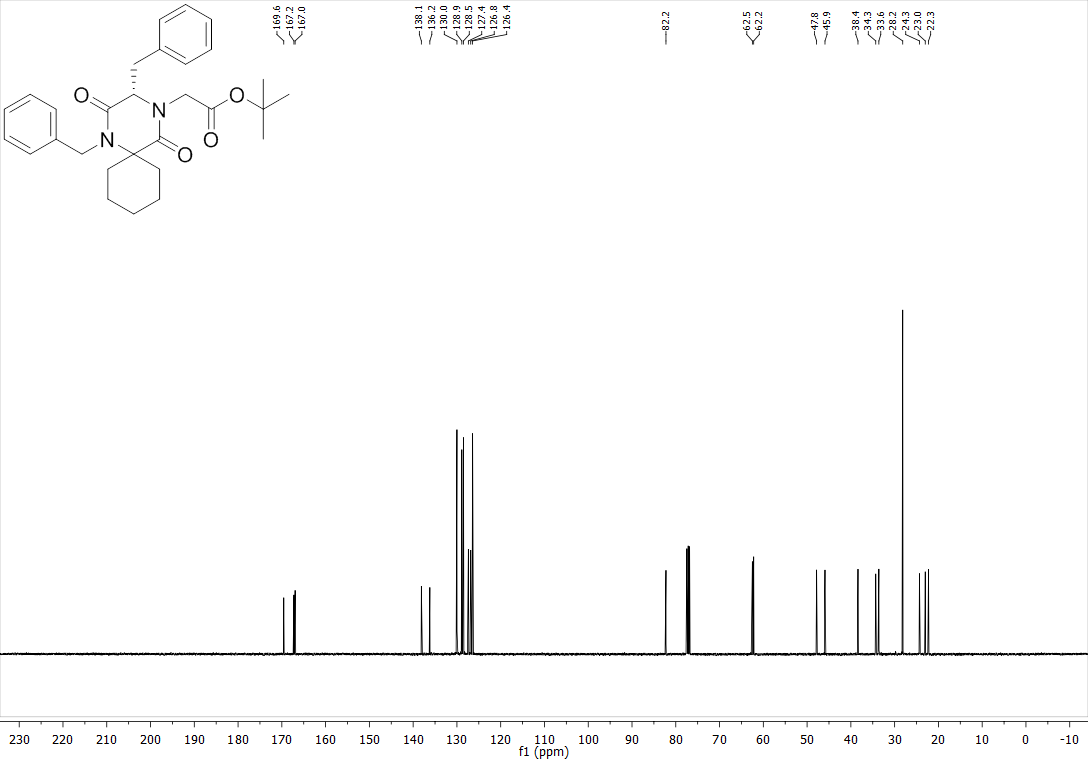


**Figure S79**. ^13^C NMR spectrum of compound **31S**.


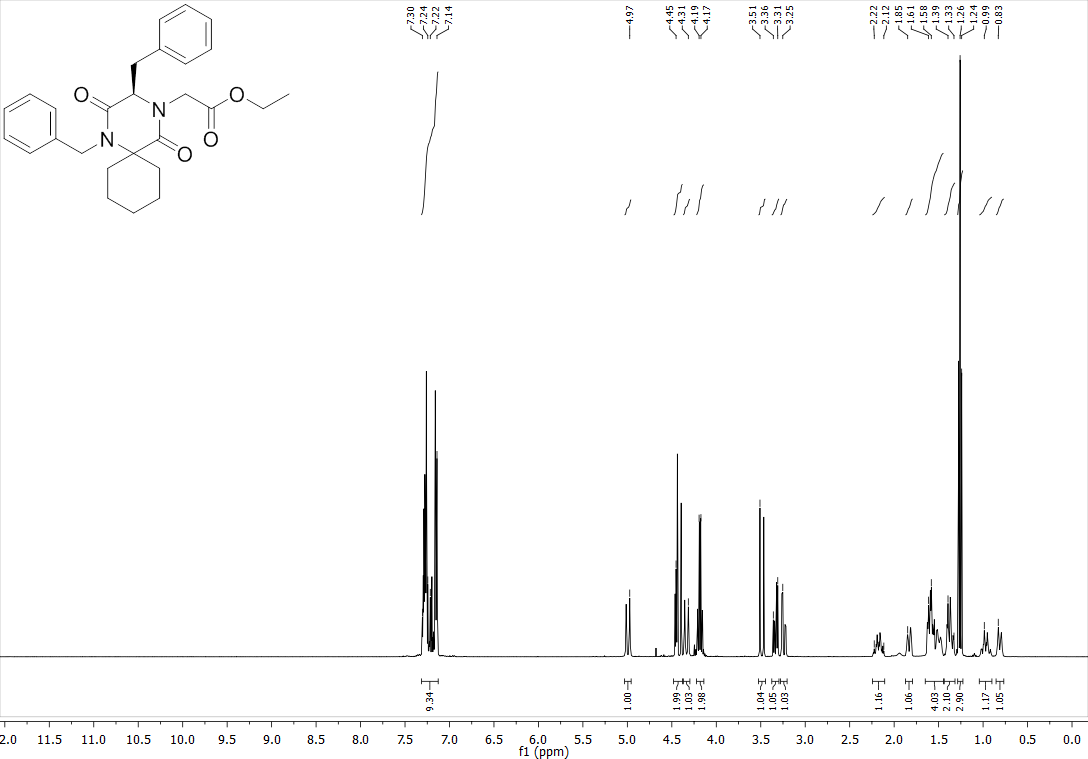


**Figure S80.** ^1^H NMR spectrum of compound **30R**.


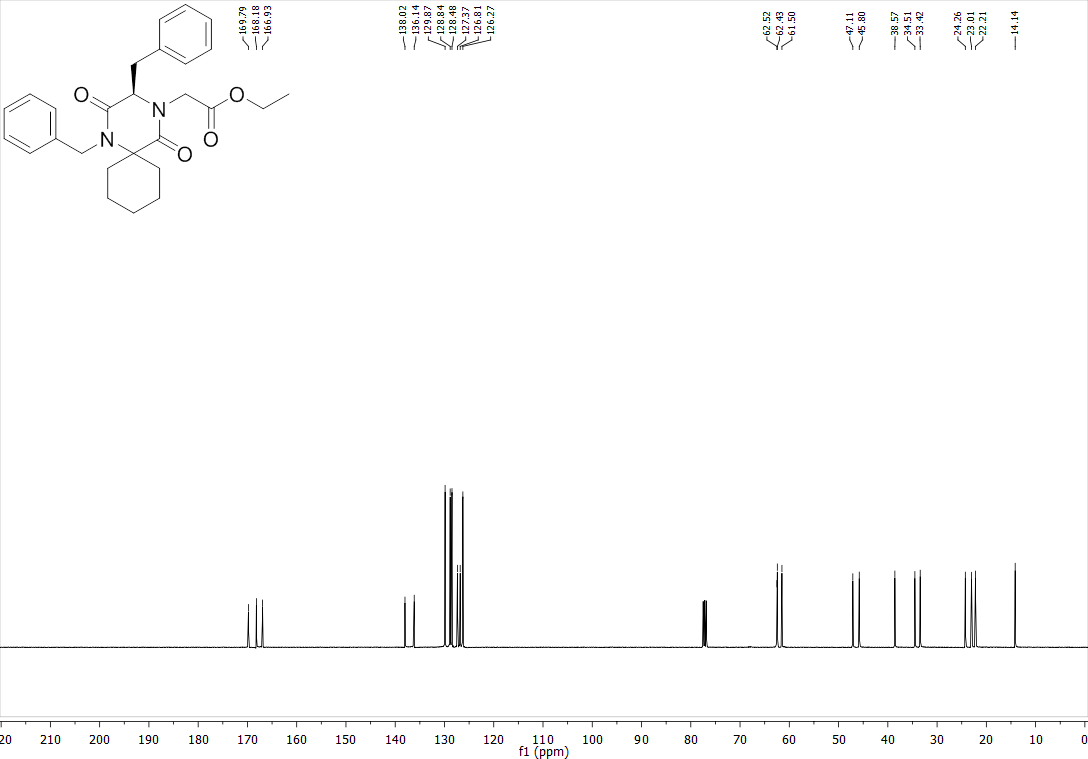


**Figure S81**. ^13^C NMR spectrum of compound **30R**.


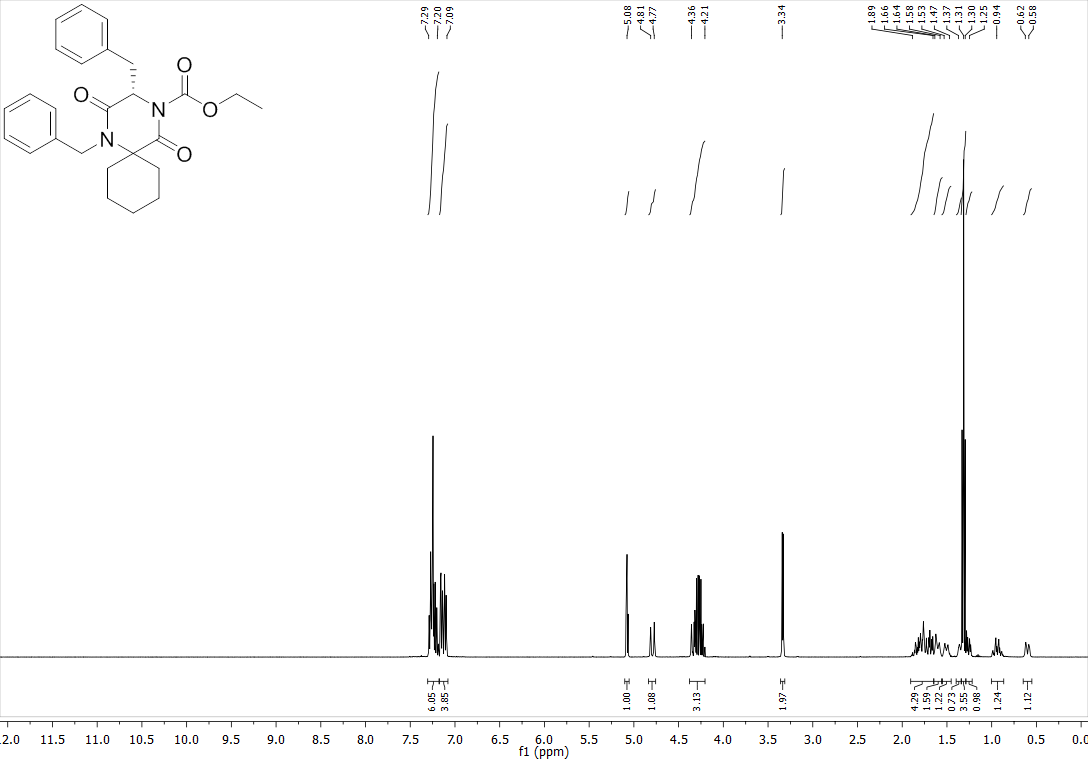


**Figure S82.** ^1^H NMR spectrum of compound **32S**.


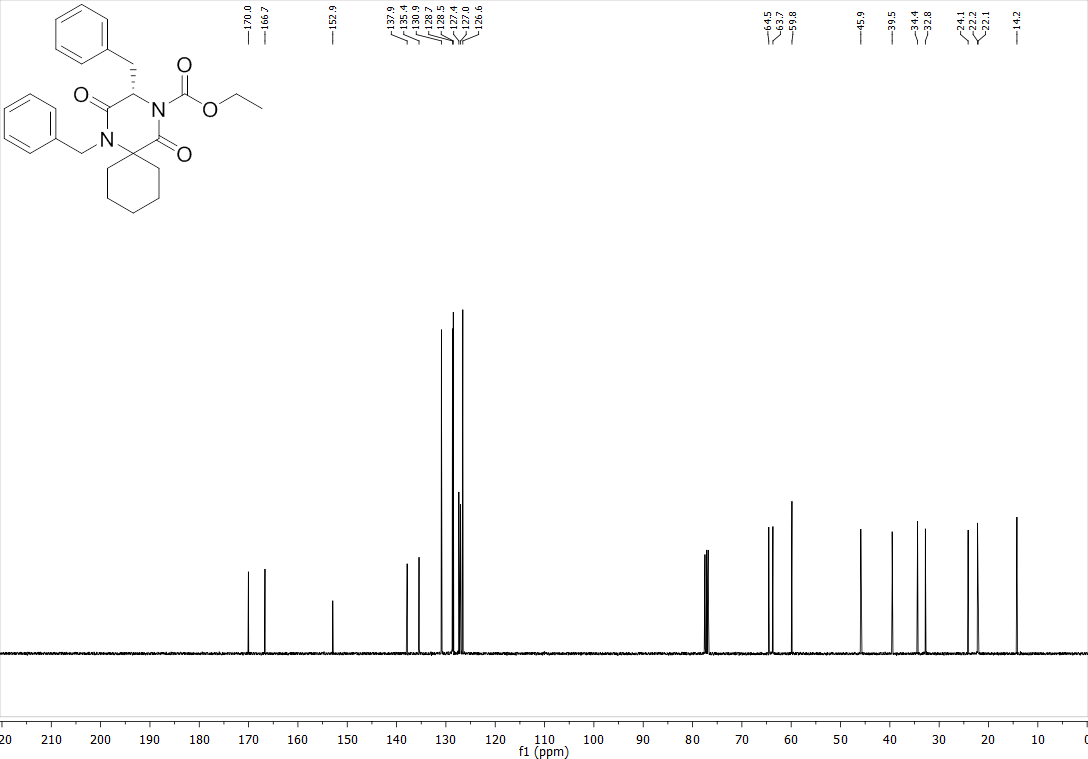


**Figure S83**. ^13^C NMR spectrum of compound **32S**.


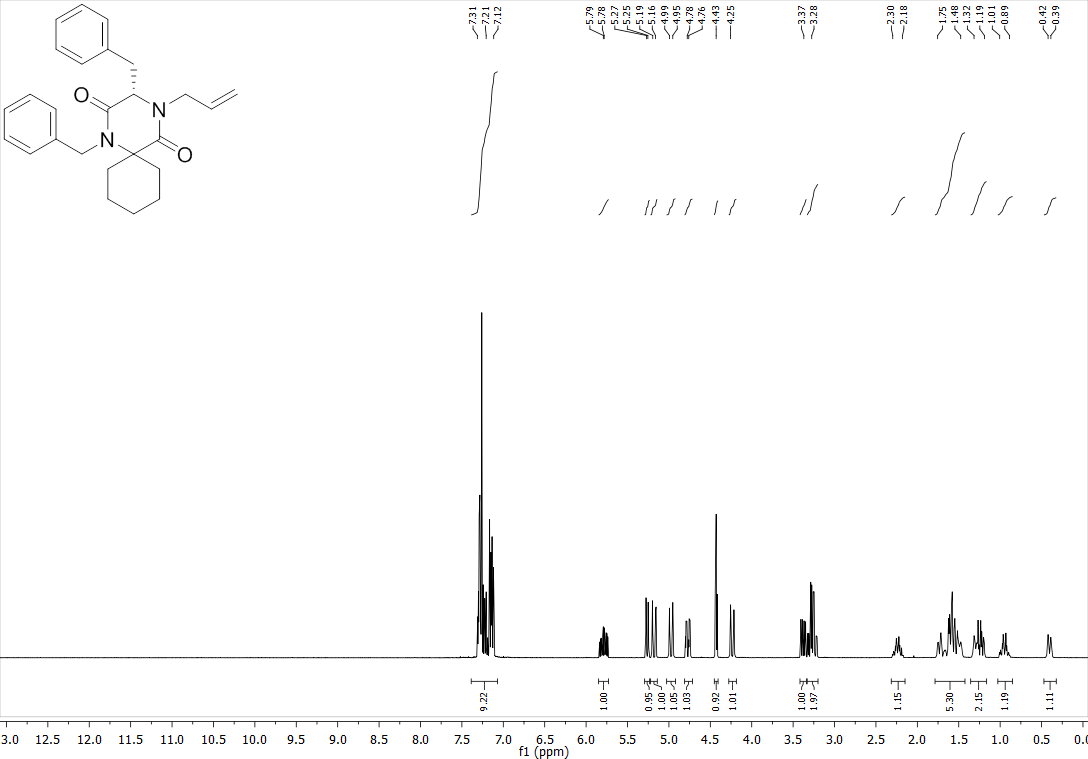


**Figure S84.** ^1^H NMR spectrum of compound **33S**.


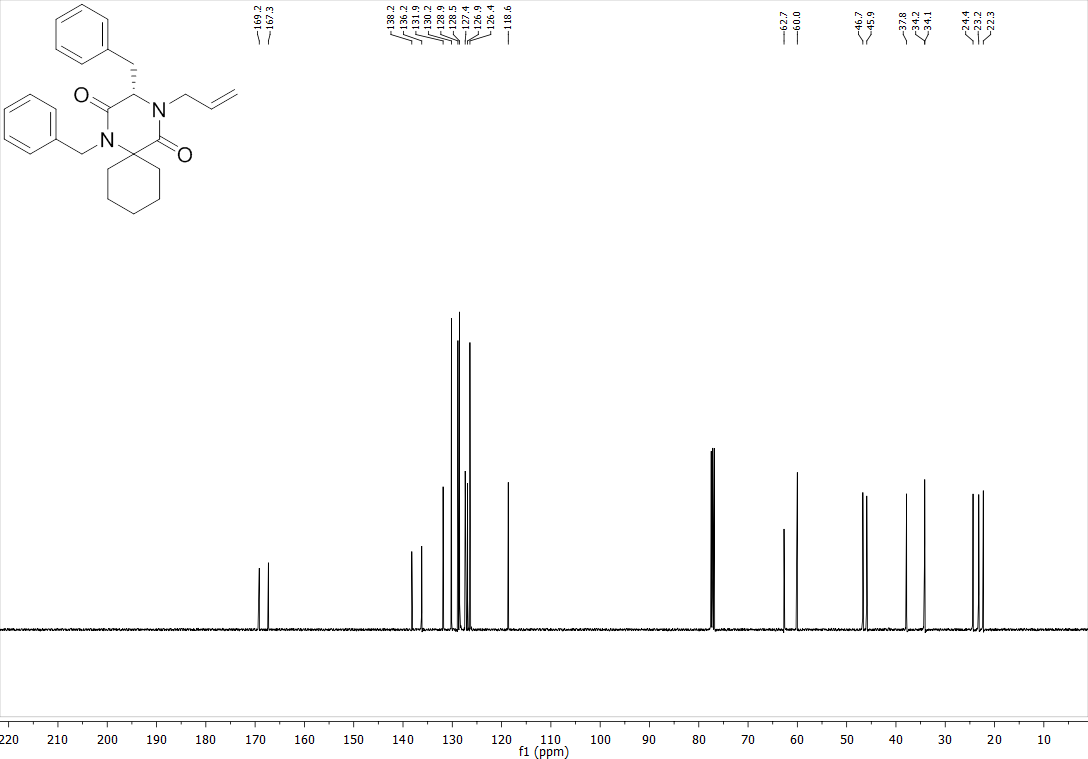


**Figure S85**. ^13^C NMR spectrum of compound **33S**.


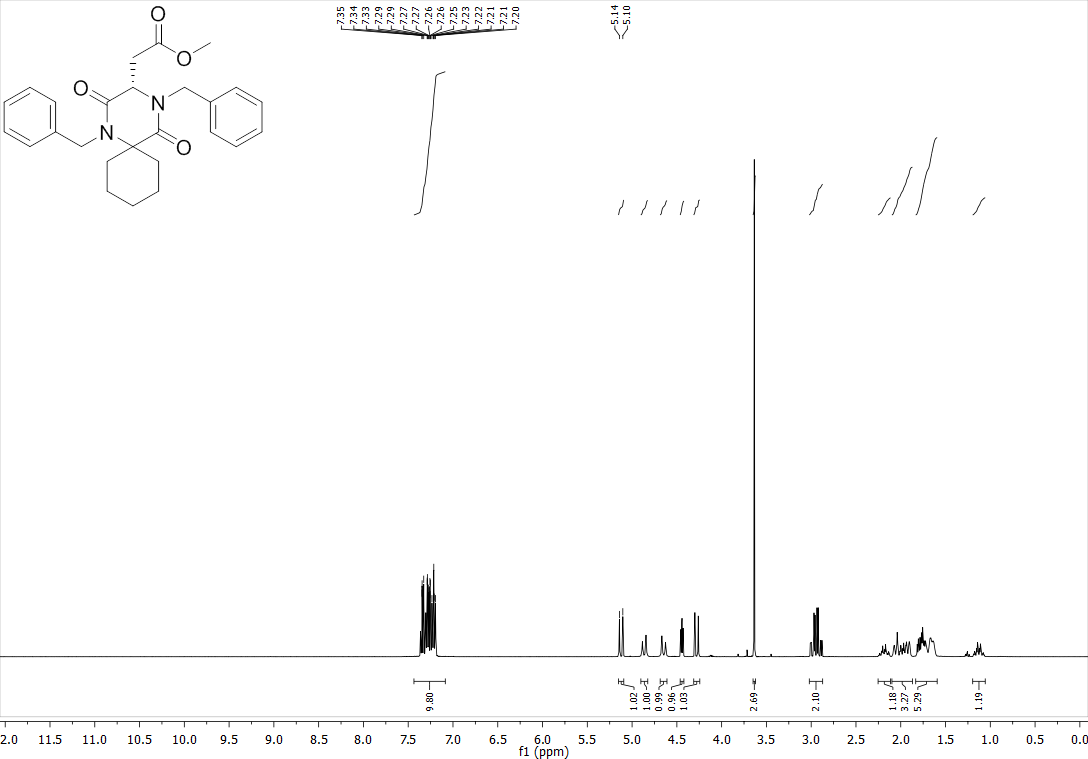


**Figure S86.** ^1^H NMR spectrum of compound **34S**.


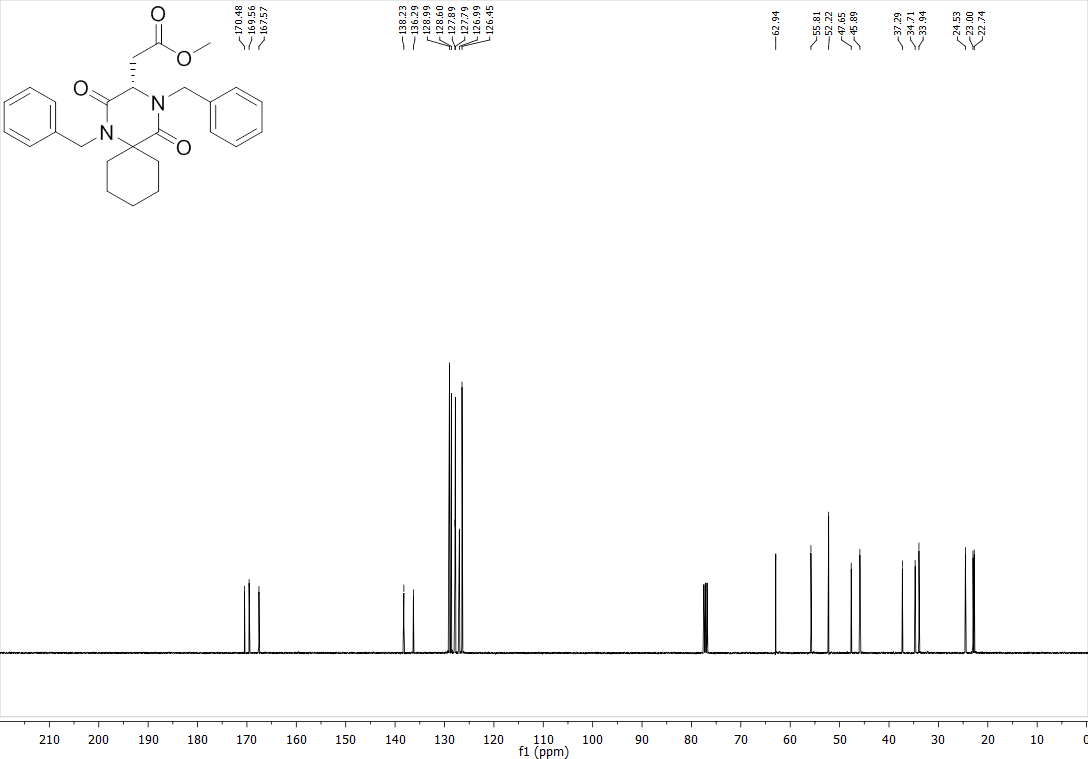


**Figure S87**. ^13^C NMR spectrum of compound **34S**.


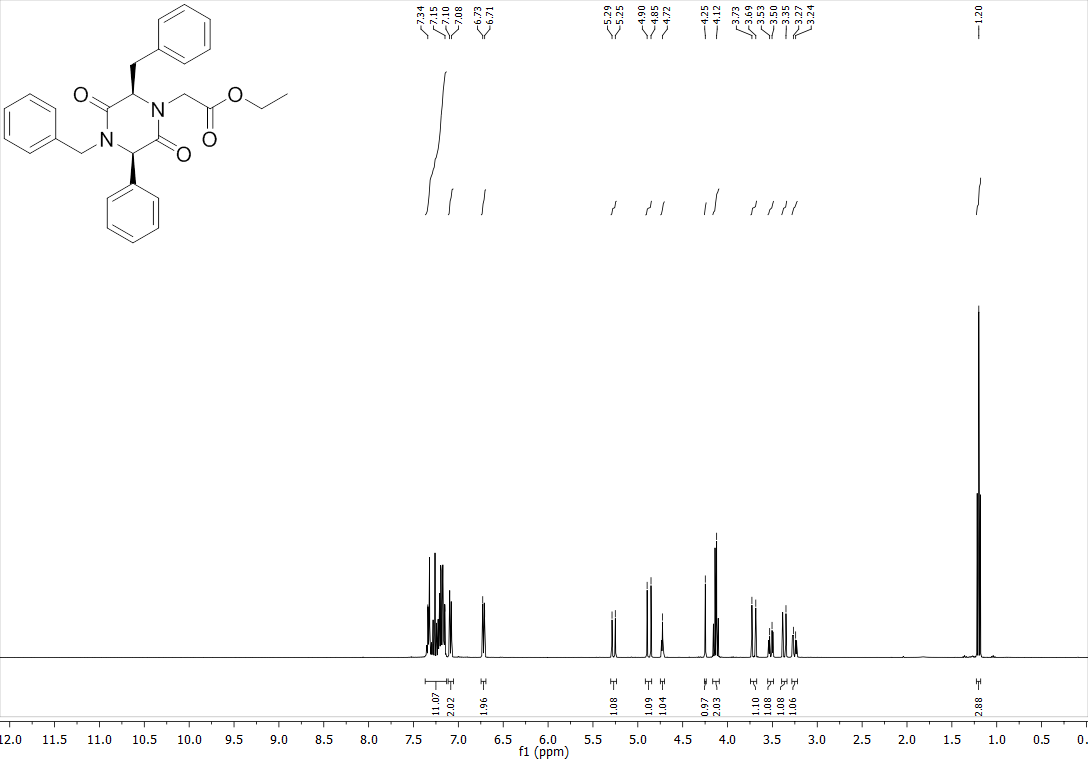


**Figure S88.** ^1^H NMR spectrum of compound **35RR**.


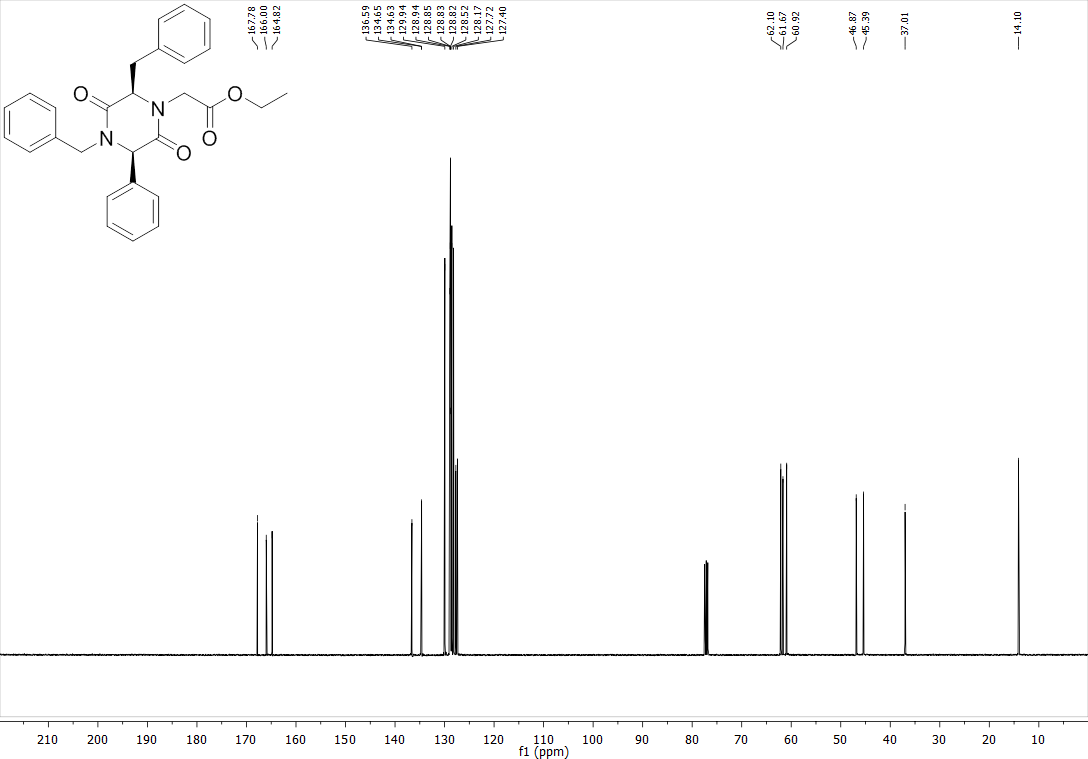


**Figure S89**. ^13^C NMR spectrum of compound **35RR**.


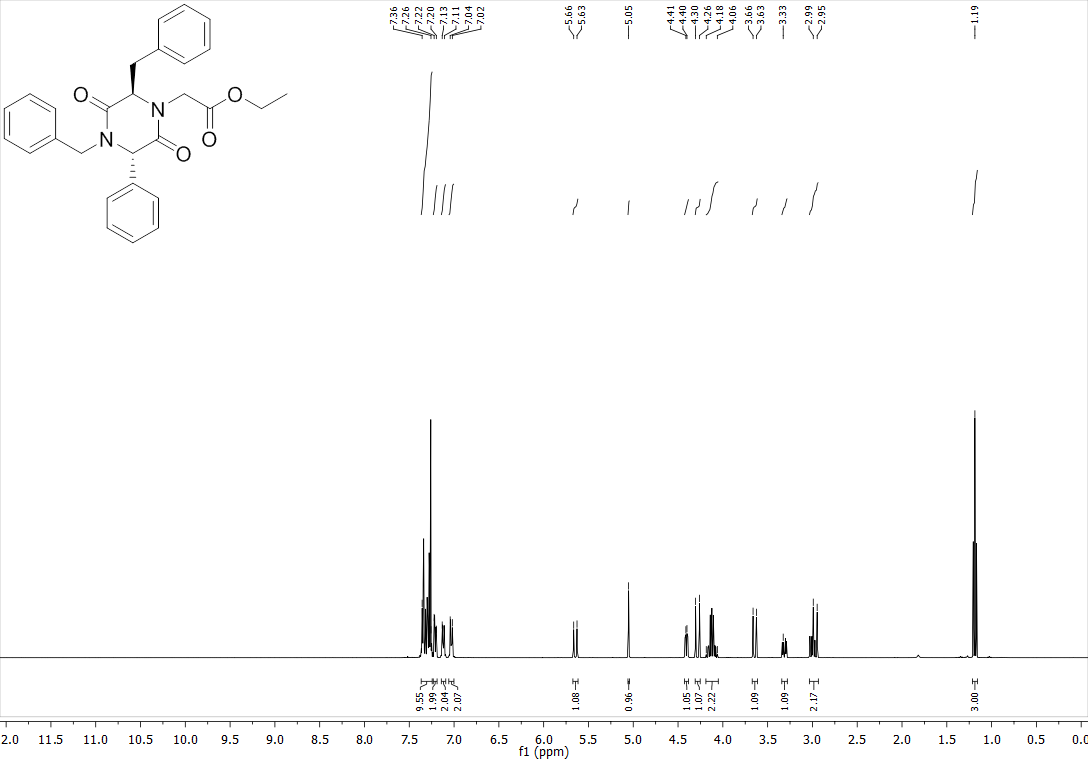


**Figure S90.** ^1^H NMR spectrum of compound **35RS**.


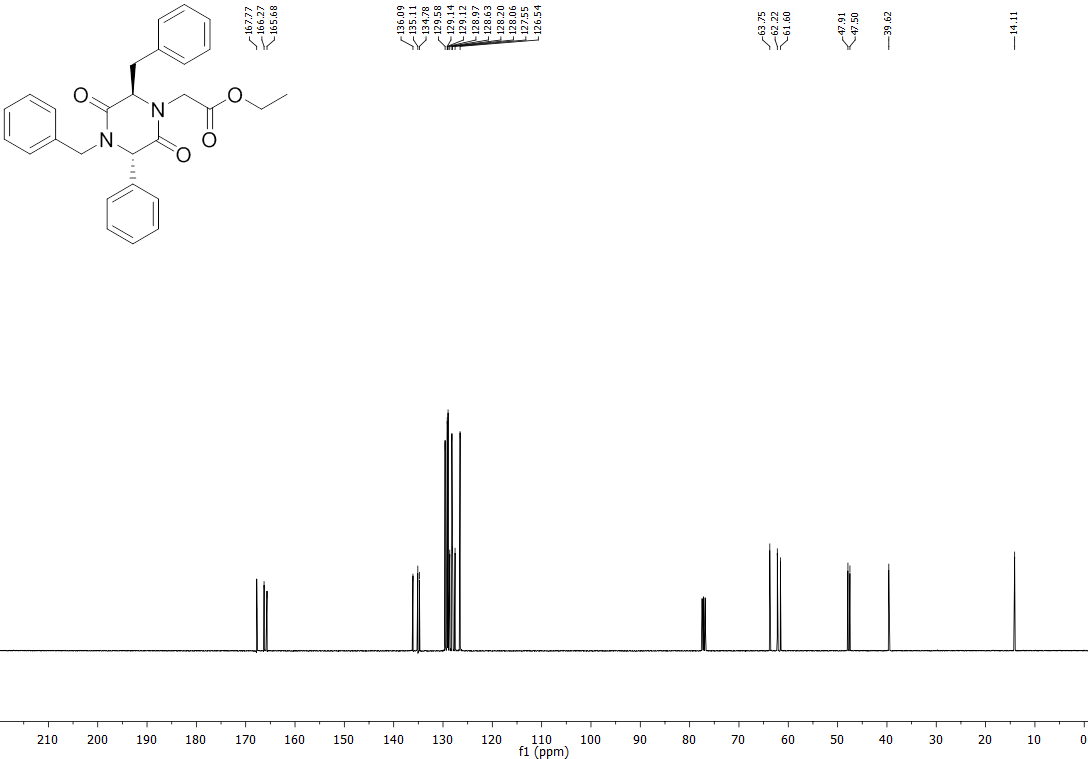


**Figure S91**. ^13^C NMR spectrum of compound **35RS**.


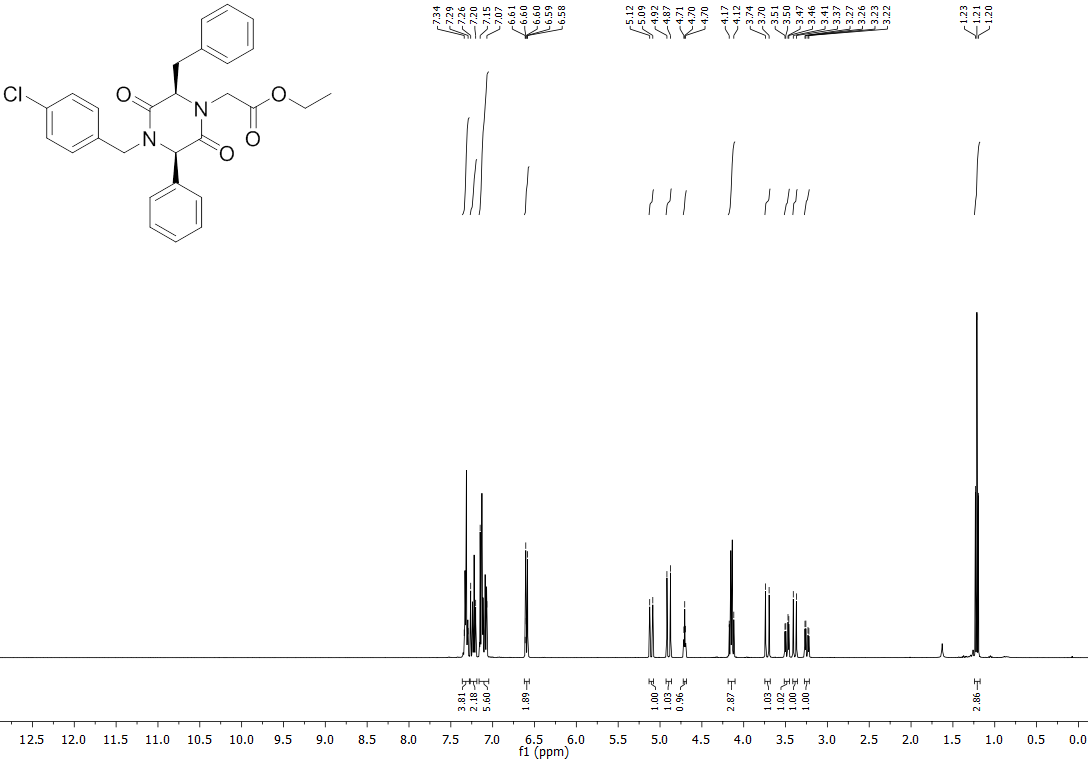


**Figure S92**. ^1^H NMR spectrum of compound **36RR**.


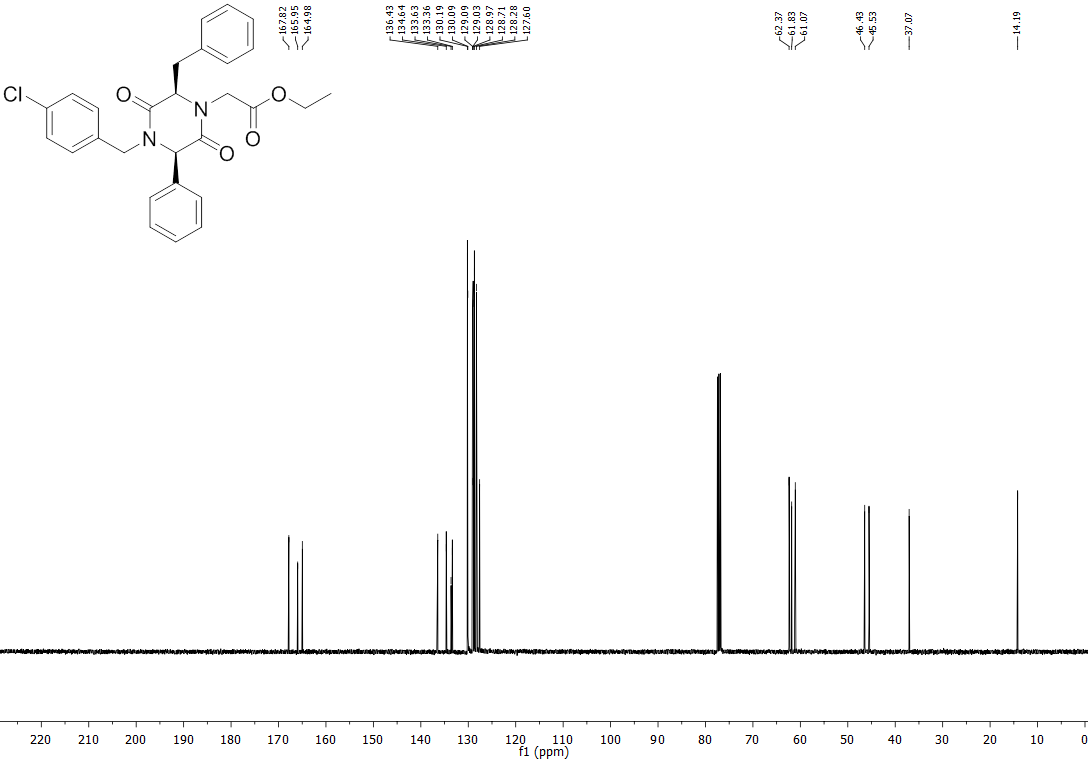


**Figure S93**. ^13^C NMR spectrum of compound **36RR**.


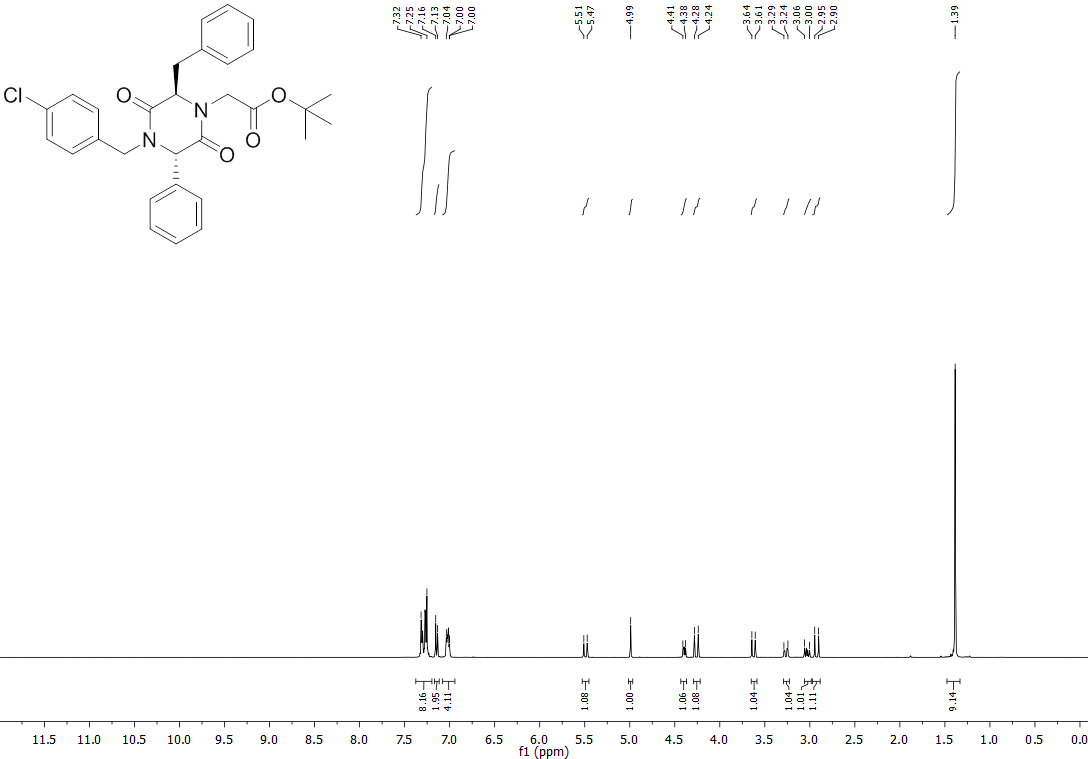


**Figure S94.** ^1^H NMR spectrum of compound **37RS**.

**
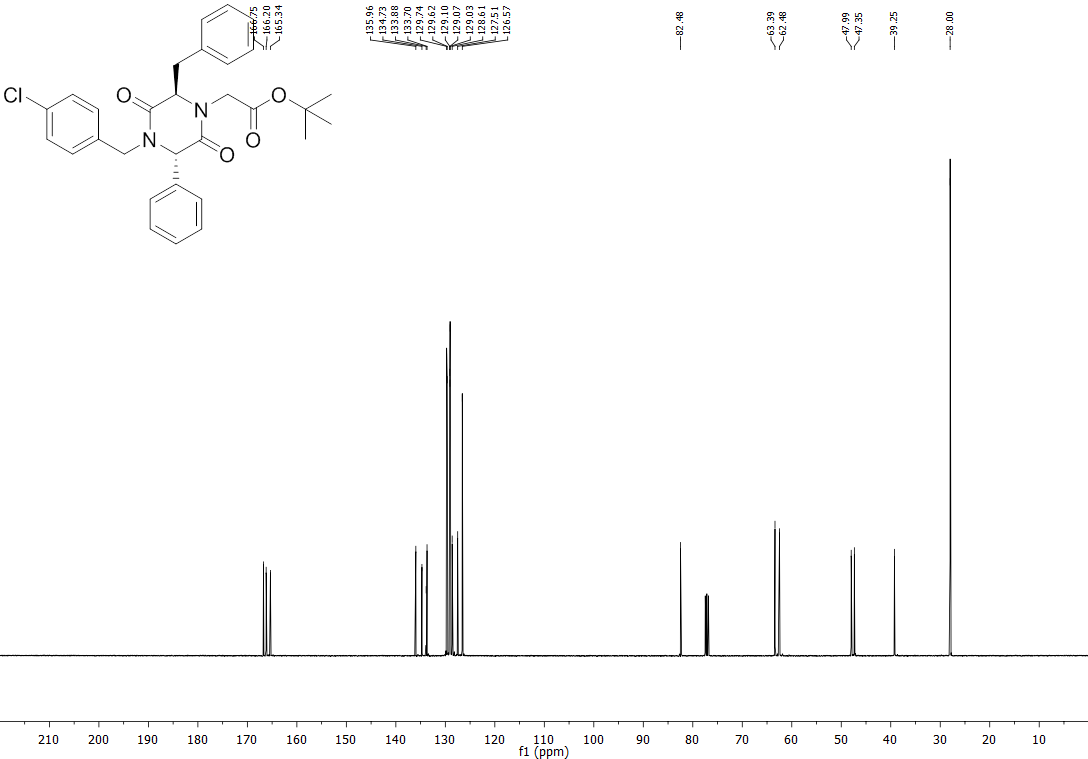
**

**Figure S95**. ^13^C NMR spectrum of compound **37RS**.


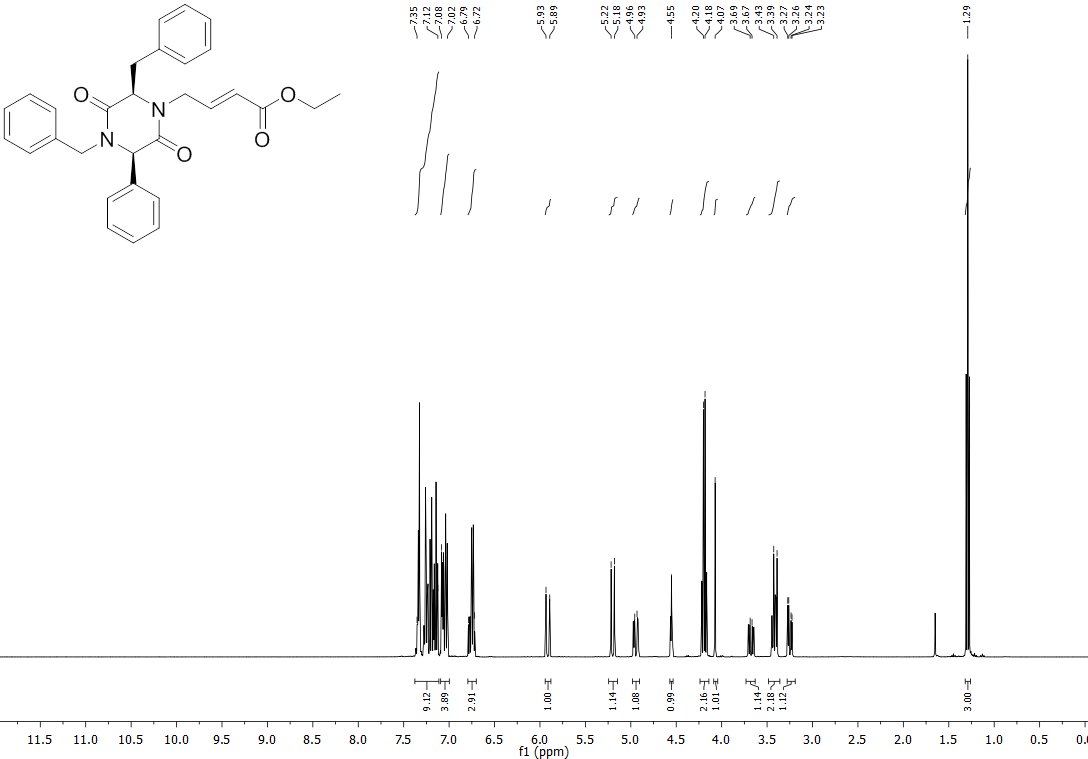


**Figure S96**. ^1^H NMR spectrum of compound **38RR**.


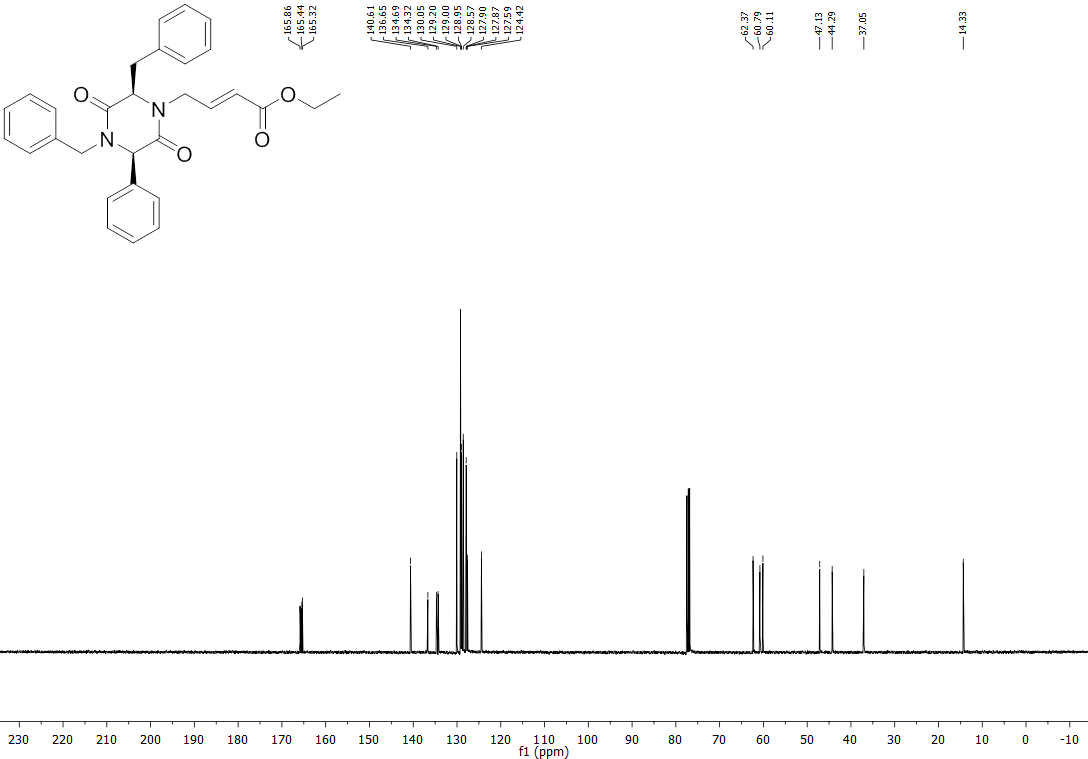


**Figure S97**. ^13^C NMR spectrum of compound **38RR**.


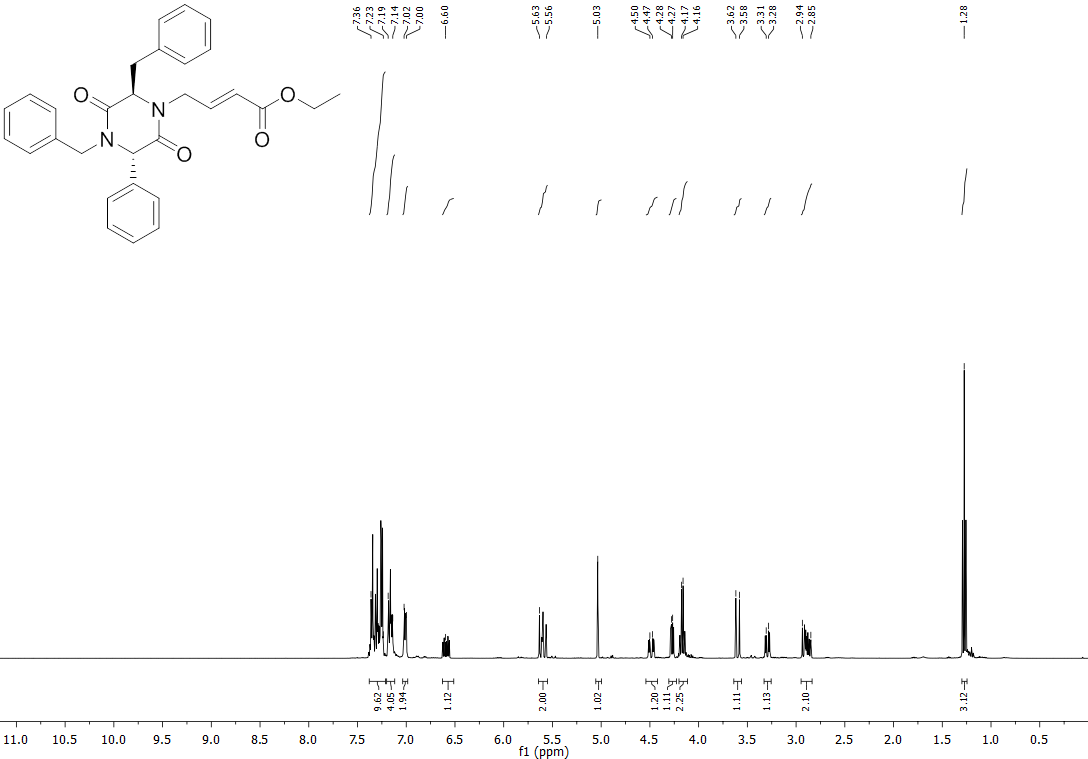


**Figure S98**. ^1^H NMR spectrum of compound **38RS**.


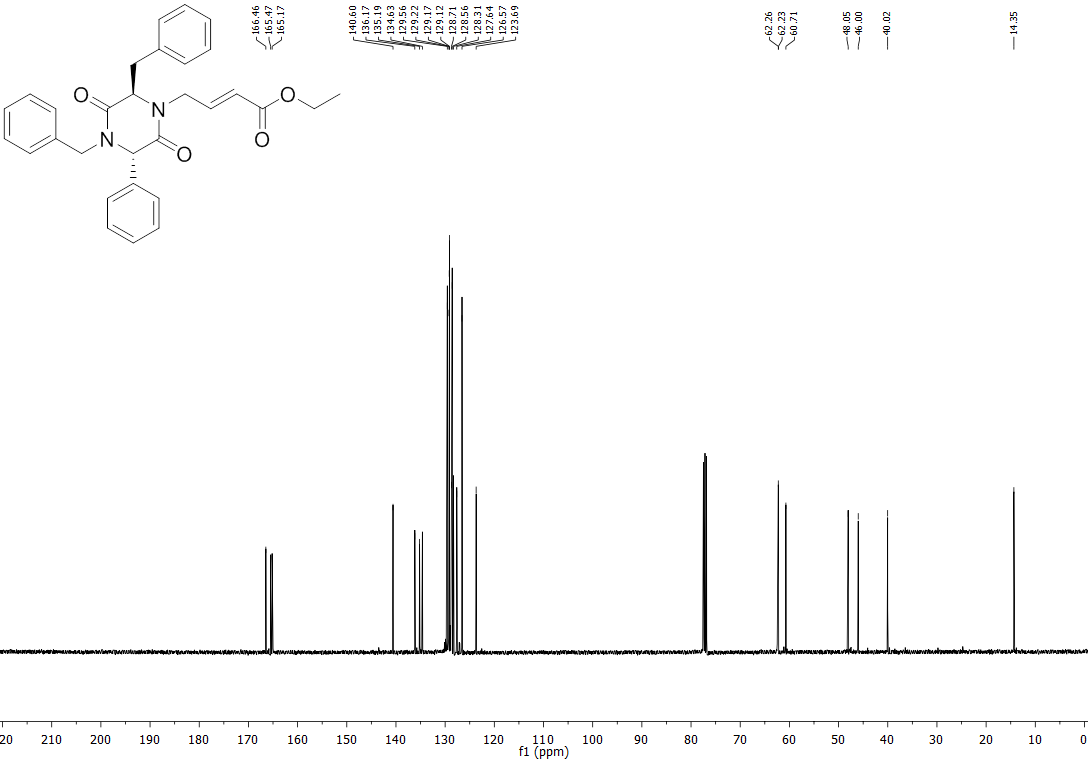


**Figure S99**. ^13^C NMR spectrum of compound **38RS**.


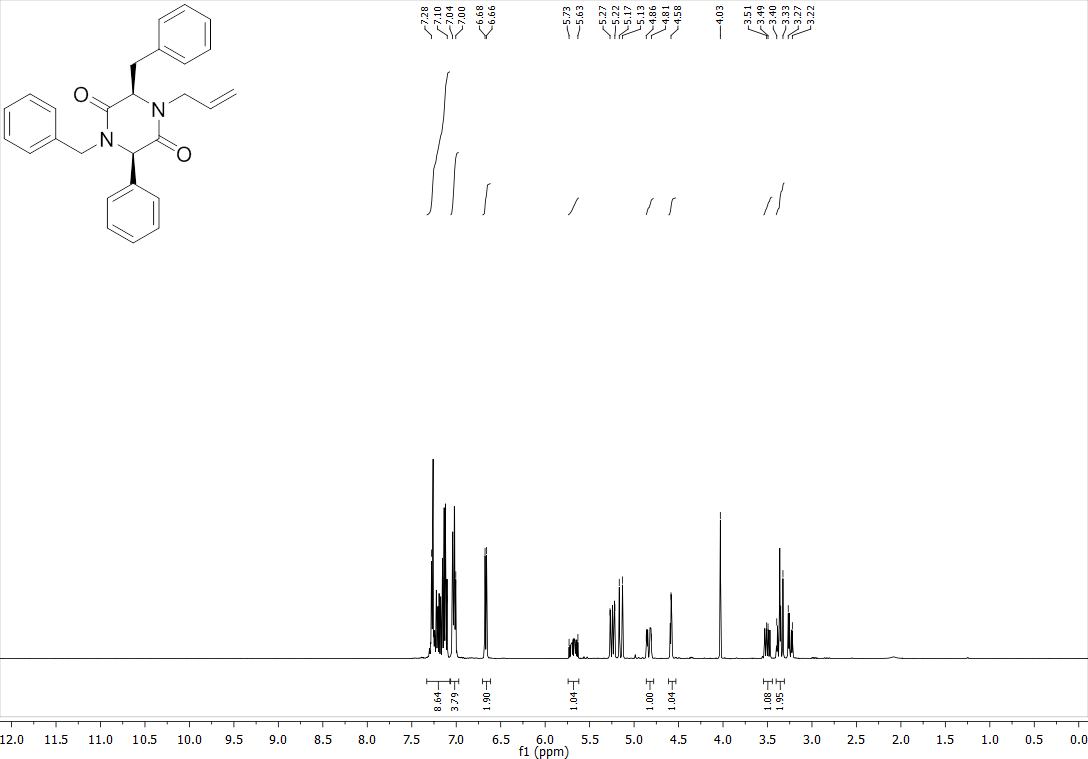


**Figure S100.** ^1^H NMR spectrum of compound **39RR**.


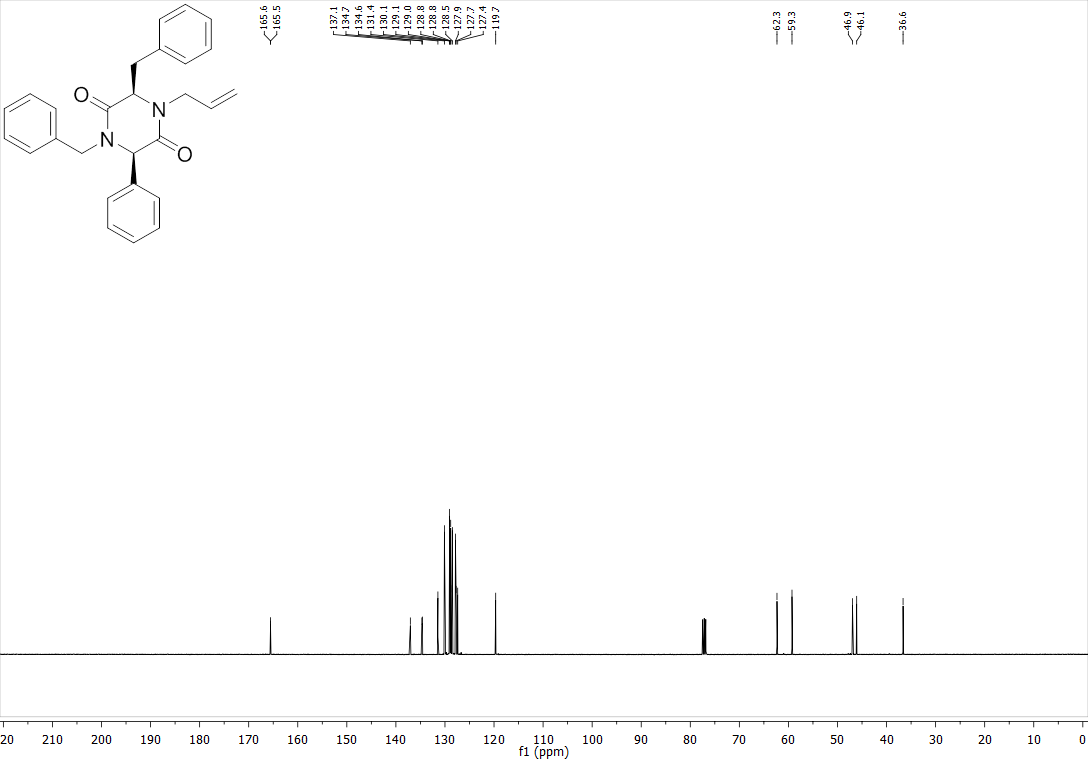


**Figure S101**. ^13^C NMR spectrum of compound **39RR**.

**
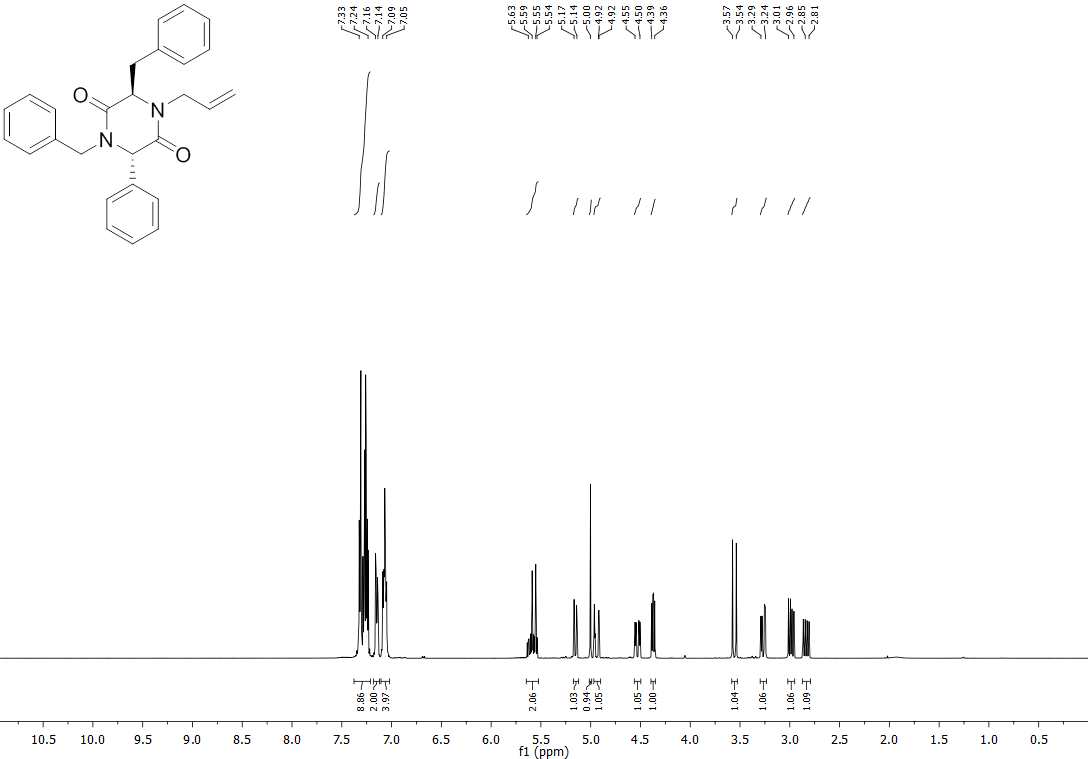
**

**Figure S102.** ^1^H NMR spectrum of compound **39RS**.


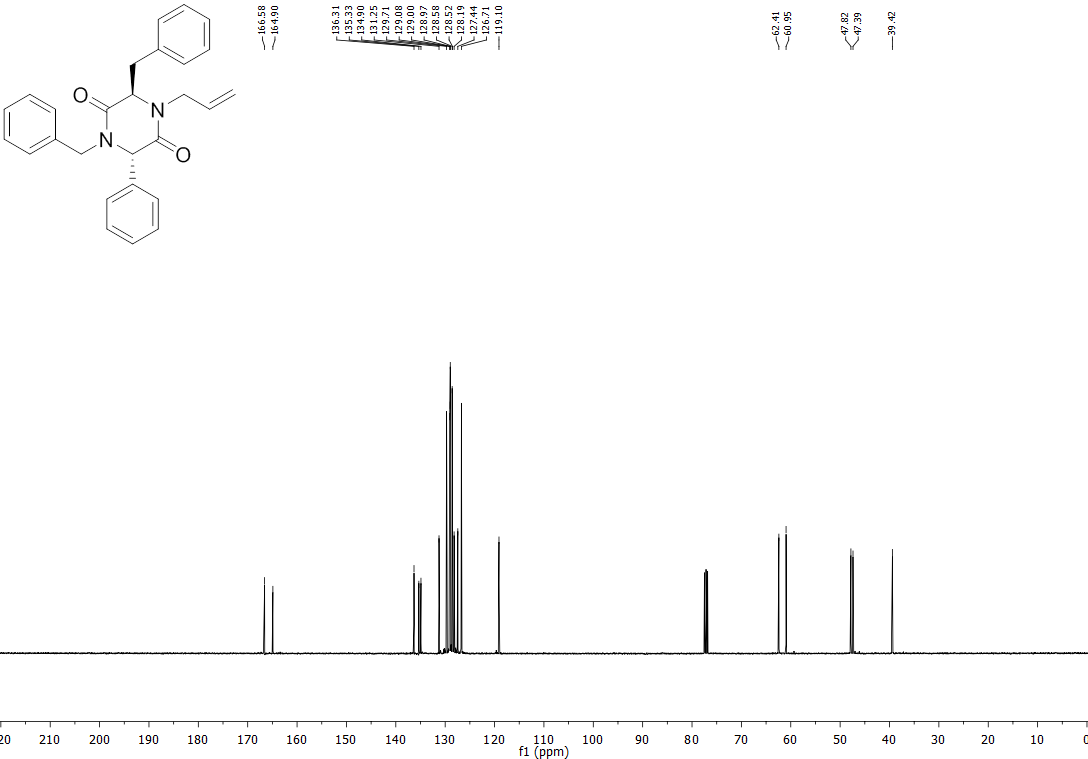


**Figure S103**. ^13^C NMR spectrum of compound **39RS**.


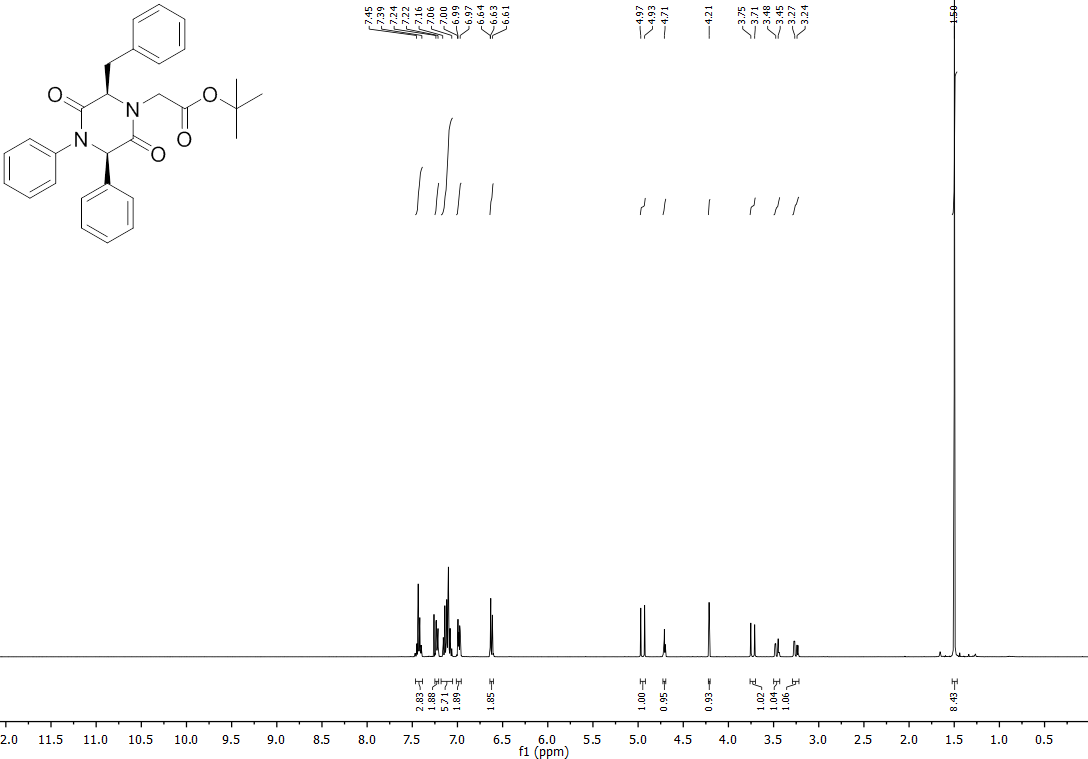


**Figure S104**. ^1^H NMR spectrum of compound **40RR**.


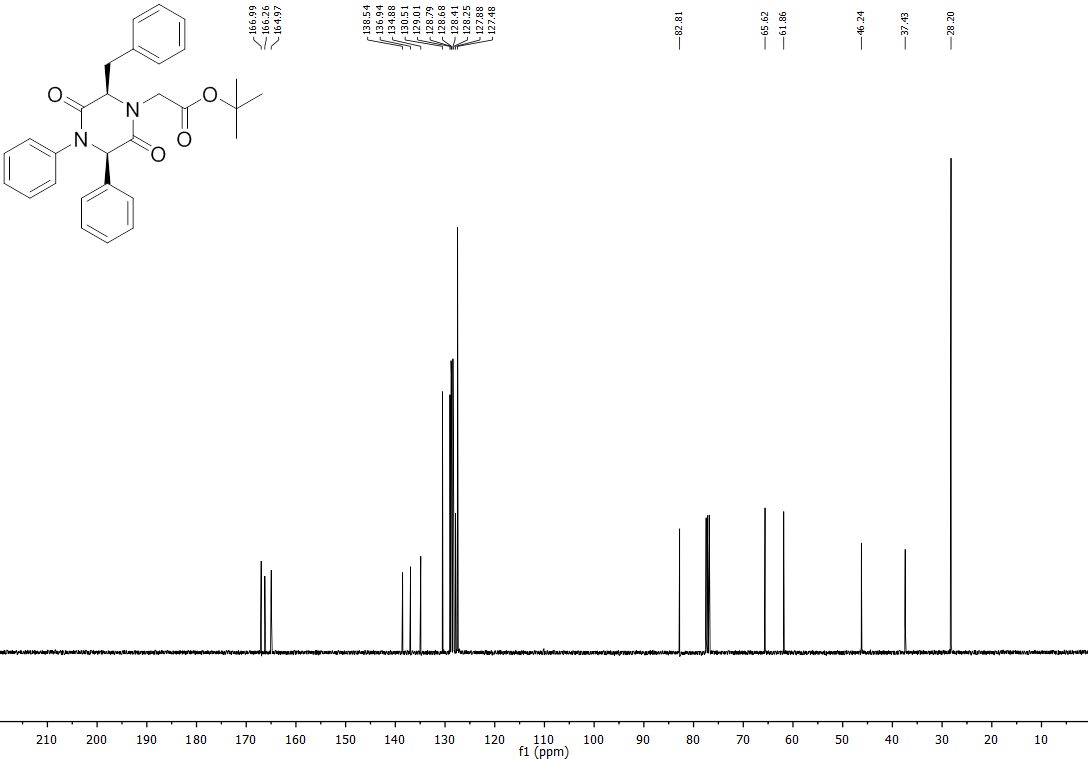


**Figure S105**. ^13^C NMR spectrum of compound **40RR**.

**Figure S106**. ^1^H NMR spectrum of compound **40RS**.

**Figure S107**. ^13^C NMR spectrum of compound **40RS**.

**Figure S108**. ^1^H NMR spectrum of compound **41RR**.

**Figure S109**. ^13^C NMR spectrum of compound **41RR**.

**Figure S110**. ^1^H NMR spectrum of compound **41RS**.

**Figure S111**. ^13^C NMR spectrum of compound **41RS**.

**Figure S112**. ^1^H NMR spectrum of compound **42RR**.

**Figure S113**. ^13^C NMR spectrum of compound **42RR**.

**Figure S114**. ^1^H NMR spectrum of compound **42RS**.

**Figure S115**. ^13^C NMR spectrum of compound **42RS**.

**Figure S116**. ^1^H NMR spectrum of compound **43RR**.

**Figure S117**. ^13^C NMR spectrum of compound **43RR**.

**Figure S118**. ^1^H NMR spectrum of compound **43RS**.

**Figure S119**. ^13^C NMR spectrum of compound **43RS**.

**Figure S120**. ^1^H NMR spectrum of compound **44RR**.

**Figure S121**. ^13^C NMR spectrum of compound **44RR**.

**Figure S122**. ^1^H NMR spectrum of compound **44RS**.

**Figure S123**. ^13^C NMR spectrum of compound **44RS**.

**Figure S124**. ^1^H NMR spectrum of compound **45SS**.

**Figure S125**. ^13^C NMR spectrum of compound **45SS**.

**Figure S126**. ^1^H NMR spectrum of compound **45SR**.

**Figure S127**. ^13^C NMR spectrum of compound **45SR**.

**Figure S128**. ^1^H NMR spectrum of compound **46RR**.

**Figure S129**. ^13^C NMR spectrum of compound **46RR**.

**Figure S130**. ^1^H NMR spectrum of compound **46RS**.

**Figure S131**. ^13^C NMR spectrum of compound **46RS**.

**Figure S132**. ^1^H NMR spectrum of compound **47RR**.

**Figure S133**. ^13^C NMR spectrum of compound **47RR**.

**Figure S134**. ^1^H NMR spectrum of compound **47RS**.

**Figure S135**. ^13^C NMR spectrum of compound **47RS**.

**Figure S136**. ^1^H NMR spectrum of compound **48RR**.

**Figure S137**. ^13^C NMR spectrum of compound **48RR**.

**Figure S138**. ^1^H NMR spectrum of compound **48RS**.

**Figure S139**. ^13^C NMR spectrum of compound **48RS**.

**Figure S140**. gHMBC spectrum of compound **48RS**.

**Figure S141**. ^1^H NMR spectrum of compound **49RR**..

**Figure S142**. ^13^C NMR spectrum of compound **49RR**.

**Figure S143**. ^1^H NMR spectrum of compound **49RS**.

**Figure S144**. ^13^C NMR spectrum of compound **49RS**.

**Figure S145**. ^1^H NMR spectrum of compound **50RS**.

**Figure S146**. ^13^C NMR spectrum of compound **50RS**.

**Figure S147**. ^1^H NMR spectrum of compound **51RR**.

**Figure S148**. ^13^C NMR spectrum of compound **51RR**.

**Figure S150**. ^1^H NMR spectrum of compound **51RS**.

**Figure S151**. ^13^C NMR spectrum of compound **51RS**.

**Figure S152**. ^1^H NMR spectrum of compound **52RR**.

**Figure S153**. ^13^C NMR spectrum of compound **52RR**.

**Figure S154**. ^1^H NMR spectrum of compound **52RS**.

**Figure S155**. ^13^C NMR spectrum of compound **52RS**.

**Figure S156**. ^1^H NMR spectrum of compound **53SS**.

**Figure S157**. ^13^C NMR spectrum of compound **53SS**.

**Figure S158**. ^1^H NMR spectrum of compound **53SR**.

**Figure S159**. ^13^C NMR spectrum of compound **53SR**.

**Figure S160.** ^1^H NMR spectrum of compound **54S**.

**Figure S161**. ^13^C NMR spectrum of compound **54S**.

**Figure S162**. ^1^H NMR spectrum of compound **55RR**.

**Figure S163**. ^13^C NMR spectrum of compound **55RR**.

**Figure S164**. ^1^H NMR spectrum of compound **55RS**.

**Figure S164**. ^13^C NMR spectrum of compound of compound **55RS**.

**Figure S165**. ^1^H NMR spectrum of compound **56RR**.

**Figure S166**. ^13^C NMR spectrum of compound **56RR**.

**Figure S167.** ^1^H NMR spectrum of compound **56RS**.

**Figure S168**. ^13^C NMR spectrum of compound **56RS**.

**Figure S169**. ^1^H NMR spectrum of compound **57RS** at 55 °C.

**Figure S170**. ^13^C NMR spectrum of compound **57RS** at 55 °C.

**Figure S171**. ^1^H NMR spectrum of compound **57RS** at 25 °C.

**Figure S172**. ^13^C NMR spectrum of compound **57RS** at 25 °C.

**Figure S173.** ^1^HNMR signals from protons H7 and H6 of **57RS** at 25 and 55 °C.

**Figure S174.** ^1^H NMR spectrum of compound **58S**.

**Figure S175**. ^13^C NMR spectrum of compound **58S**.

**Figure S176**. ^1^H NMR spectrum of compound **59RR**.

**Figure S177**. ^13^C NMR spectrum of compound **59RR**.

**Figure S178**. ^1^H NMR spectrum of compound **59RS**.

**Figure S179**. ^13^C NMR spectrum of compound **59RS**.

**Figure S180**. ^1^H NMR spectrum of compound **60RR**.

**Figure S181**. ^13^C NMR spectrum of compound **60RR**.

**Figure S182**. ^1^H NMR spectrum of compound **60RS**.

**Figure S183**. ^13^C NMR spectrum of compound **60RS**.

**Figure S184.** ^1^H NMR spectrum of compound **61S**.

**Figure S185**. ^13^C NMR spectrum of compound **61S**.

**Figure S186.** ^1^H NMR spectrum of compound **62S**.

**Figure S187**. ^13^C NMR spectrum of compound **62S**.

# Reference

^1^ Park JD, Lee KJ, Kim, DH. Bioorg. Med. Chem. 2001; 9: 237-243.

^2^ Papalia G. Myszka D. Anal. Biochem. 2010; 403, 30-35.

^3^ Giannetti AM. Methods in Enzymology Ed. Academic Press: 2011; 493: 169-218.

^4^ Myszka DG. J. Mol. Recogn. 1999; 12: 279-284.

^5^MacroModel, version 9.9, Schrödinger, LLC, New York, NY, 2012.

^6^Schrödinger Suite 2012 Protein Preparation Wizard; Epik version 2.3, Schrödinger,

LLC, New York, NY, 2012; Impact version 5.8, Schrödinger, LLC, New York, NY,

2012; Prime version 3.1, Schrödinger, LLC, New York, NY, 2012.

^7^ Nishizawa R, Nishiyama T, Hisaichi K, Minamoto C, Murota M, Takaoka Y, Nakai H, Tada H, Sagawa K, Shibayama S, Fukushima D, Maeda K, Mitsuya H. Bioorg. Med. Chem. 2011; 19: 4028-4042.

^8^ Dalvit C, Fogliatto G, Stewart A, Veronesi M, Stockman B. J. Biomol. NMR. 2001; 21: 349-359.
